# Computational study of the mechanism and selectivity of ruthenium-catalyzed hydroamidations of terminal alkynes†
†Electronic supplementary information (ESI) available: Energy-profiles for dispersion corrected density functionals, optimized geometrical parameters, NPA charges and frontier molecular orbitals of selected intermediates. Cartesian coordinates and absolute energies of all the studied intermediates and transition states. Complete references for ^ref. 37^ and ^ref. 38^. See DOI: 10.1039/c4sc03906h



**DOI:** 10.1039/c4sc03906h

**Published:** 2015-02-18

**Authors:** Bholanath Maity, Lukas J. Gooßen, Debasis Koley

**Affiliations:** a Fachbereich Chemie , TU Kaiserslautern , Erwin-Schrödinger-Straβe 54 , D-67663 Kaiserslautern , Germany . Email: goossen@chemie.uni-kl.de; b Department of Chemical Sciences , Indian Institute of Science Education and Research (IISER) Kolkata , Mohanpur 741246 , India . Email: koley@iiserkol.ac.in

## Abstract

Density functional theory calculations were performed to elucidate the mechanism of the ruthenium-catalyzed hydroamidation of terminal alkynes, a powerful and sustainable method for the stereoselective synthesis of enamides. The results provide an explanation for the puzzling experimental finding that with tri-*n*-butylphosphine (P(Bu)_3_) as the ligand, the *E*-configured enamides are obtained, whereas the stereoselectivity is inverted in favor of the *Z*-configured enamides with (dicyclohexylphosphino)methane (dcypm) ligands. Using the addition of pyrrolidinone to 1-hexyne as a model reaction, various pathways were investigated, among which a catalytic cycle turned out to be most advantageous for both ligand systems that consists of: (a) oxidative addition, (b) alkyne coordination, (c) alkyne insertion (d) vinyl-vinylidene rearrangement, (e) nucleophilic transfer and finally (f) reductive elimination. The stereoselectivity of the reaction is decided in the nucleophilic transfer step. For the P(^*n*^Bu)_3_ ligand, the butyl moiety is oriented *anti* to the incoming 2-pyrolidinyl unit during the nucleophilic transfer step, whereas for the dcypm ligand, steric repulsion between the butyl and cyclohexyl groups turns it into a *syn* orientation. Overall, the formation of *E*-configured product is favorable by 4.8 kcal mol^–1^ (Δ^‡^ *G*SDL) for the catalytic cycle computed with P(Bu)_3_ as ancillary ligand, whereas for the catalytic cycle computed with dcypm ligands, the *Z*-product is favored by 7.0 kcal mol^–1^ (Δ^‡^ *G*SDL). These calculations are in excellent agreement with experimental findings.

## Abstract 2

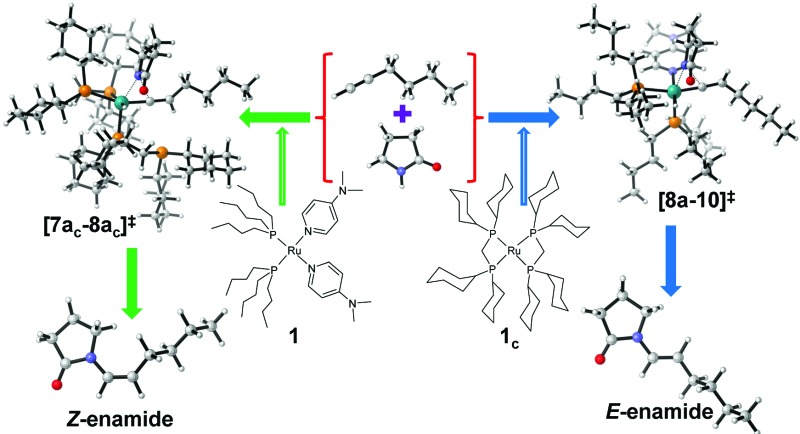
Density functional theory calculations were performed to elucidate the mechanism of the ruthenium-catalyzed hydroamidation of terminal alkynes, a powerful and sustainable method for the stereoselective synthesis of enamides.

## A. Introduction

The enamide moiety is a key functionality in numerous natural products^1^ and synthetic drugs exhibiting antibiotic,^2^ antitumor,^3^ cytotoxic,^4^ anthelmintic^5^ and antifungal^6^ activities. This class of compounds also serves as intermediates in various reactions including synthesis of heterocycles,^7^ [4 + 2]-cycloadditions,^8^ cross-coupling reactions,^9^ Heck olefinations,^10^ enantioselective additions^11^ or asymmetric hydrogenations.^12^

Established methods for syntheses of enamides all have individual drawbacks, which complicate their use in organic synthesis. Condensations of carbonyl derivatives with amides^13^ require rigorous reaction conditions and lead to mixtures of *E*- and *Z*-enamides. The isomerization of *N*-allylamides,^14^ oxidative amidation of alkenes,^15^ and co-dimerization of *N*-vinyl amides with alkenes^16^ all furnish the thermodynamically favorable *E*-isomer as the major product. Only few synthetic methods, such as the Curtius rearrangement of α,β-unsaturated acyl azides,^17^ Peterson elimination,^18^ transition metal-catalyzed cross-coupling reactions of vinyl halides,^19^ vinyl triflates,^20^ or vinyl ethers,^21^ are applicable for the synthesis of the thermodynamically less favorable *Z*-isomer, but here, the starting materials are poorly available and expensive.

**Scheme 1:**
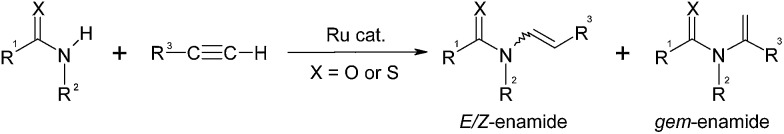
Nucleophilic addition of amides to terminal alkynes.

**Scheme 2:**
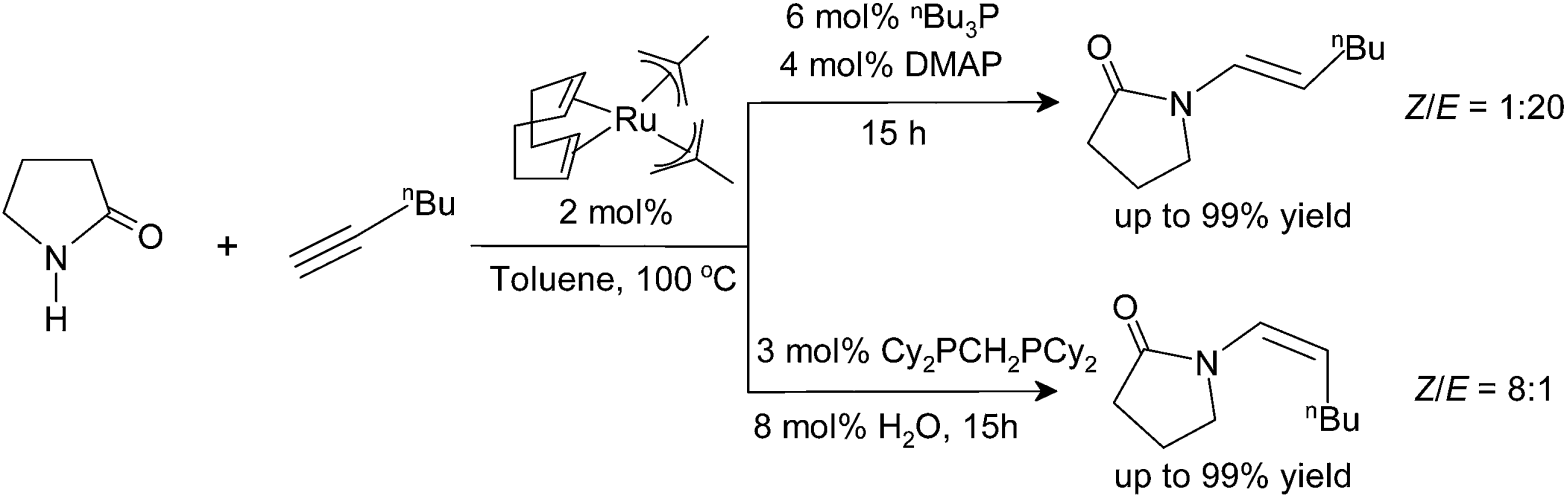
Ru(ii)-catalyzed addition of cyclic amides to terminal alkynes for different ligand and additive systems.

In 1983, Shvo *et al.* ^22^ reported the synthesis of enol esters using a ruthenium-catalyzed nucleophilic addition of carboxylic acids to non-activated alkynes. Thereafter, additions of other nucleophiles such as water,^23^ amines,^24^ alcohols,^25^ thiols,^26^ *etc.* have been investigated by several groups. In 1995, the groups of Heider^27^ and Watanabe^28^ observed that *E*-enamides can be prepared stereoselectively by ruthenium-catalyzed *anti*-Markovnikov addition of carboxamides to terminal alkynes. This was the first report of a transition metal-catalyzed N–H bond activation and addition of amides to alkynes. Based on this pioneering work, Gooßen *et al.* have developed efficient ruthenium catalysts for the atom-economic addition of amides, carbamates, lactams,^29^ imides^30^ and thioamides^31^ to terminal alkynes (Scheme 1 and 2). Recent work in this area includes, *e.g.* the stereoselective addition of alkynes to pyrazoles.^32^

The catalyst systems are generated *in situ* from bis(2-methallyl)(cycloocta-1,5-diene)ruthenium(ii) [(cod)Ru(met)_2_], phosphine ligands, and bases or Lewis acids. In all cases, the reaction proceeds highly regioselectively in favor of the *anti*-Markovnikov products.^33^ The stereoselectivity is controlled by the choice of ligands: with a combination of tri-*n*-butylphosphine (P(Bu)_3_) and 4-dimethylaminopyridine (DMAP), the *E*-enamides are obtained in high yield and selectivity. With bis(dicyclohexylphosphino)methane (dcypm), the stereoselectivity is inverted, and the *Z*-enamides are preferentially formed (Scheme 2).^34^

The dependence of the stereoselectivity on the catalyst system and the reaction conditions is a key advantage of this synthetic approach, as it allows accessing both stereoisomers from the same precursors. However, all efforts to pinpoint the origin of this effect have failed so far.

Various catalytic pathways have been considered for ruthenium-catalyzed hydroamidation and related additions of nucleophiles such as carboxylic acids, alcohols and water to C–C triple bonds. Watanabe initially proposed a mechanism that involves oxidative addition of the amide, insertion of the alkyne into the Ru–H or Ru–N bond and reductive elimination.^28^ A similar pathway was sketched out by Uchimaru for the ruthenium-catalyzed addition of aromatic amines to alkynes, which, however, proceeds with Markovnikov selectivity.^24*a*^ In both pathways, the alkyne insertion step was believed to control the regioselectivity of the addition process.

Dixneuf proposed that the key mechanistic step is the formation of a vinylidene intermediate *via* 1,2 or 1,3-proton transfer at the alkyne moiety.^35^ The electrophilic nature of the α-carbon in vinylidenes would explain the exclusive formation of the *anti*-Markovnikov products, and the required rearrangement the limitation of this reaction to terminal alkynes. Caulton and co-workers performed computational studies to investigate pathways of ruthenium–vinylidene complex formation.^36^ Their key message is that the formation of vinylidenes *via* 1,2 or 1,3-proton transfer is energetically difficult. In contrast, ruthenium–vinylidene complexes easily form *via* rearrangement of the corresponding vinyl complexes, which themselves are easily generated by the insertion of π-coordinated alkynes into Ru–H bonds. A related vinyl-vinylidene rearrangement pathway was also proposed by Wakatsuki for the hydration of alkynes.^23*b*^

**Scheme 3:**
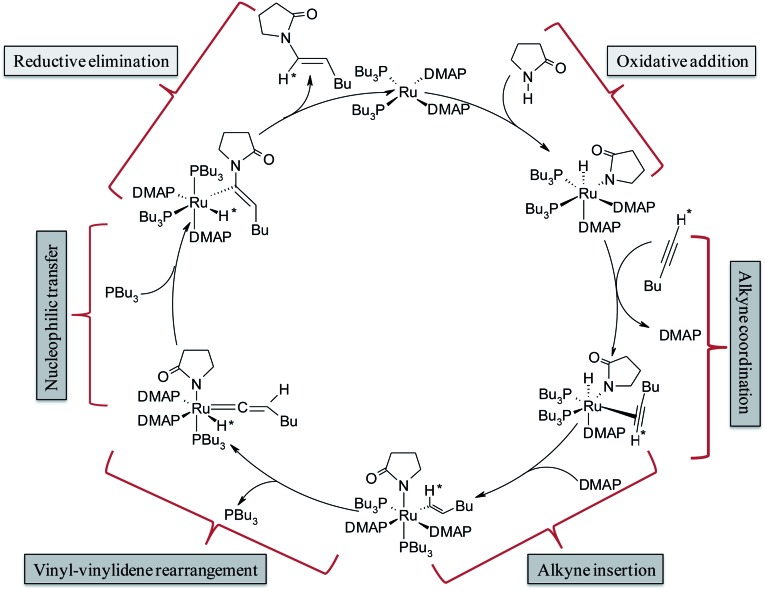
Catalytic cycle for hydroamidation reactions as proposed by Gooßen *et al.* ^37^

The complete array of potential mechanistic pathways was evaluated by Gooßen *et al.* with regards to their applicability to the hydroamidation of terminal alkynes with secondary amides.^37^ Extensive experimental and computational data including mass spectrometry, DFT optimizations of intermediates, deuterium labeling studies and NMR spectra were used to narrow down the possible mechanistic pathways to the route delineated in Scheme 3. The cycle starts with the Ru^0^ species [Ru^0^(DMAP)_2_(PBu_3_)_2_], which was confirmed by strong signals in the *in situ* ESI-MS experiments. It is followed by the oxidative addition of amide giving rise to an octahedral Ru^II^–hydride complex [Ru^II^(DMAP)_2_(PBu_3_)_2_(H)(pyr)] (pyr = 2-pyrrolidinyl anion). The NMR signals of the reaction mixture correspond to those observed for a mixture of the Ru^II^–hydride complex with amide, but not to those for a mixture of the Ru^II^–hydride complex with alkyne.^35^ Subsequent coordination and insertion of hexyne to the Ru–H bond results in the Ru^II^–vinyl complex [Ru^II^(DMAP)_2_(PBu_3_)_2_(CH*=CHBu)(pyr)] (Scheme 3). The insertion step leading to the vinyl intermediate was substantiated with ESI-MS peaks and KIE (kinetic isotope effect) values measured in presence of deuterated alkynes. Subsequently, a 1,2-hydride shift in the vinyl intermediate will provide the Ru^IV^–H–vinylidene complex [Ru^II^(DMAP)_2_(PBu_3_)(H*)(=C=CHBu)(pyr)] with loss of one phosphine ligand. The electrophilic center at C^α^ provides an ideal electronic environment for the amide to attack, giving rise to the intermediate [Ru^II^(DMAP)_2_(PBu_3_)_2_(H*)(C(pyr)=CHBu)]. Finally, reductive elimination releases the product and regenerates the Ru^0^ catalyst.

This mechanism serves well to explain the regioselectivity of the reaction. However, these extensive spectroscopic investigations do not provide any explanation for the strong dependence of the stereoselectivity on the phosphine employed since they suggest that the same intermediates are present both for P(Bu)_3_ and dcypm as phosphine ligands although they led to products with the opposite stereochemistry.^37^

In-depth theoretical studies were clearly required to gain an understanding why the stereoselectivity of this reaction can so efficiently be controlled by the ligand. Only if the origin of this effect can be understood, a rational development of highly efficient catalyst systems for both *E*- and *Z*-selective addition reactions of amides and related nucleophiles to alkynes will become possible.

In the theoretical studies disclosed herein, the entire catalytic cycle of the hydroamidation of amides to terminal alkynes has been computed using realistic model systems of high complexity. The calculations confirm that the catalytic cycle that has been proposed based on the spectroscopic studies is viable and has a realistic energy profile. They also reveal that the stereochemistry of the reaction is determined by the preferred geometry of the vinylidene intermediate that undergoes the intramolecular nucleophilic transfer step: in presence of P(Bu)_3_, the butyl chain is in *anti*-orientation to the incoming 2-pyrolidinyl unit, while the steric interaction between the cyclohexyl groups in the dcypm ligands and the butyl chain forces it into the opposite orientation. Consequently, *E*-products are predicted to form in the presence of P(Bu)_3_, whereas *Z*-products should predominate when using dcypm. This is in excellent agreement with the experimental findings.

## B. Computational details

**Fig. 1:**
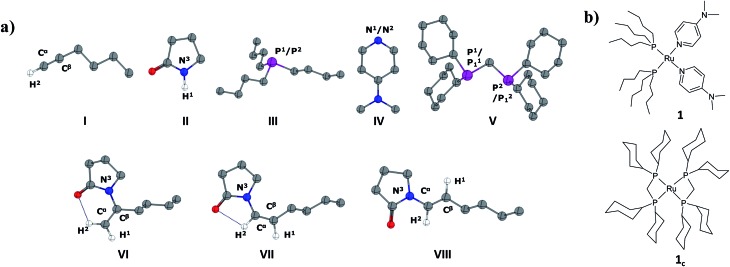
(a) Optimized geometry of reactants (**I**, **II**), ligands (**III**, **IV**, **V**) and possible products (**VI**, **VII**, **VIII**) at the BP86/LANL2DZ(Ru)/6-31G*(H, C, N, O and P) level. All hydrogen atoms (except H^1^ and H^2^) are omitted for clarity. Color code: C: grey; P: purple; N: blue; O: red. (b) Chemical structures for **1** and **1_c_** (refer to text).

All the calculations were performed using Gaussian03 (ref. 38) and Gaussian09 (ref. 39) program packages. The geometries of stationary points and transition states were optimized with the generalized gradient approximation (GGA) by means of the Becke exchange functional^40^ in addition with the Perdew correlation functional^41^ (BP86). We employed Double-ζ basis set with the relativistic effective core potential of Hay and Wadt (LANL2DZ)^42^ for the ruthenium atom and 6-31G(d)^43^ basis sets for other elements (H, C, N, O, and P). To reduce computational cost, all calculations were performed using a two-layer ONIOM(MO:MO)^44^ method for reaction pathways emanating from the cyclic phosphine-containing catalyst system **1_c_**. The ONIOM high level was designated to the whole molecules except the cyclohexyl ring (–Cy) in dcypm (**V** in Fig. 1) and treated at a BP86/LANL2DZ(Ru)/6-31G*(H, C, N, O and P) level. The ONIOM low level (–Cy) has been described at the HF/STO-3G method. For all our DFT calculations, the resolution-of-the-identity (RI) approximation (also called “density fitting”) was employed for the two-electron integrals.^45^ The geometries were optimized without any symmetry constraints. Harmonic force constants were computed at the optimized geometries to characterize the stationary points as minima or saddle points. Zero-point vibrational corrections were determined from the harmonic vibrational frequencies to convert the total energies *E*_e_ to ground state energies *E*_0_. The rigid-rotor harmonic-oscillator approximation was applied for evaluating the thermal and entropic contributions that are needed to derive the enthalpies *H*_298_ and Gibbs free energies, *G*_298_ at 298 K. All transition states were located using the linear synchronous transit (LST)^46^ method in which the reaction coordinate was kept fixed at different distances while all other degrees of freedom were relaxed. After the linear transit search the transition states were optimized using the default Berny algorithm implemented in the Gaussian09 code.^39^ All transition states were confirmed by IRC (Intrinsic Reaction Coordinate) calculations. For further validation, single-point BP86 calculations (*E*_L_) were performed on the BP86/LANL2DZ(Ru)/6-31G*(H, C, N, O and P) optimized geometries employing a valence triple-ζ-type of basis set (TZVP)^47^ for light atoms (H, C, N, O, and P) and LANL2TZ(f)^42^ basis set for ruthenium incorporated in the Gaussian program suites.^39^ It has been shown that the current DFT method (with BP86 functional) provides reliable geometries, energies and vibrational frequencies in related mechanistic studies.^48^ Using this functional, validation studies of transition metal compounds, particularly ruthenium(ii) complexes, are reported in the literature. Additionally, Tonner and Frenking studied the effect of carbodiphosphorane ligands in olefin metathesis using Grubbs catalyst at BP86/SVP level of theory.^49^ To account for dispersion effects, single-point calculations were performed using the empirical dispersion-corrected BP86-D^50^ functional using the larger basis set employed for calculating *E*_L_ energies. For further validation in computing dispersion effects, we also obtained single-point energies with functionals B97D^50^ and M06-2X^51^ using the same larger basis sets described above (refer to ESI, Fig. S1 and S2†). We have tested several dispersion corrected functionals, *e.g.* BP86-D, B97D and M06-2X. The empirical correction for dispersion interactions results in a significant reduction of the energies. These attractive forces are not considered in pure DFT methods. The variations between the methods compared result from the different way these treat exchange–correlation in the functionals. However, potential mistakes will similarly affect related catalytic pathways, so that the overall effect of systematic errors in this context is low. Solvation energies (*E*SL) were evaluated by a self-consistent reaction field (SCRF) approach for all the intermediates and transitions states, using the SMD continuum solvation model^52^ implemented in Gaussian09. Toluene was chosen as a solvent (dielectric constant *ε* = 2.374) with SMD-intrinsic Coulomb radii for the respective atoms. In the Gaussian program, the concentration can be specified by adjusting the pressure value based on the ideal gas law *p*_i_ = *RTn*_i_/*V*, where *p*_i_ is the pressure, *R* the gas constant, *T* the absolute temperature, *n*_i_ the molar quantity, and *V* the reaction volume. The experimental concentrations of catalyst and reactants at the reaction temperature are approximated by setting the partial pressures of the substrates as follows: alkyne (**I**): 2.0 × 10^–3^ mol ≙ 20 atm; amide (**II**): 1.0 × 10^–3^ mol ≙ 10 atm, of DMAP (**iv**) as 1.4 × 10^–4^ mol ≙ 1.386 atm, and of the intermediates of the catalytic cycles as follows: catalyst (**1**): 6.8 × 10^–5^ ≙ 0.693 atm; catalyst (**1_c_**): 6.6 × 10^–5^ ≙ 0.673 atm.^53^

In the present study, we have considered only four energy terms: *H*_L_, *G*_L_ *G*SL and *G*SDL. *H*_L_ and *G*_L_ represents the gas-phase enthalpy and Gibbs free energy at the higher basis set mentioned before. These values were obtained by augmenting the *E*_L_ energy terms with the respective enthalpy and free energy corrections at the BP86/LANL2DZ(Ru)/6-31G*(H, C, N, O and P) level. The Gibbs free energy in the solution phase (*G*SL) was calculated as: *G*SL = *H*SL – TSSL. Here, *H*SL represents the solvent-phase enthalpy calculated from *E*SL and the enthalpy corrections at the lower basis set, whereas the solvation entropy (*S*SL) was estimated as two/third of the gas-phase value.^54^ *G*SDL additionally contains the dispersion-corrected energies to the *G*SL values. All single-point calculations were performed with tight wavefunction convergence criteria and an “ultrafine” (99 950) grid was used in numerical integration. The charge distribution was analyzed using Weinhold's NPA (Natural Population Analysis) approach.^55^

Additionally, we have performed AIMALL calculations^56^ to characterize the electron distribution around some selected bonds (Ru–ligand, C^α^–C^β^) applying Bader's AIM (atoms-in-molecule) theory.^57^ The bond critical point (BCP) is a point on this line where the gradient of the density is equal to zero. The magnitude of the electron density (*ρ*(*r*_b_)) and its Laplacian (∇^2^ *ρ*(*r*_b_)) at the BCP provide information about the strength and type of bond. The Laplacian indicates whether the density is locally concentrated (∇^2^ *ρ*(*r*_b_) < 0) or depleted (∇^2^ *ρ*(*r*_b_) > 0). Figures were generated using the Chemcraft visualization program.^58^

## C. Results and discussion

In the current computational study, we have selected 2-pyrrolidone (**I**) and 1-hexyne (**II**) as the reactants, in analogy to those used in the experimental setup.^37^ Three possible products are likely to be generated in the nucleophilic addition of **I** to **II**, namely the Markovnikov *gem*-enamide (**VI**) and *anti*-Markovnikov *Z*-(**VII**) and *E*-enamides (**VIII**) (Fig. 1a). Two different catalyst systems generated *in situ* from the pre-catalyst [Ru^II^(methallyl)_2_(cod)] in the presence of different sets of ligands and additives are [Ru^0^(Bu_3_P)_2_(DMAP)_2_] (**1**) and [Ru^0^(dcypm)_2_] (**1_c_**) respectively (Fig. 1b). We have performed DFT calculations to construct complete catalytic pathways for both these catalyst systems. In order to elucidate the origin of the regio- and stereoselectivity of the hydroamidation reaction we have considered the active catalyst and substrate used in the experiments without going for a truncated model system. In accordance with the proposed mechanism, our calculated catalytic cycles includes the following five steps: (1) oxidative addition of amide to ruthenium(0) to generate a ruthenium(ii) complex, (2) ligand dissociation followed by alkyne coordination to furnish a π-complex, (3) alkyne insertion to the Ru–H bond to afford a vinyl intermediate, (4) vinyl-vinylidene rearrangement, (5) intramolecular nucleophilic transfer, and finally (6) reductive elimination to generate the product.

The catalytic pathway involving catalyst **1** is discussed first in subsection C.I., followed by catalyst system **1_c_** in subsection C.II. Each fundamental step of the catalytic cycles is characterized by the changes in enthalpy (Δ*H*_L_), Gibbs free energy (Δ*G*_L_), and solvent free energy changes, with and without dispersion corrections (Δ*G*SDL, Δ*G*SL), at the higher basis set. Only the Δ*G*SDL energy term is discussed in the text unless otherwise mentioned. The remaining energy values are collected in the ESI.†

### C.I. Catalyst system 1

#### C.I.a. Oxidative addition and hexyne coordination

**Fig. 2:**
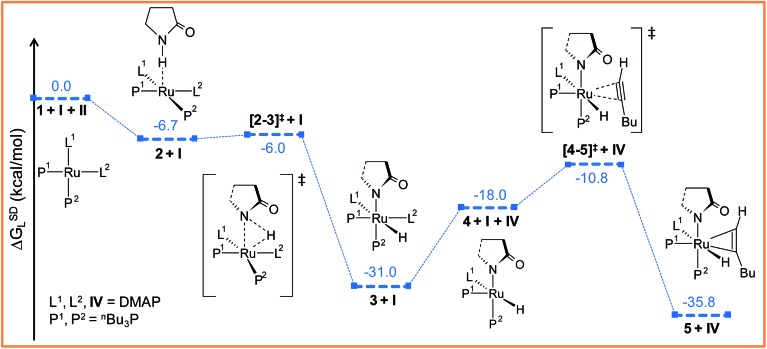
Energy profile for the oxidative addition (**1** → **3**) and hexyne coordination (**4** → **5**) steps. For energy nomenclature, refer to Computational details. Ball and stick structures of the computed species with labeling of atoms, geometrical parameters and energy profiles are collected in Fig. S3 and S4.†

The catalytic cycle for ruthenium-catalyzed hydroamidation usually starts by an oxidative addition step (Scheme 3). Fig. 2 details the energy profile of the steps studied in this sub-section, and Fig. S3 and S4† provide 3D structures of the optimized geometries and other details of the oxidative addition and hexyne coordination steps. The active catalyst **1** is a ruthenium(0) d^8^ system that possesses a typical square-planar geometry (Fig. S4†). The 4d_*z*_^2^ orbital of ruthenium is the main contributor to the HOMO of **1** (Fig. S32a†), allowing either reactant **I** or **II** to coordinate along its axis. The N^3^–H^1^ bond in **II** is more acidic than the C^α^(sp)–H^2^ bond in **I**, as is evident from the NPA charges of the H^1^ and H^2^ atoms respectively (*q*_H^1^_ = 0.402*e* in **II** and *q*_H^2^_ = 0.237*e* in **I**). In fact this might be the reason that drives the electron-rich ruthenium center (*q*_Ru_ = –0.57*e*) towards participating in the oxidative addition with **II**, a finding that is in agreement with the deuterium labeling experiments by Gooßen *et al.* ^37^ The oxidative addition step is initiated by the formation of an encounter adduct of the type of structure **2**, followed by subsequent N^3^–H^1^ bond activation (see Fig. 2) leading to the *cis*-configured octahedral complex **3**. Including dispersion corrected energy values, the encounter complex 2 is more stable than its separated reactants, a similar observation was reported in our previous calculations.^53^ The oxidative addition step is exergonic and its product more stable than the starting materials **1** and **II** taken together (Δ*G*SDL = –31.0 kcal mol^–1^, Fig. 2 and S3†). The calculated activation barrier for the transition state **[2–3]^‡^** is quite small (Δ^‡^ *G*SDL = 0.7 kcal mol^–1^). In the transition state **[2–3]^‡^**, ruthenium exhibits a five-coordinated, distorted trigonal bipyramidal-like geometry (Fig. S4†) with an N^3^–H^1^ bond elongated by 1.950 Å, and the imaginary mode (90i cm^–1^) involves stretching of N^3^–H^1^ bond. The NPA charge at Ru^0^ is decreased by 0.247*e* in going from **1** to **3**, suggesting that oxidative addition occurs in this process.

The ruthenium(ii) complex **3** represents a saturated 18-electron system with pseudo-octahedral geometry. In order to allow for hexyne coordination, a neutral ligand has to dissociate to create a vacant site at the metal center.^59^ Since the metal center in **3** is more electron-deficient than in **1** (*q*_Ru_ = –0.57*e* in **1** *vs. q*_Ru_ = –0.35*e* in **3**), it strongly binds to the electron-donor ligands, and therefore ligand dissociation should be unfavorable. Going from **1** to **3**, the NPA charge of P^1^, P^2^, N^1^ and N^2^ decreases by 0.088*e*, 0.034*e*, –0.019*e* and 0.016*e* respectively, indicating that phosphine ligands have contributed greater charge density to ruthenium than DMAP ligands (refer to Table S7†) and that hence, the decoordination of DMAP will be more facile than of a phosphine. Despite the fact that the bond distance of Ru–N^1^ (2.406 Å) is longer than that of Ru–N^2^ (2.197 Å) (refer to Fig. S4†), the Ru–N^2^ bond has to be cleaved so that hexyne can coordinate to Ru in the *cis* position to the Ru–H^1^ bond.

The particular reason for such site-specific coordination will become clear in the forthcoming sections. Following dissociation of one DMAP, the unsaturated intermediate **4** is generated (Fig. 2 and S3†). This step is endergonic (Δ*G*SDL = 13.0 kcal mol^–1^, refer to Fig. S3†) even though it involves ligand dissociation. The energetic penalty associated with ligand dissociation when including dispersion corrected energies is not surprising. Schoenebeck *et al.* reported that dissociation of PdL_2_ → PdL + L is endoergic upto 30 kcal mol^–1^ at M06L(THF)//B3LYP/{6-31+G(d), SDD (Pd)} level of theory.^60^ At this stage, the charge on ruthenium (*q*_Ru_ = –0.284*e*) has depleted, creating an electronic environment suitable for hexyne coordination to occur. Inspection of the KS-LUMO of **4** reveals that the incoming hexyne will be oriented such that it can deliver electron density to the empty 4d_*xz*_ orbital of ruthenium (Fig. S32b†). Gradual progress of hexyne to the metal center results in the typical η^2^-cordinated ruthenium(ii) intermediate **5** (Fig. 2, S3 and S4†). The hexyne coordination step involves transition state **[4–5]^‡^**, with a moderate energy barrier of 20.2 kcal mol^–1^ (Δ*G*SDL). **[4–5]^‡^** is characterized by a single imaginary frequency (39i cm^–1^), which resembles simultaneous elongations of the Ru–C^α^ and Ru–C^β^ bonds.

#### C.I.b. Hexyne insertion and vinyl-vinylidene rearrangement

Intermediate **5** is a perfect η^2^-complex as its two Ru–C (hexyne) bond lengths are similar (Ru–C^α^ = 2.189 Å, Ru–C^β^ = 2.196 Å, Fig. S4†), and hexyne acts as two-electron donor resulting in a ruthenium(ii) 18-electron system. The possibility of further oxidative addition of the hexyne *via* C(sp)–H bond to generate a Ru^IV^H_2_ species was not considered in the present study, since such a reaction requires high-energy intermediates as reported by Caulton^36^ and Wakatsuki^23*b*^ and is not supported by isotope labeling studies.^37^

**Fig. 3:**
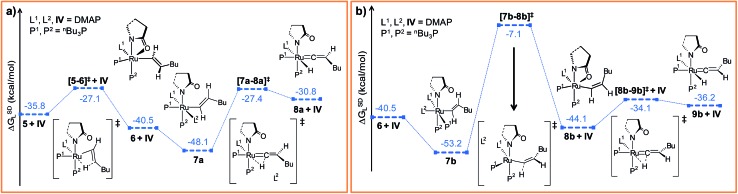
Energy profile for the hexyne insertion and vinyl-vinylidene rearrangement steps in (a) “path a” and (b) “path b”. Ball and stick structures of the computed species with labeling of atoms, geometrical parameters and energy profiles are collected in Fig. S5–S8.†

To accomplish the alkyne insertion step the Ru–H^1^ should remain coplanar with the coordinated alkyne unit. Indeed, the H^1^–Ru–C^α^–C^β^ dihedral angle of 12.1° in **5** fulfills the geometrical criteria for effective hydride transfer to C^β^ atom of hexyne fragment.^36^ However, after such insertion a coordinately unsaturated 16-electron vinyl complex would be generated. Therefore we made an attempt to study the hexyne insertion step by re-incorporating the DMAP, which was released during the preceding hexyne coordination step **3** → **5** (*vide supra*). As expected, no immediate coordination of the DMAP to the metal center took place even when placing it at the most promising position close to the metal center. So we studied the insertion step from intermediate **5** without considering the second DMAP at this stage. The vinyl complex **6** thus formed is somewhat more stable than the hexyne-coordinated complex **5** (Δ*G*SDL = –4.7 kcal mol^–1^; see Fig. 3a and S5†). The extra stabilization of **6** originates from a chelating N, O coordination of the amide through the carbonyl oxygen of the 2-pyrrolidinyl fragment.^61^ To gauge the stability of this interaction, we have optimized the isomer of **6** devoid of a Ru–O(sp^2^) bond (**6_I_**, refer to Fig. S16†). Calculated results show that **6_I_** is energetically less stable than **6** by 13.6/11.7 kcal mol^–1^ (Δ*H*_L_/Δ*G*_L_) confirming that the chelating N–O type coordination plays significant role in the stabilization of this complex.

The insertion step **[5–6]^‡^** requires a very small activation energy (Δ^‡^ *H*_L_ = 3.3 kcal mol^–1^, Δ^‡^ *G*SDL = 8.7 kcal mol^–1^, refer Fig. S5†). This is in agreement with calculations by Caulton and Eisenstein who found a value of (Δ^‡^ *H*_L_ = 6.6 kcal mol^–1^) for RuHCl(HC≡CH)(PH_3_)_2_ intermediate.^36^ The geometry of transition state **[5–6]^‡^** resembles **5**, albeit with elongated Ru–H^1^, Ru–C^β^ and C^α^–C^β^ bonds (refer to Fig. S6†). Furthermore, the insertion transition state is characterized by a unique eigenmode (622i cm^–1^) that resembles the hydride transfer from Ru to the C^β^ center.

Now the DMAP can coordinate to **6** from two opposite sites, either *syn* or *anti* to the existing DMAP ligand (see Fig. 3a and b). The pathways involving *anti* and *syn* coordination of DMAP are designated as “path a” and “path b”, respectively.

##### Path a

When bringing the second DMAP molecule closer to the metal center of **6**, the Ru–O(sp^2^) bond cleaves, and a six fold coordinated ruthenium(ii) vinyl intermediate **7a** forms, in which the two DMAP ligands are in trans position (Fig. 3a).^62^ In vinyl complex **7a**, Ru^II^ becomes electron-deficient (*q*_Ru_ = –0.035*e* in **7a**), whereas C^α^ and C^β^ are both electron-rich centers (*q*_C^α^_ = –0.361*e*, *q*_C^β^_ = –0.311*e* refer to Table S7†).

Electron density can be transferred to the ruthenium center by α-hydride migration, resulting in a vinylidene-type complex **8a**. The importance of vinylidene intermediates in organometallic chemistry cannot be neglected, and the existence of a similar vinyl-vinylidene rearrangement in catalysis has been substantiated from experimental reports.^35,37,63^ To relieve the steric strain associated with accommodating the second DMAP molecule, the Ru–C^α^ bond in **6** rotates to a pseudo-perpendicular orientation with respect to the Ru–N^1^–N^2^–P^1^ plane in **7a** (C^β^–C^α^–Ru–N^1^ = 160.4° in **6** *vs.* 72° in **7a**, Fig. S6†). Now to gain access to the vinylidene intermediate, α-hydride migration is necessary, and a coordination site at the metal center must be free. Incidentally, during the progress of the vinyl-vinylidene rearrangement step, a coordination site becomes available in the transition state **[7a–8a]^‡^** by decoordination of the newly added DMAP (see Fig. 3a). Interestingly, none of the phosphines undergo decoordination during the vinyl-vinylidene rearrangement, indicating that the Ru–phosphine bonds are stronger than the Ru–DMAP bonds. In **[7a–8a]^‡^**, the Ru–C^α^ bond distance (2.088 Å in **7a** *vs.* 1.881 Å in **[7a–8a]^‡^**) has already shortened and the Ru–C^α^–C^β^ bond angle flattened (132° in **7a** *vs.* 172° in **[7a–8a]^‡^**), resulting a geometry similar to the vinylidene intermediate **8a** (Fig. 3a and S6†). It is counter-intuitive that the intermediate **8a** in combination with a fully dissociated DMAP is higher in free enthalpy than the transition state **[7a–8a]^‡^** in step **7a** → **8a** (see Fig. S5†). The IRC revealed that this transition state actually connects to a loose adduct between **8a** and DMAP that is lower in enthalpy than the transition state. However, in solution, this adduct will not be stable, so that we have left out this loose DMAP adduct and directly depicted the separately calculated species **8a** and DMAP in Fig. 3a. Without their long-range interaction (Ru–DMAP = 6.688 Å), **8a** and DMAP are marginally higher in energy (Δ*H*_L_ = 0.8 kcal mol^–1^, refer Fig. S5†) than **[7a–8a]^‡^**, which causes the curious effect of an intermediate being seemingly higher in energy than the preceding transition state.

The transition state **[7a–8a]^‡^** is characterized by an imaginary mode (650i cm^–1^) describing the transfer of H^2^ to the ruthenium center. The activation energy of the vinyl-vinylidene rearrangement entails a moderate barrier (Δ^‡^ *H*_L_ = 7.1 kcal mol^–1^, Δ^‡^ *G*SDL = 20.7 kcal mol^–1^, refer Fig. S5†), which is in agreement with the result previously reported by Caulton (Δ^‡^ *H* = 3.6 kcal mol^–1^).^36^ The resulting intermediate, **8a** is less stable than the vinyl isomer **7a** (Δ*G*SDL = 17.3 kcal mol^–1^ for **7a** → **8a**). The vinyl-vinylidene rearrangement step is also accompanied with a significant change in the NPA charge on the Ru- and C^α^-centers (Δ*q*_Ru_ = –0.203*e* and Δ*q*_C^α^_ = 0.554*e* in **7a** → **8a**). It is, however, intriguing to note that the C^α^ in vinylidene **8a** turns out to be an electrophilic center with a NPA charge of 0.193*e*, making it susceptible towards nucleophilic transfer. Similar vinylidene complexes have been reported by Dixneuf *et al.* ^35^ to play a key role in various ruthenium-catalyzed nucleophilic addition reactions (*vide supra*).

##### Path b

**Scheme 4:**
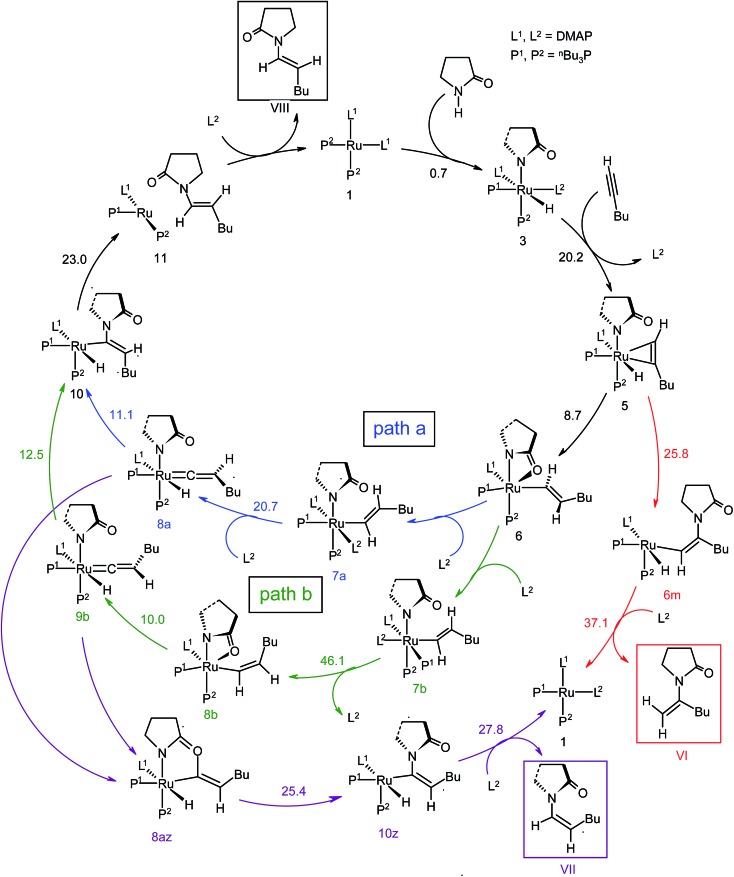
Computed catalytic cycle for catalyst system **1**. The energy values (Δ^‡^ *G*SDL in kcal mol^–1^) represent the highest barrier for a given step. Blue (“path a”) and green lines (“path b”) indicate the pathways leading to the *anti*-Markovnikov *E*-product, while red and magenta color-coded lines designate the pathways providing Markovnikov and *anti*-Markovnikov *Z*-products.

As briefly mentioned earlier, we have addressed another bifurcating reaction channel during the second DMAP addition step in intermediate **6** (see Scheme 4). Unlike “path a”, the additional DMAP is added from a direction *syn* to the coordinated DMAP, to generate intermediate **7b** (Fig. 3b). The two intermediates **7b** and **7a** are geometrical isomers that differ with respect to the two DMAP ligands bound to the ruthenium, the former being more stable by 5.1 kcal mol^–1^ (Δ*G*SDL, refer to Fig. 3a and b and S7†).^64^ Geometrical parameters and charge distributions of **7b** are similar to those of **7a** (refer to Fig. S6, Fig. S8 and Table S7†).

While allowing a transfer of H^2^ towards ruthenium with the aim of obtaining the vinylidene complex, our calculations revealed a new vinyl intermediate **8b**, in which the vinyl hydrogens are *syn* to each other, unlike in structure **6** (Fig. 3b and S8†). This step (**7b** → **8b**) describes a simple isomerization of the vinyl subunit with concomitant decoordination of the DMAP moiety. The activation barrier associated with the transition state **[7b–8b]^‡^** is very high (Δ^‡^ *G*SDL = 46.1 kcal mol^–1^), which is not surprising since the negative eigenvector depicts an out-of-plane twisting motion of H^2^ resulting in C^α^–C^β^ rotation (Fig. S8†). The coordination site at the ruthenium center liberated by removal of DMAP is filled by the oxygen atom of the 2-pyrrolidinyl ligand, which now coordinates in a chelating fashion as in the isomeric intermediate **6** (*vide supra*).

In order to progress further from **8b**, we allowed the α-hydride to migrate from C^α^ to the ruthenium center. The formation of the hydrido-vinylidene intermediate **9b** from **8b** represents our vinyl-vinylidene rearrangement step for “path b”. The resulting vinylidene **9b** is more stable than its isomer **8a** (**8a** → **9b**; Δ*G*SDL = –5.4 kcal mol^–1^). Geometrical parameters for **9b** differ from those for **8a** in the orientation of the butyl fragment coordinated to C^β^ (Fig. 3a and b). However, the electron density (*ρ*(*r*_b_)) and Laplacian of the electron density (∇^2^ *ρ*(*r*_b_)) at the BCP (bond critical point, see computational details) for the Ru–C^α^ (*ρ*(*r*_b_) = 0.178, 0.176; ∇^2^ *ρ*(*r*_b_) = 0.397, 0.349 for **8a** and **9b**) and C^α^–C^β^ (*ρ*(*r*_b_) = 0.339, 0.340; ∇^2^ *ρ*(*r*_b_) = –0.956, –0.959 for **8a** and **9b**) bonds in the vinylidene intermediates are similar. In “path b”, the conversion of vinyl to vinylidene (**8b** → **9b**) requires a lower energy barrier (Δ^‡^ *G*SDL = 10.0 kcal mol^–1^, in **8b** → **9b** *vs.* Δ^‡^ *G*SL = 20.7 kcal mol^–1^, in **7a** → **8a**, Fig. 3a and b) than that of the **7a** → **8a** step in “path a” (Fig. 3a and b), primarily because the vinyl complex **7a** is more stable. The two isomeric hydrido-vinylidene complexes **8a** and **9b** may interconvert by simple rotation of the vinylidene ligand about its Ru–C^α^–C^β^ axis. This type of transformation has been proposed by Oliván *et al.* for ruthenium and osmium vinylidene complexes.^36^ However, a similar rotation of the vinylidene ligand around Ru–C^α^–C^β^ in **8a** surprisingly generates a new stable geometry **8az**, which is reluctant to convert into the other vinylidene isomer **9b** (Fig. S14 and S15†). Intermediate **8az** is the precursor to the *Z*-stereoisomer, which is a minor product in the studied transformation to be discussed in the forthcoming paragraph.

The above calculations demonstrate the important role played by the second DMAP during the vinyl-vinylidene rearrangement steps (**6** → **8a**; **6** → **9b**, Fig. 3a and b). Its relatively low coordinating strength permits it to reversibly occupy a Ru coordination site before the vinylidene formation steps (**6** → **7a**; **6** → **7b**).

Alternative pathways for the rearrangement leading to vinylidene formation investigated without addition of a second DMAP failed to provide the correct intermediate **8a**. When vinyl-vinylidene rearrangement occurs directly from intermediate **6**, which is devoid of the second DMAP, decoordination of the 2-pyrrolidinyl unit takes place. We also investigated an alternative pathway for a vinyl-vinylidene rearrangement starting directly from **6**, but without success. Upon rotating the Ru–C^α^ bond thereby placing the hydrogen atom H^2^ *syn* to the Ru–O bond and then shortening the distance of H^2^ to the metal center, we arrived at a transition state. However, the imaginary mode seemed to resemble a movement of H^2^ towards the oxygen rather than the ruthenium center, which made it unlikely that this transition state would connect to the desired product. All these calculations led us to conclude that the coordination of an additional DMAP after the insertion step is vital for a successful vinyl-vinylidene rearrangement.

A possible line of argument against this proposed presence of a second DMAP molecule in the reaction “path a” may be that in “path b”, the vinyl-vinylidene rearrangement (**8b** → **9b**) is successful even in absence of a second DMAP molecule. However, it needs to be considered that the geometrical position of H^2^ with respect to N^3^ determines this rearrangement step. In case of both **7a** and **8b**, the H^2^ remains opposite to the N^3^ with N^3^–Ru–C^α^–H^2^ dihedral of 155.2° and 148.6° respectively (Fig. S6 and S8†). The correct orientation of the vinyl fragment is accomplished only in presence of a second DMAP as illustrated in the step **7b** → **8b**.

#### C.I.c. Nucleophilic transfer

In this section, we discuss the intramolecular nucleophilic transfer of the coordinated amide in the vinylidene complexes studied so far. “Path a” and “path b” consider the continuation of the reaction progress from intermediates **8a** and **9b**, respectively.

##### Path a

**Fig. 4:**
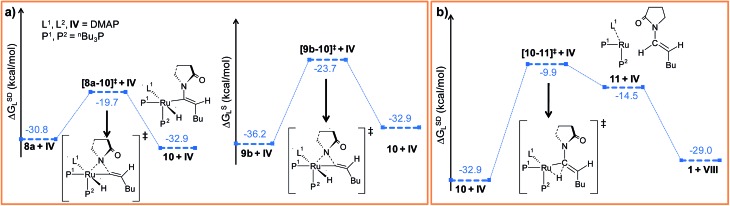
Energy profile for the (a) nucleophilic transfer steps in “path a” and “path b” and (b) the reductive elimination step. Ball and stick structures of the computed species with labeling of atoms, geometrical parameters and energy profiles are collected in Fig. S9–S12.†

In vinylidene **8a**, C^α^ is an electron-deficient center (*q*_C^α^_ = 0.193*e*) and quantitatively contributes to the LUMO of the molecule. The HOMO is localized on both N^3^ and the carbonyl O atoms of the coordinated 2-pyrrolidinyl unit (see Fig. S32d†). **8a** undergoes intramolecular nucleophilic transfer through N^3^ to generate intermediate **10** *via* the transition state **[8a–10]^‡^**, which has a moderately low activation barrier (Δ^‡^ *G*SDL = 11.1 kcal mol^–1^, Fig. 4a and S9†). One interesting fact is that in transition state **[8a–10]^‡^**, the vinylidene plane (C^β^–C^α^–H^1^–^*n*^Bu) rotates about 90° along the Ru–C^α^–C^β^ axis (N^3^–Ru–C^β^–H^1^ = –100° in **8a** *vs.* 10.9° in **[8a–10]^‡^**, Fig. S10†), and the *n*-butyl group is oriented *anti* to the incoming 2-pyrrolidinyl unit primarily due to steric hindrance. Therefore, the resulting geometry of **10** already indicates the *E*-selective formation of the enamide product after the reductive elimination step. In contrast, if the butyl group is oriented *syn* to the 2-pyrrolidinyl unit, then the *Z*-enamide would be the final product. This is considered in the later sections (*vide infra*).

##### Path b

Similar to “path a”, we explored the nucleophilic transfer from **9b**. During the progress of the nucleophilic transfer, rotation of the C^β^–C^α^–H^1^–^*n*^Bu plane occurs along the Ru–C^α^–C^β^ axis, a geometrical change analogous to “path a”. The geometry of the transition state **[9b–10]^‡^** is very similar to that of **[8a–10]^‡^**, while the activation barrier (Δ^‡^ *G*SDL = 12.5 kcal mol^–1^, Fig. S9†) is slightly higher in “path b” than in “path a” (Fig. 4a). A probable reason is the presence of slightly less charge density on C^α^ in **9b** (*q*_C^α^_ = 0.151*e* in **9b** *vs.* 0.193*e* in **8a**). However, both the vinylidene complexes **8a** and **9b** afford the same intermediate **10**, in which the butyl group is *anti* to the 2-pyrrolidinyl unit. Therefore, rotation of the vinylidene plane does not depend on the orientation of the butyl fragment in the vinylidene intermediates (Fig. 3a and b). The butyl group is placed *anti* to the 2-pyrrolidinyl unit because the alternative *syn*-arrangement is sterically overcrowded.

#### C.I.d. Reductive elimination

The final step of the catalytic cycle is the reductive elimination, in which the desired product is liberated and the catalyst regenerated. From **10**, the reductive elimination step requires the transfer of H^2^ to C^α^ to liberate the *E*-product **VIII** (Fig. 4b). Though intermediate **10** is coordinately unsaturated, the negative NPA charge on the metal center is quite high (*q*_Ru_ = –0.374*e*), making the coordination of further ligands unfavorable. Our calculations revealed that coordinating a second DMAP to the vacant site of **10** leads to an increased energetic barrier in the subsequent reductive elimination step (Δ^‡^ *E*_e_ = 31.2 kcal mol^–1^, Δ^‡^ *G*_298_ = 38.4 kcal mol^–1^ at BP86/LANL2DZ(Ru)/6-31G*(H, C, N, O & P) level of theory). Reductive elimination of the product from **10** by gradual transfer of H^2^ to C^α^ proceeds *via* the transition state **[10–11]^‡^**.^65^ The activation barrier of this step is only moderate (Δ^‡^ *G*SDL = 23.0 kcal mol^–1^, Fig. S11†). It leads to intermediate **11**, in which the *E*-enamide has been formed but remains weakly bound to the metal center (Fig. S12†).

Such weakly coordinated complexes are frequently observed in gas-phase optimizations, but can seldom be found under experimental conditions in coordinating solvents.^66^ In the final step **11** → **1**, the enamide product is easily liberated from adduct **11** by coordinating a further DMAP molecule, which regenerates the Ru^0^ catalyst (Fig. 4b and S11†).

#### C.I.e. Regio- and stereoselective products

Up to this point, we have described two different pathways (“path a” & “path b”) for the formation of the *E*-enamide. Both originate from intermediate **5** and merge at intermediate **10** later in the catalytic cycle (Scheme 4). However, we have also endeavored to explore pathways for generating hydroamidation products with different regio- and stereoselectivities. In this section, we discuss the mechanism of formation of both the Markovnikov and the *Z*-configured enamides.

The Markovnikov products form if the amide nucleophile attacks at the hexyne C^β^. We found that Markovnikov addition can proceed prior to alkyne insertion and, in our case, can initiate from intermediate **5**. The C^β^ center in intermediate **5** bears a greater positive charge than the free alkyne **I** (*q*_C^β^_ = 0.063*e* in **5** *vs. q*_C^β^_ = 0.001*e* in **I**), inciting the electron-rich N^3^ center (*q*_N^3^_ = –0.532*e* in **5**) to undergo an intramolecular nucleophilic transfer (see Fig. S13†) leading to the Markovnikov product **VI** (Fig. 1). However, under experimental conditions, no Markovnikov product was observed.^33,34^ Gratifyingly, our calculated results reveal that the formation of the Markovnikov product involves very high transition barriers for both the nucleophilic transfer **5** → **6m** (Δ^‡^ *G*SDL = 25.8 kcal mol^–1^) and subsequent reductive elimination steps from **6m** *via* the transition state **[7m–1]^‡^** (Δ^‡^ *G*SDL = 37.1 kcal mol^–1^; Fig. S13†). Such high energy barriers can be explained on the grounds of a low NPA charge on the C^β^ carbon in **5**. A similar justification was provided above, when comparing the activation barriers for the nucleophilic transfer in “path a” and “path b” (*vide supra*). Furthermore, molecular orbital analysis reveals that the KS-HOMO of **5** is the bonding π-orbital of the C^α^–C^β^ bond, which repels the nucleophilic 2-pyrrolidinyl unit (Fig. S32c†).

The vinylidene complexes (**8a** and **9b**) discussed so far involve a finite contribution of electron density from the carbonyl oxygen as seen from their KS-HOMO (Fig. S32d–e†). Hence, a fair possibility exists for oxygen to attack the electrophilic C^α^ center in both the vinylidene complexes from “path a” and “path b”. Indeed, such a nucleophilic transfer of the sp^2^ oxygen from the coordinated 2-pyrrolidinyl unit results in a stable isomer **8az**, which is more stable by –33.6 kcal mol^–1^ (Δ*G*SDL) than the starting materials (Fig. S14†). An interesting observation during these transformations (**8a** → **8az**/**9b** → **8az**) is that the butyl substituent at C^β^ arranges *syn* to the 2-pyrrolidinyl fragment, predetermining the stereoselectivity towards the *Z*-enamide product (Fig. S15†). Progressing from **8az**, we have explored the potential energy surface with the aim of creating an N^3^–C^α^ bond, resulting in intermediate **10z**. Complex **10z** can undergo reductive elimination in a similar fashion to furnish the *Z*-enamide product. The activation barrier for the step **8az** → **10z** *via* the transition state **[8az–10z]^‡^** (Δ^‡^ *G*SDL = 25.4 kcal mol^–1^, refer to Fig. S14†) requires substantially higher energy values, precluding the formation of the *Z*-enamide product in high yields, as substantiated by the experimental findings.^34^ The calculated route for *Z*-enamide formation overrules any possibility of H^2^ migrating to the oxygen atom of 2-pyrrolidinyl unit. Apart from the fact that they are well separated (H^2^–O = 2.641 Å in **8a**), the same H^2^ is necessary to accomplish a successful reductive elimination step (Fig. S11†). If coordinated, not only the transfer of H^2^ to C^α^ will be difficult, the reduced nucleophilicity at oxygen center will also affect adversely the *Z*-enamide route.

**Fig. 5:**
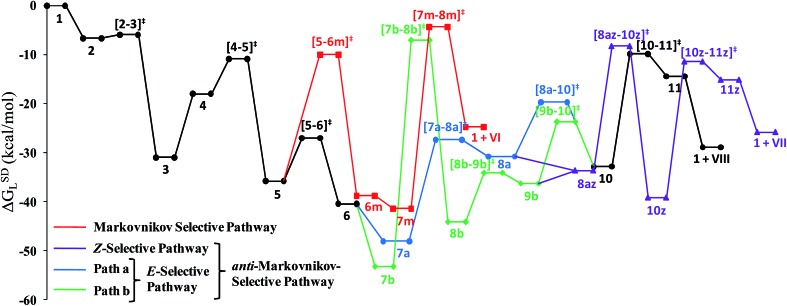
Overall reaction energy profile for the enamide formation.

The overall catalytic cycle and reaction energetics for the different pathways originating from the catalytic system **1** are represented in Scheme 4 and Fig. 5, respectively. From intermediate **5**, three different reaction channels can emanate. “Path a” and “path b” involve the formation of isomeric vinylidenes (**8a** and **9b**), which can undergo nucleophilic addition to furnish the common intermediate **10**. From **10**, the *E*-product is selectively formed by reductive elimination (Scheme 4).

However another possibility remains in which the energetically unfavorable (Fig. 5) Markovnikov addition product is liberated following the step **5** → **6m** → **VI** as discussed in the previous section. A pathway to the *Z*-product can also be connected from the vinylidene intermediates **8a** and **9b** *via* nucleophilic addition of the sp^2^ oxygen.

Closer inspection of the reaction profile (Fig. 5) reveals that the selective formation of *E*-products is more facile than its other stereo- and regio-chemical variants. This finding is supported by the experimental observations for the studied catalytic system **1**. For all investigated pathways, the oxidative addition and hexyne coordination steps (**1** → **5**, black lines) follow a common reaction route (Scheme 4, Fig. 5). For the pathway leading to *anti*-Markovnikov *E*-enamides, “path a” is more facile than “path b”, the latter involving a substantially higher barrier of 46.2 kcal mol^–1^ (Δ^‡^ *G*SDL) for the step **6** → **8b** (green lines, Fig. 5) compared to 20.7 kcal mol^–1^ (Δ^‡^ *G*SDL) for **6** → **8a** (blue lines). Undoubtedly, this signifies that “path a” is the most accessed route.

For the Markovnikov addition, the highest barrier of 37.1 kcal mol^–1^ (Δ^‡^ *G*SDL) is encountered along step **6m** → **1** (red line in Fig. 5). Obviously, this regio-isomer will not be detected under the given reaction condition. Hence, from **5**, the reaction will proceed along “path a” and refrain to follow the alternative **5** → **6m** route. Considering the *Z*-selective pathway from **8a**, the highest energy-demanding transformation (Δ^‡^ *G*SDL = 27.8 kcal mol^–1^) occurs for step **10z** → **11z** (magenta lines, Fig. 5). Even though the energy for the reductive elimination step is higher in the *Z*-selective pathway than for the analogous step (**10** → **11**; Δ^‡^ *G*SDL = 23.0 kcal mol^–1^) in the *E*-selective pathway (black lines), we believe that this barrier can be surmounted under the reaction conditions, giving rise to the *Z*-enamide as a minor product, which is supported by experimental results.

The energetic span model proposed by Amatore and Jutand,^67^ later refined by Shaik and Kozuch,^68^ says that the largest rate and hence the highest turnover is obtained from the lowest Gibbs energy span in a catalytic cycle. The energy span is the difference between the highest transition state and lowest intermediate of the entire catalytic cycle. In this study, the computed cycle deals with the formation of different stereo- and regiochemical enamide isomers branching out from a small number of key intermediates of the hydroamidation pathway (Scheme 4 and Fig. 5). For both the stereoisomeric pathways, the energy span model designates intermediate **6** as the lowest, and thus, turnover frequency-determining intermediate (TDI). The turnover frequency-determining transition state (TDTS) is different for *E*- and *Z*- selective pathways. In the former case, the TDTS is **[10–11]^‡^**, and in the latter it is **[8az–10z]^‡^**, with a relative TOF of 10.0 in favor of the *E*-enamide isomer. In order to assess the maximum influence of individual steps in contributing to the relative TOF of product formation, we have considered those steps following vinylidene intermediate **8a** from which the *E*- and *Z*-enamide pathways originate. The highest activation barrier in case of *E*-product formation is associated with the reductive elimination step **10** → **11** (Δ^‡^ *G*SDL = 23.0 kcal mol^–1^). Similarly, for the other stereoisomer, the highest surmountable energy barrier is also associated with the reductive elimination step **10z** → **11z** (Δ^‡^ *G*SDL = 27.8 kcal mol^–1^). Therefore, on the level of individual reaction steps, it is also clear that the *E*-enamide formation route is more favorable by 4.8 kcal mol^–1^ than that leading to the *Z*-isomer, concurring with the experimental observation towards the preference for the *E*-product.

### C.II. Catalyst system **1_c_**

In order to understand the influence of ligands in determining the selectivity of product formation, we have performed a similar mechanistic investigation for [Ru^0^(dcypm)_2_] (**1_c_**) as the catalyst system instead of [Ru^0^(Bu_3_P)_2_(DMAP)_2_] (**1**) (*vide supra*, Fig. 1b). The fundamental reaction steps originating from **1_c_** are similar to those corresponding to catalyst system **1**. In the following section, we briefly discuss the mechanism of the catalytic cycle with the three following subsections: (a) oxidative addition, hexyne coordination and insertion, (b) vinyl-vinylidene rearrangement and nucleophilic transfer, and (c) reductive elimination.

#### C.II.a. Oxidative addition, hexyne coordination and insertion

**Fig. 6:**
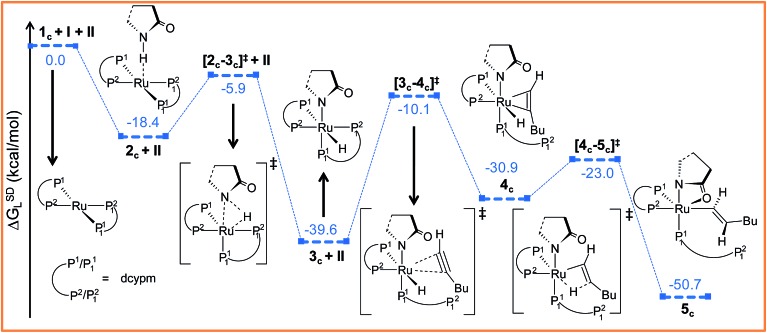
Energy profile for the oxidative addition and hexyne insertion steps for catalytic system **1_c_**. Ball and stick structures of the computed species with labeling of atoms, geometrical parameters and energy profiles are collected in Fig. S18 and S19.†

In analogy to the initiating step starting from **1**, axial addition of **II** to catalyst **1_c_** leads to the stable adduct **2_c_**, followed by N–H^1^ bond activation *via* the transition state **[2_c_–3_c_]^‡^** to generate the oxidatively added Ru^II^ intermediate **3_c_** (Fig. 6, S18 and 19†). The NPA charge on ruthenium in **2_c_** is more negative than that in **2** (*q*_Ru_ = –1.056*e* in **2_c_** *vs.* –0.656*e* in **2**), indicating a more facile oxidative addition step. However, the calculated activation barrier is higher (Δ^‡^ *G*SDL = 12.5 kcal mol^–1^, Fig. 6 and S18†) than in the step corresponding to catalytic system **1** (Δ^‡^ *G*SDL = 0.7 kcal mol^–1^, for step **2** → **3**, Fig. 2), suggesting the transformation to be governed to a greater extent by the steric environment around the metal center.

Further progress of **I** towards the ruthenium center resulted in η^2^-hexyne complex **4_c_** *via* the transition state **[3_c_–4_c_]^‡^**. In the transition state **[3_c_–4_c_]^‡^**, the P_1_^2^ atom has already moved away from ruthenium to create a vacant side for the incoming alkyne to coordinate (Fig. S18†). Unlike in catalytic system **1** (Fig. 2), the alkyne coordination step (**3_c_** → **4_c_**) is endoergic (Δ*G*SDL = 8.7 kcal mol^–1^) and has a higher activation barrier (Δ^‡^ *G*SDL = 29.5 kcal mol^–1^). An important point to consider here is that the charge on ruthenium increases by 0.219*e* during hexyne coordination (**3_c_** → **4_c_**). However, a similar increase was not observed during the transformation **3** → **5**, suggesting a superior electron-donating capability of the phosphorus atoms of the ligand dcypm (**V**) compared to the nitrogen atoms of DMAP (Tables S7 and S8†).

The next step should be the usual insertion of the η^2^-coordinated hexyne to the Ru–H^1^ bond. Migration of the H^1^ atom from ruthenium to C^β^ in **4_c_** generates the vinyl intermediate **5_c_** (refer to Fig. 6, S18 and S19†). The activation barrier (Δ^‡^ *G*SDL = 7.9 kcal mol^–1^, Fig. 6) for the transition state **[4_c_–5_c_]^‡^** is lower than in the insertion step **5** → **6** (Δ^‡^ *G*SDL = 8.8 kcal mol^–1^, Fig. 3a) calculated for catalytic system **1**. Since the electronic charge on the metal is further reduced (Δ*q*_Ru_ = 0.185*e*, for the step **4_c_** → **5_c_**, Table S8†), this insertion step can be characterized as hydride migration similar to the previously studied steps **5** → **6** (Table S7†).

#### C.II.b. Vinyl-vinylidene rearrangement and nucleophilic transfer

In the previous catalytic system **1**, we have observed that two different vinyl isomers **6** and **8b** can be generated depending on the orientation of the additional, incoming DMAP ligand to intermediate **6**. A similar possibility can be ruled out for catalytic system **1_c_**. In order to obtain two isomeric vinyl structures, we have performed direct C^α^–C^β^ bond rotation from intermediate **5_c_**. Unsurprisingly, the activation barrier for this step is high (Δ^‡^ *G*SDL = 68.0 kcal mol^–1^, Fig. S20 and S22†) due to rotation around the carbon–carbon double bond, and hence, this pathway will not be followed. An alternative, more facile pathway consisting of H^2^ migration followed by C^α^–C^β^ rotation and finally retro-transfer of H^2^ to C^α^ was calculated. The resulting vinyl isomer **9b_c_** is less stable than **5_c_** by 8.7 kcal mol^–1^ (Δ*G*SDL). These isomeric intermediates (**5_c_** and **9b_c_**) lead to two different reaction channels which referred as “path a_c_” and “path b_c_” from now onwards. Both these intermediates possess a Ru–O(sp^2^) interaction similar to that observed in the vinyl complexes **6** and **8b** (Fig. 3a and b, S6, S8, S19 and S26†).

##### Path a_c_

**Fig. 7:**
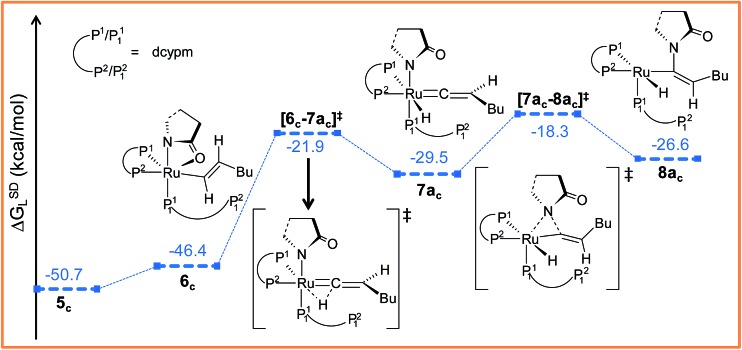
Energy profile for the vinyl-vinylidene rearrangement and nucleophilic transfer steps in “path a_c_”. Ball and stick structures of the computed species with labeling of atoms, geometrical parameters and energy profiles are collected in Fig. S23 and S24.†

Intermediate **5_c_** is not suitable for vinyl-vinylidene rearrangement because H^2^ migration to the ruthenium center is accompanied with dissociation of 2-pyrrolidinyl unit. Previous experience with catalytic system **1** led us to expect that a successful transfer of H^2^ occurs only when the C^α^–H^2^ bond faces *syn* to the Ru–O(sp^2^) bond (refer intermediate **6_c_** in Fig. S24†). In search of such an intermediate, we have performed a rotation of N–Ru–C^α^–H^2^ dihedral angle in **5_c_** by about 180°, leading to a less stable isomer **6_c_** (Δ*G*SDL = 4.3 kcal mol^–1^, Fig. S23 and S24†). Now from **6_c_**, further migration of H^2^ from C^α^ to the ruthenium center furnished the desired vinylidene intermediate **7a_c_** (Fig. 7 and S23†). Also from previous experience, we were aware that the vinyl-vinylidene rearrangement step requires a hydride ion transfer in which the metal center in the resulting vinylidene complexes acquires more electron density (Table S7†). It is important to note that the ruthenium center in **6_c_** is more electron-rich (*q*_Ru_ = –0.233*e*) than the other calculated vinyl intermediates **7a** (*q*_Ru_ = –0.035*e*) and **9b** (*q*_Ru_ = –0.027*e*, Table S8†), suggesting that the hydride transfer will be less favorable, with a higher activation barrier (Δ^‡^ *G*SDL = 24.5 kcal mol^–1^, for **6_c_** → **7a_c_**, 20.7 kcal mol^–1^ for **7a** → **8a** and 10.1 kcal mol^–1^ for **8b** → **9b**) for the step **6_c_** → **7a_c_**.

The geometry of **7a_c_** is similar to that of **8a**, in which the two hydrogen atoms (H^1^ and H^2^) are oriented *trans* to each other (Fig. 7, S23 and S24†). Like **8a** and **9b**, the C^α^ atom is electron-deficient in **7a_c_** (Table S7 and S8†) creating a suitable electronic environment for intramolecular nucleophilic transfer to occur. The activation barrier of this step (**7a_c_** → **8a_c_**) is moderately low (Δ^‡^ *G*SDL = 11.2 kcal mol^–1^), similarly to that of **8a** → **10** (Δ^‡^ *G*SDL = 11.1 kcal mol^–1^) and **9b** → **10** (Δ^‡^ *G*SDL = 12.5 kcal mol^–1^). In contrast to **[8a–10]^‡^** and **[9b–10]^‡^** (see Fig. 4a, 7 and S24†), the vinylidene plane (C^β^–C^α^–H^1^–Bu) in the transition state **[7a_c_–8a_c_]^‡^** rotates into an orientation in which the 2-pyrrolidinyl unit is *syn* to the butyl fragment, predicting the final product to be the *Z*-enamide. The N–C^α^–C^β^–H^2^ dihedral angle is 174.7° already in **8a_c_**, which will now undergo reductive elimination to liberate product **VII**.

##### Path b_c_

**Fig. 8:**
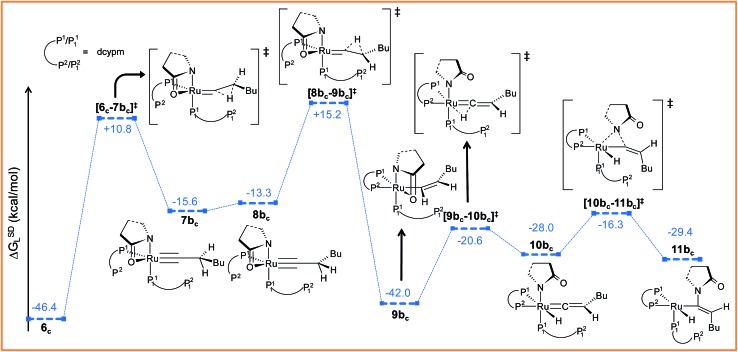
Energy profile for the vinyl isomerization, vinyl-vinylidene rearrangement and nucleophilic transfer steps in “path b_c_”. Ball and stick structures of the computed species with labeling of atoms, geometrical parameters and energy profiles are collected in Fig. S25 and S26.†

**Fig. 9:**
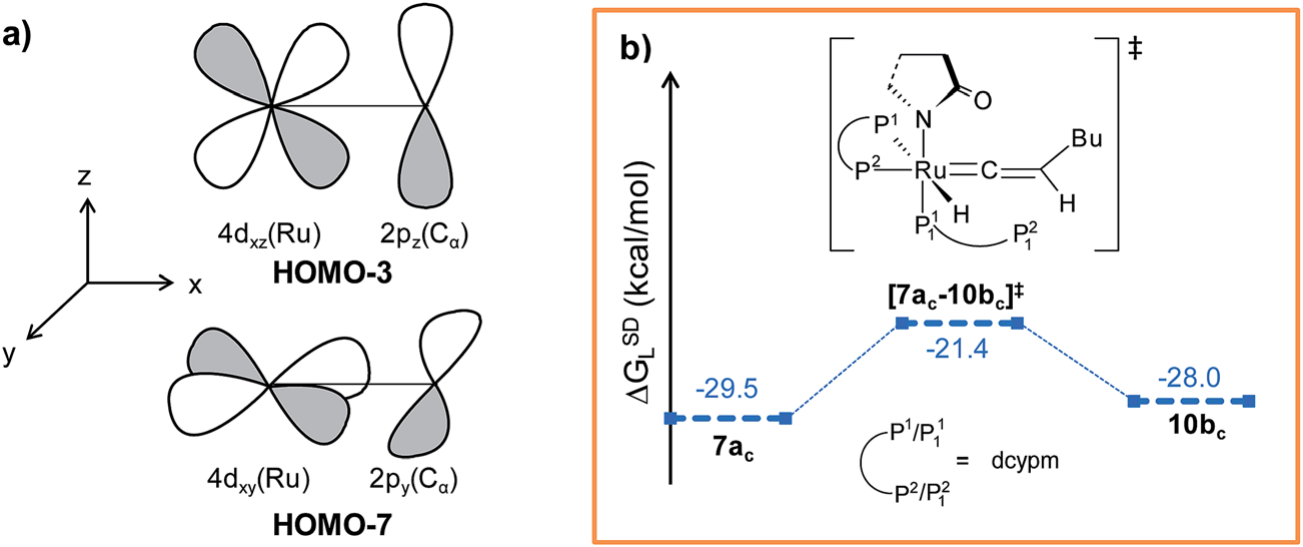
(a) π-Bonding orbitals of Ru–C^α^ bond in **7b_c_** intermediate and (b) energy profile for the vinylidene isomerization step. Ball and stick structures of the computed species with labeling of atoms, geometrical parameters and energy profiles are collected in Fig. S21 and S22.†

During step **6_c_** → **7b_c_**, H^2^ undergoes migration to C^β^ *via* a high-energy transition state **[6_c_–7b_c_]^‡^** (Δ^‡^ *G*SDL = 57.2 kcal mol^–1^, Fig. 8 and S25†), in which the imaginary mode depicts the hydride transfer between the carbon centers. A similarly high activation barrier was reported by Oliván and Clot during their study of the 1,3 H-migration in MHCl(CCH_2_)(PH_3_)_2_ (M = Ru, Os) complexes.^36^ Intermediate **7b_c_** formed in the hydride transfer shows a short Ru–C^α^ bond (1.743 Å, Fig. S26†), which is even more contracted than the calculated Ru–C^α^ vinylidene bond (1.833 Å) in **7a_c_**. NBO analysis reveals that one σ- and two π-bonds are possible between the ruthenium and C^α^ atoms (Fig. 9a). The orbitals participating in the overlap between the metal d-orbitals and the orthogonal p-orbitals of C^α^ are depicted in Fig. 9a. Additionally, the C^α^–C^β^ bond in **7b_c_** is elongated by 0.11 Å compared to **6_c_**, allowing an extra flexibility of this bond.

Now, an easy C^α^–C^β^ bond rotation can give rise to intermediate **8b_c_**, in which the orientation of H^1^ is different to its immediate predecessor **7b_c_** (Fig. S25 and S26†). Subsequent migration of H^2^ to C^α^ furnished the vinyl intermediate **9b_c_**, which differs from **6_c_** in that the vinyl hydrogens are oriented in a *syn* fashion. The calculated activation barrier for the retro-hydrogen transfer through **[8b_c_–9b_c_]^‡^** is lower than the previous barrier by 28.7 kcal mol^–1^ (Δ*G*SDL, Fig. 8 and S25†).

The optimized vinyl isomer **9b_c_** is less stable than **6_c_** by 4.4 kcal mol^–1^ (Δ*G*SDL). Subsequent hydride transfer from C^α^ to the ruthenium generates the isomeric vinylidene **10b_c_** *via* transition state **[9b_c_–10b_c_]^‡^**, which is isoenergetic to **[6_c_–7a_c_]^‡^** (refer to Fig. 7 and 8). Similarly to **9b**, the two hydrogens (H^1^ and H^2^) of the vinylidene ligand in intermediate **10b_c_** are *syn* to each other (Fig. S26†). From **10b_c_**, we allowed the intramolecular nucleophilic transfer of the 2-pyrrolidinyl unit to occur from ruthenium to the C^α^. In contrast to “path a_c_”, the butyl fragment in transition state **[10b_c_–11b_c_]^‡^** is oriented *anti* to the 2-pyrrolidinyl unit (Fig. 8). A similar orientation was visible during the analogous transition states in steps **8a** → **10** and **9b** → **10** for catalytic system **1** (see Fig. 4a). Intermediate **11b_c_** (see Fig. 8) is more stable by 2.8 kcal mol^–1^ (Δ*G*SDL) than **8a_c_** and will undergo reductive elimination to liberate the *E*-enamide product **VIII**.

At this point, we opined that during the nucleophilic transfer step, the rotation of the C^β^–C^α^–H^1^–Bu plane is controlled by the orientation of the butyl fragment in the presence of the cyclohexyl groups on the P_1_^2^ atom, as discussed in the forthcoming section. In contrast, in the previous catalytic cycle (Scheme 4), the vinylidene isomers **8a** and **9b** gave the same intermediate **10** due to the presence of less bulky monodentate phosphine ligands (*vide supra*).

It is important to mention that the isomerization step **6_c_** → **10b_c_** calculated so far to connect vinylidene intermediate **10b_c_** is still energetically highly demanding. The overall activation energy for step **6_c_** → **10b_c_** is 61.6 kcal mol^–1^ (Δ^‡^ *G*SDL), which raises doubts as to its ability to surmount the barrier even at the elevated reaction temperature of 373 K. Hence, we explored an alternate route in which the isomerization can be achieved in a single step *via* rotation of the C^β^H^1^(Bu) unit around the Ru–C^α^ axis (Fig. 9b). A similar type of rotation was suggested by Oliván and Clot to be facile for Ru(ii) hydrido-vinylidene complexes.^36^ Gratifyingly, a similar rotation from **7a_c_** *via* transition state **[7a_c_–10b_c_]^‡^** (see Fig. 9b and S21†) entails a low activation barrier of 8.1 kcal mol^–1^ (Δ^‡^ *G*SDL). In transition state the ruthenium is more negative (*q*_Ru_ = –1.121*e vs.* –0.340*e* in **7a_c_** and **10b_c_**) than vinylidines **7a_c_** and **10b_c_**, suggesting a lack of back bonding with C^α^ whereas C^α^–C^β^ π-bond remains intact during rotation. This finding unambiguously point towards the existence of a single-step, easily accessible route for the interconversion of the vinylidenes in presence of dcypm ligands.^69^

#### C.II.c. Reductive elimination

##### Path a_c_

**Fig. 10:**
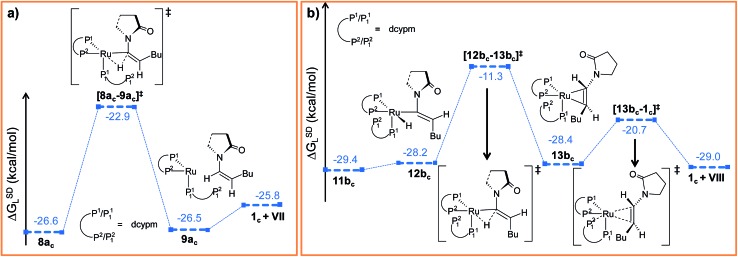
Energy profile for the reductive elimination step in (a) “path a_c_” and (b) “path b_c_”. Ball and stick structures of the computed species with labeling of atoms, geometrical parameters and energy profiles are collected in Fig. S27–S30.†

In reductive elimination step, the C^α^–H^2^ bond forms and the ruthenium(0) catalyst is regenerated (Fig. 10a). Therefore, from **8a_c_**, H^2^ was transferred from ruthenium to C^α^ to generate intermediate **9a_c_** (Fig. S27 and S28†). The activation barrier for the step **8a_c_** → **9a_c_** is quite small (Δ^‡^ *G*SDL = 3.7 kcal mol^–1^, Fig. 10a and S27†) compared to the analogous reductive elimination step (Δ^‡^ *G*SDL = 23.0 kcal mol^–1^, for **10** → **11**) for catalytic system **1**. In **[8a_c_–9a_c_]^‡^**, the electron density on ruthenium has increased by 0.136*e* (Table S8†) from **8a_c_**, characterizing this step as proton rather than hydride transfer. Intermediate **9a_c_** already reveals the product formation, with the enamide weakly bound to the coordinatively unsaturated ruthenium center (Fig. 10a and S28†). Subsequent coordination of already dissociated P_1_^2^ atom of dcypm to the ruthenium center will allow liberating the *Z*-enamide (**VII**) product and regenerating the ruthenium(0) catalyst **1_c_**.

##### Path b_c_

Similarly to “path a_c_”, we proceeded from **11b_c_** by transferring the H^2^ atom from ruthenium to C^α^ in order to accomplish the reductive elimination step. Unfortunately, we were unable to find converging intermediates along this reaction pathway after constraining geometrical features to fit the expected structures along the way. Therefore, we sought for an alternative mechanism for the reductive elimination. First, we modified the orientation of the decoordinated phosphine (P_1_^2^ part) by rotation along the Ru–P_1_^1^ bond to generate intermediate **12b_c_** (see Fig. 10b, S29 and S30†), which is less stable than **11b_c_** by 1.2 kcal mol^–1^ (Δ*G*SDL). Next, the H^2^ atom was transferred from ruthenium to furnish intermediate **13b_c_**. The enamide that is already formed is bound to the ruthenium center in a η^2^-fashion, while P_1_^2^ still remains uncoordinated. The activation barrier for reductive elimination is higher by 13.2 kcal mol^–1^ than that for **8a_c_** → **9a_c_** in “path a_c_” (Δ^‡^ *G*SDL = 16.9 kcal mol^–1^ *vs.* 3.7 kcal mol^–1^). The ruthenium center in **13b_c_** is more electron-deficient than **1_c_** (*q*_Ru_ = –0.439*e* in **13b_c_** *vs.* –0.903*e* in **1_c_**) due to the presence of a π-accepting, η^2^-coordinated enamide. Gradual elongation of the Ru–C^α^ and Ru–C^β^ distances in **13b_c_** leads to *E*-enamide with regeneration of the initial catalyst **1_c_**. This step is very facile with a small activation barrier (Δ^‡^ *G*SDL = 7.7 kcal mol^–1^, Fig. 10b and S29†). Interestingly, during the removal of enamide from **13b_c_**, there was an abrupt shortening of the Ru–P_1_^2^ bond resulting the formation of a covalent Ru–P bond (Ru–P_1_^2^ = 4.662 Å and 4.003 Å in **13b_c_** and **[13b_c_–1_c_]^‡^**).

Whenever the reaction steps constitute electron-rich metal complexes, the oxidative addition will generally be more facile than reductive elimination. In a similar line of argument, Negishi *et al.* proposed that in palladium-catalyzed C–C bond-forming reactions, the reductive elimination rate is inversely proportional to the ligand basicity.^70^ Fig. S33† depicts the NPA charges on ruthenium centers for all calculated saddle points (see Table S8†) present in “path a”, “path a_c_” and “path b_c_”, respectively. The plot shows that the ruthenium complexes with cyclic phosphines are more electron-rich than their monophosphine counterparts. This observation can directly point to more facile oxidative addition and more difficult reductive elimination steps for the catalytic system **1_c_** compared to the catalytic system **1**. However, to our surprise, the calculated thermodynamics showed a reverse trend. This was ascribed mainly to the dominant role of sterics in the proximity of the metal center in governing the reaction energetics.

**Scheme 5:**
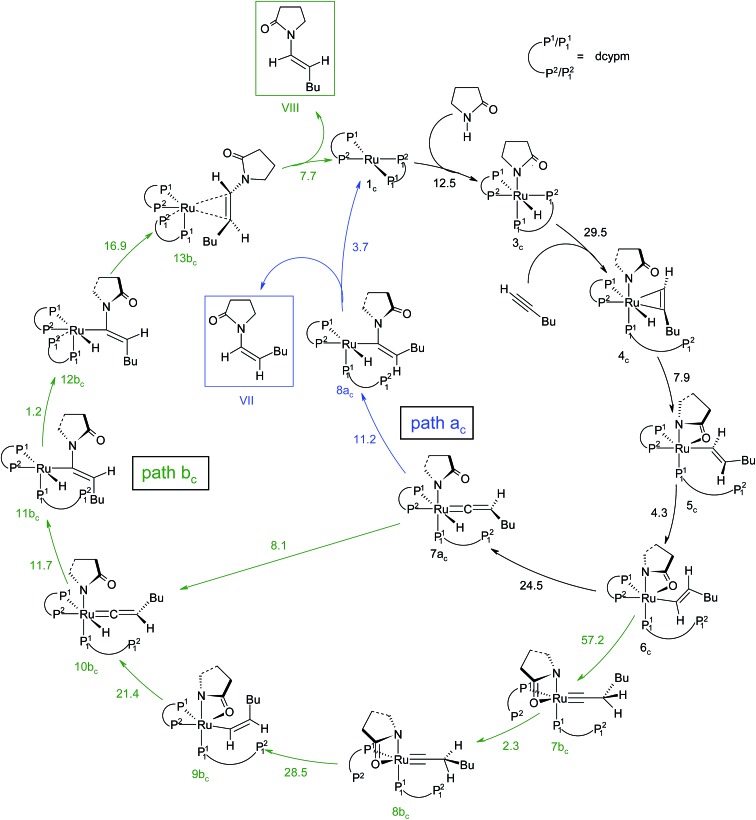
Computed catalytic cycle for the catalyst system **1_c_**. The energy values (Δ^‡^ *G*SDL, in kcal mol^–1^) represent the highest barrier for a given step. Blue (“path a_c_”) and green (“path b_c_”) lines indicate the *anti*-Markovnikov *Z*-product and *anti*-Markovnikov *E*-product formation pathways respectively.

**Fig. 11:**
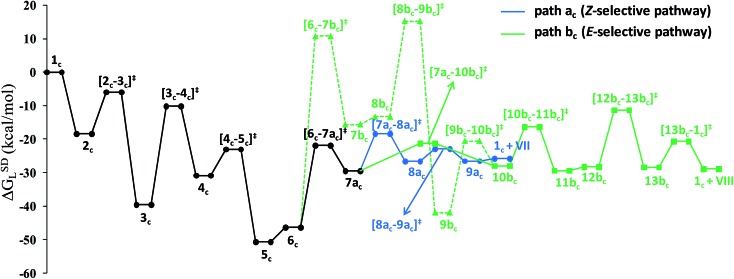
Overall reaction energy profile for the enamide formation using catalyst **1_c_**. The dotted green line shows alternative vinylidene formation steps from vinyl intermediate **6_c_**.

Overall, the catalytic cycle and reaction energies calculated for catalytic system **1_c_** are depicted in Scheme 5 and Fig. 11 respectively. Similarly to the previous catalytic system, two different pathways exist that both originate from a common intermediate **6_c_** but do not share a common reductive elimination route. Unlike **5**, which is a hexyne-coordinated η^2^ complex, **6_c_** is the vinyl intermediate formed after the hexyne insertion step (Fig. 2 and 6). For the catalytic system **1_c_**, the calculated pathways “path a_c_” (blue lines) and “path b_c_” (green lines) furnished exclusively two different stereoisomeric enamides (*Z*- and *E*-), that are contrary to those observed for the catalytic system **1** (Scheme 4, Fig. 5). “Path a_c_” and “path b_c_” involve two isomeric vinylidene complexes **7a_c_** and **11b_c_** in which the two hydrogen atoms (H^1^ and H^2^) are oriented *anti* and *syn* with respect to each other, which correspond to those observed in intermediates **8a** and **9b**. Closer inspection of the energy profile reveals that the major hydroamidation product for the catalytic system **1_c_** will be the *Z*-enamide **VII**. This result is in agreement with the experimental observation.

Neither the energetic span model nor the concept of a rate-determining highest transition state (HETS) can be used in unmodified form to decide which of the two possible stereoisomers will preferentially form, since both the highest energy span (step **3_c_** → **4_c_**) and the HETS **[3_c_–4_c_]^‡^** are identical for the pathways leading to the two isomers. They bifurcate only after reaching the vinylidene intermediate **7a_c_**, at a stage where the energy profile is relatively flat. In order to compare the rate of formation of the *E*- and *Z*-isomers, one must instead apply the models only to the pathways emanating from the bifurcation point **7a_c_**. This is a reasonable strategy since one can assume that the surrounding medium will fully absorb any excess energy of the common intermediate **7a_c_**.

Starting from the bifurcation point, formation of the *Z*-enamide involves a single activation barrier amounting to 11.2 kcal mol^–1^, so that the corresponding step **7a_c_** → **8a_c_** becomes rate-determining for this partial pathway. The largest energy span for this part of the cycle is between the TDI **7a_c_** and the TDTS **[7a_c_–8a_c_]^‡^** (11.2 kcal mol^–1^). Alternatively, formation of the *E*-enamide requires three activation barriers, among which that for step **11b_c_** → **13b_c_** (Δ^‡^ *G*SDL = 18.1 kcal mol^–1^) is rate-determining. For this partial pathway, the largest energy span is between the TDI **11b_c_** and the TDTS **[12b_c_–13b_c_]^‡^** (18.1 kcal mol^–1^).

Thus, both models predict that the formation of the *Z*-isomer should be favored, which is in excellent agreement with the experimental findings.

In their mechanistic investigation of olefin metathesis reactions using ruthenium(ii) carbene complexes,^49^ Thiel and Bühl found a dissociative pathway involving liberation of one phosphine to be the most favorable route. Since the hydroamidation pathway investigated herein has some mechanistic similarity, intermediates with only one dcypm ligand had to be considered. Fig. S34† depicts the reaction route containing dcypm decoordination, hexyne coordination and subsequent insertion from intermediate **3_c_**. Whereas the dissociation of the chelating phosphine is indeed exergonic by –15.4 kcal mol^–1^ (Δ*G*SL), the next hexyne coordination step involves a particularly high-energy intermediate **3_c__I_P** that is 40.0 kcal mol^–1^ above **3_c__I** (Fig. S34†), which renders this dissociative pathway unfavorable overall. A pair of isomeric 16e^–^ ruthenium(ii) vinylidene complexes **7a_c_-D** and **10b_c_-D** (Fig. S35†), obtained after removal of the dangling dcypm unit from **7a_c_** and **10b_c_** complexes, were also considered as starting points for the subsequent intramolecular nucleophilic attack of the amide moiety (Fig. 7 and 8). The activation energies for the steps **7a_c_-D** → **8a_c_-D** and **10b_c_-D** → **11b_c_-D** are as high as 23.1 and 21.3 kcal mol^–1^ (Δ*G*SL, Fig. S35†), values that are far greater than those for the analogous steps involving saturated 18e^–^ ruthenium(ii) intermediates (Scheme 5, **7a_c_** → **8a_c_**, 11.1 kcal mol^–1^; **10b_c_** → **11b_c_**, 11.7 kcal mol^–1^). It also has to be taken into account that regeneration of **1_c_** by re-coordination of the second dcypm ligand has a considerable entropic penalty (see Fig. S35,† **8a_c_-D** → **8a_c_**, **11b_c_-D** → **11b_c_**). Overall, the investigation of pathways involving coordinatively unsaturated ruthenium(ii) species did not lead to more favorable energy profiles.

The groups of Grubbs and Hoffmann have reported that deactivation of ruthenium alkylidene complexes can occur by various means including external phosphine attack or intramolecular coordination from a dangling nucleophilic center.^71^ Herein, the possibility for a similar deactivation route was considered, where the dangling phosphorus atoms in intermediates **7a_c_** and **11b_c_** were allowed to undergo intramolecular attack to the C^α^ of the vinylidene fragments. The intramolecular coordination of the P_1_^2^ following the steps **7a_c_** → **Pa_c_** and **11b_c_** → **Pb_c_** is facile with activation barriers below 11 kcal mol^–1^ (Δ*G*SL; refer to Fig. S36†). Similar reactivity was reported for *N*-phosphino-functionalized N-heterocyclic carbenes at RuCl_2_(PCy_3_)py_2_(CHPh) complexes.^71*c*^ In the resulting C^α^–P_1_^2^ coordinated intermediates **Pa_c_** and **Pb_c_**, the butyl group is in the orientation required for a stereo-selective nucleophilic attack of the 2-pyrrolidinyl unit (**Pa_c_** → **8′a_c_**, **Pb_c_** → **11′b_c_**; Fig. S36†). However, this step requires an excessive activation barrier of 34–37 kcal mol^–1^ (Fig. S36†). Therefore, **Pa_c_** and **Pb_c_** may be present in rapid equilibrium with **7a_c_** and **11b_c_**, but the nucleophilic attack to the vinylidene complexes is likely to proceed as depicted in Scheme 5.

### C.III. Steric and electronic factors influencing the stereoselectivity

**Fig. 12:**
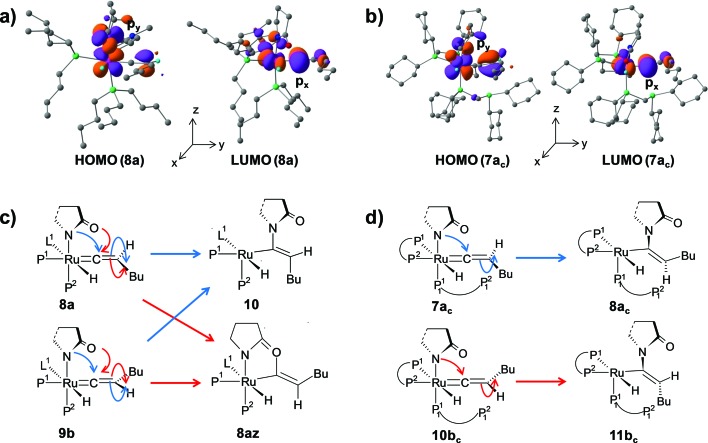
KS-MOs (isosurface = 0.048 au) of (a) **8a** and (b) **7a_c_**. Hydrogen atoms are omitted for clarity. Schemes showing the direction of butyl group rotation from vinylidene intermediates (c) **8a**, **9b** and (d) **7a_c_**, **10b_c_**.

The stereoselectivity of product formation for both catalytic systems (**1** and **1_c_**) is controlled in the intramolecular nucleophilic transfer step from the computed vinylidene intermediates. The KS-HOMO of vinylidenes (**8a**, **9b**, **7a_c_** and **10b_c_**, Fig. S32†) is mostly localized in the 2p_*y*_ orbital of nitrogen and oxygen of the 2-pyrrolidinyl unit, whereas the KS-LUMO resides in the 2p_*x*_ orbital of C^α^ atom (Fig. 12a and b for **8a**, **7a_c_**). Therefore, whenever we brought the nitrogen atom closer to C^α^, the Ru–C^β^–H^1^–Bu plane underwent rotation to match the HOMO–LUMO symmetry. This rotation allowed the incoming 2-pyrrolidine unit to move into the vinylidene plane with N–C^α^–C^β^–Bu dihedral angle of either 0° or 180° for products **VII** and **VIII**, respectively.

For catalytic system **1**, intermediates **8a** and **9b** were found to be equally likely to rotate the vinylidene plane during the nucleophilic transfer. However, in both cases, the butyl group turned *anti* to the nucleophile (N–C^α^–C^β^–Bu angle –174.4° in **10**), allowing us to predict that the steric interaction with the 2-pyrrolidinyl unit is more dominant than the *n*-butyl phosphine (P^2^). In contrast, in the case of oxygen attack to C^α^, the steric interaction of the butyl chain with the 2-pyrrolidinyl is greatly reduced compared to *n*-butyl phosphine, eventually generating the *Z*-product (see Fig. 12c and S14†).

For catalytic system **1_c_**, our calculated results showed the formation of different stereoisomeric products from the vinylidene intermediates **7a_c_** and **10b_c_** (Scheme 5). Although vinylidenes **10b_c_** and **9b** gave the same *E*-product, different stereoisomeric enamides were obtained from intermediates **7a_c_** and **8a** (Scheme 4 and Scheme 5). Formation of *Z*- and *E*-enamides from **7a_c_** and **10b_c_** can be explained after considering the steric interaction between the butyl chain with the decoordinated phosphine ligand (P_1_^2^) containing two bulky cyclohexyl groups (Cy) lying just under the vinylidene plane (Ru–C^β^–H^1^–Bu; Fig. 7 and 8). To conclusively prove the presence of such steric effects, we moved the decoordinated phosphine (P_1_^2^) from the vinylidene side to the H^2^ side by simple rotation along the Ru–P_1_^1^ bond to generate another isomer, which is isoenergetic to **7a_c_** (0.6 kcal mol^–1^ stable than **7a_c_**). Unfortunately, this gave the same *Z*-enamide after the nucleophilic transfer and reductive elimination steps. Our efforts in replacing the cyclohexyl groups of the dangling P_1_^2^ to methyl groups or hydrogens also resulted in the same *Z*-enamide product (**7a_c_-I**, **7a_c_-II**, Scheme S1†). We then reasoned that the presence of the –CH_2_P_1_^2^(R)_2_ (R = –Cy, –Me, –H) fragment may hinder the butyl chain to rotate into the position opposite to the incoming 2-pyrrolidinyl moiety. Hence, we replaced the –CH_2_P_1_^2^(Cy)_2_ by –Me (**7a_c_-V**, Scheme S1†), but to our surprise, we obtained the same stereoisomer. These findings prompted us to suspect the existence of steric influence from the other set of coordinated dcypm ligands (P^1^, P^2^). To this end, we modified structure **7a_c_-V** by replacing all the –Cy groups by –Me, and then performed the nucleophilic transfer step. Gratifyingly, the *E*-enamide product was formed justifying the absence of any spatial hindrance for the butyl group to bend downwards. These results suggest that the stereoselective outcome of the nucleophilic addition can be controlled by adjusting the steric bulk of the substituents at the phosphorous atoms.

We can also consider the steric influence of dcypm ligands from a different perspective. Closer inspection of the geometries of vinylidenes **7a_c_** and **10b_c_** reveals that the 2-pyrrolidinyl unit is inclined towards H^2^ atom (˪N–Ru–P_1_^1^ = 151.1°, 154.6° for **7a_c_** and **10b_c_** *vs.* 171.8°, 171.5° for **8a** and **9b** respectively) due to steric congestion by the bulky cyclohexyl groups present at the P^1^ and P^2^ atoms of the dcypm ligand (see Fig. 7 and 9a). Therefore, during the nucleophilic transfer, the 2-pyrrolidinyl unit turns to the *xy* plane and attacks the C^α^ atom, at which the 2p_*x*_ orbital comprising the LUMO is situated. Thus, the butyl fragments in **7a_c_** remain *syn* to the 2-pyrrolidinyl unit (dihedral angle N–Ru–C^β^–Bu = 5.6° in **8a_c_**) and *anti* in **10b_c_** (dihedral N–Ru–C^β^–Bu = –173.9° in **11b_c_**).

## C. Conclusion

Using DFT calculations, catalytic pathways for the ruthenium-catalyzed hydroamidation of 1-hexyne with pyrrolidinone were modeled both for a ruthenium catalyst bearing tri-*n*-butylphosphine and a complementary one bearing the sterically demanding bis(dicyclohexylphosphino)methane (dcypm) ligand. These two catalysts had been reported to give complementary stereoselectivities in catalytic hydroamidation reactions, an observation that could not be explained by spectroscopic investigations. The DFT calculations resulted in the discovery of plausible catalytic pathways for all possible regio- and stereoisomeric enamide products, which could then compared to each other to assess their relative rates.

For a catalyst generated *in situ* from [(cod)Ru(met)_2_], tri-*n*-butylphosphine and DMAP, both the energy span model and the concept of a rate-determining highest transition state predict that the reaction pathway leading to the *E*-configured *anti*-Markovnikov product should be favored over the competing pathway leading to the *Z*-configured enamide. This is in good agreement with the experimental findings. Pathways leading to the *anti*-Markovnikov product were found to involve highly endergonic steps, which are unlikely to proceed at the low reaction temperatures. This explains why in contrast to the addition of carboxylates to alkynes not even traces of *anti*-Markovnikov products are detected in Ru-catalyzed hydroamidations. The experimentally observed decisive influence of the DMAP additive on the catalyst activity was found to result from its unique ability to reversible coordinate to the ruthenium center facilitating a vinyl-vinylidene rearrangement.

Similar catalytic cycles were also computed for a catalyst bearing sterically demanding, chelating dcypm ligands. The pathways leading to *E*- and *Z*-configured *anti*-Markovnikov products were found to bifurcate relatively late after formation of a ruthenium vinylidene complex. The overall rate determining step and energy span were found to be within the common part of the catalytic cycle, namely within the initial hexyne coordination step. In order to allow us to compare the rates of *E*- and *Z*-product formation, we, thus, had to adapt the standard models to this rather unusual situation. This was done by comparing only the parts of the complementary pathways after the bifurcation. Applying this restriction, the model of the highest transition state and the energy span model both predicted the *Z*-selective pathway to be favorable for this catalyst system. This is in excellent agreement with experimental results.

The calculations for the first time provide an explanation why the stereoselectivity of ruthenium catalyzed hydroamidations can be controlled so remarkably well by the ligand system. They revealed that a strong steric interaction between the dcypm ligand and the vinylidene group is the key factor that causes the reversal of the stereoselectivity when substituting *n*-butylphosphine with chelating dcypm ligands. During the nucleophilic attack of the amide to the vinylidene carbon **7a_c_**, the carbon side chain, which was previously oriented *syn* to the hydride substituent, is forced into a *Z*-configuration by the steric bulk of the chelating ligand. This effect is not observed for the sterically less crowded tri-*n*-butylphosphine.

This detailed understanding of the reaction mechanism will serve as the basis for the rational design of hydroamidation catalysts with a new level of activity and stereoselectivity.

## D. Conflict of interest

The authors declare no competing financial interests.
