# Supplementary material for: Computational study of the mechanism and selectivity of ruthenium-catalyzed hydroamidations of terminal alkynes
Source: Chem Sci. 2015 Feb 18;6(4):2532–52. doi: 10.1039/c4sc03906h (PMC5539791; doi:10.1039/c4sc03906h)
Supplement: Supplementary file 1 [file SC-006-C4SC03906H-s001.pdf]

Electronic Supplementary Material (ESI) for Chemical Science.
This journal is © The Royal Society of Chemistry 2015

Computational Study of the Mechanism and Selectivity of the Ru(II) Catalyzed Hydroamidation Reaction of Terminal Alkynes

BholanathMaity,[†] Lukas J. Gooßen,*[,‡] and DebasisKoley*[,†]

[‡]FachbereichChemie – OrganischeChemie,
TechnischeUniversität Kaiserslautern,
Erwin-Schrödinger-StraβeGeb.54, D-67663 Kaiserslautern, Germany.
Fax: (+49) 631-205-3921, E-mail: goossen@chemie.uni-kl.de

[†]Department of Chemical Sciences,
Indian Institute of Science Education and Research (IISER) Kolkata,
Mohanpur 741246, India.
E-mail: koley@iiserkol.ac.in

SUPPORTING INFORMATION

Contents

1. Complete reference of Gaussian 03 and Gaussian 09. S3

2. Computed single point energy profile with dispersion corrected functionals (BP86-D, B97D and M06-2X) (Figure S1→Figure S2). S3–4

3. Energy profile and optimized structures of intermediates and transition states involved in catalytic cycle for the catalyst system **1** (Figure S3→Figure S15). S5–11

4. Chemical structures for alkyne coordinated compound (**5**) and its two different vinyl isomers (**6** and **6$_I$**) (Figure S16). S11

5. Energy profile with dispersion effect for the catalyst system **1** (Figure S17). S12

6. Energy profile and optimized structures of intermediates and transition states involved in catalytic cycle for the catalyst system **1$_c$** (Figure S18→Figure S30). S12–18

7. Energy profile with dispersion effect for the catalyst system **1** (Figure S31). S19

8. KS-MO of some important intermediates (Figure S32). S19–20

9. Chemical structures of several modified vinylidene complexes from **7a$_c$** and their respective enamide products (Scheme S1). S20

10. Absolute energies (hartree) of all intermediates and transition states involve in catalyst system **1** and $\mathbf{1_c}$ (Table S1→Table S2). S20–24

11. Energy changes (in kcal/mol) for all steps involved in catalyst system **1** and $\mathbf{1_c}$ (Table S3→Table S6). S24–27

12. NPA charges of selected atoms for all intermediates and transition states involved in the reaction pathways for catalyst **1** and $\mathbf{1_c}$ (Table S5→Table S6). S28–31

13. Plot of NPA charge on ruthenium center of intermediates and transition states involved in different pathways for catalyst **1** and $\mathbf{1_c}$ (Figure S33). S31

14. Reaction path involving dcypm decoordination, hexyne coordination and insertion step from ruthenium(II) intermediate $\mathbf{3_c}$.The energies (ΔG_L^S) above the arrows are in kcal/mol (Figure S34). S31

15 Nucleophilic transfer steps from 16e$^-$ vinylidene intermediates $\mathbf{7a_c}$**-D** and $\mathbf{10b_c}$**-D**. The energies (ΔG_L^S) above the arrows are in kcal/mol (Figure S35). S32

16. Reaction path involving C_α–P bond formation and nucleophilic transfer steps from vinylidene intermediates $\mathbf{7a_c}$ and $\mathbf{10b_c}$. The energies (ΔG_L^S) above the arrows are in kcal/mol (Figure S36). S32

17. Cartesian coordinates (Å) of the optimized structures of all intermediates and transition states involved in catalytic system **1** (Table S7). S32–75

18. Cartesian coordinates (Å) of the optimized structures of all intermediates and transition states involved in catalytic system $\mathbf{1_c}$ (Table S7). S76–134

Complete Reference of Gaussian03 and Gaussian 09

Gaussian 03, Revision **E.01**, M. J. Frisch, G. W. Trucks, H. B. Schlegel, G. E. Scuseria, M. A. Robb, J. R. Cheeseman, Jr., J. A. Montgomery, T. Vreven, K. N. Kudin, J. C. Burant, J. M. Millam, S. S. Iyengar, J. Tomasi, V. Barone, B. Mennucci, M. Cossi, G. Scalmani, N. Rega, G. A. Petersson, H. Nakatsuji, M. Hada, M. Ehara, K. Toyota, R. Fukuda, J. Hasegawa, M. Ishida, T. Nakajima, Y. Honda, O. Kitao, H. Nakai, M. Klene, X. Li, J. E. Knox, H. P. Hratchian, J. B. Cross, V. Bakken, C. Adamo, J. Jaramillo, R. Gomperts, R. E.Stratmann, O. Yazyev, A. J. Austin, R. Cammi, C. Pomelli, J. W. Ochterski, P. Y. Ayala, K. Morokuma, G. A. Voth, P. Salvador, J. J. Dannenberg, V. G. Zakrzewski, S. Dapprich, A. D. Daniels, M. C. Strain, O. Farkas, D. K. Malick, A. D. Rabuck, K. Raghavachari, J. B. Foresman, J. V. Ortiz, Q. Cui, A. G. Baboul, S. Clifford, J. Cioslowski, B. B. Stefanov, G. Liu, A. Liashenko, P. Piskorz, I. Komaromi, R. L. Martin, D. J. Fox, T. Keith, M. A. Al-Laham, C. Y. Peng, A. Nanayakkara, M. Challacombe, P. M. W. Gill, B. Johnson, W. Chen, M. W. Wong, C. Gonzalez, and J. A. Pople, Gaussian, Inc., Wallingford CT, 2004.

Gaussian09, Revision **C.01**, M. J. Frisch, G. W. Trucks, H. B. Schlegel, G. E. Scuseria, M. A. Robb, J. R. Cheeseman, G. Scalmani, V. Barone, B. Mennucci, G. A. Petersson, G. H. Nakatsuji, M. Caricato, X. Li, H. P. Hratchian, A. F. Izmaylov, J. Bloino, G. Zheng, J. L. Sonnenberg, M. Hada, M. Ehara, K. Toyota, R. Fukuda, J. Hasegawa, M. Ishida, T. Nakajima, Y. Honda, O. Kitao, H. Nakai, T. Vreven, Jr. J. A. Montgomery, J. E. Peralta, F. Ogliaro, M. Bearpark, J. J. Heyd, E. Brothers, K. N. Kudin, V. N. Staroverov, R. Kobayashi, J. Normand, K. Raghavachari, A. Rendell, J. C. Burant, S. S. Iyengar, J. Tomasi, M. Cossi, N. Rega, J. M. Millam, M. Klene, J. E. Knox, J. B. Cross, V. Bakken, C. Adamo, J. Jaramillo, R. Gomperts, R. E. Stratmann, O. Yazyev, A. J. Austin, R. Cammi, C. Pomelli, J. W. Ochterski, R. L. Martin, K. Morokuma, V. G. Zakrzewski, G. A. Voth, P. Salvador, J. J. Dannenberg, S. Dapprich, A. D. Daniels, Ö. Farkas, J. B. Foresman, J. V. Ortiz, J. Cioslowski and D. J. Fox, Gaussian, Inc., Wallingford CT, 2010.

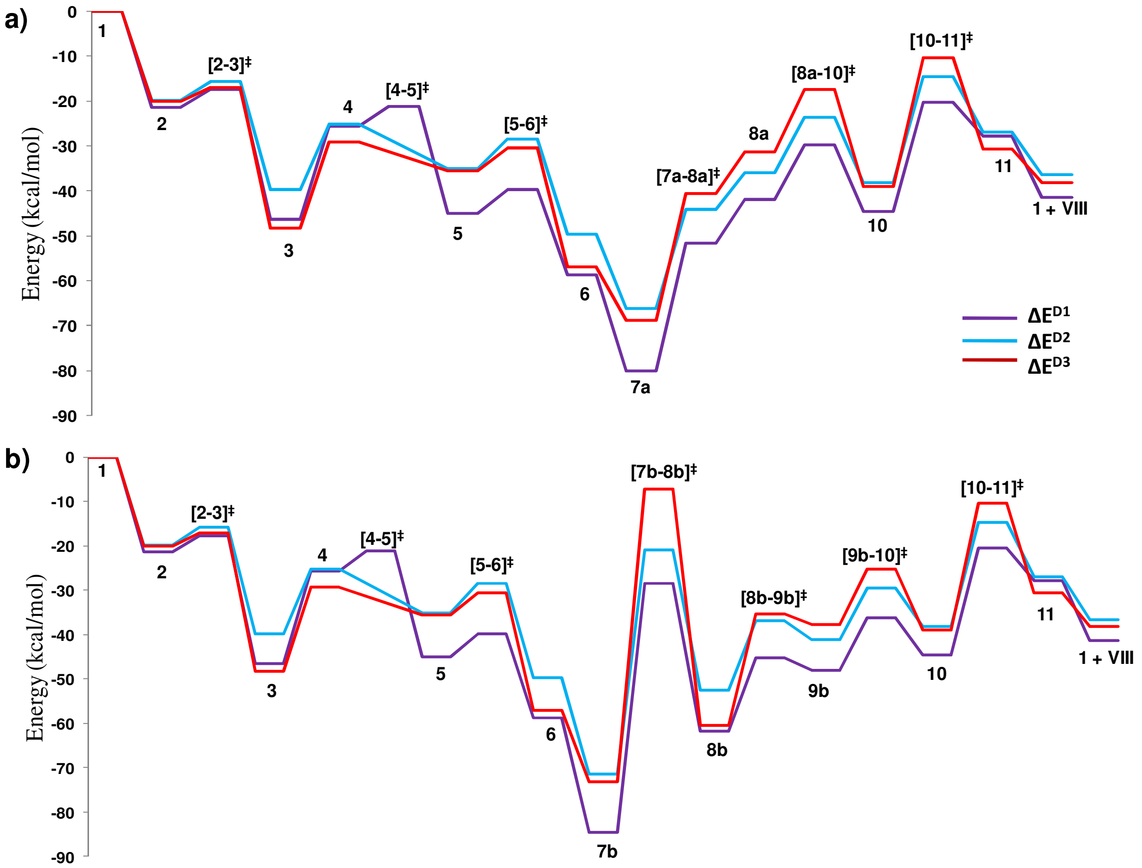

Figure S1. Computed single pointenergy profile for a) "path a" and b) "path b" in catalyst system **1** with functional BP86-D (ΔE^{D1}, purple), B97D (ΔE^{D2}, light blue) and M06-2X (ΔE^{D3}, red) at higher basis sets

LANL2TZ(f)(Ru)/TZVP(H, C, N, O and P) on the optimized geometry at BP86/LANL2DZ(Ru)/6-31G*(H, C, N, O and P) level of theory.

Note: To elucidate the dispersion effect we have performed single point calculations at higher basis set using the functionals BP86-D, B97D and M06-2X on the optimized geometries at BP86/LANL2DZ(Ru)/6-31G*(H, C, N, O and P) level. The computed energy profile incorporating the dispersion corrected energies (BP86-D) shows good agreement with the energy trend calculated with BP86 functional. Unfortunately only during the **4→5** step, both B97D and M06-2X methods could not validate the existence of **[4-5]**‡ transition state. Additionally, our efforts in optimizing the same transition states using B97D and M06-2X functionals failed even after repeated attempts.

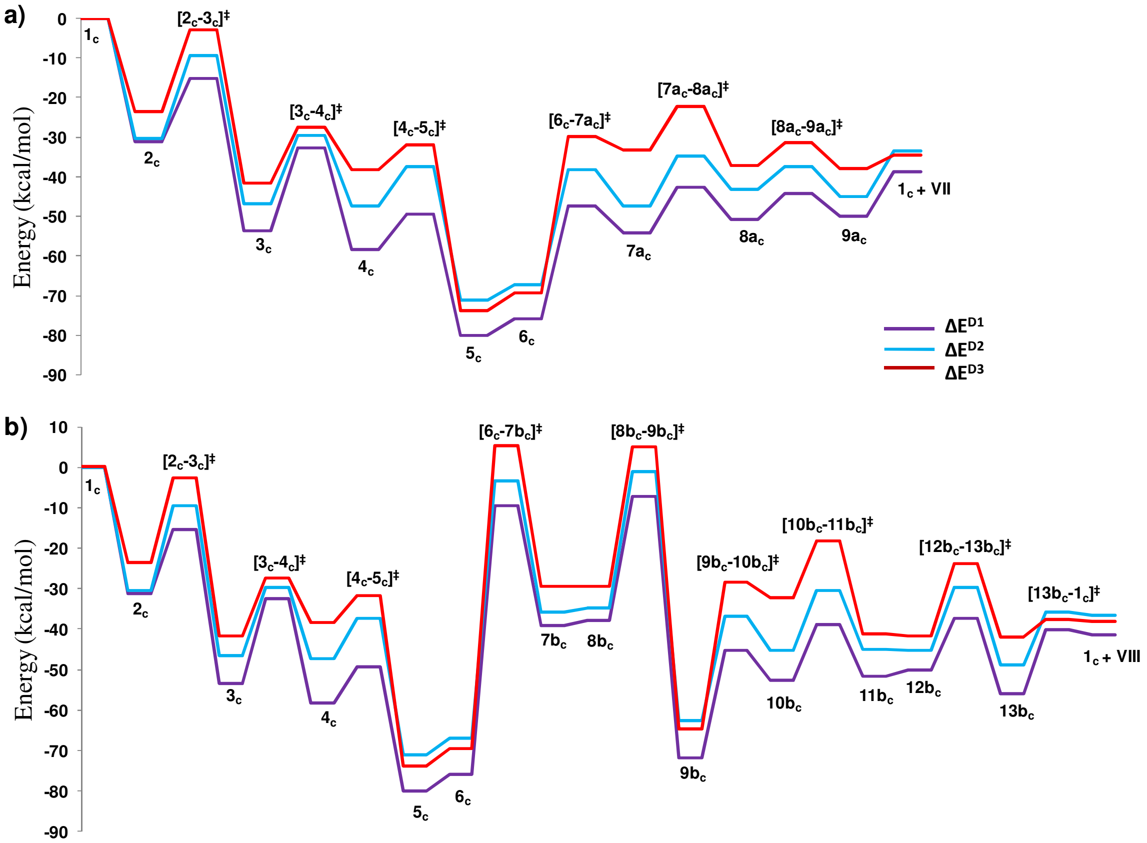

Figure S2. Computed single point energy profile for a) "path a_c" and b) "path b_c" in catalyst system**1**$_c$ with functional BP86-D (ΔE^{D1}, purple), B97D (ΔE^{D2}, light blue) and M06-2X (ΔE^{D3}, red) at higher basis sets LANL2TZ(f)(Ru)/TZVP(H, C, N, O and P) on geometries optimized at ONIOM{BP86/LANL2DZ(Ru)/6-31G*(H, C, N, O and P):HF/STO-3G} level of theory.

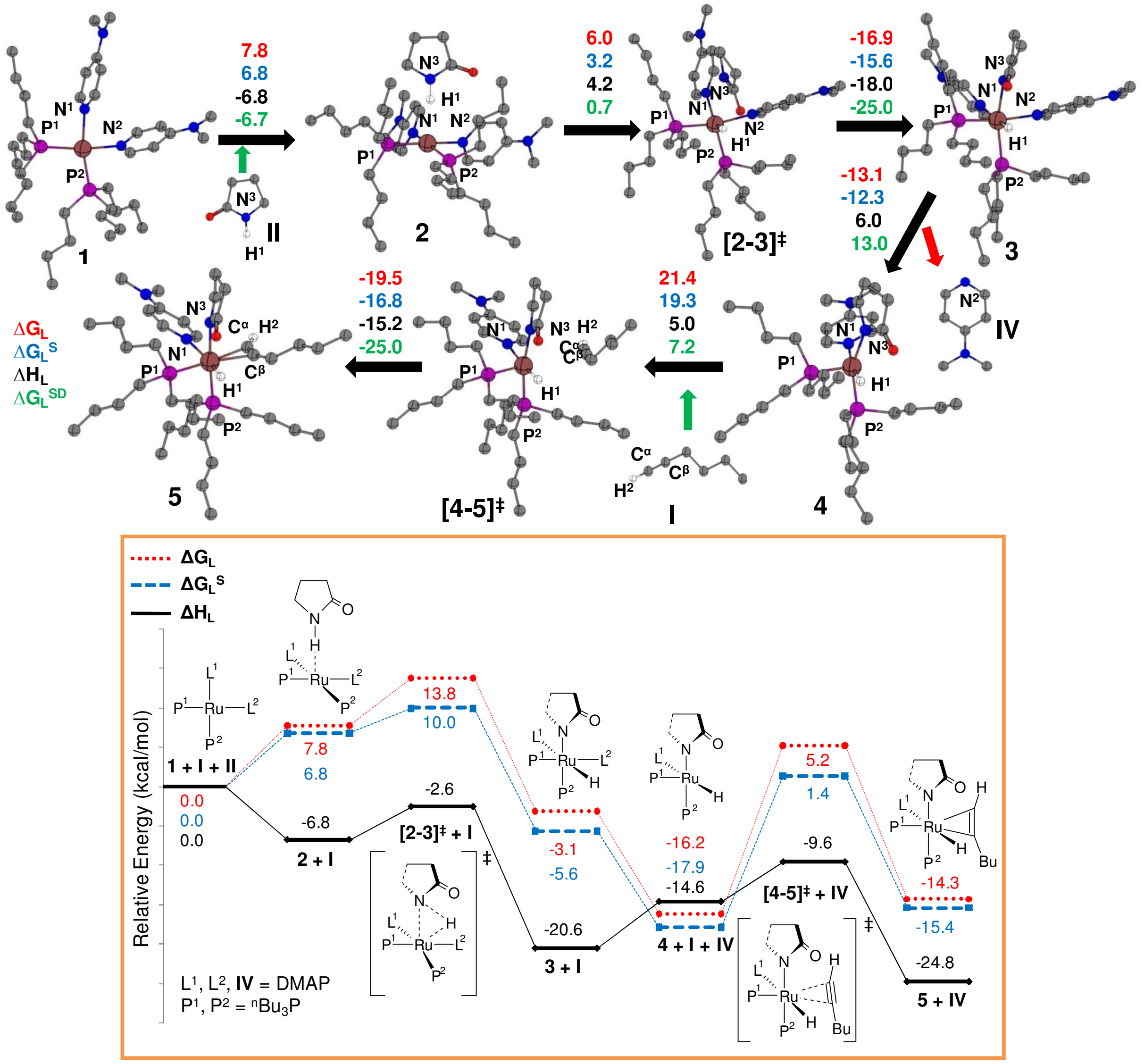

Figure S3. Optimized structures and energy profile (inset) for the oxidative addition (**1**→**3**) and hexyne coordination (**4**→**5**) steps. For energy nomenclature, refer to Computational Details. For other conventions refer to Figure 1.

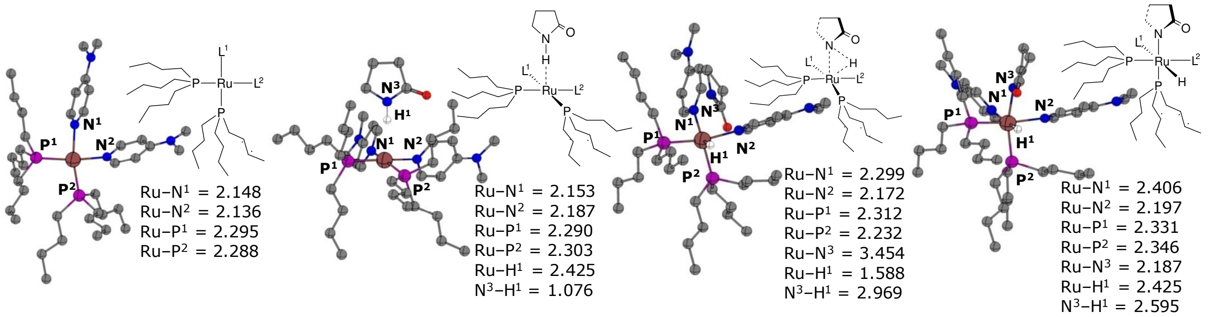

1 (0.0/0.0/0.0) **2 (-6.8/7.8/6.8)** **[2-3]‡ (-2.6/18.8/10.0)** **3 (-20.6/-4.1/-5.6)**

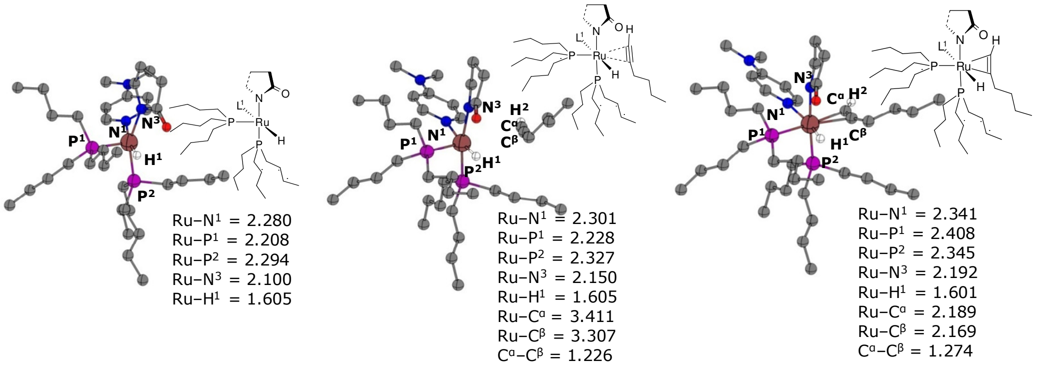

4 (-14.6/-16.2/-17.9) [4-5]‡ (-9.6/5.2/1.4) **5** (-24.8/-14.3/-15.4)

Figure S4. Optimized Structures of intermediates and transition states involved in oxidative addition and hexyne coordination steps for catalyst system **1**. Key bond lengths are in angstroms (Å), bond angles and dihedral angles are in degrees (°). All hydrogen atoms (except H^1 and H^2) are omitted for clarity. Energies (ΔH_L/ ΔG_L/ΔG_L^S) in kcal/mol are given in parentheses. For color code refer Figure 1 in main text.

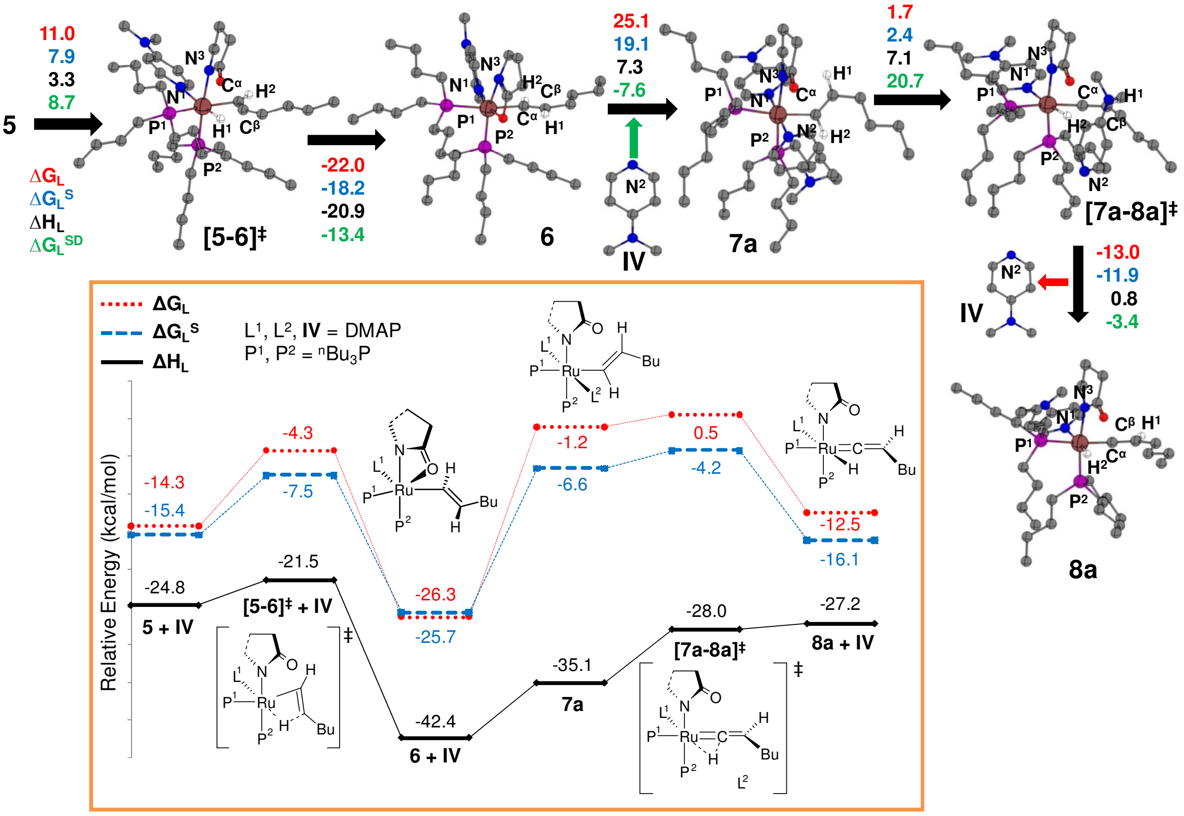

Figure S5. Optimized structures and energy profile (inset) for the hexyne insertion and vinyl-vinylidene rearrangement steps in "path a". For other conventions refer to Figure 1 and Figure S3.

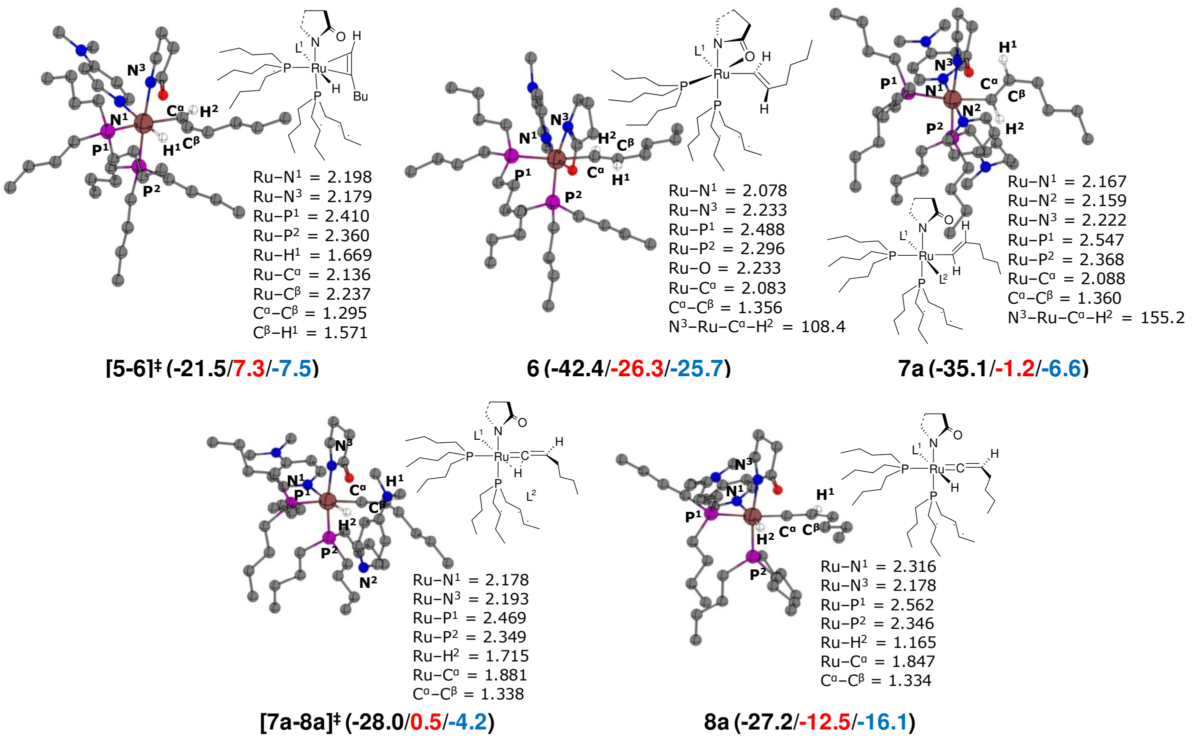

Figure S6. Optimized Structures of intermediates and transition states involved in hexyne insertion and vinyl-vinylidene rearrangement steps in "path a" for catalyst system **1**. For energy and other conventions refer Figure 1 and Figure S3.

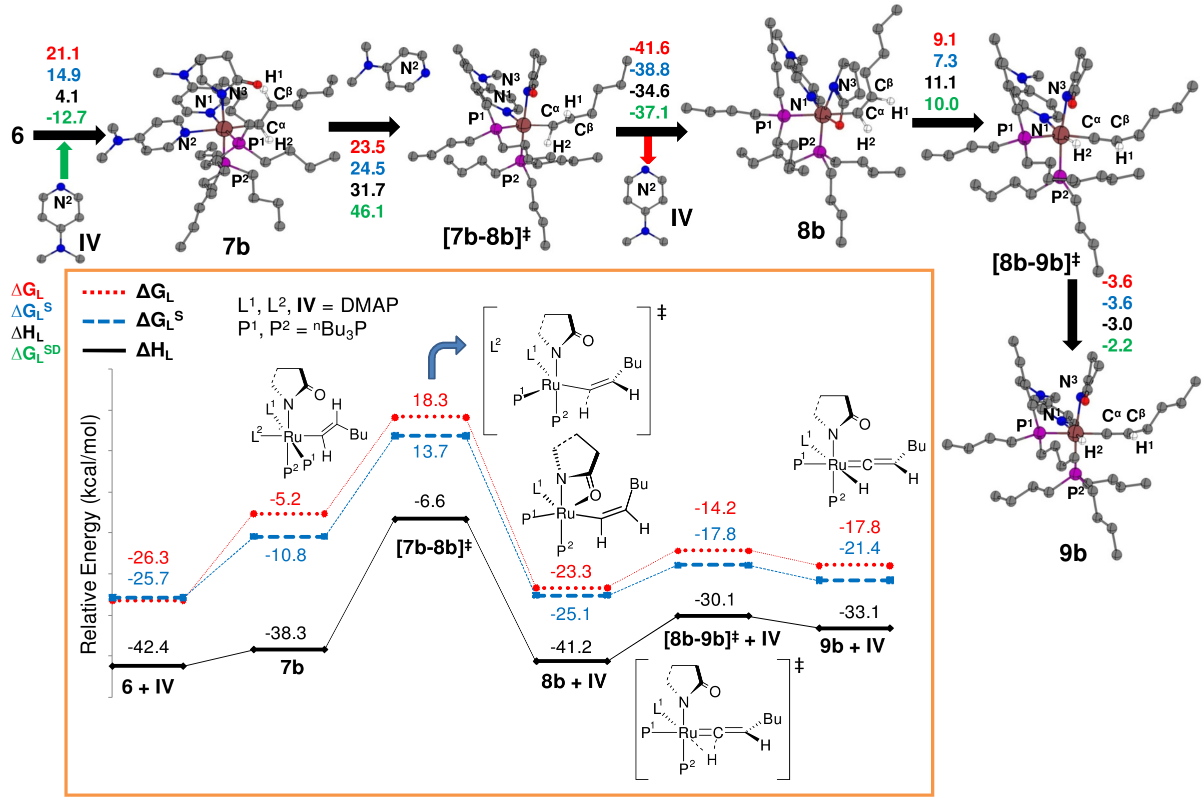

Figure S7. Optimized structures and energy profile (inset) for the hexyne insertion and vinyl-vinylidene rearrangement steps in "path b". For other conventions refer to Figure 1 and Figure S3.

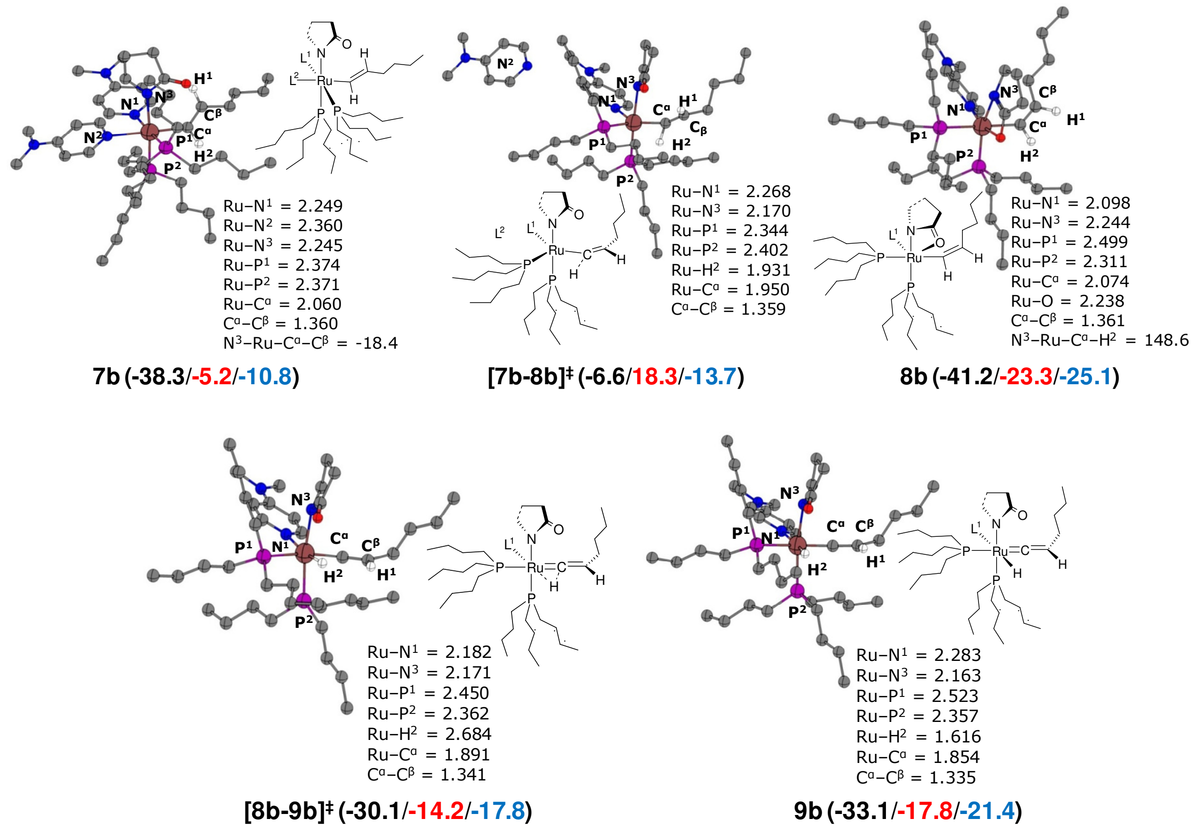

Figure S8. Optimized Structures of intermediates and transition states involved in hexyne insertion and vinyl-vinylidene rearrangement steps in "path b" for catalyst system **1**. For energy and other conventions refer Figure 1 and Figure S3.

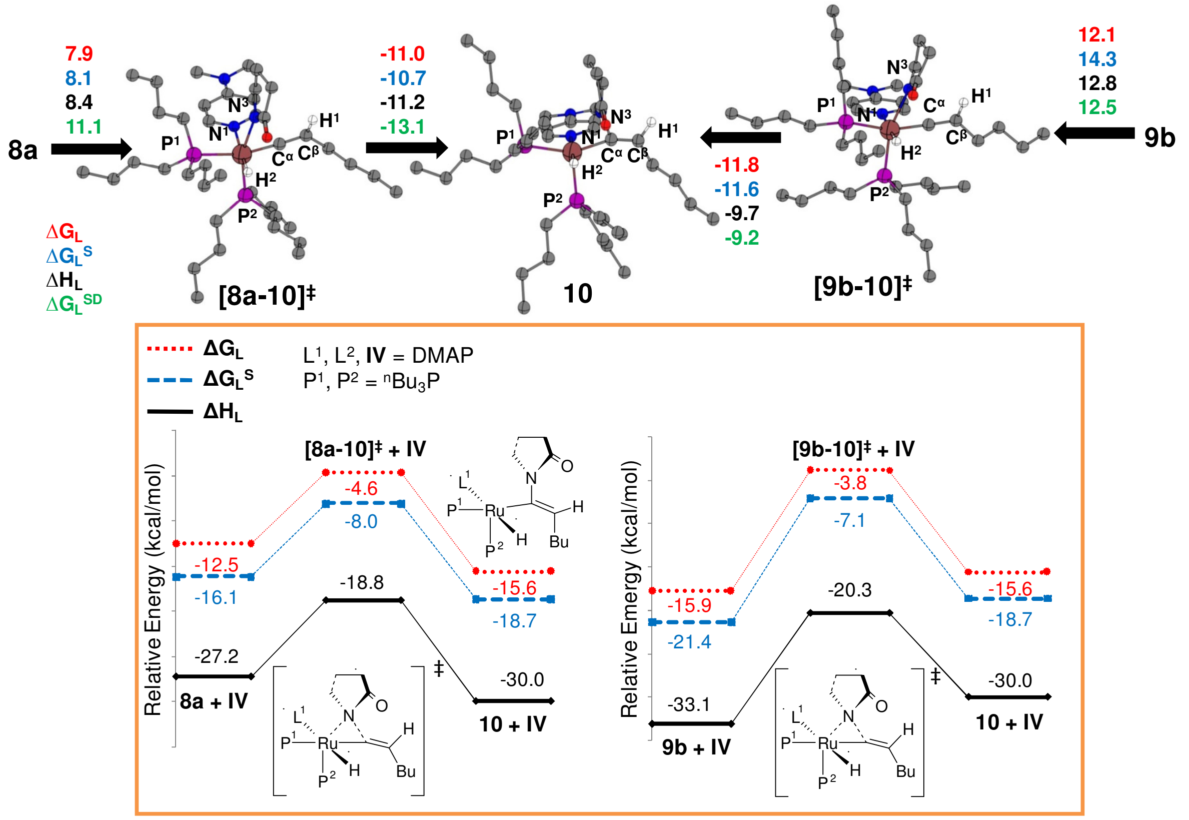

Figure S9. Optimized structures and energy profile (inset) for the nucleophilic transfer steps in “path a” and “path b”. For other conventions refer to Figure 1 and Figure S3.

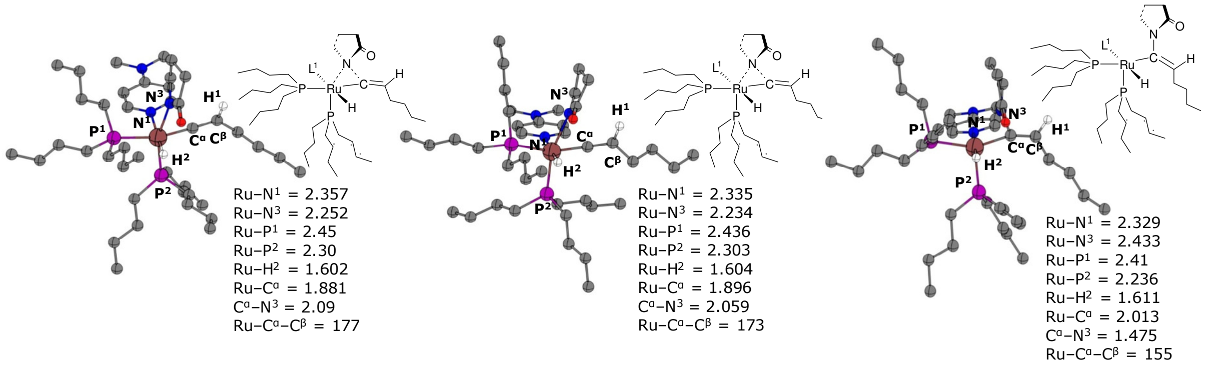

[8a-10]‡ (-18.8/-4.6/-8.0) **[9b-10]‡ (-20.3/-3.8/-7.1)** **10 (-30.0/-15.6/-18.7)**

Figure S10. Optimized Structures of intermediates and transition states involved in nucleophilic attack step in both “path a” and “path b” for catalyst system **1**. For energy and other conventions refer Figure 1 in main text and Figure S3.

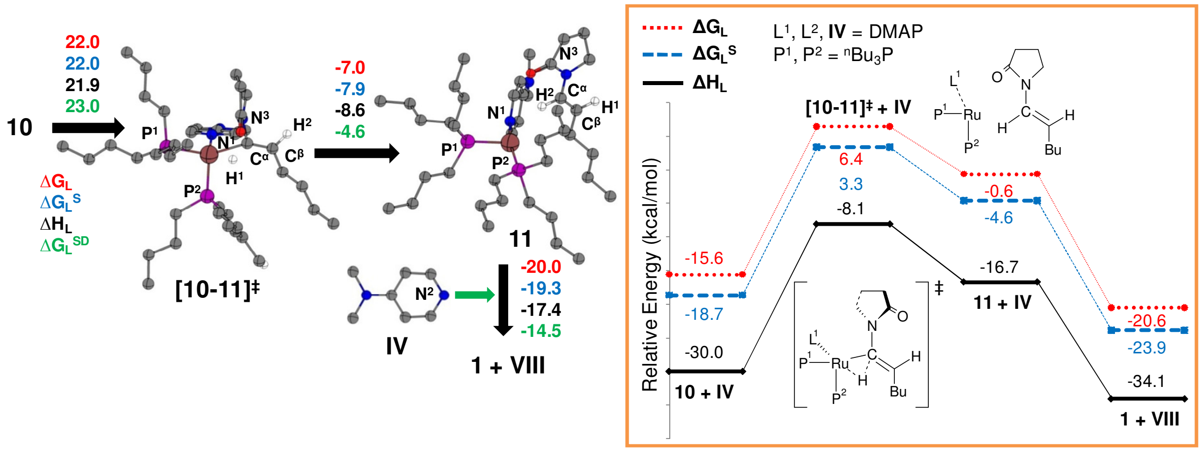

Figure S11. Optimized structures and energy profile (inset) for the reductive elimination steps. For other conventions refer to Figure 1 and Figure S3.

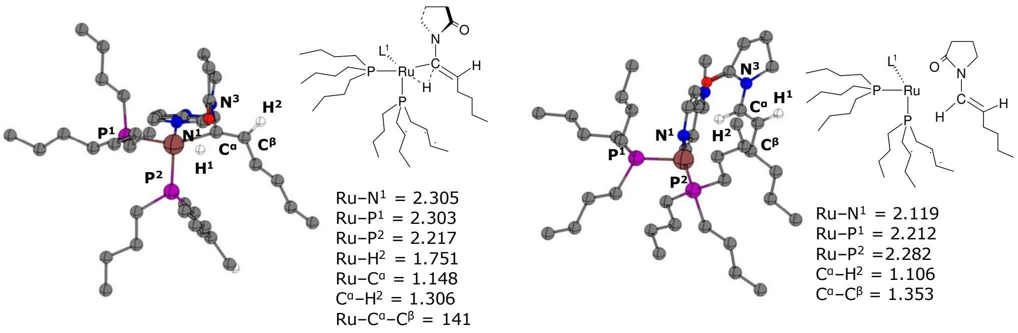

[10-11]‡ (-8.1/6.4/3.3) **11 (-16.7/-0.6/-7.6)**

Figure S12. Optimized Structures of intermediates and transition states involved in reductive elimination step for catalyst system **1**. For energy and other conventions refer Figure 1 in main text and Figure S3.

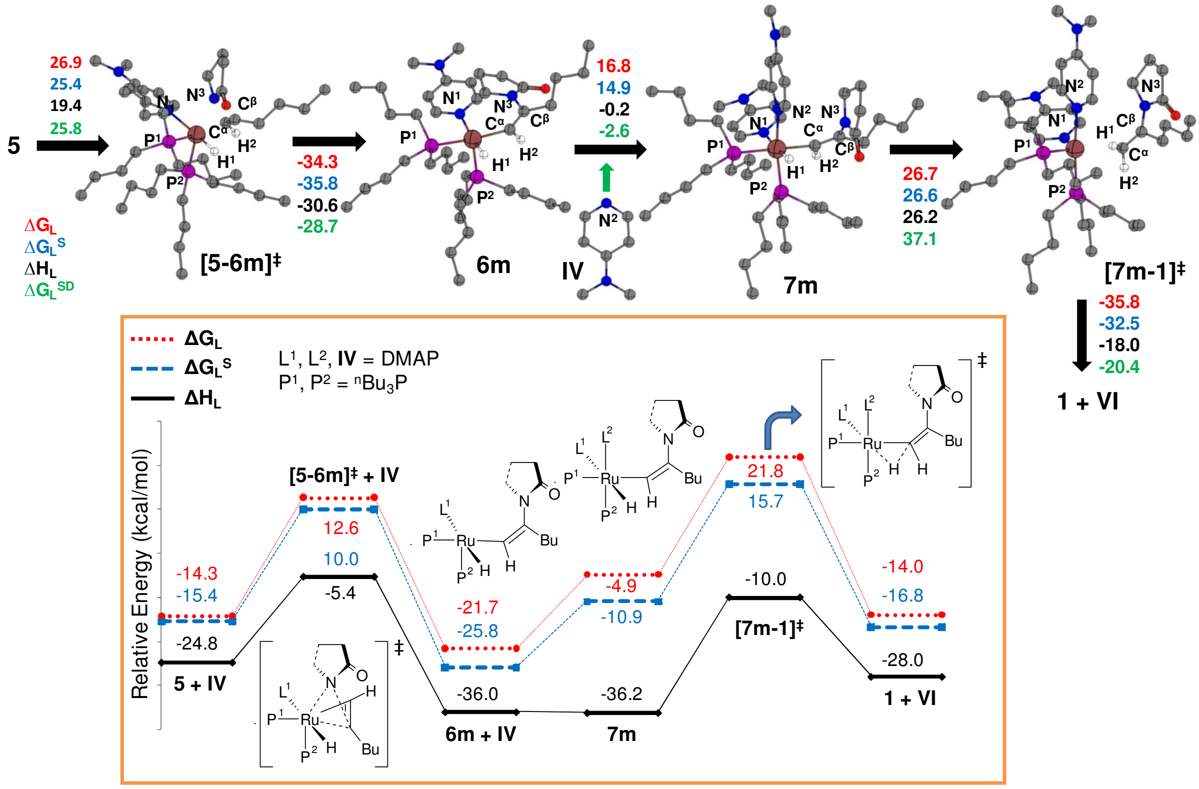

Figure S13. Optimized structures and energy profile (inset) for the Markovnikov product formation pathway. For other conventions refer to Figure 1 and Figure S3.

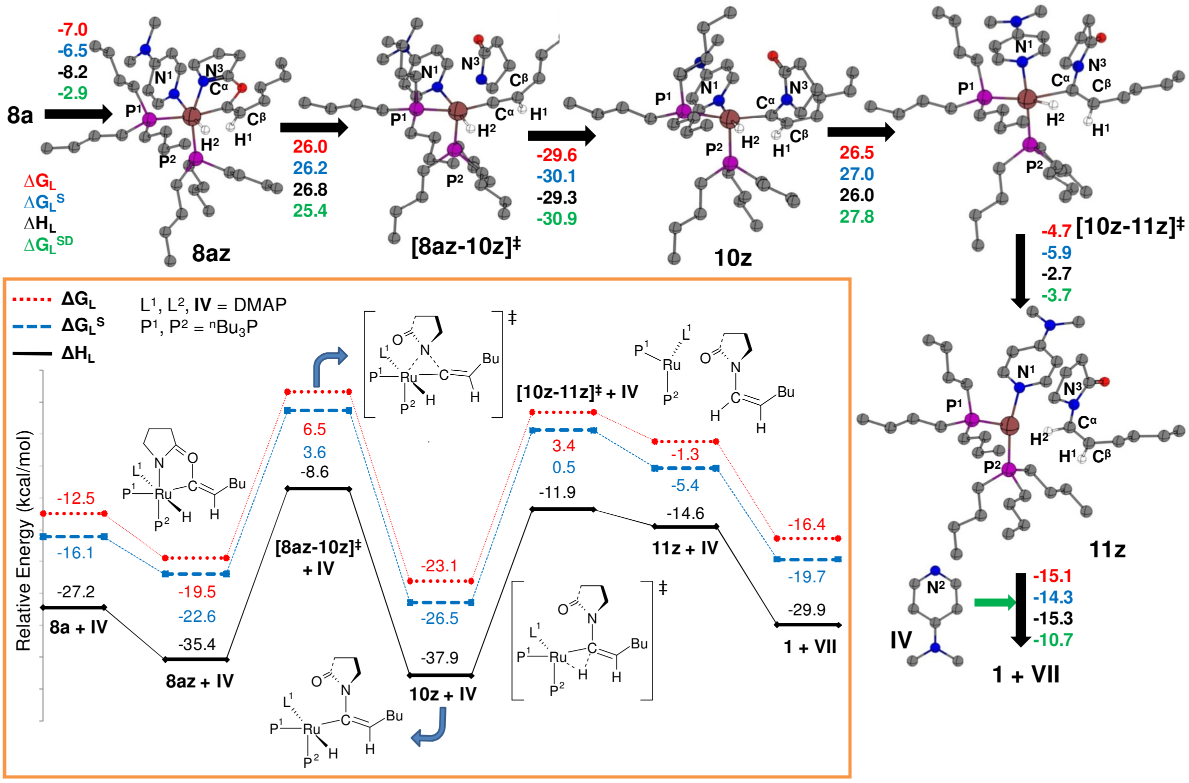

Figure S14. Optimized structures and energy profile (inset) for the *Z*-enamide product formation pathway. For other conventions refer to Figure 1 and Figure S3.

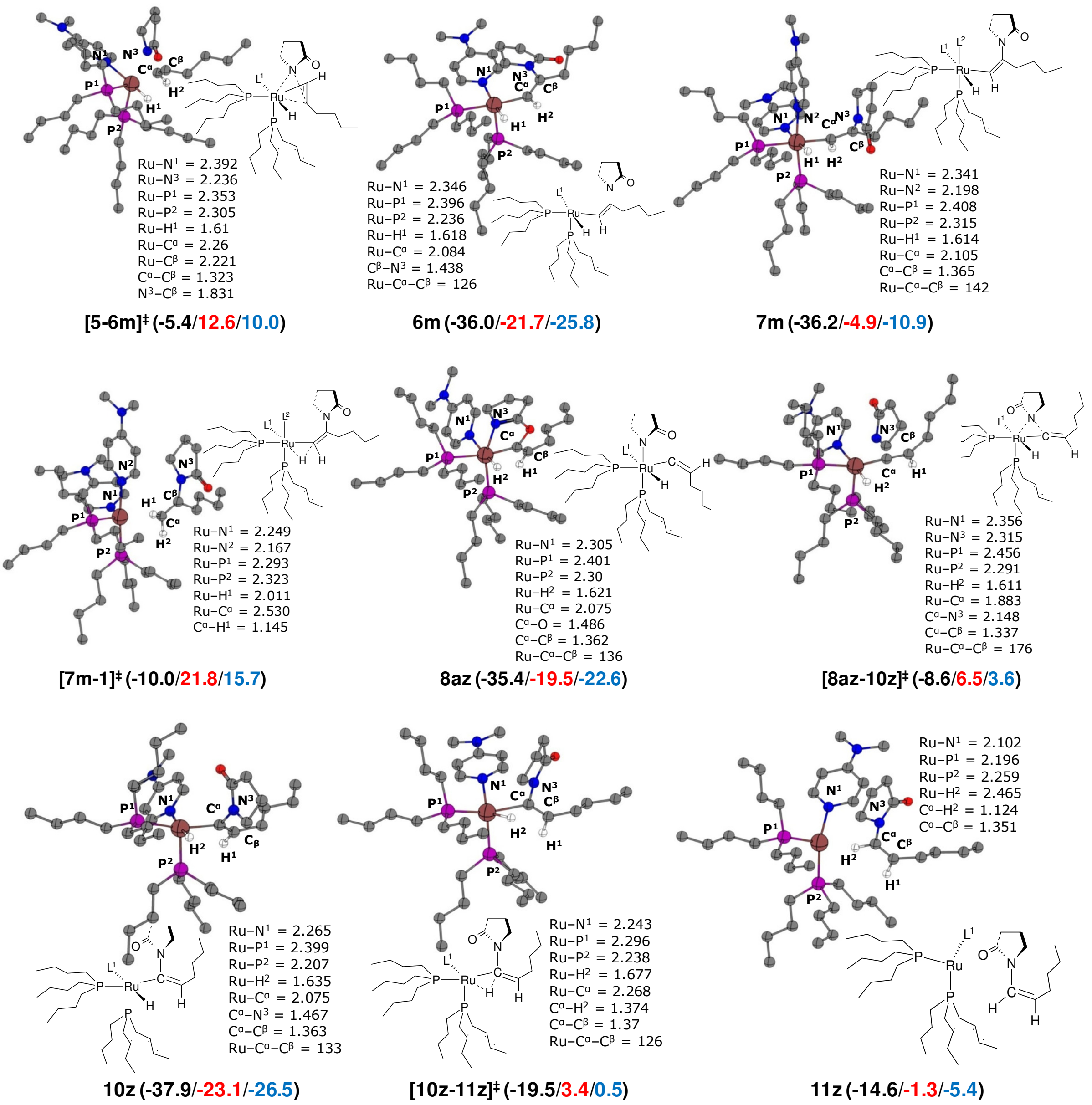

Figure S15. Optimized Structures of intermediates and transition states involved in Markovnikov product and *Z*-selective product formation. For energy and other conventions refer Figure 1 in main text and Figure S3.

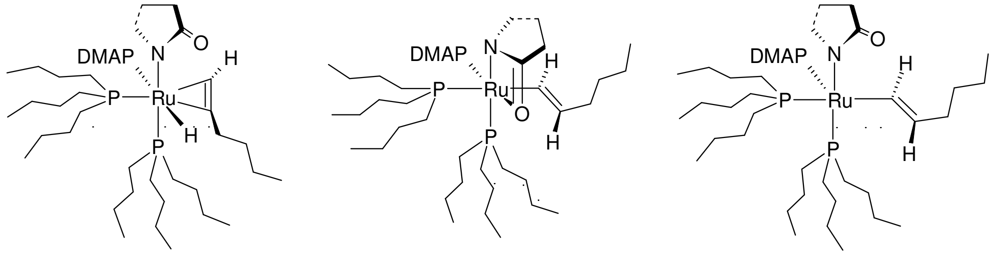

5 (0.0/0.0) **6** (-17.6/-12.0) **6_I** (-3.2/-3.6)

Figure S16. Chemical structures for alkyne coordinated compound (**5**) and its two different vinyl isomers (**6** and **6_I**). Energy values ($ΔH_L$/$ΔG_L$) in kcal/mol at BP86/LANL2DZ(Ru)/6-31G*(H, C, N, O and P)

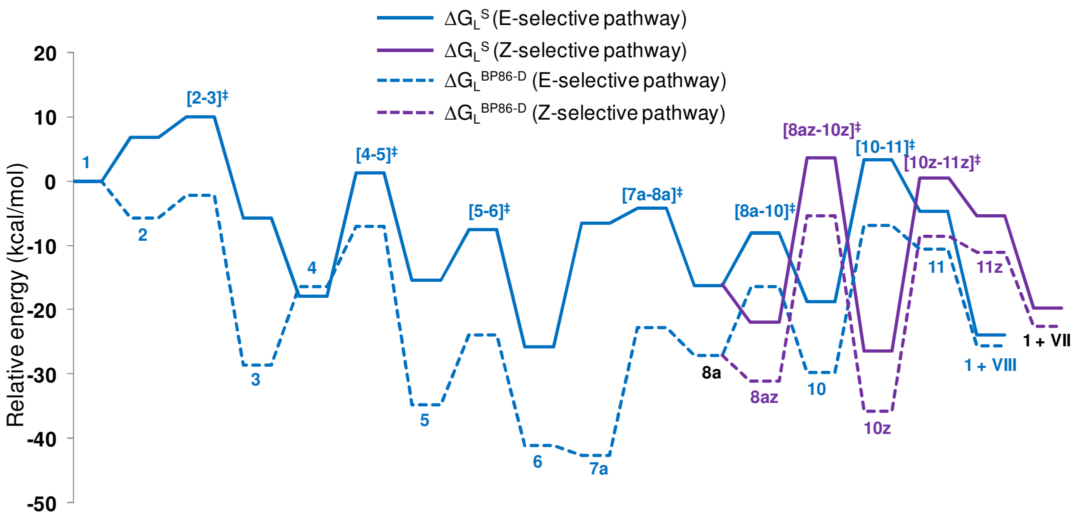

Figure S17. Comparison of the dispersion corrected energy profile (BP86-D/LANL2TZ(f)(Ru)/TZVP(H, C, N, O and P)//BP86/LANL2DZ(Ru)/6-31G*(H, C, N, O and P); dotted line) with the one reported in the text (BP86/LANL2TZ(f)(Ru)/TZVP(H, C, N, O and P)//BP86/LANL2DZ(Ru)/6-31G*(H, C, N, O and P); dark line) for favorable pathways of *E*- and *Z*- enamide formation in catalyst system **1**. For energy terms refer to the computational details.

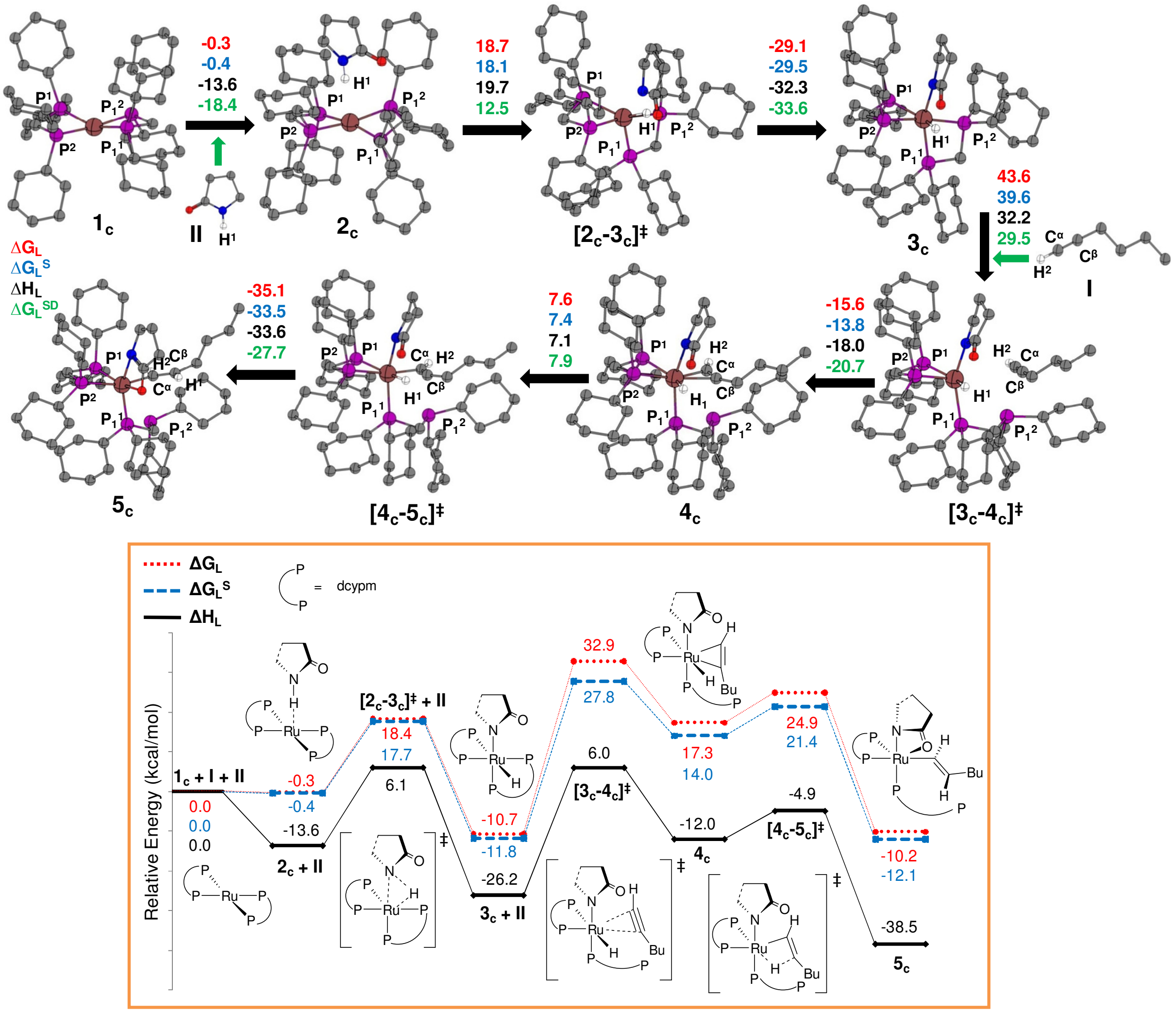

Figure S18. Optimized structures and energy profile (inset) for the oxidative addition and hexyne insertion steps for catalytic system $\mathbf{1_c}$. For other conventions refer to Figure 1 and Figure S3.

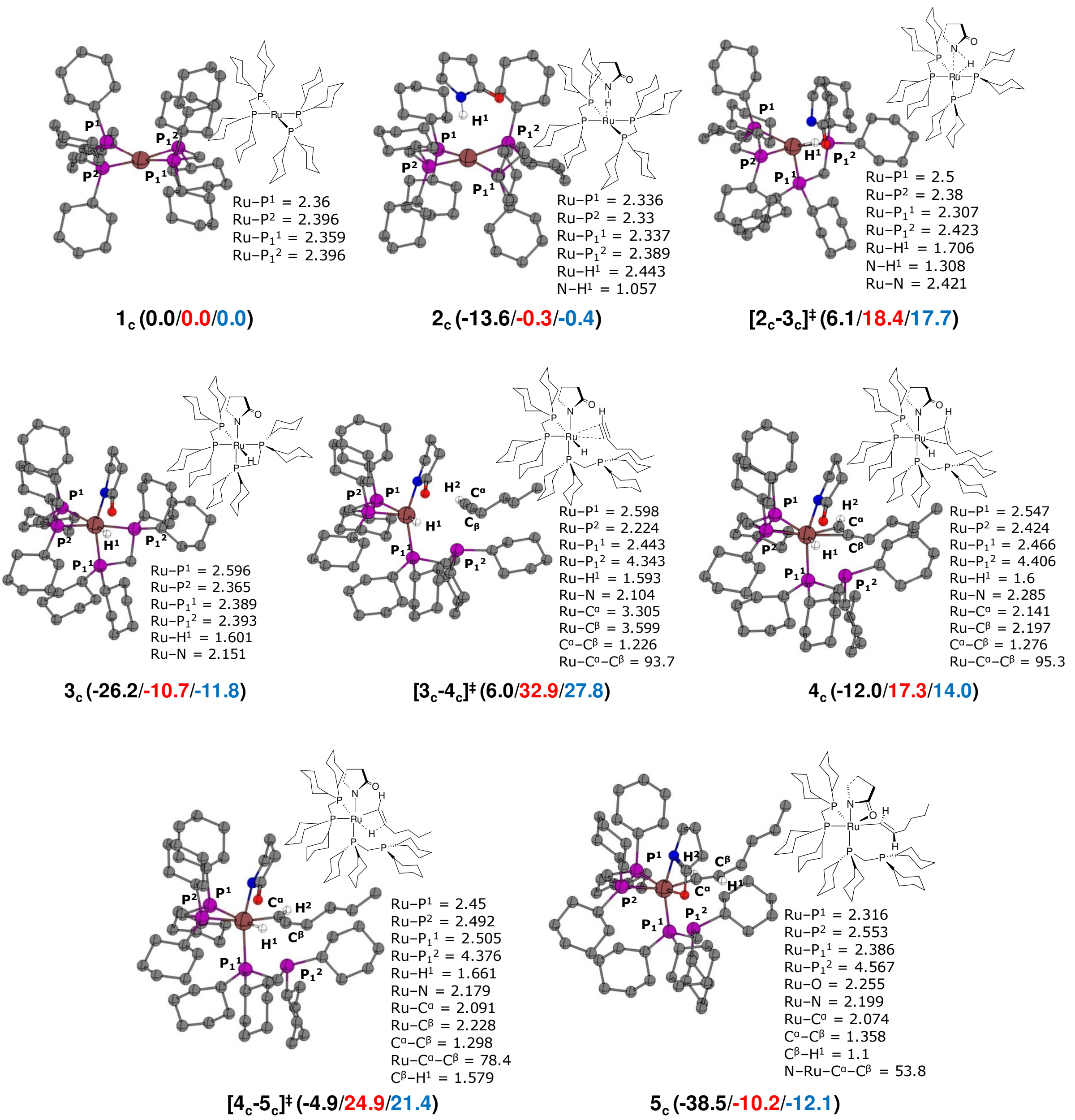

Figure S19. Optimized Structures of intermediates and transition states involved in oxidative addition, hexyne coordination and insertion steps for catalyst system $\mathbf{1_c}$. For energy and other conventions refer Figure 1 in main text and Figure S3.

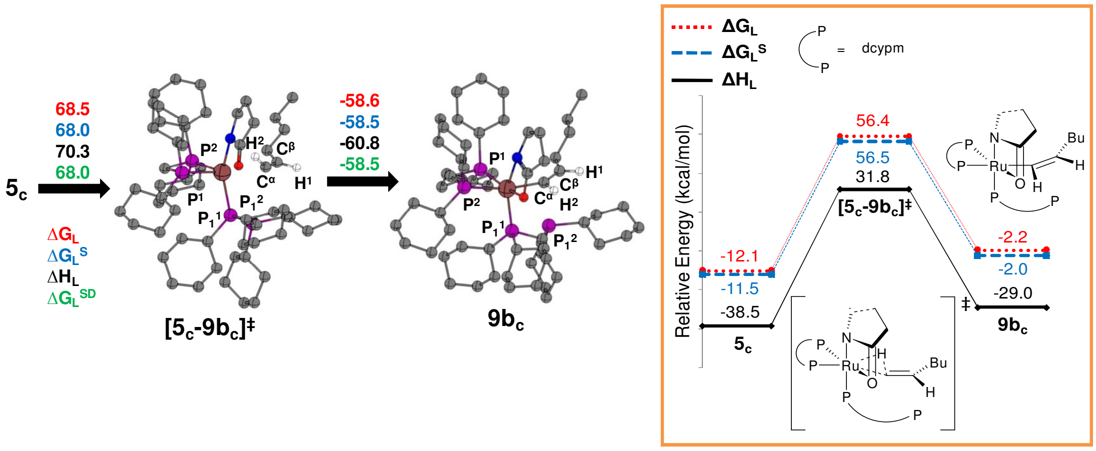

Figure S20. Optimized structures and energy profile (inset) for the vinyl isomerization step. For other conventions refer Figure 1 and Figure S3.

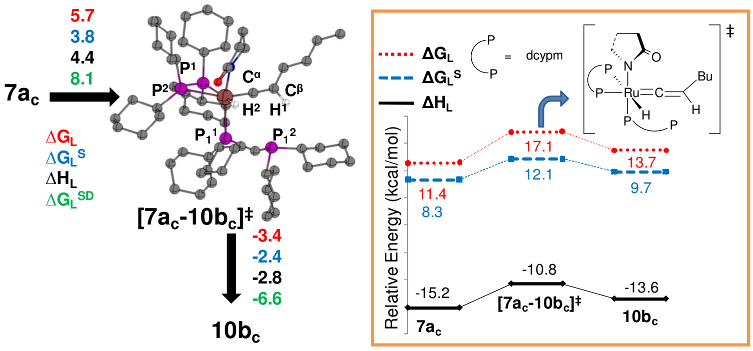

Figure S21. Optimized structures and energy profile (inset) for the vinylidene isomerization step. For other conventions refer to Figure 1 and Figure S3.

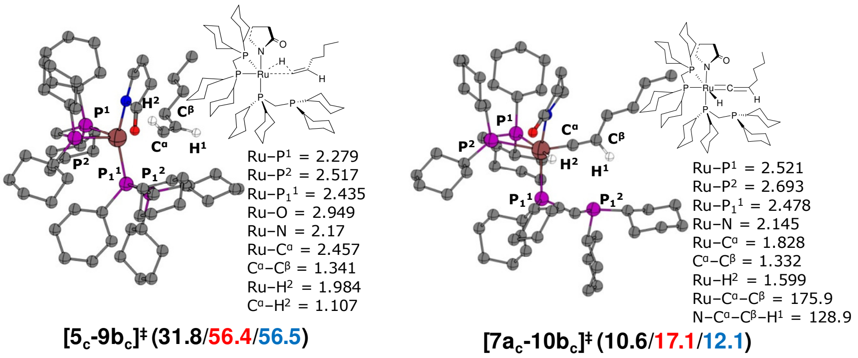

Figure S22. Optimized Structures of transition states involved in isomerization of vinyl (left) and vinylidene (right) by simple bond rotation. For energy and other conventions refer Figure 1 in main text and Figure S3.

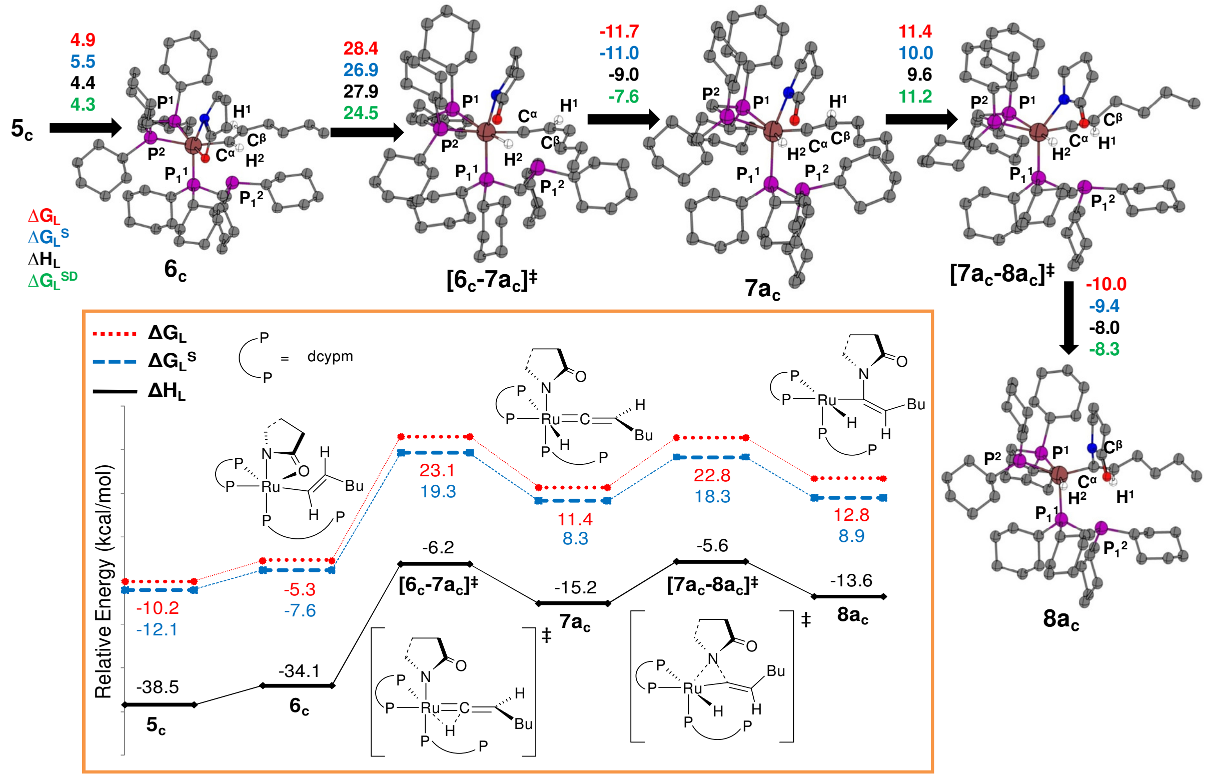

Figure S23. Optimized structures and energy profile (inset) for the vinyl-vinylidene rearrangement and nucleophilic transfer steps in "path a_c". For other conventions refer to Figure 1 and Figure S3.

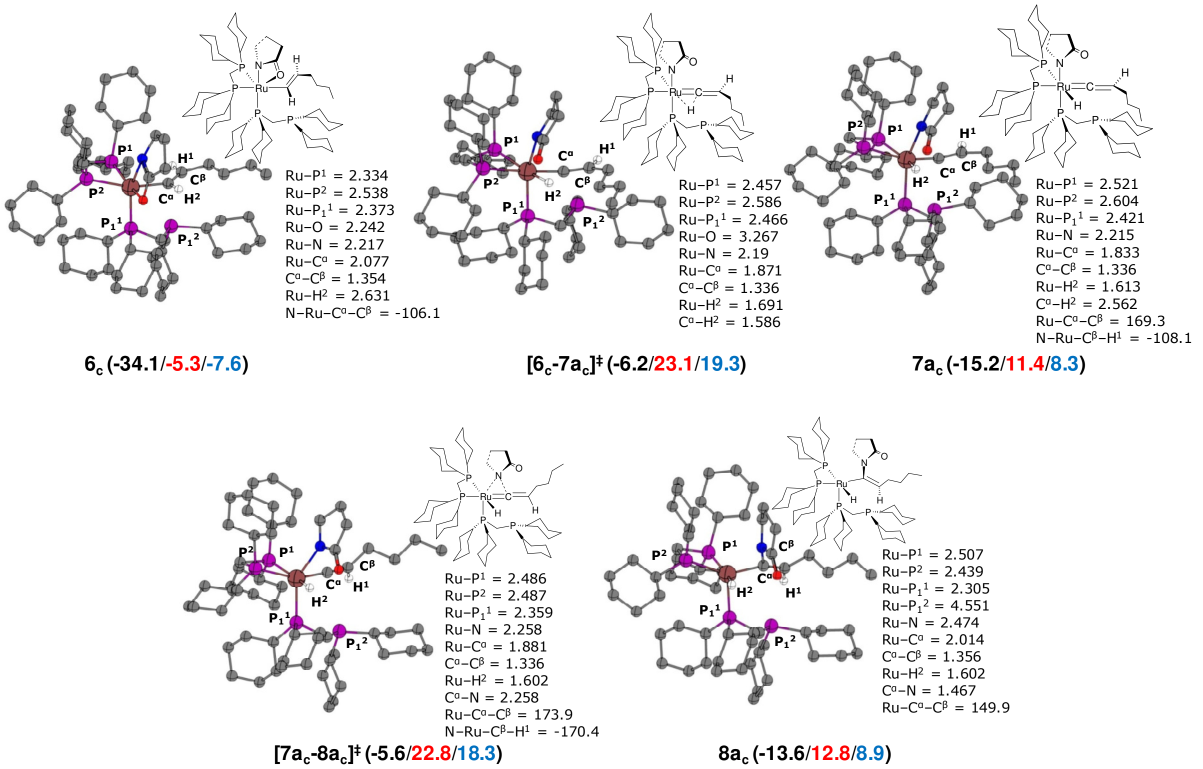

Figure S24. Optimized Structures of intermediates and transition states involved in vinyl-vinylidene rearrangement and nucleophilic attack steps for catalytic system $\mathbf{1_c}$ in "path a_c". For energy and other conventions refer Figure 1 in main text and Figure S3.

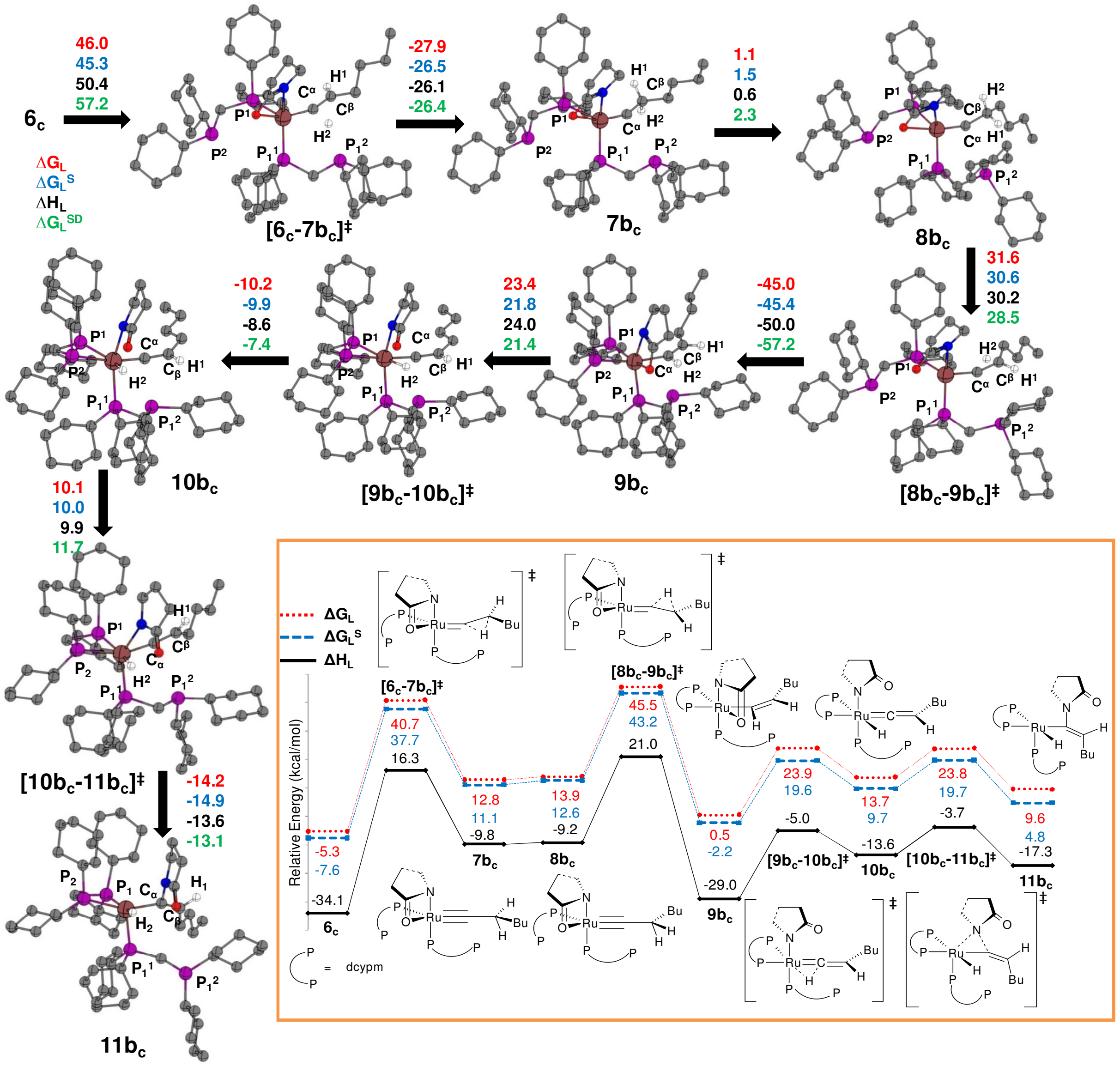

Figure S25. Optimized structures and energy profile (inset) for the vinyl isomerization, vinyl-vinylidene rearrangement and nucleophilic transfer steps in "path b_c". For other conventions refer to Figure 1 and Figure S3.

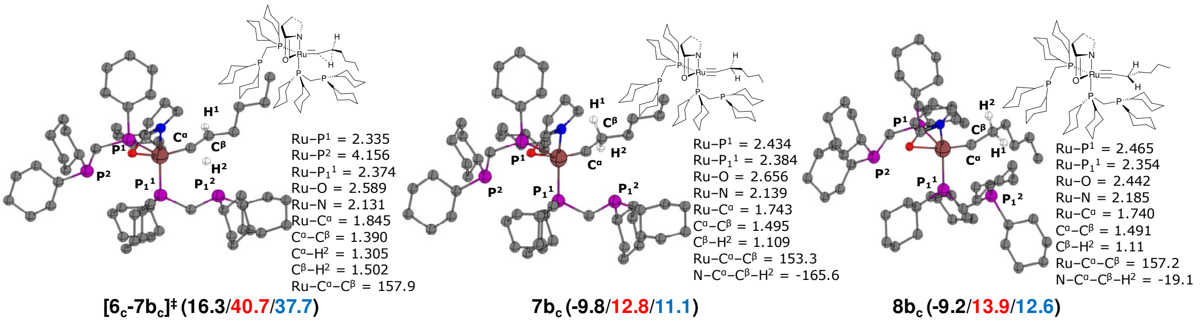

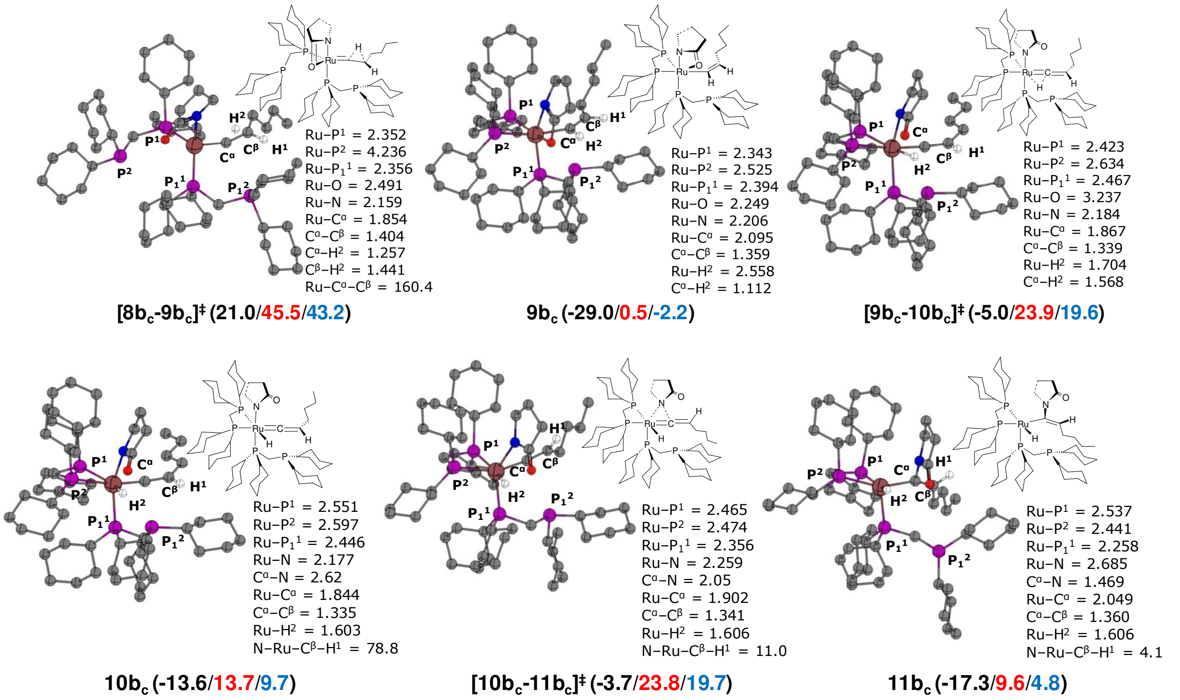

Figure S26. Optimized Structures of intermediates and transition states involved in vinyl-vinylidene rearrangement and nucleophilic attack steps for catalytic system $\mathbf{1_c}$ in "path b_c". For energy and other conventions refer Figure 1 in main text and Figure S3.

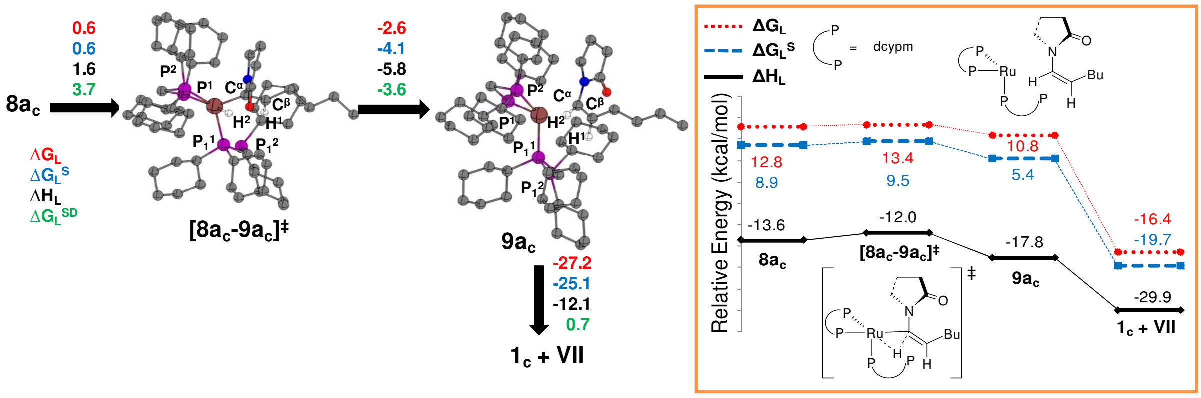

Figure S27. Optimized structures and energy profile (inset) for the reductive elimination step in "path a_c". For other conventions refer to Figure 1 and Figure S3.

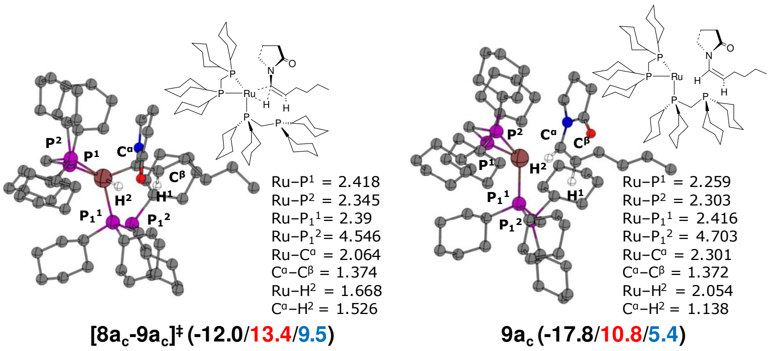

Figure S28. Optimized Structures of intermediates and transition states involved in reductive elimination step for catalyst system$\mathbf{1_c}$ in "path a_c". For energy and other conventions refer Figure 1 in main text and Figure S3.

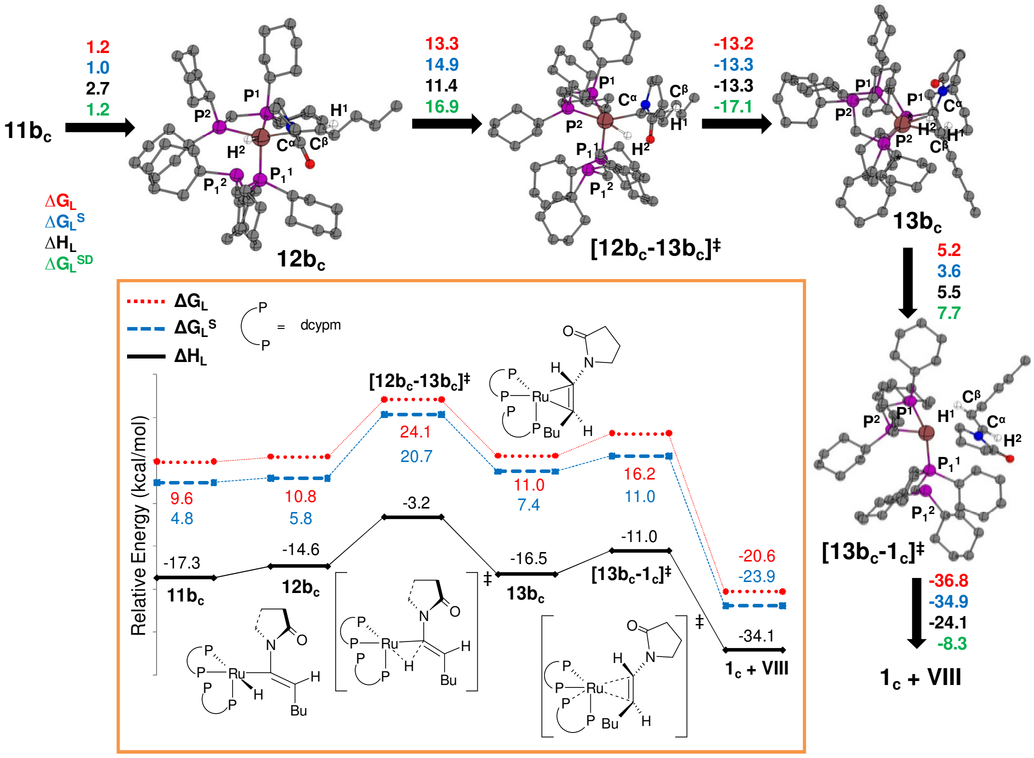

Figure S29. Optimized structures and energy profile (inset) for reductive elimination step in "path b$_c$". For other conventions refer to Figure 1 and Figure S3.

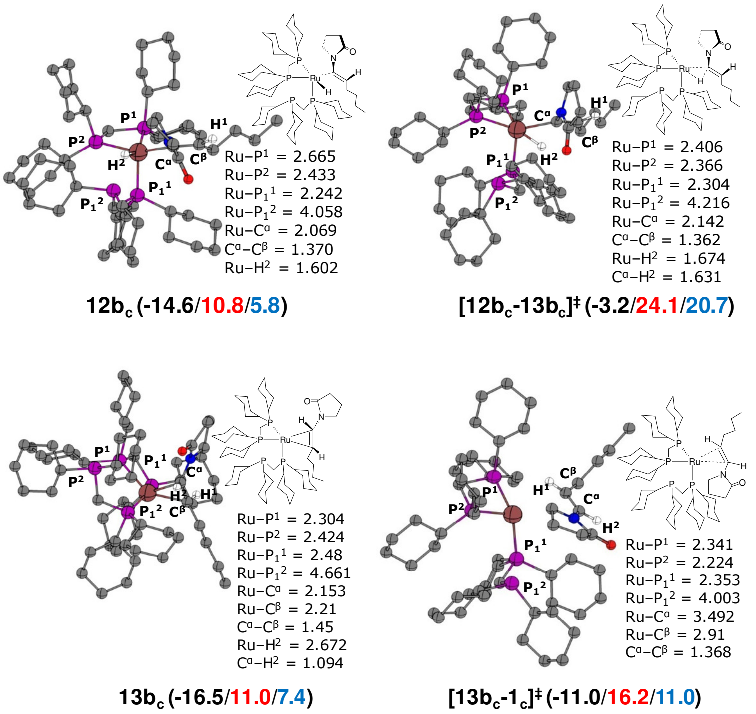

Figure S30. Optimized Structures of intermediates and transition states involved in reductive elimination step for catalyst system **1$_c$** in "path b$_c$". For energy and other conventions refer Figure 1 in main text and Figure S3.

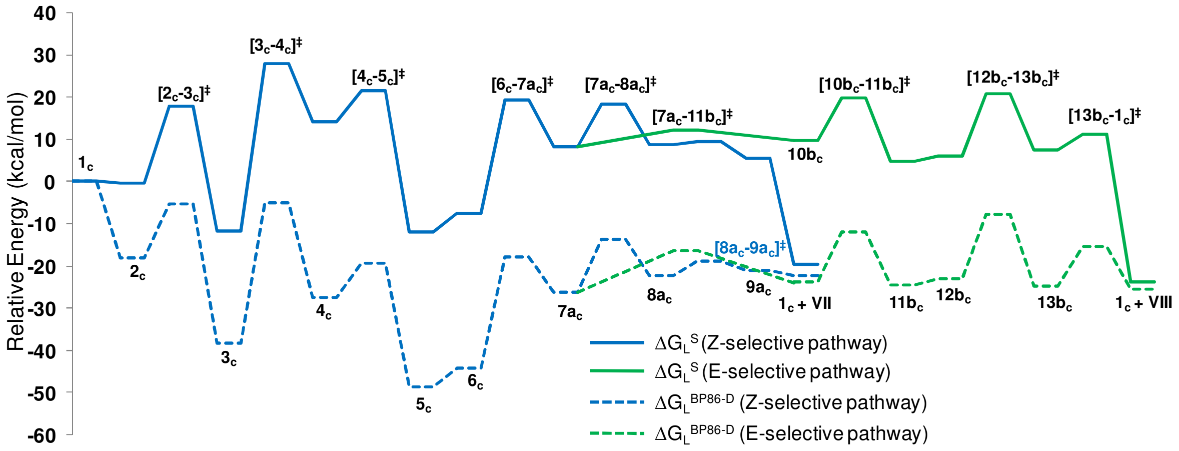

Figure S31. Comparison of the dispersion corrected energy profile (BP86-D/LANL2TZ(f)(Ru)/TZVP(H, C, N, O and P)//ONIOM{BP86/LANL2DZ(Ru)/6-31G*(H, C, N, O and P):HF/STO-3G}; dotted line) with the one reported in the text (BP86/LANL2TZ(f)(Ru)/TZVP(H, C, N, O and P)//ONIOM{BP86/LANL2DZ(Ru)/6-31G*(H, C, N, O and P):HF/STO-3G}; dark line) for favorable pathways of *E*- and *Z*- enamide formation in catalyst system $\mathbf{1_c}$. For energy terms refer to the computational details.

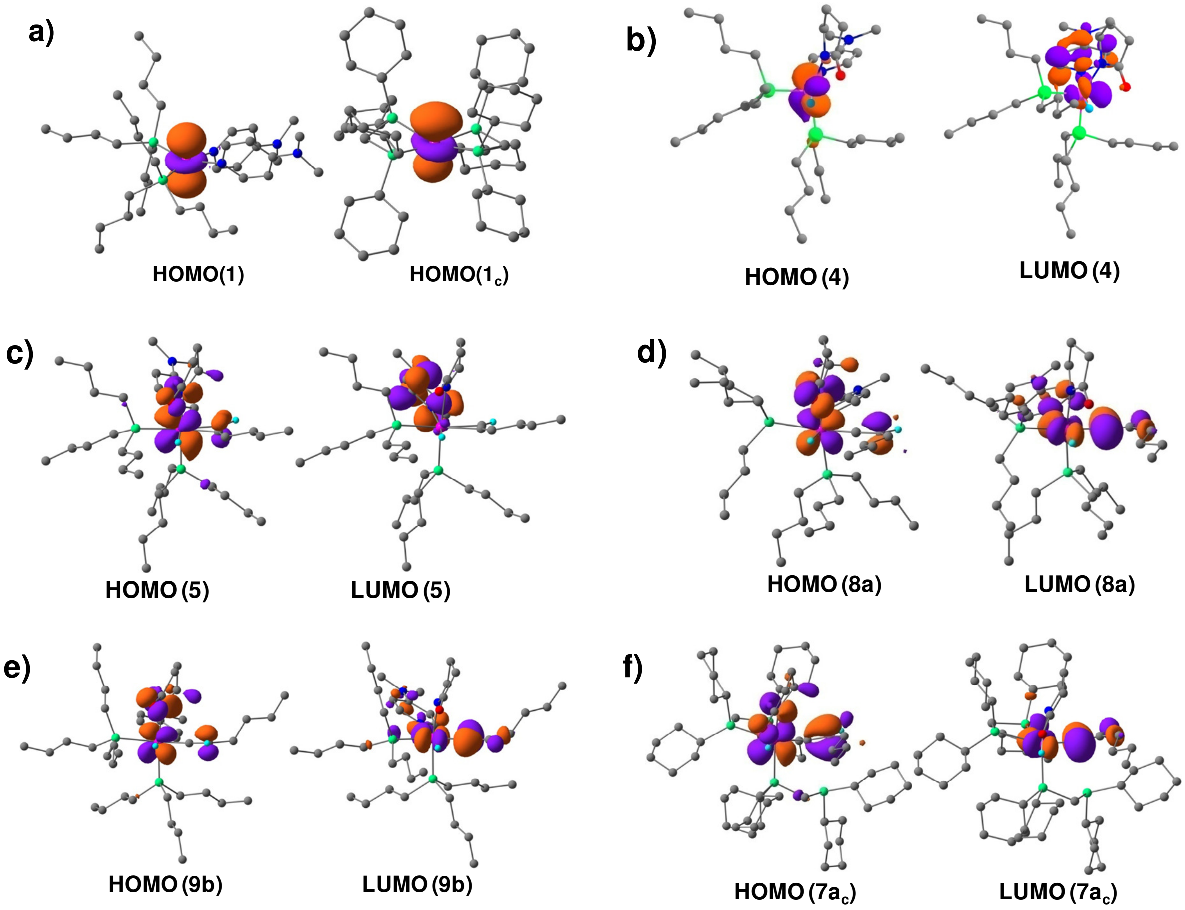

g)

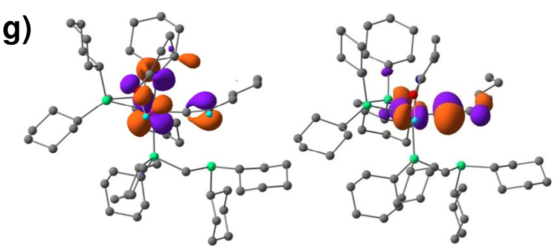

Figure S32. KS-MO (isosurface=0.048 au) of some important intermediates included in parentheses.

Scheme S1. Chemical structures of several modified vinylidene complexes from **7a$_c$** and their respective enamide products.

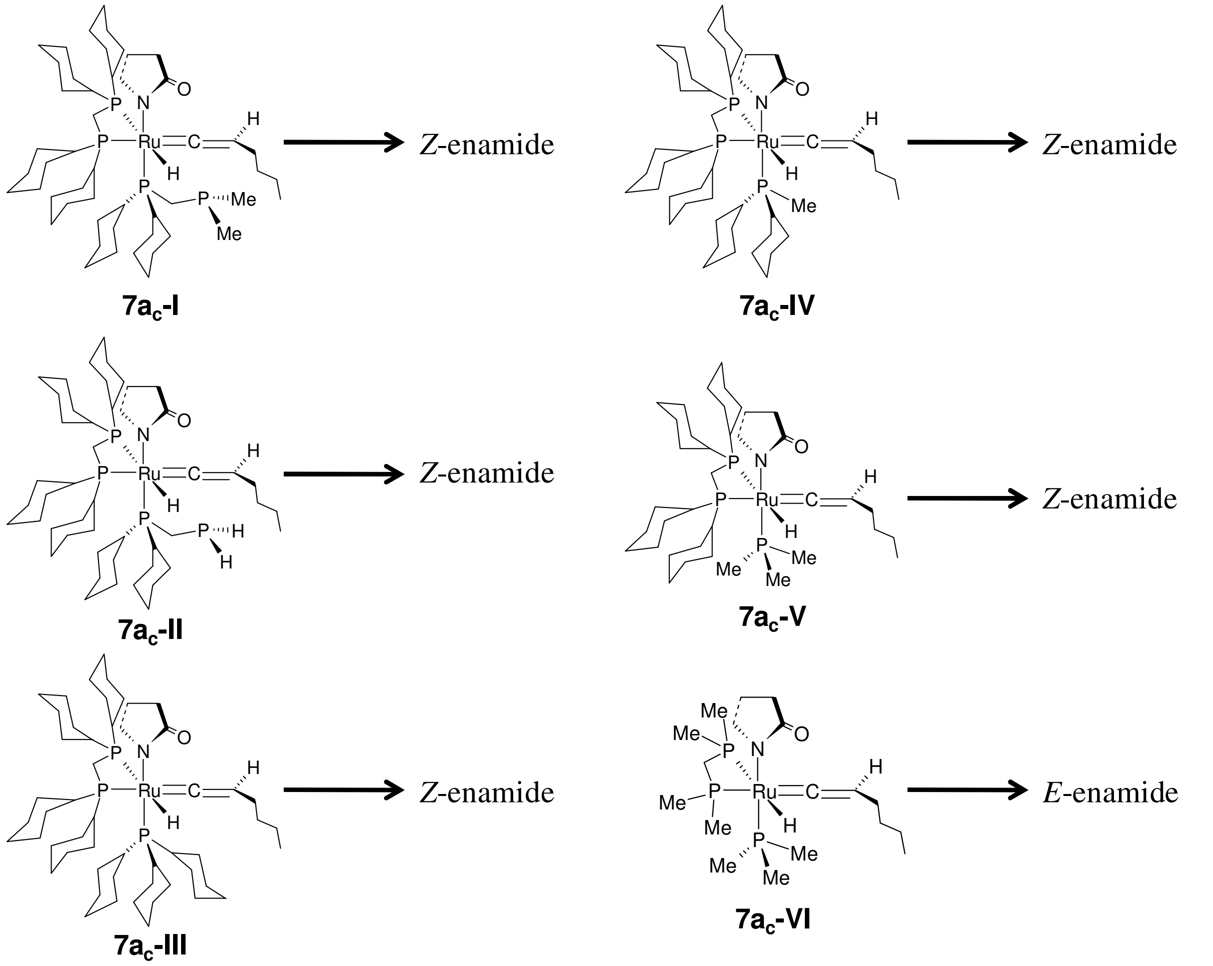

Table S1. Absolute energies (hartree) of all intermediates and transition states involve in catalyst system **1**. E_e = Electronic energy, E_0 = Total energy plus zero-point energy, H_{298} = enthalpy at 298 K, G_{298} = Gibbs free energy and E_{Sol} = Electronic energy at SMD solvation model. First four energy terms (E_e, E_0, H_{298}, G_{298}) are from BP86/LANL2DZ(Ru)/6-31G*(H, C, N, O and P) level and last one (E_{Sol})at BP86/LANL2TZ(f)(Ru)/TZVP(H, C, N, O and P)//BP86/LANL2DZ(Ru)/6-31G*(H, C, N, O and P) level.

No.	E_e	E_0	H_{298}	G_{298}	E_{Sol}

**1**	-2488.313264	-2487.271189	-2487.207193	-2487.38213	-2488.98055
**2**	-2774.96175	-2773.809459	-2773.738828	-2773.926977	-2775.732077
**[2-3]‡**	-2774.950564	-2773.801376	-2773.731339	-2773.917908	-2775.725511
**3**	-2774.979057	-2773.827759	-2773.757754	-2773.943084	-2775.75393
**4**	-2392.714841	-2391.723489	-2391.6636	-2391.826144	-2393.362564
**[4-5]‡**	-2627.291357	-2626.159772	-2626.091394	-2626.270688	-2628.020645
**5**	-2627.313511	-2626.182499	-2626.113696	-2626.295292	-2628.0438
**[5-6]‡**	-2627.306289	-2626.175755	-2626.107612	-2626.287522	-2628.036366
**6**	-2627.352095	-2626.216344	-2626.148467	-2626.327615	-2628.072693
**7a**	-3009.592628	-3008.297337	-3008.21916	-3008.42065	-3010.447767
**[7a-8a]‡**	-3009.573560	-3008.284994	-3008.205753	-3008.414181	-3010.433151
**8a**	-2627.319819	-2626.188928	-2626.120085	-2626.30408	-2628.049852
**[8a-10]‡**	-2627.307976	-2626.177472	-2626.109045	-2626.293039	-2628.034909
**7b**	-3009.599754	-3008.302396	-3008.224804	-3008.423646	-3010.454084
**[7b-8b]‡**	-3009.540835	-3008.251555	-3008.171855	-3008.385136	-3010.401829
**8b**	-2627.350255	-2626.213922	-2626.146399	-2626.323158	-2628.070026
**[8b-9b]‡**	-2627.320819	-2626.190983	-2626.122679	-2626.304294	-2628.05175
**9b**	-2627.329538	-2626.197026	-2626.128826	-2626.308919	-2628.058792
**[9b-10]‡**	-2627.311048	-2626.18019	-2626.112105	-2626.293171	-2628.035810
**10**	-2627.332578	-2626.198404	-2626.130731	-2626.30959	-2628.054002
**[10-11]‡**	-2627.294064	-2626.163857	-2626.09546	-2626.279028	-2628.016801
**11**	-2627.315082	-2626.17855	-2626.110417	-2626.292765	-2628.034666
**[5-6m]‡**	-2627.285381	-2626.154859	-2626.087129	-2626.266143	-2628.009726

**6m**	-2627.339176	-2626.205018	-2626.137422	-2626.318186	-2628.065264
**7m**	-3009.595182	-3008.301101	-3008.223038	-3008.427283	-3010.450022
**[7m-1]‡**	-3009.552721	-3008.259968	-3008.181840	-3008.386229	-3010.407418
**8az**	-2627.341588	-2626.206708	-2626.139151	-2626.319405	-2628.061276
**[8az-10z]‡**	-2627.292277	-2626.161682	-2626.093304	-2626.275981	-2628.033412
**10z**	-2627.346742	-2626.212283	-2626.144581	-2626.323921	-2628.067249
**[10z-11z]‡**	-2627.299419	-2626.168427	-2626.100228	-2626.282902	-2628.022619
**11z**	-2627.310154	-2626.174212	-2626.105609	-2626.290055	-2628.03288
**I**	-234.5775755	-234.439683	-234.430826	-234.471449	-234.669748
**II**	-286.6289429	-286.520721	-286.514213	-286.549702	-286.745095
**III**	-814.8988526	-814.537956	-814.51755	-814.588402	-815.096793
**IV**	-382.249473	-382.091809	-382.081941	-382.126034	-382.387185
**V**	-1663.093456	-1662.437293	-1662.406391	-1662.499608	-1663.479558
**VI**	-521.2666051	-521.015390	-521.001173	-521.056177	-521.459973
**VII**	-521.2704073	-521.018521	-521.004288	-521.060047	-521.464625
**VIII**	-521.2762643	-521.024904	-521.010508	-521.066288	-521.471002

Table S2. Absolute energies (hartree) of all intermediates and transition states involve in catalyst system $\mathbf{1_c}$. E_e = Electronic energy, E_0 = Total energy plus zero-point energy, H_{298} = enthalpy at 298 K, G_{298} = Gibbs free energy at 298 K and E_{Sol} = Electronic energy at SMD salvation model. First four energy terms (E_e, E_0, H_{298}, G_{298}) are from ONIOM{BP86/LANL2DZ(Ru)/6-31G*(H, C, N, O and P):HF/STO-3G}and last one (E_{Sol}) from BP86/LANL2TZ(f)(Ru)/TZVP(H, C, N, O and P)//ONIOM{BP86/LANL2DZ(Ru)/6-31G*(H, C, N, O and P):HF/STO-3G} level.

No.	$\mathbf{E_e}$	$\mathbf{E_0}$	$\mathbf{H_{298}}$	$\mathbf{G_{298}}$	$\mathbf{E_{Sol}}$
$\mathbf{1_c}$	-3385.601179	-3383.994895	-3383.945114	-3384.086746	-3420.95466
$\mathbf{2_c}$	-3672.252939	-3670.539655	-3670.475673	-3670.641565	-3707.714035
$\mathbf{[2_c\text{-}3_c]^‡}$	-3672.19905	-3670.490082	-3670.426606	-3670.591313	-3707.680759

**3_c**	-3672.24912	-3670.53607	-3670.473281	-3670.634027	-3707.734159
**$[3_c\text{-}4_c]^‡$**	-3906.780791	-3904.928416	-3904.856536	-3905.039789	-3942.354687
**4_c**	-3906.812673	-3904.958689	-3904.887213	-3905.067655	-3942.38057
**$[4_c\text{-}5_c]^‡$**	-3906.796055	-3904.943774	-3904.87264	-3905.052226	-3942.367223
**5_c**	-3906.866123	-3905.008798	-3904.93748	-3905.118407	-3942.423551
**6_c**	-3906.858277	-3905.000789	-3904.929522	-3905.110259	-3942.416889
**$[6_c\text{-}7a_c]^‡$**	-3906.798691	-3904.946205	-3904.875081	-3905.055528	-3942.370077
**$7a_c$**	-3906.819227	-3904.965628	-3904.893865	-3905.07743	-3942.386537
**$[7a_c\text{-}8a_c]_‡$**	-3906.806344	-3904.953625	-3904.882376	-3905.063959	-3942.370976
**$8a_c$**	-3906.828683	-3904.973336	-3904.901902	-3905.085046	-3942.387124
**$[8a_c\text{-}9a_c]^‡$**	-3906.822979	-3904.970649	-3904.899003	-3905.083818	-3942.391014
**$9a_c$**	-3906.82902	-3904.971394	-3904.899529	-3905.084147	-3942.381842
**$[5_c\text{-}9b_c]^‡$**	-3906.756113	-3904.90261	-3904.831011	-3905.014	-3942.310166
**$[6_c\text{-}7b_c]^‡$**	-3906.770944	-3904.9217	-3904.849578	-3905.036048	-3942.3336751
**$7b_c$**	-3906.821847	-3904.966439	-3904.893896	-3905.082693	-3942.37997545
**$8b_c$**	-3906.823375	-3904.967667	-3904.895197	-3905.083426	-3942.37823762
**$[8b_c\text{-}9b_c]^‡$**	-3906.766462	-3904.915077	-3904.842976	-3905.029419	-3942.3267696
**$9b_c$**	-3906.848541	-3904.990592	-3904.919535	-3905.099571	-3942.409294
**$[9b_c\text{-}10b_c]^‡$**	-3906.79549	-3904.943146	-3904.871979	-3905.05288	-3942.369098
**$10b_c$**	-3906.815081	-3904.961195	-3904.889599	-3905.072392	-3942.385015
**$[7a_c\text{-}10b_c]^‡$**	-3906.808365	-3904.955481	-3904.884206	-3905.066466	-3942.38048522
**$[10b_c\text{-}11b_c]^‡$**	-3905.803241	-3904.950684	-3904.879391	-3905.0614	-3942.366983
**$11b_c$**	-3906.835208	-3904.981073	-3904.909381	-3905.0927	-3942.392764

**$12b_c$**	-3906.813375	-3904.959442	-3904.887667	-3905.072286	-3942.38952
**$[12b_c\text{-}13b_c]^{\ddagger}$**	-3906.810709	-3904.958504	-3904.886963	-3905.070043	-3942.365679
**$13b_c$**	-3906.840866	-3904.981986	-3904.910596	-3905.093394	-3942.392962
**$[13b_c\text{-}1_c]^{\ddagger}$**	-3906.834296	-3904.977382	-3904.905967	-3905.08929	-3942.384989

Table S3. Energy changes (in kcal/mol) for all steps involved in catalyst system **1**. For different energy terms refer Computational Details.

Step	ΔE_e	ΔH_{298}	ΔG_{298}	ΔE_L	ΔE_L^S	$\Delta E_L^{BP86\text{-}D}$	ΔE_L^{B97D}	$\Delta E_L^{M06\text{-}2X}$
**1+I→2**	-12.3	-10.9	3.0	-7.9	-4.0	-21.4	-19.8	-20.0
**2→[2-3]‡**	7.0	4.7	5.7	6.3	4.1	3.8	4.2	2.9
**[2-3]‡→3**	-17.9	-16.6	-15.8	-19.5	-17.8	-28.9	-24.0	-31.1
**3→4+IV**	9.3	7.7	-5.7	8.2	2.6	20.9	14.6	19.0
**4+II→[4-5]‡**	0.7	1.9	16.9	4.0	7.3	4.4	—	—
**[4-5]‡→5**	-13.9	-14.0	-15.4	-15.8	-14.5	-24.0	-10.0[a]	-6.3[a]
**5→[5-6]‡**	5.2	3.8	4.9	4.5	4.7	5.4	6.7	5.1
**[5-6]‡→6**	-29.4	-25.6	-23.3	-23.7	-20.3	-18.9	-21.2	-26.5
**6+IV→7a**	5.6	7.1	18.8	5.2	5.1	-21.5	-16.5	-11.8
**7a→[7a-8a]‡**	12.0	8.4	4.1	10.3	9.2	28.6	22.1	28.3
**[7a-8a]‡→8a+IV**	2.7	2.3	-10.0	1.0	-2.4	9.6	8.1	9.1
**8a→[8a-10]‡**	7.4	6.9	6.9	9.4	9.4	12.3	12.4	14.0
**[8a-10]‡→10**	-15.4	-13.6	-10.4	-12.4	-12.0	-14.9	-14.5	-21.6
**6+IV→7b**	1.1	3.5	16.9	1.7	1.1	-25.9	-21.7	-16.2
**7b→[7b-8b]‡**	37.0	32.2	24.2	34.5	32.8	56.2	50.4	66.0
**[7b-8b]‡→8b+IV**	-37.0	-35.4	-40.2	-35.2	-34.8	-33.4	-31.5	-53.4
**8b→[8b-9b]‡**	18.5	14.9	11.8	13.9	11.5	16.6	15.6	25.2
**[8b-9b]‡→9b**	-5.5	-3.9	-2.9	-4.2	-4.4	-2.7	-4.3	-2.2
**9b→[9b-10]‡**	11.6	10.5	9.9	13.7	14.4	11.9	11.7	12.5
**[9b-10]‡→10**	-13.5	-11.7	-10.3	-10.9	-11.4	-8.5	-8.7	-13.8
**10→[10-11]‡**	24.2	22.1	19.2	23.3	23.3	24.3	23.4	28.7
**[10-11]‡→11**	-13.2	-9.4	-8.6	-10.8	-11.2	-7.5	-12.2	-20.4
**11+IV→1+III**	-15.7	-15.9	-18.6	-18.4	-18.6	-13.6	-9.7	-7.5

**5→[5-6m]**‡	17.7	16.7	18.3	20.4	21.4	20.7	22.3	22.5
**[5-6m]**‡**→6m**	-33.8	-31.6	-32.7	-32.1	-34.9	-25.0	-27.3	-30.8
**6m+IV→7m**	-4.1	-2.3	10.6	-2.3	1.5	-19.9	-17.5	-14.2
**7m→[7m-1]**‡	26.6	25.9	25.8	26.6	26.7	27.7	25.8	32.1
**[7m-1]**‡**→1+VII**	-17.0	-18.6	-32.7	-18.1	-20.8	-0.4	-0.9	-8.8
**8a→8az**	-13.7	-12.0	-9.6	-8.7	-7.2	-5.7	-1.2	-9.2
**8az→[8az-10z]**‡	30.9	28.8	27.2	28.2	27.5	28.0	13.5	22.1
**[8az-10z]**‡**→10z**	-34.2	-32.2	-30.1	-30.7	-31.3	-31.5	-21.6	-29.0
**10z→[10z-11z]**‡	29.7	27.8	25.7	27.4	28.0	28.2	29.4	32.3
**[10z-11z]**‡**→11z**	-6.7	-3.4	-4.5	-4.5	-6.4	-4.0	-3.4	-6.9
**11z+IV→1+VI**	-15.1	-15.0	-16.4	-16.7	-15.8	-19.4	-14.2	-12.6

[a] for the step **4** + **II** → **5**.

Table S4. Energy changes at higher basis (in kcal/mol) for all steps involved in catalyst system **1**. For different energy terms refer Computational Details.

Step	ΔH_L	ΔG_L	ΔG_L^S	ΔG_L^{SD}	Step	ΔH_L	ΔG_L	ΔG_L^S	ΔG_L^{SD}
**1+I→2**	-6.8	7.8	6.8	-6.7	**[8b-9b]**‡**→9b**	-3.0	-3.6	-3.6	-2.2
**2→[2-3]**‡	4.2	6.0	3.2	0.7	**9b→[9b-10]**‡	12.8	12.1	14.3	12.5
**[2-3]**‡**→3**	-18.0	-16.9	-15.6	-25.0	**[9b-10]**‡**→10**	-9.7	-11.8	-11.6	-9.2
**3→4+IV**	6.0	-13.1	-12.3	13.0	**10→[10-11]**‡	21.9	22.0	22.0	23.0
**4+II→[4-5]**‡	5.0	21.4	19.3	7.2	**[10-11]**‡**→11**	-8.6	-7.0	-7.9	-4.6
**[4-5]**‡**→5**	-15.2	-19.5	-16.8	-25.0	**11+IV→1+III**	-17.4	-20.0	-19.3	-14.5
**5→[5-6]**‡	3.3	11.0	7.9	8.8	**5→[5-6m]**‡	19.4	26.9	25.4	25.8
**[5-6]**‡**→6**	-20.9	-22.0	-18.2	-13.4	**[5-6m]**‡**→6m**	-30.6	-34.3	-35.8	-28.7
**6+IV→7a**	7.3	25.1	19.1	-7.6	**6m+IV→7m**	-0.2	16.8	14.9	-2.6
**7a→[7a-8a]**‡	7.1	1.7	2.4	20.7	**7m→[7m-1]**‡	26.2	26.7	26.6	37.1
**[7a-8a]**‡**→8a+IV**	0.8	-13.0	-11.9	-3.4	**[7m-1]**‡**→1+VII**	-18.0	-35.8	-32.5	-20.4
**8a→[8a-10]**‡	8.4	7.9	8.1	11.0	**8a→8az**	-8.2	-7.0	-6.5	-2.9
**[8a-10]**‡**→10**	-11.2	-11.0	-10.7	-13.1	**8az→[8az-10z]**‡	26.8	26.0	26.2	25.4
**6+IV→7b**	4.1	21.1	14.9	-12.7	**[8az-10z]**‡**→10z**	-29.3	-29.6	-30.1	-30.9
**7b→[7b-8b]**‡	31.7	23.5	24.5	46.2	**10z→[10z-11z]**‡	26.0	26.5	27.0	27.8
**[7b-8b]**‡**→8b+IV**	-34.6	-41.6	-38.8	-37.1	**[10z-11z]**‡**→11z**	-2.7	-4.7	-5.9	-3.7
**8b→[8b-9b]**‡	11.1	9.1	7.3	10.1	**11z+IV→1+VI**	-15.3	-15.1	-14.3	-10.7

Table S5. Energy changes (in kcal/mol) for all steps involved in catalyst system $\mathbf{1_c}$. For different energy terms refer Computational Details.

Step	ΔE_e	ΔH_{298}	ΔG_{298}	ΔE_L	ΔE_L^S	ΔE_L^{BP86D}	ΔE_L^{B97D}	$\Delta E_L^{M06\text{-}2X}$
$\mathbf{1_c+I \rightarrow 2_c}$	-14.3	-10.3	-3.2	-13.3	-9.0	-31.2	-30.3	-23.7
$\mathbf{2_c \rightarrow [2_c\text{-}3_c]^{\ddagger}}$	33.8	30.8	31.5	21.7	20.9	16.0	20.8	20.9
$\mathbf{[2_c\text{-}3_c]^{\ddagger} \rightarrow 3_c}$	-31.4	-29.3	-26.8	-34.2	-33.5	-38.3	-37.2	-38.8
$\mathbf{3_c+II \rightarrow [3_c\text{-}4_c]^{\ddagger}}$	28.8	29.9	41.2	31.1	30.9	20.9	17.0	14.1
$\mathbf{[3_c\text{-}4_c]^{\ddagger} \rightarrow 4_c}$	-20.0	-19.3	-17.5	-18.8	-16.2	-25.7	-17.6	-10.8
$\mathbf{4_c \rightarrow [4_c\text{-}5_c]^{\ddagger}}$	10.4	9.1	9.7	8.4	8.4	8.8	9.8	6.5
$\mathbf{[4_c\text{-}5_c]^{\ddagger} \rightarrow 5_c}$	-44.0	-40.7	-41.5	-36.4	-35.3	-30.6	-33.8	-42.0
$\mathbf{5_c \rightarrow 6_c}$	4.9	5.0	5.1	4.3	4.2	4.1	4.0	4.5
$\mathbf{6_c \rightarrow [6_c\text{-}7a_c]^{\ddagger}}$	37.4	34.2	34.3	30.8	29.4	28.5	28.9	39.7
$\mathbf{[6_c\text{-}7a_c]^{\ddagger} \rightarrow 7a_c}$	-12.9	-11.8	-13.7	-10.1	-10.3	-6.7	-9.1	-3.5
$\mathbf{7a_c \rightarrow [7a_c\text{-}8a_c]^{\ddagger}}$	8.1	7.2	8.5	10.5	9.8	11.6	12.3	10.9
$\mathbf{[7a_c\text{-}8a_c]^{\ddagger} \rightarrow 8a_c}$	-14.0	-12.3	-13.2	-9.7	-10.1	-8.2	-8.3	-14.9
$\mathbf{8a_c \rightarrow [8a_c\text{-}9a_c]^{\ddagger}}$	3.6	1.8	0.8	3.8	3.8	6.5	5.6	5.8
$\mathbf{[8a_c\text{-}9a_c]^{\ddagger} \rightarrow 9a_c}$	-3.8	-0.3	-0.2	-6.3	-6.8	-5.9	-7.5	-6.6
$\mathbf{9a_c \rightarrow 1_c+VI}$	-26.7	-31.3	-39.3	-14.3	-23.5	11.5	11.5	3.3
$\mathbf{5_c \rightarrow [5_c\text{-}9b_c]^{\ddagger}}$	69.0	66.8	65.5	72.3	71.2	71.0	69.9	87.3
$\mathbf{[5_c\text{-}9b_c]^{\ddagger} \rightarrow 9b_c}$	-58.0	-55.5	-53.7	-63.0	-62.2	-62.9	-61.4	-78.2
$\mathbf{6_c \rightarrow [6_c\text{-}7b_c]^{\ddagger}}$	54.8	50.2	46.6	54.4	52.2	66.4	63.9	74.6
$\mathbf{[6_c\text{-}7b_c]^{\ddagger} \rightarrow 7b_c}$	-31.9	-27.8	-29.3	-29.8	-29.1	-29.5	-32.6	-34.7
$\mathbf{7b_c \rightarrow 8b_c}$	-1.0	-0.8	-0.5	0.5	1.1	1.2	1.2	0.2
$\mathbf{8b_c \rightarrow [8b_c\text{-}9b_c]^{\ddagger}}$	35.7	32.8	33.9	32.9	32.3	30.8	33.6	34.3
$\mathbf{[8b_c\text{-}9b_c]^{\ddagger} \rightarrow 9b_c}$	-51.5	-48.0	-44.0	-53.1	-51.8	-64.8	-61.5	69.6
$\mathbf{9b_c \rightarrow [9b_c\text{-}10b_c]^{\ddagger}}$	33.3	29.8	29.3	27.1	25.2	26.7	25.8	36.3
$\mathbf{[9b_c\text{-}10b_c]^{\ddagger} \rightarrow 10b_c}$	-12.3	-11.1	-12.2	-9.8	-10.0	-7.3	-8.6	-3.8
$\mathbf{7a_c \rightarrow [7a_c\text{-}10b_c]^{\ddagger}}$	6.8	6.1	6.9	5.2	3.8	9.5	8.9	6.6
$\mathbf{[7a_c\text{-}10b_c]^{\ddagger} \rightarrow 10b_c}$	-4.2	-3.4	-3.7	-3.7	-2.8	-7.9	-7.1	-5.6
$\mathbf{10b_c \rightarrow [10b_c \rightarrow 11b_c]^{\ddagger}}$	7.4	6.4	6.9	11.4	11.3	13.7	15.0	14.0
$\mathbf{[10b_c\text{-}11b_c]^{\ddagger} \rightarrow 11b_c}$	-20.1	-18.8	-19.6	-15.3	-16.2	-12.8	-14.7	-22.9
$\mathbf{11b_c \rightarrow 12b_c}$	13.7	13.6	12.8	2.7	2.0	1.6	-0.1	-0.5
$\mathbf{12b_c \rightarrow [12b_c\text{-}13b_c]^{\ddagger}}$	1.7	0.4	1.4	12.8	15.0	12.9	15.5	17.8

**[12b$_c$-13b$_c$]‡→13b$_c$**	-18.9	-14.8	-14.7	-17.0	-17.1	-18.9	-19.2	-18.1
**13b$_c$→[13b$_c$-1$_c$]‡**	4.1	2.9	2.6	6.7	5.0	15.8	13.0	4.4
**[13b$_c$-1$_c$]‡→1$_c$+III**	-27.1	-31.2	-40.0	-22.8	-25.5	-1.2	-0.5	-0.6

Table S6. Energy changes at higher basis (in kcal/mol) for all steps involved in catalyst system **1$_c$**. For different energy terms refer Computational Details.

Step	ΔH_L	ΔG_L	ΔG_L^S	ΔG_L^{SD}	Step	ΔH_L	ΔG_L	ΔG_L^S	ΔG_L^{SD}
**1$_c$+I→2$_c$**	-13.6	-0.3	-0.4	-18.4	**6$_c$→[6$_c$-7b$_c$]‡**	50.4	46.0	45.3	57.2
**2$_c$→[2$_c$-3$_c$]‡**	19.7	18.7	18.1	12.4	**[6$_c$-7b$_c$]‡→7b$_c$**	-26.1	-27.9	-26.5	-26.4
**[2$_c$-3$_c$]‡→3$_c$**	-32.3	-29.1	-29.5	-33.6	**7b$_c$→8b$_c$**	0.6	1.1	1.5	2.3
**3$_c$+II→[3$_c$-4$_c$]‡**	32.2	43.6	39.6	29.4	**8b$_c$→[8b$_c$-9b$_c$]‡**	30.2	31.6	30.6	28.5
**[3$_c$-4$_c$]‡→4$_c$**	-18.0	-15.6	-13.8	-20.7	**[8b$_c$-9b$_c$]‡→9b$_c$**	-50.0	-45.0	-45.4	-57.2
**4$_c$→[4$_c$-5$_c$]‡**	7.1	7.6	7.4	7.9	**9b$_c$→[9b$_c$-10b$_c$]‡**	24.0	23.4	21.8	21.4
**[4$_c$-5$_c$]‡→5$_c$**	-33.6	-35.1	-33.5	-27.7	**[9b$_c$-10b$_c$]‡→10b$_c$**	-8.6	-10.2	-9.9	-7.4
**5$_c$→6$_c$**	4.4	4.9	5.5	4.3	**7a$_c$→[7a$_c$-10b$_c$]‡**	4.4	5.7	3.8	8.1
**6$_c$→[6$_c$-7a$_c$]‡**	27.9	28.4	26.9	24.6	**[7a$_c$-10b$_c$]‡→10b$_c$**	-2.8	-3.4	-2.4	-6.6
**[6$_c$-7a$_c$]‡→7a$_c$**	-9.0	-11.7	-11.0	-7.6	**10b$_c$→[10b$_c$-11b$_c$]‡**	9.9	10.1	10.0	11.7
**7a$_c$→[7a$_c$-8a$_c$]‡**	9.6	11.4	10.0	11.1	**[10b$_c$-11b$_c$]‡→11b$_c$**	-13.6	-14.2	-14.9	-13.1
**[7a$_c$-8a$_c$]‡→8a$_c$**	-8.0	-10.0	-9.4	-8.3	**11b$_c$→12b$_c$**	2.7	1.2	1.0	1.2
**8a$_c$→[8a$_c$-9a$_c$]‡**	1.6	0.6	0.6	3.7	**12b$_c$→[12b$_c$-13b$_c$]‡**	11.4	13.3	14.9	16.9
**[8a$_c$-9a$_c$]‡→9a$_c$**	-5.8	-2.6	-4.0	-3.6	**[12b$_c$-13b$_c$]‡→13b$_c$**	-13.3	-13.2	-13.3	-17.1
**9a$_c$→1$_c$+VI**	-12.1	-27.2	-25.1	0.7	**13b$_c$→[13b$_c$-1$_c$]‡**	5.5	5.2	3.6	7.7
**5$_c$→[5$_c$-9b$_c$]‡**	70.3	69.2	68.5	68.0	**[13b$_c$-1$_c$]‡→1$_c$+III**	-24.1	-36.8	-34.9	-8.3
**[5$_c$-9b$_c$]‡→9b$_c$**	-60.8	-58.6	-58.6	-58.5					

Table S7. NPA charges of selected atoms for all intermediates and transition states involved in the reaction pathways for catalyst **1**.

No.	**Ru**	**N^1**	**N^2**	**N^3**	**O**	**P^1**	**P^2**	**C$^{\alpha}$**	**C$^{\beta}$**	**H^1**	**H^2**
**1**	-0.570	-0.422	-0.422	—	—	1.071	1.067	—	—	—	—
**2**	-0.656	-0.416	-0.427	-0.584	-0.615	1.073	1.083	—	—	0.433	—
**[2-3]‡**	-0.351	-0.499	-0.434	-0.622	-0.709	1.105	1.225	—	—	0.115	—

**3**	-0.323	-0.441	-0.406	-0.558	-0.661	1.155	1.105	–	–	0.017	–
**4**	-0.284	-0.460	–	-0.606	-0.613	1.268	1.092	–	–	0.005	–
**[4-5]‡**	-0.273	-0.456	–	-0.563	-0.676	1.276	1.083	-0.332	0.062	-0.013	0.233
**5**	-0.323	-0.436	–	-0.532	-0.680	1.116	1.146	-0.378	0.063	0.094	0.224
**[5-6]‡**	-0.273	-0.408	-0.442	-0.515	-0.698	1.057	1.130	-0.383	0.032	0.181	0.219
**6**	-0.021	-0.338	–	-0.516	-0.590	0.993	1.165	-0.348	-0.304	0.195	0.156
**7a**	-0.035	-0.372	-0.364	-0.543	-0.659	0.949	1.127	-0.361	-0.311	0.189	0.154
**[7a-8a]‡**	-0.203	-0.404	-0.449	-0.527	-0.697	0.990	1.155	-0.044	-0.421	0.228	0.151
**8a**	-0.238	-0.447	–	-0.543	-0.656	0.968	1.147	0.193	-0.501	0.236	0.070
**[8a-10]‡**	-0.349	-0.444	–	-0.520	-0.589	0.982	1.162	0.230	-0.503	0.230	0.044
**7b**	-0.125	-0.407	-0.439	-0.570	-0.652	1.181	1.120	-0.300	-0.304	0.202	0.143
**[7b-8b]‡**	-0.082	-0.442	-0.466	-0.520	-0.677	1.059	1.059	-0.322	-0.262	0.229	0.230
**8b**	-0.027	-0.346	–	-0.511	-0.594	0.972	1.160	-0.338	-0.306	0.177	0.172
**[8b-9b]‡**	-0.201	-0.405	–	-0.534	-0.668	1.015	1.135	-0.073	-0.435	0.227	0.185
**9b**	-0.234	-0.441	–	-0.536	-0.660	0.996	1.136	0.151	-0.505	0.242	0.089
**[9b-10]‡**	-0.348	-0.447	–	-0.511	-0.592	0.991	1.163	0.198	-0.492	0.226	0.055
**10**	-0.374	-0.450	–	-0.465	-0.534	0.954	1.222	0.058	-0.339	0.182	-0.004
**[10-11]‡**	-0.328	-0.487	–	-0.423	-0.571	0.999	1.170	-0.146	-0.258	0.189	0.201
**11**	-0.683	-0.437	–	-0.373	0.564	1.169	1.040	-0.044	-0.250	0.197	0.253
**[5-6m]‡**	-0.379	-0.447	–	-0.473	-0.588	1.093	1.188	-0.559	0.152	0.042	0.215
**6m**	-1.172	-0.381	–	-0.391	-0.573	1.127	1.387	-0.315	0.071	0.98	0.180
**7m**	-0.392	-0.437	-0.401	-0.402	-0.598	1.023	1.202	-0.344	0.028	-0.025	0.156
**[7m-1]‡**	-0.932	-0.385	-0.366	-0.417	-0.570	1.118	1.193	-0.403	0.115	0.204	0.229
**8az**	-0.415	-0.419	–	-0.442	-0.456	1.018	1.163	0.140	-0.367	0.199	-0.029
**[8az-10z]‡**	-0.328	-0.445	–	-0.530	-0.625	0.974	1.175	0.247	-0.484	0.248	0.015

**10z**	-0.353	-0.453	—	-0.426	-0.629	0.980	1.303	0.014	-0.288	0.192	-0.080
**[10z-11z]**‡	-0.419	-0.481	—	-0.412	-0.600	1.001	1.155	-0.121	-0.220	0.194	0.192
**11z**	-0.576	-0.454	—	-0.403	-0.581	1.157	1.028	-0.070	-0.184	0.201	0.226

Table S8. NPA charges of atoms particularly involve on reaction coordinates for all intermediates and transition states involve in the reaction pathway of catalyst $\mathbf{1_c}$.

No	**Ru**	**P^1**	**P^2**	**$P_1^{\,1}$**	**$P_1^{\,2}$**	**N**	**O**	**C^{α}**	**C^{β}**	**H^1**	**H^2**
$\mathbf{1_c}$	-0.903	0.973	1.009	0.973	1.009	—	—	—	—	—	—
$\mathbf{2_c}$	-1.056	1.004	1.034	1.013	0.999	-0.584	-0.615	—	—	0.442	—
$[\mathbf{2_c}\text{-}\mathbf{3_c}]^{\ddagger}$	-0.690	0.868	1.073	1.036	0.999	-0.612	-0.610	—	—	0.289	—
$\mathbf{3_c}$	-0.638	0.890	1.130	1.051	1.059	-0.535	-0.655	—	—	0.066	—
$[\mathbf{3_c}\text{-}\mathbf{4_c}]^{\ddagger}$	-0.341	0.855	1.276	0.970	0.747	-0.550	-0.658	-0.320	0.036	0.022	0.259
$\mathbf{4_c}$	-0.419	0.946	1.112	1.072	0.753	-0.521	-0.658	-0.346	0.022	0.102	0.233
$[\mathbf{4_c}\text{-}\mathbf{5_c}]^{\ddagger}$	-0.404	1.030	1.030	1.059	0.754	-0.513	-0.665	-0.340	-0.075	0.189	0.227
$\mathbf{5_c}$	-0.234	1.163	0.940	1.106	0.736	-0.502	-0.616	-0.321	-0.321	0.188	0.161
$\mathbf{6_c}$	-0.233	1.141	0.946	1.114	0.762	-0.501	-0.611	-0.328	-0.309	0.133	0.157
$[\mathbf{6_c}\text{-}\mathbf{7a_c}]^{\ddagger}$	-0.363	1.031	0.976	1.070	0.785	-0.525	-0.663	0.019	-0.469	0.253	0.140
$\mathbf{7a_c}$	-0.340	0.961	0.932	1.081	0.746	-0.546	-0.635	0.183	-0.535	0.245	0.107
$[\mathbf{7a_c}\text{-}\mathbf{8a_c}]^{\ddagger}$	-0.447	0.946	0.946	1.091	0.760	-0.519	-0.592	0.264	-0.528	0.271	0.079
$\mathbf{8a_c}$	-0.447	0.917	0.929	1.151	0.745	-0.468	-0.541	0.073	-0.341	0.220	-0.002
$[\mathbf{8a_c}\text{-}\mathbf{9a_c}]^{\ddagger}$	-0.583	0.959	1.001	0.961	0.730	-0.433	-0.580	0.047	-0.292	0.205	0.182
$\mathbf{9a_c}$	-0.599	1.113	0.966	0.903	0.729	-0.416	-0.570	-0.123	-0.191	0.211	0.225
$[\mathbf{5_c}\text{-}\mathbf{9b_c}]^{\ddagger}$	-0.207	1.153	0.982	1.019	0.736	-0.565	-0.651	-0.420	-0.230	0.185	0.239
$[\mathbf{6_c}\text{-}\mathbf{7b_c}]^{\ddagger}$	-0.618	1.145	0.772	1.107	0.856	-0.457	-0.638	-0/207	-0.323	0.209	0.316
$\mathbf{7b_c}$	-0.592	1.063	0.777	1.106	0.820	-0.470	-0.653	0.192	-0.573	0.235	0.249
$\mathbf{8b_c}$	-0.574	1.034	0.778	1.124	0.793	-0.470	-0.634	0.193	-0.573	0.235	0.250

**[8b$_c$-9b$_c$]‡**	-0.600	1.153	0.779	1.149	0.766	-0.478	-0.626	-0.207	-0.304	0.213	-0.341
**9b$_c$**	-0.236	1.129	0.956	1.103	0.765	-0.493	-0.614	-0.318	-0.312	0.174	0.161
**[9b$_c$-10b$_c$]‡**	-0.340	1.052	0.953	1.058	0.754	-0.534	-0.648	-0.007	-0.459	0.233	0.152
**10b$_c$**	-0.340	0.942	0.961	1.069	0.765	-0.530	-0.645	0.161	-0.517	0.244	0.114
**[7a$_c$-10b$_c$]‡**	-1.121	1.097	1.060	1.169	0.762	-0.452	-0.643	0.373	-0.511	0.241	0.152
**[10b$_c$-11b$_c$]‡**	-0.455	0.957	0.956	1.086	0.776	-0.509	-0.586	0.241	-0.511	0.226	0.080
**11b$_c$**	-0.491	0.887	0.947	1.215	0.742	-0.444	-0.574	0.041	-0.320	0.176	0.019
**12b$_c$**	-0.492	0.828	0.949	1.225	0.789	-0.421	-0.618	-0.013	-0.239	0.184	0.031
**[12b$_c$-13b$_c$]‡**	-0.550	0.961	1.028	1.084	0.747	-0.427	-0.594	-0.039	-0.297	0.180	0.160
**13b$_c$**	-0.438	1.061	0.924	0.915	0.755	-0.372	-0.589	-0.061	-0.364	0.189	0.228
**[13b$_c$-1$_c$]‡**	-0.680	0.935	1.107	0.930	0.745	-0.370	-0.554	-0.001	-0.312	0.234	0.230

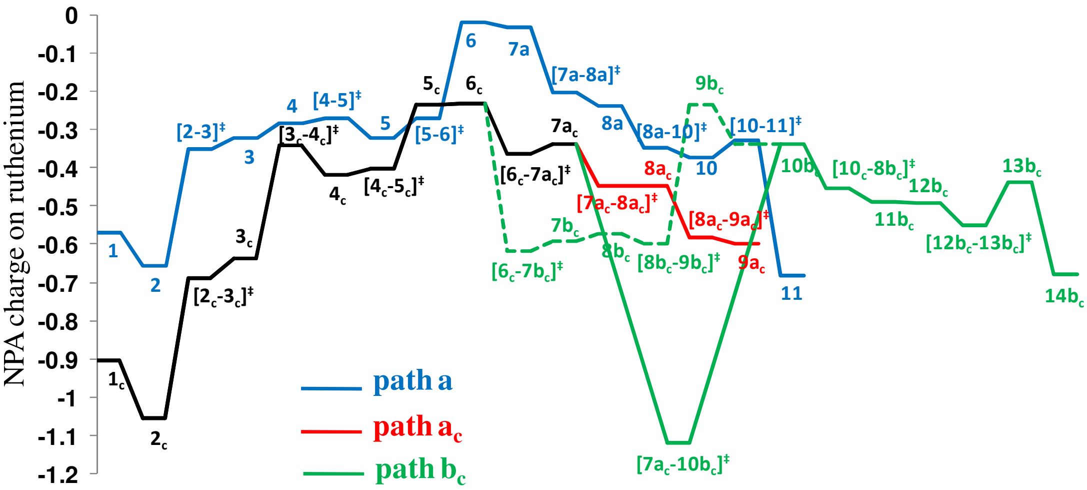

Figure S33. NPA charge on ruthenium center of intermediates and transition states involved in "path a" (blue line) for catalyst **1** (Scheme 3), "path a$_c$" (red line), "path b$_c$" (green line) and single-step isomerization of vinylidene **8a$_c$** (purple line) for catalyst **1$_c$** (Scheme 4).

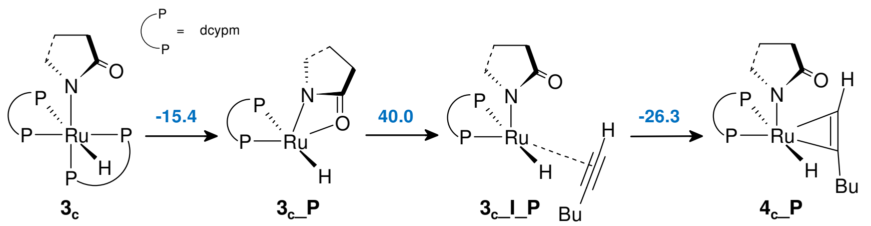

Figure S34. Reaction path involving dcypm decoordination, hexyne coordination and insertion step from ruthenium(II) intermediate $\mathbf{3_c}$. The energies (ΔG_L^S) above the arrows are in kcal/mol.

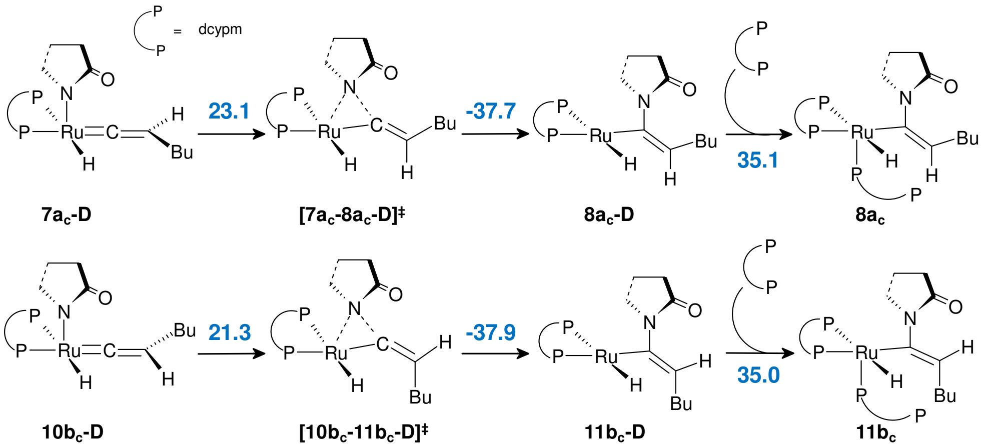

Figure S35. Nucleophilic transfer steps from 16e$^-$ vinylidene intermediates $\mathbf{7a_c}$**-D** and $\mathbf{10b_c}$**-D**. The energies (ΔG_L^S) above the arrows are in kcal/mol.

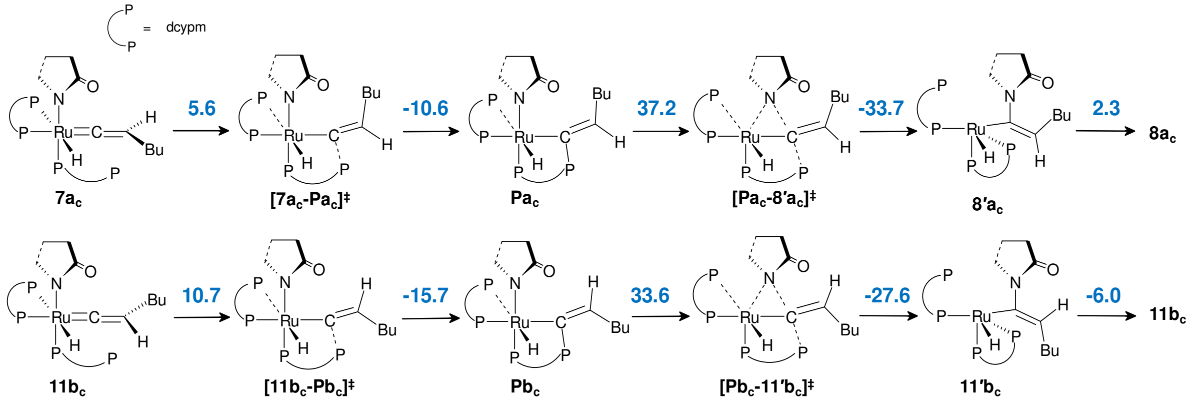

Figure S36. Reaction path involving C_α–P bond formation and nucleophilic transfer steps from vinylidene intermediates $\mathbf{7a_c}$ and $\mathbf{10b_c}$. The energies (ΔG_L^S) above the arrows are in kcal/mol.

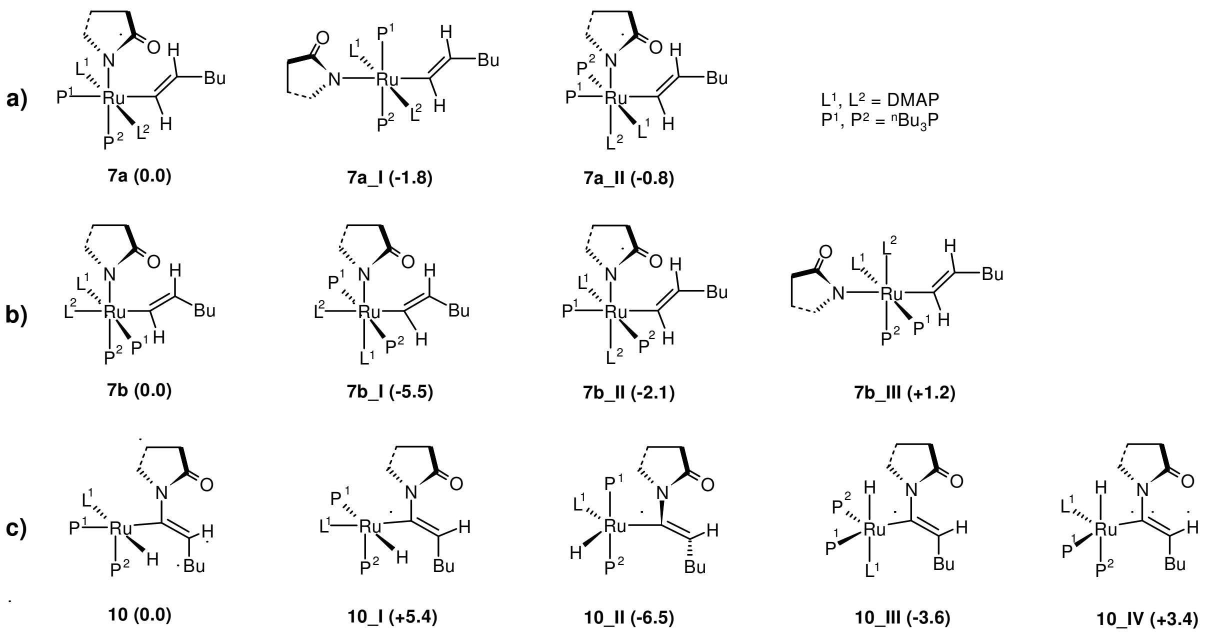

Figure S37. Optimized geometrical isomers of a) **7a**, b) **7b** and c) **10**. The energy values (kcal/mol) in parentheses represents ΔG_{298} (see Computational Details)

Table S9. Cartesian coordinates (Å) of the optimized structures involved in catalytic system **1** at BP86/LANL2DZ(Ru)/6-31G*(H, C, N, O & P) level.

I

16

XYZ

C	0.10423	0.00000	-0.14008
C	0.04128	0.00000	1.39656
H	1.14872	0.00000	-0.49995
H	-0.39616	0.89205	-0.56049
H	-0.39616	-0.89205	-0.56049
C	-1.40063	0.00000	1.93584
H	0.57961	-0.88597	1.78977
H	0.57961	0.88597	1.78977
C	-1.45314	0.00000	3.48614
H	-1.94570	0.88624	1.55784
H	-1.94570	-0.88624	1.55784
C	-2.82060	0.00000	4.01516
H	-0.90598	-0.88528	3.87074
H	-0.90598	0.88528	3.87074
C	-3.96780	0.00000	4.43094
H	-4.97635	0.00000	4.80183

II

13

XYZ

H	-0.03194	0.16950	0.04705
N	0.00673	-0.02028	1.04799
C	1.23293	-0.00757	1.69196
C	0.93039	-0.02355	3.20533
C	-0.57549	-0.34881	3.29952
C	-1.14983	0.11136	1.93034
O	2.33298	0.02765	1.14423
H	-1.51408	1.15986	1.97507
H	-1.98942	-0.52297	1.59223
H	1.58433	-0.74432	3.72220
H	1.16786	0.98067	3.60601
H	-1.08462	0.14518	4.14351
H	-0.72041	-1.43819	3.40885

III

40

XYZ

P	-0.17089	0.29446	-0.12056
C	-0.13476	-0.02433	1.73956
C	1.66902	0.10438	-0.49726
C	-0.77677	-1.39437	-0.70696
C	-1.51963	0.08384	2.40801
H	0.32022	-1.01506	1.94797
H	0.54229	0.73481	2.17926
C	-1.46981	-0.11575	3.93630
H	-1.96054	1.07484	2.18043
H	-2.21016	-0.66558	1.97053
C	-2.85149	-0.00694	4.60316
H	-1.02542	-1.10618	4.16112

H	-0.78377	0.63488	4.37779
H	-2.78540	-0.15172	5.69652
H	-3.30516	0.98532	4.42277
H	-3.54843	-0.76824	4.20579
C	-0.95474	-1.48553	-2.23547
H	-0.09220	-2.19096	-0.34805
H	-1.75412	-1.57029	-0.21519
C	-1.50831	-2.84816	-2.69904
H	-1.63362	-0.67780	-2.57454
H	0.01523	-1.30159	-2.74063
C	-1.68713	-2.93817	-4.22399
H	-0.82899	-3.65433	-2.35613
H	-2.48028	-3.03365	-2.19916
H	-2.08655	-3.92277	-4.52695
H	-2.38735	-2.16466	-4.59046
H	-0.72484	-2.79093	-4.74891
H	1.75184	-0.10205	-1.58288
C	2.49853	1.35778	-0.15490
H	2.07594	-0.78507	0.02745
C	3.98915	1.21460	-0.52309
H	2.07204	2.23506	-0.68098
H	2.41630	1.58127	0.92822
C	4.81652	2.46550	-0.18212
H	4.41224	0.33458	0.00183
H	4.07561	0.99557	-1.60640
H	5.87843	2.33568	-0.45823
H	4.43637	3.35448	-0.71889
H	4.77654	2.68859	0.90040

IV

19

XYZ

C	-0.11250	-0.06709	-0.02760
H	-0.14264	-0.14933	1.06840
C	1.11789	0.14096	-0.65853
C	1.16412	0.24666	-2.07671
C	-0.08578	0.14184	-2.74848
C	-1.24905	-0.06625	-2.00101
N	-1.30363	-0.17600	-0.65563
H	2.02359	0.22090	-0.05113
N	2.35212	0.43569	-2.76083
H	-0.15661	0.22250	-3.83665
H	-2.21220	-0.14781	-2.52501
C	2.32906	0.66489	-4.20149
C	3.58674	0.66399	-2.01775
H	4.41972	0.77585	-2.72847
H	3.54046	1.57862	-1.39025
H	3.81952	-0.19300	-1.35673
H	3.36187	0.77656	-4.56525
H	1.87378	-0.19159	-4.73509
H	1.76343	1.57991	-4.47581

V

73

XYZ

P	1.43075	0.60218	-0.57287
P	-1.43067	-0.60221	-0.57280
C	0.00004	0.00004	0.50084
H	-0.30507	0.83703	1.15884
H	0.30515	-0.83691	1.15890
C	2.55049	1.40372	0.74634
C	2.72277	0.72920	2.12785
C	3.92837	1.78100	0.14859
H	2.00812	2.36079	0.92228
C	3.52029	1.63789	3.09121
H	3.25911	-0.23172	2.01393
H	1.73749	0.49119	2.57036
C	4.73545	2.67885	1.11151
H	4.50696	0.85587	-0.05030
H	3.79659	2.28740	-0.82688
C	4.88985	2.02659	2.49981
H	3.65176	1.13106	4.06666
H	2.93425	2.55880	3.29134
H	5.72921	2.90203	0.67795
H	4.21330	3.65174	1.22035
H	5.42648	2.70820	3.18706
H	5.51687	1.11583	2.40649
C	2.17268	-1.06128	-1.11691
C	3.11422	-0.87609	-2.33399
C	2.82536	-1.98923	-0.06787
H	1.25772	-1.57276	-1.49269
C	3.50782	-2.23491	-2.95283
H	4.03315	-0.34240	-2.01586
H	2.62691	-0.23595	-3.09299
C	3.20277	-3.35423	-0.68747
H	3.74615	-1.51196	0.32347
H	2.15363	-2.14277	0.79813
C	4.13053	-3.18018	-1.90618
H	4.20859	-2.07923	-3.79553
H	2.60149	-2.71114	-3.38009
H	3.68477	-3.99605	0.07525
H	2.27694	-3.87775	-1.00254
H	4.35613	-4.16348	-2.36136
H	5.10022	-2.76048	-1.56667
C	-2.17268	1.06117	-1.11700
C	-3.11410	0.87584	-2.33414
C	-2.82556	1.98904	-0.06801
H	-1.25775	1.57273	-1.49272
C	-3.50791	2.23460	-2.95300
H	-4.03295	0.34196	-2.01612
H	-2.62661	0.23582	-3.09312
C	-3.20323	3.35396	-0.68764
H	-3.74625	1.51160	0.32337
H	-2.15386	2.14277	0.79797

C	-4.13089	3.17973	-1.90639
H	-4.20859	2.07877	-3.79576
H	-2.60164	2.71101	-3.38018
H	-3.68539	3.99567	0.07507
H	-2.27749	3.87766	-1.00266
H	-4.35666	4.16300	-2.36158
H	-5.10052	2.75983	-1.56694
C	-2.55038	-1.40366	0.74649
C	-2.72234	-0.72921	2.12807
C	-3.92842	-1.78059	0.14885
C	-3.51997	-1.63779	3.09145
H	-3.25842	0.23187	2.01433
H	-1.73694	-0.49152	2.57049
C	-4.73560	-2.67830	1.11181
H	-4.50682	-0.85533	-0.04995
H	-3.79684	-2.28699	-0.82664
C	-4.88970	-2.02610	2.50017
H	-3.65121	-1.13102	4.06696
H	-2.93415	-2.55887	3.29142
H	-5.72946	-2.90122	0.67834
H	-4.21367	-3.65133	1.22054
H	-5.42642	-2.70762	3.18743
H	-5.51651	-1.11518	2.40697
H	-2.00821	-2.36084	0.92230

VI

29

XYZ

C	0.13536	1.16880	0.55244
C	0.42971	2.47825	0.38746
N	1.08327	0.13650	0.28707
C	2.14459	0.22593	-0.62625
C	2.81582	-1.15609	-0.68471
C	2.35905	-1.85045	0.60821
C	0.99147	-1.19478	0.91563
O	2.46460	1.21815	-1.27758
H	1.40459	2.81965	0.03907
H	0.81038	-1.10038	2.00093
H	0.15494	-1.78068	0.48289
H	2.45237	-1.67963	-1.59064
H	3.90723	-1.04421	-0.78455
H	3.06798	-1.62999	1.42604
H	2.27557	-2.94641	0.52083
H	-0.35027	3.22540	0.56518
C	-1.25816	0.70781	0.94906
C	-2.07084	0.11831	-0.23016
H	-1.21630	-0.03788	1.76866
H	-1.79829	1.58043	1.35973
C	-3.48181	-0.33980	0.18430
H	-2.14222	0.88428	-1.02685
H	-1.52305	-0.73564	-0.67710
C	-4.29734	-0.90802	-0.98935
H	-3.39954	-1.10363	0.98397
H	-4.02528	0.51548	0.63375
H	-5.30445	-1.22576	-0.66489
H	-4.42407	-0.15484	-1.78881
H	-3.79629	-1.78654	-1.43687

VII

29

XYZ

C	-1.06192	1.09480	0.37369
N	-1.52198	-0.25808	0.03752
C	-2.90641	-0.32275	-0.19628
C	-3.47366	1.08665	0.01350
C	-2.24112	2.00715	-0.05338
C	-0.74028	-1.41661	-0.06321
C	0.56524	-1.62847	0.23082
O	-3.52467	-1.34348	-0.49188
H	0.90665	-2.65006	0.01303
C	1.63898	-0.72090	0.78431
H	-1.35506	-2.24247	-0.43992
H	-0.85310	1.17493	1.45961
H	-0.13096	1.32999	-0.17120
H	-4.24708	1.30267	-0.74102
H	-3.96503	1.11778	1.00560
H	-2.31938	2.89859	0.59039
H	-2.08357	2.35323	-1.09023
C	2.73936	-0.38103	-0.25276
H	1.22182	0.21569	1.19417
H	2.12228	-1.23472	1.64221
C	3.90106	0.43817	0.34099
H	3.13464	-1.32224	-0.68605
H	2.28267	0.17280	-1.09781
C	4.98984	0.77618	-0.69133
H	3.50218	1.37544	0.77933
H	4.35021	-0.12534	1.18348
H	5.80969	1.36172	-0.23777
H	5.43139	-0.14210	-1.12105
H	4.57727	1.36942	-1.52852

VIII

29

XYZ

C	0.12449	0.48900	0.04280
N	0.02337	-0.02654	1.40938
C	1.19950	-0.63369	1.86885
C	2.22979	-0.54129	0.73473
C	1.65116	0.51382	-0.22971
C	-1.15028	-0.02033	2.16883
C	-2.34832	0.46371	1.77026
O	1.33598	-1.15908	2.97236
H	-2.46647	0.88488	0.76192
C	-3.58264	0.43083	2.63695

H	-0.99296	-0.46409	3.15922
H	-0.42037	-0.17621	-0.65848
H	-0.33529	1.49247	-0.02251
H	3.22408	-0.29117	1.13902
H	2.31098	-1.53987	0.26292
H	1.87928	0.31310	-1.28932
H	2.05389	1.51209	0.01642
C	-4.72191	-0.43305	2.04272
H	-3.32127	0.05271	3.64498
H	-3.96821	1.46297	2.78359
C	-5.99910	-0.42646	2.90392
H	-4.96443	-0.06861	1.02309
H	-4.35872	-1.47260	1.91898
C	-7.13004	-1.28452	2.31239
H	-5.75356	-0.78832	3.92258
H	-6.35076	0.61783	3.02701
H	-8.03175	-1.26123	2.95049
H	-7.42099	-0.92523	1.30762
H	-6.81868	-2.34074	2.21044

1

119

XYZ

C	2.71221	-1.04697	1.24227
N	2.22912	-0.50255	0.08573
C	3.16581	0.03515	-0.74730
C	4.53704	0.06200	-0.47692
C	5.03592	-0.47837	0.73916
C	4.05926	-1.05111	1.60442
Ru	0.12825	-0.16447	-0.10415
P	-1.99080	0.54834	0.41604
C	-2.73096	2.06029	-0.45763
C	-3.06313	1.82237	-1.94203
C	-3.47933	3.10654	-2.68736
C	-3.80907	2.86655	-4.17048
N	6.38593	-0.44869	1.06625
C	7.35490	-0.06069	0.04653
N	0.72731	1.89786	-0.16519
C	0.54516	2.63652	-1.29824
C	1.11933	3.88899	-1.51808
C	1.94066	4.48509	-0.51890
C	2.11075	3.72618	0.67141
C	1.50816	2.47172	0.79386
N	2.53663	5.72488	-0.69729
C	3.19784	6.36915	0.43267
P	-0.18617	-2.39355	-0.51309
C	-0.01576	-3.70045	0.85789
C	-1.00625	-3.51314	2.02030
C	-0.76266	-4.48285	3.19427
C	-1.75616	-4.29006	4.35276
C	1.04864	-3.11196	-1.78169
C	2.39325	-3.66027	-1.26799
C	3.30764	-4.13660	-2.41625
C	4.65146	-4.69688	-1.92014
C	-1.75258	-3.09090	-1.31794
C	-2.12967	-2.42016	-2.64978
C	-3.45965	-2.93637	-3.23525
C	-3.82023	-2.28366	-4.58044
C	2.17169	6.53625	-1.85409
C	-1.84870	1.24110	2.18035
C	-3.05315	1.80496	2.96239
C	-2.62323	2.50731	4.26834
C	-3.80898	3.05025	5.08406
C	-3.50883	-0.58413	0.49415
C	-4.93077	-0.01057	0.66606
C	-6.00385	-1.11991	0.69884
C	-7.43454	-0.57203	0.83666
C	6.85822	-1.21466	2.21532
H	-1.07388	2.02801	2.08092
H	-1.36716	0.43066	2.76006
H	-3.62224	2.52562	2.34176
H	-3.75592	0.98778	3.21710
H	-2.03998	1.79673	4.88798
H	-1.93052	3.33701	4.02140
H	-3.47041	3.55164	6.00913
H	-4.39330	3.78510	4.49906
H	-4.49845	2.23665	5.37752
H	-3.62898	2.42198	0.08088
H	-1.96779	2.85840	-0.37456
H	-2.18327	1.36657	-2.44078
H	-3.87708	1.07404	-2.03238
H	-4.35626	3.55765	-2.18050
H	-2.66291	3.85246	-2.60377
H	-4.09776	3.80363	-4.68081
H	-2.94053	2.44387	-4.70931
H	-4.64683	2.15307	-4.28277
H	-3.29123	-1.31691	1.29652
H	-3.47976	-1.15699	-0.45009
H	-5.00833	0.58985	1.59014
H	-5.16175	0.67972	-0.16900
H	-5.92680	-1.72841	-0.22493
H	-5.78837	-1.81146	1.53808
H	-8.18092	-1.38684	0.85725
H	-7.55082	0.01263	1.76839
H	-7.69001	0.09520	-0.00773
H	0.52680	-3.91734	-2.33843
H	1.23608	-2.29465	-2.50312
H	2.92650	-2.88761	-0.68492
H	2.21871	-4.50991	-0.57849
H	2.77965	-4.90900	-3.01156
H	3.49192	-3.28974	-3.10744
H	5.29214	-5.02362	-2.75966
H	5.21004	-3.93393	-1.34636
H	4.50052	-5.56803	-1.25518

H	-0.11240	-4.71805	0.42435
H	1.01852	-3.61767	1.24544
H	-0.93363	-2.46534	2.37712
H	-2.04600	-3.64480	1.65718
H	-0.81953	-5.52768	2.82733
H	0.27279	-4.34726	3.56780
H	-1.55682	-4.99328	5.18194
H	-1.69799	-3.26377	4.76104
H	-2.79725	-4.45462	4.01716
H	-2.58198	-2.98677	-0.59454
H	-1.59927	-4.17963	-1.46773
H	-2.17936	-1.32298	-2.49983
H	-1.32133	-2.58434	-3.39081
H	-3.40525	-4.03702	-3.35994
H	-4.27456	-2.75412	-2.50475
H	-4.77856	-2.66738	-4.97572
H	-3.91284	-1.18647	-4.47848
H	-3.04092	-2.48069	-5.34033
H	1.65387	1.87104	1.69696
H	2.71197	4.09718	1.50522
H	-0.10099	2.17282	-2.04920
H	0.90920	4.39758	-2.46227
H	3.62078	7.33030	0.10211
H	2.50555	6.56445	1.27925
H	4.03161	5.74675	0.80902
H	2.75819	7.46781	-1.83947
H	2.40625	6.00711	-2.79681
H	1.09322	6.80255	-1.86815
H	1.95242	-1.49866	1.88689
H	4.33964	-1.51887	2.55150
H	2.77398	0.46694	-1.67285
H	5.20286	0.50899	-1.21949
H	7.94049	-1.05127	2.33546
H	6.36365	-0.87415	3.14395
H	6.67860	-2.30629	2.10975
H	8.36461	-0.07583	0.48546
H	7.34605	-0.73663	-0.83558
H	7.15918	0.96826	-0.30896

2

132

XYZ

C	1.172141	2.513208	1.188034
N	0.732556	1.852959	0.081771
C	0.880116	2.516049	-1.103155
C	1.437500	3.791212	-1.211162
C	1.903494	4.477342	-0.056276
C	1.740906	3.786608	1.175049
Ru	0.069143	-0.220004	0.300371
P	-0.347156	-2.437600	0.691977
C	0.973551	-3.679753	0.076283
C	2.122364	-4.050275	1.037101
C	3.183565	-4.952261	0.370818
C	4.301493	-5.376353	1.338832
N	2.484242	5.733189	-0.119705
C	2.717977	6.466362	1.120094
N	0.590642	-0.830837	-3.089772
C	1.064852	-2.147540	-3.521692
C	1.022711	-2.060881	-5.073344
C	1.165751	-0.552514	-5.357189
C	0.626787	0.119500	-4.074993
O	0.317762	1.319573	-3.966893
P	-2.067971	0.632302	0.413960
C	-2.312026	1.543489	2.078127
C	-3.247096	2.765536	2.197842
C	-3.329173	3.302180	3.642105
C	-4.234116	4.539086	3.777952
N	2.194852	-0.535572	0.438325
C	2.783178	-0.516495	1.670957
C	4.151631	-0.350433	1.886807
C	5.034567	-0.200134	0.780937
C	4.427037	-0.249220	-0.502364
C	3.044663	-0.408298	-0.619577
N	6.398682	-0.021628	0.945179
C	7.273692	-0.037358	-0.222352
C	-3.624663	-0.446081	0.354472
C	-5.029402	0.186415	0.385906
C	-6.147590	-0.842427	0.118974
C	-7.556964	-0.228064	0.173997
C	-2.554446	1.987013	-0.808642
C	-2.728640	1.508711	-2.261477
C	-3.055341	2.666965	-3.228998
C	-3.094546	2.222843	-4.701121
C	6.984408	-0.101320	2.278430
C	-1.857533	-3.393409	0.052614
C	-2.010119	-3.393837	-1.478083
C	-3.293401	-4.098916	-1.960755
C	-3.424357	-4.137576	-3.492950
C	-0.447575	-3.015695	2.503694
C	-1.548719	-2.332102	3.330562
C	-1.492922	-2.684324	4.831729
C	-2.595887	-1.991593	5.650786
C	2.434438	6.481041	-1.373580
H	0.293352	-0.623118	-2.080005
H	-1.294898	1.842307	2.392798
H	-2.623903	0.759830	2.797963
H	-2.886864	3.576328	1.535276
H	-4.268490	2.519009	1.850499
H	-3.699044	2.497420	4.309396
H	-2.308307	3.548070	3.997893
H	-4.282162	4.894964	4.823351
H	-3.864682	5.375522	3.155553
H	-5.266924	4.315165	3.452121
H	-3.480804	2.481051	-0.458556

H	-1.755648	2.751312	-0.769768
H	-1.799696	1.011787	-2.598848
H	-3.536854	0.751686	-2.321401
H	-4.023310	3.128947	-2.944106
H	-2.288000	3.457756	-3.110084
H	-3.353769	3.062607	-5.372433
H	-2.106982	1.832740	-5.004725
H	-3.844113	1.424258	-4.860424
H	-3.518325	-1.152323	1.200480
H	-3.510673	-1.051827	-0.565513
H	-5.210901	0.657572	1.371671
H	-5.107281	0.997243	-0.365898
H	-5.983380	-1.305518	-0.875135
H	-6.075287	-1.667028	0.856894
H	-8.337969	-0.988509	-0.008126
H	-7.755889	0.226386	1.162669
H	-7.677910	0.565701	-0.586721
H	0.453789	-4.610474	-0.232046
H	1.399834	-3.236988	-0.844041
H	2.617850	-3.137091	1.418966
H	1.716458	-4.582653	1.919072
H	2.692962	-5.851532	-0.051389
H	3.627443	-4.412085	-0.490653
H	5.056571	-6.008690	0.837214
H	4.822858	-4.494222	1.754883
H	3.894333	-5.954020	2.190734
H	-0.563302	-4.118752	2.543610
H	0.533879	-2.785960	2.961465
H	-1.445863	-1.236985	3.201243
H	-2.549117	-2.604890	2.936470
H	-1.567673	-3.782958	4.962647
H	-0.499718	-2.395640	5.233337
H	-2.529368	-2.247849	6.724055
H	-2.525800	-0.891402	5.562373
H	-3.601400	-2.289194	5.297476
H	-2.764941	-2.960883	0.511371
H	-1.779362	-4.433642	0.430127
H	-1.990379	-2.345904	-1.844673
H	-1.134182	-3.895995	-1.939082
H	-3.318381	-5.132299	-1.558896
H	-4.174060	-3.582894	-1.527042
H	-4.350425	-4.651889	-3.808960
H	-3.445951	-3.117079	-3.918447
H	-2.571417	-4.671666	-3.953013
H	2.096467	-2.338434	-3.154564
H	0.417383	-2.949360	-3.126782
H	0.613047	-0.195642	-6.241874
H	2.223397	-0.248384	-5.485467
H	1.802885	-2.682136	-5.544944
H	0.041822	-2.420018	-5.434473
H	0.534893	1.999104	-2.005938
H	1.497507	4.236094	-2.206892
H	1.053445	1.969887	2.130061
H	2.048638	4.229826	2.126052
H	2.982700	7.427717	-1.249166
H	2.924481	5.914404	-2.186723
H	1.397016	6.714618	-1.693582
H	3.183594	7.435016	0.882008
H	1.782953	6.657668	1.688195
H	3.413766	5.913570	1.779718
H	2.571003	-0.439440	-1.604720
H	5.019582	-0.166283	-1.416375
H	2.095917	-0.657515	2.511162
H	4.522201	-0.360159	2.915229
H	8.311508	0.135478	0.101482
H	7.235975	-1.004132	-0.767142
H	7.002854	0.767329	-0.933254
H	8.057157	0.137020	2.214761
H	6.516688	0.633545	2.960769
H	6.879034	-1.109373	2.733671

[2-3]‡

132

XYZ

C	0.89806	2.54265	1.27495
N	0.50791	1.90285	0.14158
C	0.56102	2.62209	-1.01676
C	0.97421	3.95315	-1.06359
C	1.37629	4.63295	0.11996
C	1.32120	3.86978	1.32098
Ru	-0.05524	-0.19491	0.08215
P	-0.30209	-2.44986	-0.36753
C	1.20588	-3.08864	-1.31479
C	2.39958	-3.61120	-0.49411
C	3.58399	-4.02528	-1.39191
C	4.78717	-4.55094	-0.59125
N	1.79643	5.94826	0.10543
C	2.09648	6.62983	1.35974
N	1.87604	0.06018	-2.77060
C	3.11722	-0.19291	-3.51373
C	2.72759	-0.39359	-5.00938
C	1.42951	0.42063	-5.10650
C	0.92214	0.43346	-3.63418
O	-0.26975	0.80833	-3.36378
P	-2.12408	0.36003	0.70900
C	-2.26797	0.54756	2.59796
C	-3.16324	1.65095	3.19990
C	-3.16119	1.63113	4.74294
C	-4.02695	2.74202	5.36144
N	2.01677	-0.48155	1.03631
C	2.26519	-0.87115	2.31229
C	3.53112	-0.87579	2.89730
C	4.66196	-0.46338	2.13525
C	4.39778	-0.07919	0.78959

C	3.09329	-0.09027	0.29766
N	5.93474	-0.43932	2.66903
C	7.07490	-0.12320	1.81233
C	-3.60320	-0.72731	0.29745
C	-5.00457	-0.31202	0.78803
C	-6.10649	-1.26224	0.27232
C	-7.51313	-0.87159	0.75595
C	-2.72618	2.02324	0.05812
C	-2.93904	2.03075	-1.46974
C	-3.08309	3.44827	-2.05817
C	-3.18632	3.42193	-3.59317
C	6.16757	-0.94534	4.01821
C	-1.64309	-3.03615	-1.55374
C	-1.54518	-2.40277	-2.95653
C	-2.79527	-2.65118	-3.82378
C	-2.65543	-2.03288	-5.22545
C	-0.44015	-3.76053	0.98903
C	-1.70201	-3.69155	1.86505
C	-1.69572	-4.72098	3.01440
C	-2.95693	-4.65323	3.89169
C	1.73073	6.71585	-1.13548
H	-0.72373	0.04942	-1.33711
H	-1.22879	0.69226	2.95030
H	-2.57841	-0.44663	2.97896
H	-2.81423	2.64299	2.85286
H	-4.20381	1.54668	2.83808
H	-3.51730	0.64245	5.09626
H	-2.11786	1.72743	5.10587
H	-4.00682	2.70372	6.46566
H	-3.67210	3.74327	5.05364
H	-5.08187	2.65111	5.04218
H	-3.65373	2.32172	0.58419
H	-1.95290	2.76444	0.33174
H	-2.09028	1.52731	-1.97064
H	-3.84455	1.44172	-1.72247
H	-3.96469	3.96091	-1.62082
H	-2.19665	4.04422	-1.76025
H	-3.22677	4.44222	-4.01771
H	-2.31555	2.89556	-4.02475
H	-4.09696	2.88696	-3.92306
H	-3.35838	-1.73402	0.68209
H	-3.60155	-0.80740	-0.80601
H	-5.03336	-0.30005	1.89547
H	-5.23748	0.71868	0.45649
H	-6.08377	-1.27484	-0.83553
H	-5.87596	-2.29728	0.59519
H	-8.28055	-1.56628	0.36952
H	-7.57515	-0.88445	1.86028
H	-7.78291	0.14668	0.41910
H	0.84975	-3.89342	-1.98959
H	1.51564	-2.23290	-1.94881
H	2.74413	-2.83702	0.21571

H	2.09698	-4.48590	0.11544
H	3.24778	-4.80053	-2.10959
H	3.89419	-3.15495	-2.00315
H	5.62161	-4.84180	-1.25525
H	5.16497	-3.78022	0.10670
H	4.51276	-5.43816	0.01031
H	-0.35813	-4.76176	0.51809
H	0.45487	-3.63515	1.62890
H	-1.79435	-2.67111	2.28865
H	-2.60524	-3.85671	1.24368
H	-1.59176	-5.74036	2.59161
H	-0.79633	-4.55728	3.64183
H	-2.92466	-5.39882	4.70656
H	-3.06842	-3.65488	4.35466
H	-3.86869	-4.84703	3.29645
H	-2.62415	-2.80165	-1.10356
H	-1.57693	-4.14226	-1.61815
H	-1.36959	-1.31226	-2.87600
H	-0.65745	-2.80715	-3.48178
H	-2.99463	-3.73972	-3.90750
H	-3.68033	-2.21641	-3.31502
H	-3.58567	-2.13752	-5.81364
H	-2.40716	-0.95865	-5.15189
H	-1.84197	-2.52116	-5.79413
H	3.82399	0.66725	-3.41630
H	3.65615	-1.07638	-3.10819
H	0.66585	0.00148	-5.78476
H	1.61378	1.46515	-5.43217
H	3.52208	-0.07922	-5.71181
H	2.51944	-1.46392	-5.19937
H	0.24013	2.08001	-1.92204
H	0.98103	4.45122	-2.03618
H	0.86451	1.94583	2.19268
H	1.60647	4.29603	2.28615
H	2.11122	7.73183	-0.95095
H	2.35649	6.25597	-1.92403
H	0.69446	6.79708	-1.52264
H	2.42445	7.65757	1.14244
H	1.21445	6.68244	2.03151
H	2.91471	6.12201	1.90655
H	2.85606	0.19133	-0.74135
H	5.20259	0.23002	0.11814
H	1.39508	-1.20691	2.88744
H	3.63045	-1.21114	3.93272
H	7.99343	-0.13117	2.41830
H	7.19576	-0.85501	0.98750
H	6.97057	0.88402	1.36584
H	7.23051	-0.81732	4.27235
H	5.57151	-0.38424	4.76346
H	5.91524	-2.02172	4.11419

3

132

XYZ

C	0.63650	2.41457	-1.90507
N	0.88514	2.00671	-0.63475
C	1.64576	2.85102	0.11980
C	2.16513	4.05674	-0.34841
C	1.91686	4.47712	-1.68539
C	1.11031	3.60132	-2.46432
Ru	0.00331	0.27871	0.39679
P	-0.83680	-1.24417	1.94929
C	-1.72269	-0.56143	3.47538
C	-3.10808	0.08783	3.31980
C	-3.71167	0.48874	4.68272
C	-5.09839	1.14188	4.55949
N	2.42715	5.65628	-2.19284
C	2.02285	6.10728	-3.52038
N	-0.85907	1.92265	1.55237
C	-1.66880	2.98196	0.92610
C	-2.06219	3.95477	2.06745
C	-0.89057	3.79554	3.04369
C	-0.34673	2.37284	2.73590
O	0.46009	1.78808	3.49754
P	1.44583	-1.12103	-0.81370
C	0.90610	-1.68118	-2.55082
C	1.72374	-2.66965	-3.40501
C	1.17156	-2.79783	-4.84115
C	1.95874	-3.79922	-5.70321
N	-1.97609	0.22026	-0.97075
C	-2.05937	0.04624	-2.31522
C	-3.24340	-0.05267	-3.04873
C	-4.49622	0.01680	-2.38060
C	-4.42330	0.21491	-0.97363
C	-3.18176	0.30728	-0.34590
N	-5.69915	-0.10418	-3.04732
C	-6.95249	0.07593	-2.32103
C	2.12054	-2.70726	-0.03537
C	3.41970	-3.38206	-0.52783
C	3.75914	-4.63307	0.31053
C	5.06720	-5.31422	-0.12645
C	3.06105	-0.26043	-1.28225
C	3.93802	0.17756	-0.09549
C	5.13725	1.04733	-0.52384
C	6.03020	1.45542	0.65997
C	-5.71462	-0.21484	-4.50178
C	0.35065	-2.35330	2.91360
C	1.53069	-1.62457	3.58297
C	2.37356	-2.55807	4.47450
C	3.55970	-1.83751	5.13765
C	-2.10674	-2.55284	1.44394
C	-1.67535	-3.48411	0.30040
C	-2.80304	-4.41566	-0.18824
C	-2.36050	-5.35122	-1.32558

C	3.13843	6.57554	-1.30895
H	1.22788	0.38677	1.41928
H	0.80737	-0.72983	-3.11495
H	-0.12332	-2.07088	-2.43142
H	2.78435	-2.35500	-3.45675
H	1.71491	-3.66945	-2.93020
H	0.10643	-3.10270	-4.79588
H	1.18478	-1.80009	-5.32520
H	1.54397	-3.86853	-6.72523
H	3.02028	-3.50178	-5.79164
H	1.93328	-4.81291	-5.26190
H	3.64009	-0.92099	-1.95760
H	2.77258	0.62831	-1.87474
H	3.31568	0.73017	0.63195
H	4.31381	-0.71325	0.44745
H	5.74269	0.50277	-1.27704
H	4.75638	1.95641	-1.03140
H	6.87443	2.09021	0.33375
H	5.45354	2.02088	1.41521
H	6.45428	0.56773	1.16550
H	1.28801	-3.43777	-0.06069
H	2.25382	-2.43239	1.02550
H	3.34501	-3.67452	-1.59008
H	4.26628	-2.67214	-0.46241
H	3.82751	-4.34726	1.37934
H	2.92192	-5.35656	0.24125
H	5.28309	-6.20790	0.48671
H	5.01725	-5.63714	-1.18332
H	5.92781	-4.62627	-0.02963
H	-1.80490	-1.41269	4.18252
H	-1.01503	0.17198	3.90393
H	-3.01004	0.99225	2.69154
H	-3.81515	-0.59985	2.81037
H	-3.78104	-0.40911	5.33004
H	-3.01588	1.18266	5.19343
H	-5.50956	1.41262	5.54915
H	-5.05194	2.06599	3.95307
H	-5.82118	0.45972	4.07249
H	-2.37464	-3.14054	2.34598
H	-3.02008	-2.00825	1.13696
H	-1.31506	-2.86190	-0.54254
H	-0.81417	-4.10467	0.62192
H	-3.17749	-5.01867	0.66356
H	-3.65882	-3.79780	-0.52719
H	-3.18953	-5.99756	-1.66719
H	-2.00262	-4.77424	-2.19890
H	-1.53368	-6.01127	-1.00250
H	0.72030	-3.14262	2.23443
H	-0.26098	-2.86883	3.68216
H	2.17148	-1.17164	2.80208
H	1.16393	-0.76410	4.17245
H	1.72700	-3.00445	5.25755

H	2.74787	-3.40973	3.86840
H	4.14809	-2.52283	5.77529
H	4.24275	-1.41325	4.37848
H	3.21127	-1.00078	5.77046
H	-1.08634	3.52121	0.14498
H	-2.55665	2.56921	0.41382
H	-1.15444	3.89219	4.11087
H	-0.08161	4.52698	2.84121
H	-2.22614	4.98782	1.70857
H	-3.00225	3.61490	2.54387
H	1.81211	2.52727	1.15141
H	2.75726	4.66521	0.33945
H	0.00036	1.75289	-2.49892
H	0.83665	3.83916	-3.49541
H	3.48895	7.43883	-1.89464
H	2.49705	6.95072	-0.48463
H	4.02460	6.08777	-0.86060
H	2.54340	7.04800	-3.75530
H	2.29985	5.36546	-4.29390
H	0.92972	6.28764	-3.59077
H	-3.13305	0.46564	0.73373
H	-5.32462	0.30262	-0.36150
H	-1.10549	-0.03793	-2.84155
H	-3.17916	-0.18985	-4.13138
H	-7.79485	-0.07869	-3.01204
H	-7.04839	-0.65994	-1.49941
H	-7.04292	1.09223	-1.88514
H	-6.75383	-0.33350	-4.84393
H	-5.28682	0.68152	-4.99729
H	-5.14284	-1.10042	-4.84081

4

113

XYZ

C	2.76757	-1.61354	-1.07685
N	1.85653	-1.34009	-0.10319
C	2.24117	-1.66432	1.15877
C	3.46917	-2.23497	1.49133
C	4.42561	-2.50666	0.47175
C	4.02509	-2.17077	-0.85279
Ru	-0.06545	-0.29046	-0.73683
N	5.65962	-3.05901	0.74797
C	5.98623	-3.46442	2.11134
P	0.61886	1.82184	-0.16184
C	1.42561	2.50227	-1.72598
C	0.50575	2.46002	-2.96546
C	1.24648	2.79464	-4.27477
C	0.31415	2.75632	-5.49780
N	-0.42667	-1.86776	-2.07615
C	-1.12455	-1.70459	-3.25569
C	-1.23479	-3.06988	-3.98508
C	-0.93148	-4.09127	-2.88088
C	-0.01832	-3.27786	-1.92700
O	-1.58299	-0.64484	-3.72222
P	-1.57289	-0.57282	0.84908
C	-1.18458	-0.02269	2.62771
C	-2.32523	0.41459	3.57106
C	-1.81046	0.78536	4.97797
C	-2.92654	1.26892	5.91928
C	2.01086	2.05110	1.09990
C	2.64425	3.43341	1.35353
C	3.88141	3.35847	2.27371
C	4.51609	4.73323	2.54413
C	-0.64461	3.14275	0.31056
C	-0.39861	4.61996	-0.05925
C	-1.52316	5.54451	0.45277
C	-1.31977	7.01648	0.05597
C	-1.93986	-2.40710	1.07243
C	-2.99858	-2.84510	2.10399
C	-3.21932	-4.37292	2.10371
C	-4.25845	-4.83263	3.14015
C	-3.28364	0.14254	0.53764
C	-3.98267	-0.41511	-0.71765
C	-5.28864	0.33713	-1.04284
C	-6.00421	-0.22910	-2.28100
C	6.56571	-3.39665	-0.34591
H	-1.28851	0.43786	-1.47852
H	2.78974	1.33724	0.76492
H	1.62730	1.63707	2.05404
H	2.94076	3.90061	0.39367
H	1.89871	4.11171	1.81258
H	3.59357	2.88633	3.23471
H	4.63281	2.68486	1.81482
H	5.40165	4.64890	3.19965
H	4.84003	5.21545	1.60303
H	3.79842	5.41546	3.03679
H	1.80777	3.52875	-1.55466
H	2.31326	1.85885	-1.89665
H	0.02802	1.46328	-3.05187
H	-0.33208	3.17370	-2.82918
H	1.72598	3.79226	-4.19456
H	2.07235	2.06760	-4.41671
H	0.86555	2.94947	-6.43623
H	-0.17888	1.77110	-5.58187
H	-0.48241	3.51904	-5.41331
H	-0.79577	3.04883	1.40563
H	-1.58657	2.80325	-0.15934
H	0.56943	4.97147	0.34371
H	-0.32959	4.71800	-1.15969
H	-2.49548	5.18536	0.06025
H	-1.58924	5.46259	1.55657
H	-2.13774	7.65427	0.43685
H	-0.36952	7.41339	0.45960
H	-1.28851	7.13270	-1.04327

H	-2.21402	-2.76275	0.06162
H	-0.96487	-2.87887	1.30531
H	-2.69249	-2.52943	3.12106
H	-3.96417	-2.34183	1.89762
H	-3.53665	-4.69266	1.09138
H	-2.25214	-4.87949	2.29549
H	-4.39726	-5.92848	3.11389
H	-3.94993	-4.55857	4.16641
H	-5.24334	-4.36581	2.95299
H	-0.61990	-0.86116	3.08552
H	-0.46243	0.80806	2.52706
H	-2.85037	1.28831	3.13846
H	-3.08328	-0.38640	3.66455
H	-1.30210	-0.09346	5.42348
H	-1.03531	1.57283	4.88626
H	-2.52910	1.52777	6.91722
H	-3.42851	2.16726	5.51466
H	-3.69935	0.49013	6.05799
H	-3.14061	1.23487	0.42954
H	-3.91878	-0.01286	1.43186
H	-3.29433	-0.35367	-1.58189
H	-4.21437	-1.48964	-0.56998
H	-5.97068	0.30419	-0.16815
H	-5.05388	1.40756	-1.21054
H	-6.92538	0.33691	-2.51113
H	-5.34726	-0.18705	-3.16850
H	-6.29065	-1.28667	-2.12760
H	1.04913	-3.41780	-2.21142
H	-0.10670	-3.60490	-0.87493
H	-2.22585	-3.17221	-4.45817
H	-0.47968	-3.10200	-4.79638
H	-0.45749	-5.02504	-3.23474
H	-1.86637	-4.36835	-2.35657
H	1.51083	-1.44068	1.94339
H	3.67412	-2.46368	2.54034
H	2.44733	-1.37269	-2.09793
H	4.67863	-2.35045	-1.71004
H	7.01077	-3.86490	2.13282
H	5.94372	-2.60457	2.80797
H	5.29976	-4.24948	2.48991
H	7.49186	-3.82032	0.07040
H	6.12433	-4.14283	-1.03788
H	6.83746	-2.49933	-0.93533

[4-5]‡

129

XYZ

C	2.11973	-1.45655	-2.21526
N	1.65268	-1.46900	-0.93849
C	2.36623	-2.24198	-0.07747
C	3.50228	-2.97433	-0.41744
C	3.99157	-2.94895	-1.75451
C	3.24043	-2.15343	-2.66491
Ru	-0.26516	-0.32952	-0.37289
C	-2.25748	-0.42523	-3.01111
C	-1.22054	-0.62586	-3.63432
N	5.11747	-3.64794	-2.13994
C	5.77595	-4.54108	-1.19102
P	0.71169	1.75167	-0.73620
C	0.24078	2.51961	-2.39316
C	-1.23023	2.96217	-2.50015
C	-1.61702	3.39408	-3.92843
C	-3.06129	3.91340	-4.02794
N	-1.33133	-2.19636	-0.40420
C	-2.60094	-2.45598	0.01075
C	-3.04261	-3.86783	-0.45176
C	-1.72569	-4.55256	-0.83630
C	-0.79916	-3.34826	-1.15492
O	-3.36795	-1.70117	0.66257
P	-0.18865	-0.21552	1.85132
C	1.52080	0.10785	2.62066
C	1.62934	0.78746	4.00294
C	3.09295	0.91021	4.47633
C	3.22923	1.60674	5.84099
C	2.60001	1.73015	-0.89736
C	3.43281	3.02151	-1.01481
C	4.90958	2.73658	-1.36337
C	5.76925	4.01001	-1.43258
C	0.34503	3.16946	0.45827
C	0.62657	4.64116	0.08573
C	0.06447	5.61969	1.13926
C	0.33179	7.09393	0.79157
C	-0.70904	-1.83386	2.65936
C	-0.51404	-2.03215	4.17450
C	-1.06742	-3.39302	4.65010
C	-0.88140	-3.62589	6.15889
C	-1.26857	1.02511	2.76884
C	-2.72286	1.14614	2.27321
C	-3.62803	1.85540	3.30031
C	-5.06844	2.03954	2.79335
C	5.50306	-3.68312	-3.54776
H	-1.67399	0.40875	-0.15629
H	2.78706	1.09459	-1.78579
H	2.96382	1.13144	-0.03999
H	3.00827	3.69527	-1.78525
H	3.39541	3.57882	-0.05900
H	5.33373	2.04158	-0.61099
H	4.95592	2.20202	-2.33350
H	6.81850	3.77695	-1.68905
H	5.38518	4.71114	-2.19684
H	5.77205	4.54346	-0.46386
H	0.91759	3.36805	-2.61793
H	0.43799	1.73714	-3.15211
H	-1.87725	2.12849	-2.17245

H	-1.42701	3.80044	-1.80240
H	-0.91690	4.17942	-4.27995
H	-1.48499	2.52974	-4.60863
H	-3.31619	4.20679	-5.06244
H	-3.78575	3.14017	-3.71172
H	-3.21518	4.79708	-3.38064
H	0.86986	2.92076	1.40207
H	-0.73400	3.05169	0.67627
H	1.71157	4.81768	-0.02259
H	0.17706	4.88073	-0.89679
H	-1.02639	5.45439	1.24421
H	0.50589	5.38582	2.12901
H	-0.08311	7.77172	1.55918
H	1.41624	7.29770	0.71514
H	-0.12668	7.36476	-0.17770
H	-1.77510	-1.94073	2.38212
H	-0.17671	-2.62865	2.10348
H	0.56062	-1.97247	4.44136
H	-1.02171	-1.22817	4.74377
H	-2.14245	-3.45509	4.39165
H	-0.56932	-4.20552	4.08392
H	-1.28414	-4.60717	6.46863
H	0.18809	-3.59826	6.44106
H	-1.40134	-2.84935	6.75048
H	2.03225	-0.87536	2.65354
H	2.07840	0.70650	1.87687
H	1.17858	1.79825	3.96241
H	1.05230	0.22410	4.75996
H	3.54372	-0.10147	4.52868
H	3.67740	1.46562	3.71523
H	4.28622	1.67695	6.15538
H	2.82030	2.63368	5.80841
H	2.68118	1.05561	6.62762
H	-0.77113	2.01174	2.73271
H	-1.25612	0.71666	3.83332
H	-2.72780	1.70986	1.32034
H	-3.12959	0.15139	2.01248
H	-3.64203	1.26956	4.24228
H	-3.19739	2.84416	3.56326
H	-5.70623	2.53323	3.54948
H	-5.09212	2.65914	1.87742
H	-5.52655	1.06504	2.54474
H	-0.79410	-3.12431	-2.24533
H	0.24617	-3.56042	-0.86982
H	-3.61532	-4.37373	0.34443
H	-3.72426	-3.75530	-1.31865
H	-1.80662	-5.25903	-1.68231
H	-1.32417	-5.11438	0.02851
H	1.98295	-2.27791	0.94653
H	3.99302	-3.56748	0.35834
H	1.54309	-0.84400	-2.91729
H	3.51826	-2.07608	-3.71913
H	6.65954	-4.98811	-1.67076
H	6.12038	-3.98818	-0.29619
H	5.11016	-5.36270	-0.85502
H	6.42767	-4.27017	-3.65315
H	4.72288	-4.14662	-4.18635
H	5.70360	-2.66470	-3.93233
H	-0.36304	-0.84704	-4.24154
C	-3.54825	-0.21552	-2.34760
C	-4.68016	-1.10280	-2.93006
H	-3.43591	-0.42427	-1.26524
H	-3.83750	0.85209	-2.43215
C	-5.95798	-1.00843	-2.07635
H	-4.88775	-0.81254	-3.97918
H	-4.33224	-2.15327	-2.95738
C	-7.10400	-1.88139	-2.61321
H	-5.70456	-1.30571	-1.04053
H	-6.28935	0.04879	-2.02687
H	-8.00386	-1.79958	-1.97709
H	-7.39098	-1.58578	-3.64016
H	-6.81269	-2.94802	-2.64325

5

129

XYZ

C	1.85619	0.08599	-2.84587
N	1.72806	-0.41526	-1.59031
C	2.86310	-0.94622	-1.06699
C	4.10082	-0.97228	-1.70784
C	4.23526	-0.42318	-3.01394
C	3.04121	0.10730	-3.57915
Ru	-0.32907	-0.55234	-0.48184
C	-2.11217	-1.21227	-1.58110
C	-1.24543	-0.86140	-2.44610
N	5.43869	-0.40958	-3.68648
C	6.60451	-1.06777	-3.10262
P	-0.78106	1.74514	-0.60745
C	-2.05210	2.36791	-1.85498
C	-3.50989	1.92813	-1.63512
C	-4.49081	2.62840	-2.59730
C	-5.94954	2.18647	-2.39341
N	-0.03500	-2.72429	-0.46793
C	-0.73781	-3.60582	0.29051
C	-0.63455	-5.03057	-0.31318
C	0.52939	-4.91672	-1.30591
C	0.52668	-3.40286	-1.64502
O	-1.40673	-3.36595	1.32803
P	0.63906	-0.46647	1.72142
C	2.07850	0.75311	1.95396
C	2.35557	1.37012	3.34090
C	3.59469	2.28968	3.33198
C	3.86938	2.94186	4.69751
C	0.69817	2.77933	-1.19104

C	0.74849	4.30592	-0.99297
C	1.98629	4.94300	-1.66012
C	2.07070	6.46361	-1.44589
C	-1.34324	2.60183	0.98286
C	-2.05401	3.97158	0.96268
C	-2.44068	4.43843	2.38251
C	-3.16060	5.79731	2.39435
C	1.36748	-2.09762	2.31223
C	2.28648	-2.12259	3.54681
C	2.73654	-3.55664	3.90015
C	3.67167	-3.61157	5.11979
C	-0.46515	-0.03824	3.18506
C	-1.85176	-0.70987	3.20900
C	-2.53976	-0.56098	4.58045
C	-3.95067	-1.17294	4.60473
C	5.49644	0.07238	-5.06379
H	-1.57306	-0.69850	0.51534
H	0.77909	2.55422	-2.27263
H	1.59064	2.31417	-0.73345
H	-0.16341	4.78144	-1.40473
H	0.76443	4.54579	0.08831
H	2.90202	4.45956	-1.26459
H	1.96774	4.72051	-2.74593
H	2.96326	6.89259	-1.93591
H	1.18254	6.97605	-1.86001
H	2.12703	6.71401	-0.37017
H	-1.98714	3.47399	-1.88123
H	-1.70819	2.00359	-2.84241
H	-3.57018	0.83531	-1.77463
H	-3.82479	2.12745	-0.59157
H	-4.41408	3.72711	-2.46615
H	-4.18358	2.42307	-3.64258
H	-6.63077	2.69945	-3.09610
H	-6.06198	1.09798	-2.55053
H	-6.29529	2.41097	-1.36715
H	-0.43978	2.66422	1.62217
H	-2.00182	1.86002	1.47239
H	-1.41474	4.74123	0.49457
H	-2.97040	3.91533	0.34520
H	-3.08614	3.67164	2.85541
H	-1.52768	4.49772	3.00885
H	-3.42304	6.10587	3.42223
H	-2.52602	6.59104	1.95802
H	-4.09686	5.75966	1.80708
H	0.48120	-2.74561	2.45499
H	1.89154	-2.53375	1.44153
H	3.18628	-1.49769	3.37356
H	1.77196	-1.68786	4.42727
H	1.84049	-4.18051	4.08536
H	3.24325	-4.00434	3.02172
H	3.97906	-4.64820	5.34746
H	4.59033	-3.01972	4.94785

H	3.17764	-3.20384	6.02141
H	2.98688	0.23161	1.59308
H	1.89938	1.56730	1.22789
H	1.47802	1.95760	3.67365
H	2.49924	0.57810	4.09878
H	4.48102	1.70434	3.01425
H	3.45877	3.07791	2.56416
H	4.75620	3.59989	4.66042
H	3.01052	3.55531	5.02747
H	4.05095	2.17845	5.47650
H	-0.57723	1.06083	3.22494
H	0.10097	-0.32152	4.09380
H	-2.48782	-0.25161	2.42620
H	-1.77241	-1.77400	2.92051
H	-1.91451	-1.04504	5.35835
H	-2.59456	0.51225	4.85841
H	-4.42245	-1.06918	5.59902
H	-4.61070	-0.68031	3.86651
H	-3.91917	-2.24851	4.35337
H	-0.09734	-3.19342	-2.54243
H	1.54314	-3.03539	-1.87548
H	-0.50236	-5.78720	0.47910
H	-1.59311	-5.25639	-0.82268
H	0.43109	-5.55394	-2.20361
H	1.47991	-5.18694	-0.80731
H	2.75636	-1.40015	-0.07834
H	4.94632	-1.43412	-1.19224
H	0.93832	0.48347	-3.28747
H	3.02441	0.52783	-4.58762
H	7.46699	-0.93395	-3.77232
H	6.86380	-0.62409	-2.12238
H	6.44247	-2.15545	-2.95831
H	6.53828	0.03535	-5.41547
H	4.87694	-0.54088	-5.74978
H	5.15277	1.12212	-5.13349
H	-0.95419	-0.79355	-3.49065
C	-3.37394	-1.81376	-1.08409
C	-4.22666	-2.44740	-2.20814
H	-3.12438	-2.56332	-0.30741
H	-3.96996	-1.03888	-0.55759
C	-5.52410	-3.08606	-1.67824
H	-4.47340	-1.67534	-2.96475
H	-3.62170	-3.21290	-2.73282
C	-6.38039	-3.71729	-2.78877
H	-5.26785	-3.85397	-0.92197
H	-6.11827	-2.31729	-1.14393
H	-7.30446	-4.16687	-2.38237
H	-6.67701	-2.96349	-3.54198
H	-5.82480	-4.51393	-3.31778

[5-6]‡

129

XYZ

C	2.11978	1.02550	-2.26004
N	1.78741	0.02871	-1.39135
C	2.75590	-0.90318	-1.18361
C	4.01612	-0.88334	-1.77792
C	4.36486	0.16330	-2.67669
C	3.34828	1.13341	-2.90710
Ru	-0.27877	-0.15171	-0.66804
C	-2.25678	0.22075	-1.62659
C	-1.29813	0.76722	-2.30683
N	5.60235	0.23403	-3.28159
C	6.56151	-0.85229	-3.09976
P	-0.18229	1.92159	0.46278
C	-1.05706	3.37549	-0.36899
C	-2.59246	3.38328	-0.23734
C	-3.23970	4.55177	-1.00850
C	-4.77051	4.58953	-0.86568
N	-0.39486	-1.94902	-1.88634
C	-1.39621	-2.86405	-1.81965
C	-1.44931	-3.69420	-3.12760
C	-0.12816	-3.35027	-3.82846
C	0.20936	-1.96222	-3.22503
O	-2.20248	-3.05301	-0.87245
P	0.22561	-1.49789	1.26763
C	1.79268	-1.02691	2.25020
C	1.83382	-1.19116	3.78310
C	3.20904	-0.81933	4.37603
C	3.25910	-0.94551	5.90820
C	1.58136	2.59197	0.74094
C	1.90046	4.07650	0.47385
C	3.38751	4.40448	0.72351
C	3.73426	5.87746	0.44823
C	-0.85575	2.07485	2.21935
C	-0.76821	3.43811	2.93325
C	-1.37197	3.40185	4.35335
C	-1.27907	4.75359	5.08102
C	0.50193	-3.32648	0.93798
C	0.91100	-4.24206	2.10623
C	1.00549	-5.72328	1.68194
C	1.41921	-6.65587	2.83282
C	-1.10017	-1.56415	2.60646
C	-2.35073	-2.38987	2.24441
C	-3.52377	-2.11433	3.20623
C	-4.75495	-2.98700	2.90929
C	5.86254	1.26362	-4.28403
H	-1.87491	-0.37294	-0.22924
H	2.24440	1.96843	0.11773
H	1.83497	2.34705	1.79194
H	1.64866	4.33439	-0.57404
H	1.27565	4.73245	1.11104
H	3.64607	4.15135	1.77140
H	4.01387	3.74804	0.08677
H	4.80435	6.08245	0.63232
H	3.51592	6.15070	-0.60110
H	3.14752	6.55481	1.09624
H	-0.64983	4.32660	0.02150
H	-0.78640	3.32031	-1.44079
H	-2.99558	2.42671	-0.61473
H	-2.88350	3.45449	0.82944
H	-2.80952	5.51072	-0.65426
H	-2.96924	4.47067	-2.08038
H	-5.20972	5.42999	-1.43298
H	-5.22829	3.65557	-1.24065
H	-5.07133	4.70573	0.19236
H	-0.31072	1.31639	2.81377
H	-1.90711	1.72944	2.18212
H	0.28971	3.76017	3.00287
H	-1.28969	4.21579	2.34164
H	-2.43221	3.08556	4.28961
H	-0.85588	2.62247	4.94906
H	-1.71871	4.69846	6.09311
H	-0.22736	5.07838	5.18973
H	-1.81591	5.54486	4.52527
H	-0.45253	-3.65650	0.48503
H	1.24886	-3.39113	0.12275
H	1.88970	-3.92704	2.52227
H	0.17966	-4.15555	2.93478
H	0.02718	-6.04394	1.27358
H	1.73011	-5.81719	0.84799
H	1.47743	-7.70819	2.50042
H	2.41001	-6.37755	3.23906
H	0.69348	-6.60822	3.66615
H	2.61954	-1.61401	1.80190
H	2.00710	0.02727	1.99442
H	1.05842	-0.55249	4.25127
H	1.58761	-2.23172	4.06889
H	3.98704	-1.46737	3.92424
H	3.46618	0.21768	4.07951
H	4.25448	-0.67478	6.30444
H	2.51548	-0.28264	6.38861
H	3.03946	-1.98001	6.23149
H	-1.39606	-0.51783	2.79967
H	-0.65740	-1.94824	3.54476
H	-2.64792	-2.19663	1.19771
H	-2.10248	-3.46908	2.27903
H	-3.19771	-2.27761	4.25454
H	-3.80477	-1.04319	3.13847
H	-5.59052	-2.75872	3.59627
H	-5.11130	-2.83053	1.87495
H	-4.51584	-4.06147	3.01565
H	-0.22064	-1.14167	-3.84577
H	1.29932	-1.78877	-3.18043
H	-1.59778	-4.76563	-2.90843
H	-2.32893	-3.35655	-3.71216

H	-0.18619	-3.33181	-4.93209
H	0.65402	-4.08047	-3.54587
H	2.47994	-1.72454	-0.51880
H	4.71292	-1.69220	-1.54512
H	1.33133	1.76034	-2.44292
H	3.50089	1.96824	-3.59551
H	7.49022	-0.60614	-3.63596
H	6.81343	-0.98635	-2.03042
H	6.18056	-1.81853	-3.48972
H	6.90331	1.17458	-4.62974
H	5.19462	1.17113	-5.16506
H	5.73348	2.27669	-3.85736
H	-1.17799	1.29698	-3.25454
C	-3.69746	-0.18151	-1.57530
C	-4.45718	0.19235	-2.86510
H	-3.73887	-1.27522	-1.39816
H	-4.19478	0.28853	-0.69917
C	-5.93621	-0.23562	-2.82827
H	-4.38934	1.28656	-3.03136
H	-3.95269	-0.28452	-3.72831
C	-6.69565	0.11632	-4.11858
H	-5.99370	-1.32752	-2.64918
H	-6.43576	0.24366	-1.96211
H	-7.75272	-0.20154	-4.06572
H	-6.68196	1.20627	-4.30694
H	-6.24023	-0.37827	-4.99672

6

129

XYZ

C	1.40858	-1.74495	1.68701
N	0.67045	-1.75688	0.53965
C	0.35933	-3.00215	0.06677
C	0.71142	-4.19280	0.69140
C	1.45133	-4.17692	1.90911
C	1.80585	-2.88637	2.38393
Ru	0.22754	-0.12806	-0.68416
C	2.91742	0.40500	-2.19719
C	2.25896	-0.14002	-1.14505
N	1.79903	-5.33783	2.57920
C	1.58060	-6.63054	1.93668
P	0.56901	1.53332	0.85897
C	2.32755	2.00801	1.37155
C	3.11566	2.88043	0.37348
C	4.56469	3.12540	0.84171
C	5.35766	4.02717	-0.11933
N	-0.08384	-1.05479	-2.70124
C	-0.11098	0.15745	-3.23531
C	-0.02721	0.16391	-4.75800
C	-0.20588	-1.34052	-5.07953
C	0.16072	-2.05570	-3.73859
O	-0.12255	1.18419	-2.44520
P	-2.19738	-0.38972	-0.29927
C	-2.68743	-1.23403	1.33038
C	-3.98902	-0.82410	2.04808
C	-4.22897	-1.63461	3.33872
C	-5.51265	-1.22290	4.07924
C	-0.29330	1.33999	2.54310
C	0.21663	2.07737	3.79953
C	-0.72809	1.89895	5.00669
C	-0.21328	2.58959	6.28105
C	-0.10033	3.16346	0.20389
C	-0.10916	4.40549	1.11710
C	-0.53378	5.68324	0.36250
C	-0.58237	6.92829	1.26402
C	-3.02738	-1.52980	-1.55490
C	-4.53967	-1.80167	-1.45794
C	-5.04369	-2.74235	-2.57334
C	-6.55467	-3.01847	-2.49232
C	-3.34548	1.10585	-0.36746
C	-3.36194	1.79672	-1.74671
C	-4.14710	3.12343	-1.73206
C	-4.22216	3.79093	-3.11561
C	2.71936	-5.26068	3.70974
H	2.35875	1.04763	-2.89792
H	-0.29691	0.25214	2.74782
H	-1.35250	1.60945	2.35568
H	1.22026	1.69646	4.07449
H	0.34340	3.15814	3.59832
H	-1.72914	2.29768	4.74627
H	-0.87142	0.81696	5.20165
H	-0.91283	2.45390	7.12552
H	0.76735	2.18147	6.58935
H	-0.08563	3.67655	6.12262
H	2.29314	2.51563	2.35388
H	2.87282	1.05517	1.51828
H	3.12162	2.38164	-0.61224
H	2.61317	3.85908	0.24313
H	4.55741	3.57832	1.85461
H	5.08155	2.15005	0.94401
H	6.39381	4.18341	0.23218
H	5.40818	3.58121	-1.12979
H	4.88316	5.02153	-0.21800
H	-1.12591	2.95104	-0.15077
H	0.48618	3.35241	-0.71487
H	-0.80396	4.24425	1.96525
H	0.89179	4.56883	1.56338
H	0.16801	5.85686	-0.47718
H	-1.52774	5.51876	-0.09987
H	-0.89217	7.82550	0.69843
H	-1.29892	6.79253	2.09577
H	0.40802	7.13850	1.70934
H	-2.76791	-1.11536	-2.54725
H	-2.47006	-2.48530	-1.49105

H	-4.78593	-2.25102	-0.47524
H	-5.10341	-0.84951	-1.51398
H	-4.79888	-2.29974	-3.55951
H	-4.48719	-3.69981	-2.52142
H	-6.88732	-3.69335	-3.30156
H	-6.82424	-3.49146	-1.52943
H	-7.13668	-2.08179	-2.57662
H	-2.70574	-2.32122	1.11526
H	-1.83401	-1.08184	2.01506
H	-3.95260	0.25384	2.30266
H	-4.86059	-0.95008	1.37679
H	-4.27404	-2.71354	3.08771
H	-3.35654	-1.51412	4.01189
H	-5.65837	-1.81849	4.99848
H	-5.48076	-0.15690	4.37202
H	-6.40541	-1.36576	3.44226
H	-2.98568	1.81982	0.39907
H	-4.37373	0.82370	-0.07000
H	-2.32312	1.96942	-2.08838
H	-3.82179	1.11634	-2.49237
H	-5.17248	2.94395	-1.34889
H	-3.66981	3.81924	-1.01195
H	-4.78253	4.74276	-3.07722
H	-3.21152	4.00946	-3.50657
H	-4.72751	3.13396	-3.84813
H	1.22743	-2.36572	-3.72549
H	-0.44783	-2.96827	-3.57972
H	-0.78474	0.81986	-5.22241
H	0.96719	0.54478	-5.06335
H	0.41650	-1.68365	-5.92409
H	-1.26107	-1.54883	-5.33498
H	-0.18683	-3.00480	-0.88050
H	0.41662	-5.13074	0.21429
H	1.70159	-0.75697	2.04945
H	2.40346	-2.75421	3.28941
H	1.89498	-7.43047	2.62430
H	0.50808	-6.78316	1.71318
H	2.15085	-6.74000	0.99002
H	2.86049	-6.26875	4.12859
H	3.71412	-4.85788	3.42397
H	2.30533	-4.61946	4.51052
H	2.87295	-0.75567	-0.45330
C	4.39126	0.25515	-2.55470
C	5.22632	-0.68337	-1.66790
H	4.47189	-0.09357	-3.60880
H	4.87420	1.25967	-2.56020
C	6.69527	-0.79589	-2.11808
H	5.19235	-0.32984	-0.61725
H	4.76344	-1.69104	-1.66413
C	7.52938	-1.74065	-1.23611
H	6.72996	-1.14692	-3.16947
H	7.15737	0.21216	-2.11881
H	8.57818	-1.80146	-1.58019
H	7.54101	-1.39809	-0.18423
H	7.11303	-2.76558	-1.24564

7a

148

XYZ

C	-2.60960	1.75342	0.39312
N	-2.18299	0.90112	-0.58197
C	-3.14765	0.51521	-1.46761
C	-4.47017	0.95155	-1.43054
C	-4.90767	1.84932	-0.41870
C	-3.90893	2.24012	0.51705
Ru	-0.16395	0.11586	-0.64331
C	-1.09441	-1.63762	-3.10512
C	-0.72455	-1.52891	-1.80086
N	-6.20969	2.30261	-0.34148
C	-7.16150	1.95080	-1.39096
P	-0.87133	-1.24480	1.16062
C	0.17115	-2.77450	1.56204
C	1.17445	-2.65297	2.72755
C	2.11072	-3.87508	2.81755
C	3.07409	-3.80632	4.01426
N	0.19742	1.24636	-2.52169
C	1.28923	1.22076	-3.33471
C	0.97696	1.93245	-4.67742
C	-0.27060	2.75916	-4.34905
C	-0.91000	1.93404	-3.20343
O	2.41733	0.72207	-3.09729
P	0.86042	2.25909	0.27584
C	-0.00941	3.80127	-0.40342
C	0.47172	5.19312	0.04980
C	-0.14459	6.32998	-0.79304
C	0.30278	7.72930	-0.33752
C	-2.57896	-1.97721	0.75456
C	-2.96176	-3.39302	1.22781
C	-4.44396	-3.71678	0.94415
C	-4.84062	-5.14222	1.36418
C	-1.22200	-0.52902	2.87710
C	-2.04417	-1.34455	3.89543
C	-2.17045	-0.62947	5.25741
C	-3.00704	-1.41936	6.27828
C	2.57799	2.58159	-0.47748
C	3.81300	2.71784	0.43158
C	5.07637	3.07493	-0.38006
C	6.33862	3.19180	0.49088
C	1.01937	2.82124	2.08809
C	1.74721	1.88858	3.07267
C	1.57572	2.32241	4.54319
C	2.34845	1.43068	5.52971
C	-6.57069	3.30532	0.65515
H	-1.19035	-0.72716	-3.71876

H	-2.64931	-1.93723	-0.34641
H	-3.31266	-1.24054	1.13860
H	-2.33119	-4.14000	0.70613
H	-2.76774	-3.52403	2.31006
H	-5.08424	-2.98139	1.47226
H	-4.64496	-3.57720	-0.13662
H	-5.90641	-5.34470	1.15358
H	-4.24291	-5.89898	0.82297
H	-4.67679	-5.30112	2.44652
H	-0.49762	-3.63108	1.76111
H	0.71623	-3.00665	0.62795
H	1.79303	-1.74317	2.61573
H	0.63015	-2.54487	3.68648
H	1.50216	-4.79951	2.88262
H	2.69000	-3.94985	1.87650
H	3.73540	-4.69087	4.05319
H	3.71785	-2.90856	3.95680
H	2.52302	-3.75847	4.97221
H	-1.72843	0.43804	2.69373
H	-0.23889	-0.27419	3.31430
H	-3.05913	-1.53335	3.49538
H	-1.58447	-2.34028	4.05371
H	-1.15740	-0.44707	5.66873
H	-2.62186	0.37125	5.10185
H	-3.07954	-0.88463	7.24252
H	-4.03608	-1.58601	5.90876
H	-2.56123	-2.41150	6.47827
H	2.72860	1.77068	-1.21577
H	2.48109	3.51152	-1.07118
H	3.65503	3.49352	1.20871
H	4.00136	1.76714	0.96698
H	5.22765	2.30714	-1.16297
H	4.90606	4.02991	-0.91671
H	7.22618	3.45154	-0.11437
H	6.22227	3.97251	1.26628
H	6.55562	2.23916	1.01026
H	0.12002	3.69661	-1.49591
H	-1.09458	3.70580	-0.21432
H	0.21465	5.36063	1.11444
H	1.57665	5.26055	-0.01523
H	0.12715	6.17984	-1.85684
H	-1.25016	6.25984	-0.74789
H	-0.14938	8.52213	-0.96056
H	0.01189	7.92114	0.71241
H	1.40149	7.83810	-0.40265
H	-0.02636	2.96164	2.43512
H	1.48918	3.82286	2.12348
H	1.37227	0.85329	2.95645
H	2.82624	1.85630	2.83202
H	1.90865	3.37389	4.65442
H	0.49713	2.31360	4.80006
H	2.20203	1.76005	6.57428
H	2.01791	0.37738	5.46098
H	3.43436	1.45191	5.32105
H	-1.64178	1.20776	-3.62582
H	-1.47925	2.56905	-2.50047
H	1.84861	2.51383	-5.02320
H	0.76892	1.15925	-5.44466
H	-0.96177	2.91347	-5.19792
H	0.02502	3.75871	-3.97380
H	-2.81800	-0.20184	-2.22397
H	-5.15488	0.57926	-2.19697
H	-1.84498	2.04664	1.11759
H	-4.13035	2.92442	1.33990
H	-8.14012	2.39467	-1.15384
H	-6.84336	2.32211	-2.38692
H	-7.29433	0.85385	-1.45952
H	-7.64395	3.53163	0.56708
H	-6.38663	2.93451	1.68223
H	-6.00499	4.25106	0.52380
H	-0.60400	-2.51504	-1.30054
C	-1.36167	-2.91399	-3.89591
C	-1.21047	-4.24595	-3.14221
H	-2.38664	-2.86864	-4.32985
H	-0.68870	-2.93290	-4.78270
C	-1.49699	-5.47602	-4.02446
H	-0.18438	-4.32267	-2.72916
H	-1.89173	-4.25605	-2.26685
C	-1.34369	-6.80961	-3.27348
H	-2.52364	-5.40026	-4.43701
H	-0.81619	-5.46487	-4.89969
H	-1.55376	-7.67408	-3.92958
H	-0.31721	-6.92943	-2.87900
H	-2.03707	-6.86539	-2.41333
C	2.23587	-1.52689	-1.72985
C	3.46835	-2.17584	-1.77211
C	4.38610	-2.05426	-0.69593
C	3.94341	-1.25692	0.39490
C	2.67646	-0.67384	0.35806
N	1.79544	-0.79145	-0.66776
H	1.53410	-1.61517	-2.55761
H	3.71766	-2.73683	-2.67570
H	4.57106	-1.07919	1.27151
H	2.33338	-0.06613	1.19864
N	5.62422	-2.67469	-0.70295
C	6.09924	-3.30731	-1.93074
H	6.19012	-2.58873	-2.77185
H	7.08807	-3.75433	-1.74498
H	5.41641	-4.11890	-2.24435
C	6.59058	-2.35707	0.34297
H	6.18798	-2.61125	1.34167
H	7.50020	-2.95733	0.18710
H	6.87698	-1.28385	0.34964

[7a-8a]‡

148

XYZ

C	-3.61692	1.24098	-0.18307
N	-2.78010	0.63698	-1.07100
C	-3.34603	0.29547	-2.26572
C	-4.67616	0.52881	-2.60014
C	-5.55110	1.15810	-1.66822
C	-4.96317	1.51229	-0.42189
Ru	-0.70198	0.05450	-0.78045
C	1.00070	-1.83528	-2.73939
C	0.19659	-1.10406	-1.95831
N	-6.87587	1.40387	-1.95373
C	-7.41929	1.04582	-3.26122
P	-1.45130	-1.87514	0.33096
C	-0.17432	-3.14665	0.83959
C	0.81982	-2.71814	1.93438
C	1.80503	-3.85188	2.29221
C	2.83056	-3.46587	3.37253
N	0.02390	1.65393	-2.09450
C	1.32322	1.89485	-2.39905
C	1.41776	2.79189	-3.65692
C	-0.00585	3.34562	-3.80777
C	-0.85332	2.25381	-3.10478
O	2.34563	1.47878	-1.79151
P	-0.36362	1.75887	0.97335
C	-0.93309	3.46025	0.35451
C	-1.26992	4.58947	1.34702
C	-1.58832	5.91819	0.62923
C	-1.96018	7.05320	1.59831
C	-2.56221	-2.87662	-0.82885
C	-2.66214	-4.40999	-0.69069
C	-3.65961	-5.01793	-1.69916
C	-3.74765	-6.55083	-1.60898
C	-2.52700	-1.67171	1.86197
C	-3.17890	-2.90835	2.51129
C	-3.94606	-2.55912	3.80422
C	-4.61141	-3.78125	4.45936
C	1.46937	1.98763	1.25394
C	1.95568	3.23757	2.01186
C	3.47743	3.20707	2.26715
C	3.99286	4.47269	2.97238
C	-1.02893	1.72686	2.75139
C	-0.25396	0.81338	3.72154
C	-0.95241	0.67165	5.08954
C	-0.14954	-0.17910	6.08807
C	-7.72183	2.07218	-0.96915
H	0.51447	-2.29705	-3.61556
H	-2.18493	-2.63078	-1.83827
H	-3.56996	-2.42004	-0.75526
H	-1.66572	-4.86227	-0.85810
H	-2.96558	-4.70005	0.33331
H	-4.66313	-4.57690	-1.53104
H	-3.36294	-4.72248	-2.72553
H	-4.46900	-6.95823	-2.34022
H	-2.76637	-7.01934	-1.80951
H	-4.07144	-6.87495	-0.60230
H	-0.69489	-4.07284	1.15044
H	0.37620	-3.38081	-0.09215
H	1.37530	-1.82191	1.59742
H	0.27285	-2.42169	2.85295
H	1.22111	-4.73357	2.62795
H	2.33876	-4.16962	1.37447
H	3.41401	-4.34892	3.69146
H	3.55436	-2.71458	3.00583
H	2.32909	-3.05861	4.27146
H	-3.31192	-0.94614	1.56974
H	-1.89611	-1.14913	2.60487
H	-3.87930	-3.38375	1.79792
H	-2.40762	-3.66852	2.74567
H	-3.24825	-2.08600	4.52386
H	-4.71572	-1.79439	3.57562
H	-5.15040	-3.50147	5.38236
H	-5.34045	-4.25482	3.77562
H	-3.86138	-4.54778	4.72881
H	1.80325	1.06893	1.77454
H	1.91624	1.93170	0.24162
H	1.71020	4.14607	1.42653
H	1.43075	3.34467	2.98394
H	3.72774	2.31388	2.87272
H	4.00319	3.07169	1.30260
H	5.08292	4.42148	3.14588
H	3.79181	5.37820	2.36924
H	3.50427	4.61317	3.95523
H	-0.13038	3.77672	-0.33710
H	-1.81035	3.27013	-0.29191
H	-2.14359	4.30382	1.96746
H	-0.43262	4.75453	2.05183
H	-0.71243	6.22019	0.02122
H	-2.41687	5.75504	-0.08921
H	-2.17873	7.99167	1.05746
H	-2.85490	6.79403	2.19503
H	-1.13677	7.25925	2.30718
H	-2.08771	1.40150	2.70690
H	-1.03600	2.75487	3.15949
H	-0.10945	-0.19098	3.27757
H	0.76312	1.22078	3.87858
H	-1.12707	1.67998	5.51609
H	-1.95695	0.22480	4.94173
H	-0.67552	-0.27165	7.05538
H	0.02046	-1.20000	5.69851
H	0.84201	0.26794	6.28616
H	-1.18658	1.48663	-3.84187
H	-1.76823	2.66985	-2.64156

H	2.20349	3.55756	-3.53729
H	1.70784	2.15330	-4.51544
H	-0.32062	3.52246	-4.85206
H	-0.10232	4.30280	-3.26094
H	-2.66770	-0.19242	-2.97042
H	-5.02401	0.21575	-3.58748
H	-3.16939	1.51276	0.77655
H	-5.54210	2.00195	0.36491
H	-8.48449	1.31829	-3.29577
H	-6.90067	1.57978	-4.08241
H	-7.33850	-0.04262	-3.45131
H	-8.73886	2.17315	-1.37598
H	-7.78639	1.49168	-0.02760
H	-7.34674	3.08636	-0.72484
H	0.92119	-0.50005	-0.76947
C	2.49225	-2.06933	-2.58566
C	2.87117	-3.56046	-2.44113
H	3.01711	-1.65522	-3.47260
H	2.86667	-1.49641	-1.71809
C	4.39251	-3.79336	-2.37247
H	2.39338	-3.97053	-1.52777
H	2.44769	-4.13523	-3.29154
C	4.76932	-5.27556	-2.20979
H	4.86283	-3.38959	-3.29189
H	4.81217	-3.20761	-1.53175
H	5.86513	-5.41268	-2.16624
H	4.34368	-5.69550	-1.27922
H	4.38814	-5.88219	-3.05312
C	4.50512	-0.63845	0.93681
C	4.81409	0.16916	-0.15901
C	6.17405	0.29852	-0.55967
C	7.12186	-0.41110	0.23217
C	6.68011	-1.18851	1.30927
N	5.39276	-1.32785	1.69189
H	3.45107	-0.74032	1.23134
H	3.99602	0.66096	-0.69350
H	8.19450	-0.35721	0.02695
H	7.42090	-1.73281	1.91326
N	6.55239	1.04845	-1.66534
C	5.66386	2.07472	-2.21786
H	5.73994	3.03386	-1.65951
H	5.95438	2.26438	-3.26624
H	4.60800	1.75789	-2.21038
C	7.97248	1.22910	-1.94258
H	8.48242	0.25223	-2.02227
H	8.08060	1.74524	-2.91044
H	8.49658	1.83179	-1.16780

8a

129

XYZ

C	1.15218	2.63188	0.35656
N	0.89803	1.85973	-0.72750
C	1.31465	2.35576	-1.92112
C	1.95135	3.58406	-2.07574
C	2.21865	4.39800	-0.93933
C	1.79419	3.86823	0.31131
Ru	-0.14556	-0.15219	-0.73826
C	-2.24015	-0.01868	-3.12921
C	-1.35045	-0.03166	-2.13153
N	2.84655	5.62227	-1.04382
C	3.39452	6.04710	-2.32995
P	-1.98952	0.67870	0.50357
C	-3.53808	-0.37611	0.41538
C	-3.41599	-1.73304	1.13204
C	-4.68065	-2.60327	0.98009
C	-4.56770	-3.95501	1.70441
N	1.32268	-0.76028	-2.21707
C	2.66213	-0.57857	-2.10590
C	3.37579	-1.08810	-3.38281
C	2.23094	-1.25592	-4.39176
C	1.00595	-1.46999	-3.46604
O	3.28471	-0.11066	-1.11961
P	1.35855	-1.11841	1.12050
C	0.45636	-1.41813	2.75659
C	1.17624	-2.18987	3.88128
C	0.35240	-2.23120	5.18600
C	1.05335	-3.00233	6.31722
C	-2.53251	2.33827	-0.23500
C	-4.03469	2.65018	-0.38473
C	-4.27823	4.04372	-1.00133
C	-5.77056	4.36706	-1.18541
C	-1.93489	1.09954	2.34594
C	-3.11192	1.87537	2.96993
C	-2.93323	2.09018	4.48822
C	-4.09285	2.87448	5.12509
C	2.83925	-0.12813	1.75611
C	4.17319	-0.86977	1.95151
C	5.29596	0.07410	2.42814
C	6.64196	-0.64517	2.62052
C	1.92975	-2.89640	0.85975
C	2.56781	-3.31460	-0.47280
C	2.95041	-4.80865	-0.47325
C	3.56456	-5.27070	-1.80522
C	3.21061	6.35467	0.16575
H	-2.35274	0.95373	-3.64056
H	-2.05607	2.37345	-1.22984
H	-2.03870	3.12464	0.37078
H	-4.50577	1.88433	-1.03081
H	-4.55132	2.59515	0.59302
H	-3.80667	4.81512	-0.35935
H	-3.76083	4.10209	-1.97963
H	-5.91622	5.37087	-1.62374
H	-6.25820	3.63474	-1.85533

H	-6.30726	4.34219	-0.21858
H	-4.40144	0.19032	0.81248
H	-3.71798	-0.53484	-0.66398
H	-2.53780	-2.27471	0.72913
H	-3.22246	-1.57808	2.21319
H	-5.55912	-2.04731	1.36566
H	-4.87522	-2.77515	-0.09739
H	-5.48553	-4.55764	1.58092
H	-3.72087	-4.54811	1.31229
H	-4.40217	-3.81549	2.78922
H	-0.99505	1.66298	2.51222
H	-1.80804	0.13941	2.87733
H	-3.22077	2.86238	2.48042
H	-4.06209	1.33328	2.79462
H	-2.83230	1.10535	4.98634
H	-1.97913	2.62470	4.67049
H	-3.93835	3.01297	6.21024
H	-4.19546	3.87746	4.67044
H	-5.05525	2.34721	4.98837
H	2.98757	0.65527	0.99647
H	2.52425	0.34946	2.70714
H	4.06524	-1.69755	2.68103
H	4.46760	-1.31784	0.98500
H	5.41299	0.89013	1.68853
H	4.99426	0.55709	3.38041
H	7.43133	0.05342	2.95321
H	6.56390	-1.44736	3.37832
H	6.98140	-1.11042	1.67673
H	0.18781	-0.41399	3.13251
H	-0.49701	-1.92118	2.50249
H	1.38718	-3.22809	3.56143
H	2.15928	-1.72396	4.09242
H	0.14281	-1.19429	5.51832
H	-0.63447	-2.69160	4.97839
H	0.44218	-3.01560	7.23776
H	1.24426	-4.05205	6.02638
H	2.02824	-2.54441	6.56827
H	1.00456	-3.48811	1.01589
H	2.61009	-3.15515	1.69300
H	1.85757	-3.10795	-1.29255
H	3.46260	-2.69766	-0.67279
H	3.66453	-5.00777	0.35200
H	2.05042	-5.41826	-0.25154
H	3.82944	-6.34360	-1.78032
H	2.85884	-5.11800	-2.64282
H	4.48480	-4.70323	-2.03878
H	0.85399	-2.55725	-3.26564
H	0.06973	-1.09203	-3.90828
H	4.17411	-0.39107	-3.69130
H	3.86178	-2.05613	-3.14480
H	2.37318	-2.08345	-5.11028
H	2.09785	-0.32522	-4.97653
H	1.14182	1.69712	-2.77685
H	2.25257	3.89119	-3.08012
H	0.82945	2.22328	1.31966
H	1.96803	4.40279	1.24824
H	3.85130	7.04159	-2.21504
H	4.16954	5.34864	-2.70668
H	2.59905	6.12620	-3.09513
H	3.67524	7.31083	-0.11853
H	2.31608	6.58259	0.77609
H	3.92849	5.79352	0.79916
H	-0.65583	-1.68886	-0.56061
C	-3.04414	-1.16954	-3.72577
C	-2.86347	-2.53569	-3.04160
H	-4.12275	-0.89970	-3.72443
H	-2.77259	-1.26882	-4.79950
C	-3.65777	-3.65694	-3.73757
H	-1.78822	-2.79726	-3.02012
H	-3.17601	-2.46461	-1.98111
C	-3.48149	-5.02707	-3.06124
H	-4.73426	-3.38999	-3.75716
H	-3.34101	-3.72546	-4.79803
H	-4.06031	-5.81360	-3.57869
H	-2.41990	-5.33654	-3.05934
H	-3.82000	-4.99874	-2.00873

[8a-10]‡

129

XYZ

C	1.10916	2.88669	0.18715
N	0.19355	2.17017	-0.51248
C	-0.67729	2.91186	-1.24887
C	-0.67918	4.30494	-1.30729
C	0.27457	5.05168	-0.56002
C	1.19616	4.27867	0.20080
Ru	-0.01617	-0.17721	-0.47249
C	-2.12136	-0.10827	-2.91100
C	-1.26889	-0.15625	-1.87626
N	0.30384	6.43124	-0.57125
C	-0.60193	7.17004	-1.44620
P	-1.43424	-0.30200	1.33361
C	-2.88302	-1.46577	1.05205
C	-2.49840	-2.94900	0.89968
C	-3.71439	-3.84575	0.59079
C	-3.33747	-5.32888	0.43791
N	0.68212	-0.31484	-2.60965
C	0.78380	-1.47729	-3.36277
C	1.27801	-1.10717	-4.78388
C	1.87147	0.29826	-4.61285
C	1.06467	0.85994	-3.41323
O	0.51111	-2.63078	-3.00583
P	2.17318	-0.67403	0.50956
C	2.65618	-0.01380	2.22171

C	4.12815	0.02568	2.67728
C	4.27149	0.41675	4.16372
C	5.73549	0.48809	4.62978
C	-2.28262	1.33042	1.78720
C	-3.72705	1.33450	2.32895
C	-4.21594	2.75713	2.67323
C	-5.66652	2.78922	3.18375
C	-0.78321	-0.89706	3.00034
C	-1.75690	-1.14061	4.17016
C	-1.04507	-1.69807	5.42120
C	-2.00110	-1.93803	6.60182
C	3.52250	-0.08494	-0.66757
C	4.96114	-0.63766	-0.59454
C	5.83130	-0.13390	-1.76578
C	7.26980	-0.67664	-1.72347
C	2.55720	-2.49530	0.76250
C	2.50444	-3.35657	-0.51253
C	2.55058	-4.86764	-0.20993
C	2.55817	-5.71847	-1.49151
C	1.36400	7.14158	0.13722
H	-1.79312	-0.35480	-3.93010
H	-2.24171	1.93775	0.86442
H	-1.60460	1.83305	2.50664
H	-4.40904	0.89467	1.57511
H	-3.81353	0.69737	3.23012
H	-3.54314	3.19871	3.43589
H	-4.12306	3.40007	1.77500
H	-5.98862	3.81873	3.42357
H	-6.36558	2.38694	2.42712
H	-5.78214	2.18027	4.09974
H	-3.61553	-1.34552	1.87360
H	-3.37005	-1.11514	0.12245
H	-1.74893	-3.04443	0.09201
H	-2.01205	-3.31141	1.82876
H	-4.47401	-3.73440	1.39158
H	-4.19592	-3.48975	-0.34211
H	-4.22223	-5.95007	0.20819
H	-2.60531	-5.46908	-0.37815
H	-2.88282	-5.72369	1.36599
H	-0.02187	-0.15205	3.30104
H	-0.22617	-1.82802	2.77618
H	-2.26668	-0.19548	4.44068
H	-2.55256	-1.84845	3.86567
H	-0.53636	-2.64661	5.15633
H	-0.24394	-0.99591	5.72833
H	-1.46521	-2.33914	7.48088
H	-2.49814	-0.99981	6.91162
H	-2.79339	-2.66139	6.33356
H	3.09117	-0.30855	-1.66255
H	3.53335	1.02136	-0.59425
H	5.44529	-0.35489	0.35767
H	4.94247	-1.74404	-0.61530

H	5.35322	-0.42318	-2.72305
H	5.85164	0.97476	-1.75629
H	7.86839	-0.30300	-2.57382
H	7.78495	-0.37446	-0.79241
H	7.28074	-1.78160	-1.76593
H	2.21577	0.99793	2.31519
H	2.07753	-0.64762	2.92197
H	4.60738	-0.96000	2.51791
H	4.69349	0.75111	2.06037
H	3.77921	1.39585	4.33291
H	3.71843	-0.31500	4.78603
H	5.80690	0.76569	5.69684
H	6.24311	-0.48600	4.50216
H	6.30461	1.23808	4.04939
H	1.78751	-2.84903	1.47738
H	3.53838	-2.60130	1.26807
H	1.59018	-3.12293	-1.08982
H	3.35562	-3.09824	-1.17455
H	3.44253	-5.10736	0.40584
H	1.66945	-5.13653	0.40734
H	2.52131	-6.79935	-1.26250
H	1.69297	-5.46889	-2.13109
H	3.47255	-5.53271	-2.08594
H	0.15790	1.39749	-3.76621
H	1.65003	1.56976	-2.80262
H	1.97872	-1.86974	-5.16201
H	0.39960	-1.10315	-5.46130
H	1.79481	0.93793	-5.51031
H	2.94240	0.22665	-4.34363
H	-1.39858	2.33067	-1.83472
H	-1.42168	4.79973	-1.93890
H	1.81773	2.29673	0.77602
H	1.98055	4.74948	0.79894
H	-0.44390	8.24911	-1.29994
H	-0.43295	6.93682	-2.51755
H	-1.65971	6.94665	-1.20797
H	1.20505	8.22525	0.03170
H	1.35420	6.90139	1.21800
H	2.37075	6.89874	-0.26109
H	-0.16562	-1.77229	-0.49314
C	-3.60176	0.18740	-2.71942
C	-4.51401	-1.00917	-3.07519
H	-3.78901	0.49292	-1.67102
H	-3.89545	1.05486	-3.35080
C	-6.01535	-0.69101	-2.94208
H	-4.29632	-1.33710	-4.11216
H	-4.24989	-1.86588	-2.42395
C	-6.91954	-1.88271	-3.29986
H	-6.22915	-0.36327	-1.90434
H	-6.26549	0.17213	-3.59214
H	-7.98966	-1.62694	-3.19495
H	-6.75323	-2.21059	-4.34290

H	-6.71523	-2.74920	-2.64394

7b

148

XYZ

C	2.52227	1.82899	-1.00094
N	1.63563	1.58893	0.00449
C	1.91735	2.22433	1.17269
C	3.01556	3.05690	1.39217
C	3.94347	3.29434	0.33957
C	3.65197	2.63784	-0.88973
Ru	-0.06861	-0.00113	-0.36836
C	-1.48659	-1.43233	-0.79865
C	-2.30921	-1.59187	-1.86958
N	5.04631	4.10922	0.49828
C	5.35966	4.65622	1.81467
N	-1.61401	1.63224	-0.30435
C	-1.32156	2.90753	0.05685
C	-2.20030	3.98473	-0.05469
C	-3.50587	3.78393	-0.58446
C	-3.81463	2.44407	-0.95162
C	-2.86610	1.43500	-0.79529
N	-4.40935	4.81615	-0.73260
C	-5.68249	4.57155	-1.40557
P	1.57454	-1.70083	-0.58981
C	3.23351	-1.31728	0.24707
C	4.37430	-2.35252	0.17008
C	5.69207	-1.80670	0.76164
C	6.84243	-2.82670	0.71675
N	0.05494	0.63426	-2.51803
C	-0.11948	2.07299	-2.81316
C	0.34066	2.27124	-4.27424
C	-0.09514	0.94198	-4.89476
C	-0.00628	-0.05431	-3.70297
O	-0.01062	-1.28966	-3.90070
P	-0.44187	-0.38029	1.94275
C	-1.35141	0.99162	2.89160
C	-2.88698	1.02047	2.75777
C	-3.50871	2.25983	3.43459
C	-5.04254	2.28939	3.32560
C	2.18543	-2.25672	-2.27112
C	3.08050	-1.28397	-3.05766
C	3.41262	-1.83412	-4.45959
C	4.32458	-0.90065	-5.27295
C	1.19486	-3.40694	0.13132
C	0.35082	-4.34886	-0.75416
C	-0.06298	-5.63387	-0.00857
C	-0.85882	-6.60698	-0.89446
C	-3.99868	6.18702	-0.44555
C	1.08707	-0.63697	3.04980
C	1.10115	-0.22229	4.53584
C	2.39622	-0.66534	5.24906
C	2.45411	-0.22286	6.72104
C	-1.50797	-1.85931	2.44859
C	-1.70285	-2.19826	3.93931
C	-2.74317	-3.32043	4.14609
C	-2.94045	-3.69544	5.62448
C	6.02224	4.22786	-0.58180
H	1.91060	-0.11058	2.53640
H	1.32457	-1.71673	2.96174
H	1.01166	0.87959	4.61865
H	0.23251	-0.64280	5.07564
H	2.48488	-1.76864	5.18940
H	3.27088	-0.25910	4.70250
H	3.38926	-0.55548	7.20649
H	2.40280	0.87845	6.81094
H	1.60946	-0.64265	7.29843
H	-1.09002	0.91217	3.96304
H	-0.94082	1.95761	2.54047
H	-3.18039	1.00415	1.69354
H	-3.32041	0.11031	3.21584
H	-3.20968	2.28781	4.50225
H	-3.08734	3.17266	2.96863
H	-5.46707	3.18945	3.80653
H	-5.36123	2.28931	2.26689
H	-5.49647	1.40495	3.81037
H	-1.08765	-2.74137	1.93128
H	-2.48977	-1.68905	1.96776
H	-0.74070	-2.52028	4.38369
H	-2.02524	-1.30446	4.51003
H	-3.71084	-3.00134	3.71006
H	-2.43102	-4.21603	3.57291
H	-3.69402	-4.49498	5.74111
H	-1.99738	-4.05624	6.07597
H	-3.28106	-2.82507	6.21618
H	2.71564	-3.21997	-2.12654
H	1.26476	-2.42272	-2.85836
H	2.54985	-0.32168	-3.17498
H	4.02353	-1.07140	-2.51168
H	3.89400	-2.82891	-4.36195
H	2.45770	-1.99328	-4.99449
H	4.53815	-1.31384	-6.27578
H	3.85362	0.09034	-5.41476
H	5.29479	-0.73895	-4.76469
H	3.57281	-0.36936	-0.21076
H	3.02603	-1.08058	1.30383
H	4.09898	-3.27494	0.71828
H	4.55257	-2.65837	-0.87884
H	5.98426	-0.89135	0.20879
H	5.51766	-1.48855	1.80935
H	7.77228	-2.40918	1.14413
H	6.59082	-3.73871	1.28967
H	7.06062	-3.13734	-0.32194
H	0.68831	-3.25126	1.10175

H	2.15943	-3.90056	0.35966
H	-0.54773	-3.82075	-1.11958
H	0.93077	-4.62701	-1.65453
H	0.84118	-6.14257	0.38502
H	-0.67090	-5.36214	0.87893
H	-1.15556	-7.51420	-0.33698
H	-1.77855	-6.12982	-1.27951
H	-0.26217	-6.92895	-1.76812
H	-1.18595	2.37669	-2.72430
H	0.44533	2.70230	-2.10110
H	0.51624	0.58560	-5.74103
H	-1.14820	0.98089	-5.24148
H	-0.09794	3.17201	-4.74286
H	1.44361	2.37089	-4.31596
H	-3.09003	0.40013	-1.07922
H	-4.78726	2.17719	-1.37323
H	-0.30772	3.07759	0.42758
H	-1.85204	4.97491	0.25016
H	-6.26034	5.50759	-1.43501
H	-5.54309	4.21566	-2.44680
H	-6.28425	3.81837	-0.86180
H	-4.85415	6.86050	-0.60540
H	-3.67606	6.29488	0.60790
H	-3.16591	6.52250	-1.09794
H	2.28583	1.31775	-1.94037
H	4.30121	2.74587	-1.76229
H	1.20854	2.04919	1.98947
H	3.14175	3.50810	2.37975
H	6.80955	4.93482	-0.27972
H	6.50113	3.25692	-0.82461
H	5.55120	4.61923	-1.50353
H	6.26379	5.27889	1.74029
H	4.53790	5.29715	2.18861
H	5.54691	3.86189	2.56672
H	-1.61220	-2.23234	-0.04386
C	-3.29949	-2.72687	-2.04776
H	-2.25088	-0.89930	-2.72105
C	-4.76287	-2.25742	-2.22697
H	-3.02140	-3.32711	-2.94272
H	-3.24402	-3.41503	-1.17809
C	-5.75952	-3.40463	-2.47901
H	-5.07405	-1.69100	-1.32439
H	-4.81166	-1.53993	-3.07262
C	-7.20991	-2.92440	-2.65812
H	-5.44323	-3.96991	-3.37885
H	-5.71003	-4.12286	-1.63543
H	-7.90105	-3.76883	-2.83572
H	-7.56565	-2.38378	-1.76061
H	-7.29803	-2.23349	-3.51765

[7b-8b]‡

148

XYZ

C	-6.12139	-1.90356	0.21321
N	-5.63551	-1.29389	1.31929
C	-6.22836	-0.12076	1.63973
C	-7.27109	0.47116	0.92497
C	-7.77939	-0.17755	-0.23688
C	-7.15771	-1.41177	-0.58230
Ru	1.54036	-0.09721	-0.18119
C	3.32978	-0.61698	-0.75533
C	4.22055	-1.21348	-1.58997
N	-8.81038	0.35509	-0.98119
C	-9.36872	1.65679	-0.62640
N	0.04580	-0.94326	1.30069
C	-1.30273	-0.97813	1.12342
C	-2.19325	-1.69563	1.91907
C	-1.70327	-2.45827	3.01881
C	-0.28949	-2.43437	3.19800
C	0.51256	-1.68104	2.34223
N	-2.53368	-3.16327	3.87074
C	-1.94015	-4.01867	4.89548
P	0.25414	1.24087	-1.61238
C	-1.04650	2.41519	-0.86847
C	-1.94504	3.23665	-1.81527
C	-2.86764	4.21338	-1.05561
C	-3.77697	5.03151	-1.98851
N	1.08166	-1.84256	-1.38616
C	0.89714	-3.13312	-0.70349
C	0.56998	-4.15805	-1.81952
C	1.29847	-3.56223	-3.02966
C	1.39752	-2.05359	-2.69455
O	1.73967	-1.20534	-3.55562
P	2.10513	1.61277	1.40832
C	2.93657	0.95513	2.97110
C	4.24653	0.17882	2.75093
C	4.79263	-0.44928	4.04929
C	6.11351	-1.20753	3.83658
C	-0.73754	0.47541	-3.00986
C	-1.87816	-0.46576	-2.58765
C	-2.62937	-1.05045	-3.80195
C	-3.74362	-2.03392	-3.40639
C	1.24590	2.51172	-2.59424
C	2.53757	1.99205	-3.24797
C	3.20277	3.04699	-4.15402
C	4.51547	2.54715	-4.78092
C	-3.91426	-3.47691	3.49381
C	0.86065	2.76237	2.23833
C	-0.34272	2.08850	2.91966
C	-1.27906	3.10736	3.60058
C	-2.47063	2.44215	4.30962
C	3.36249	2.88575	0.80469
C	3.94519	3.91322	1.79466
C	4.97210	4.85115	1.12496

C	5.56447	5.88442	2.09790
C	-9.24199	-0.30926	-2.20823
H	0.50721	3.47525	1.46918
H	1.41953	3.36113	2.98443
H	-0.91255	1.50358	2.17559
H	0.00957	1.35866	3.67521
H	-0.70115	3.70940	4.33071
H	-1.65097	3.82271	2.83972
H	-3.13538	3.19239	4.77477
H	-3.07448	1.84791	3.59931
H	-2.12857	1.75607	5.10647
H	3.10432	1.80696	3.66025
H	2.19035	0.30048	3.46115
H	4.08256	-0.60911	1.98895
H	5.01587	0.85525	2.32587
H	4.93662	0.34185	4.81296
H	4.03141	-1.13997	4.46598
H	6.47804	-1.65861	4.77714
H	5.99102	-2.02061	3.09762
H	6.90446	-0.53321	3.45926
H	2.86956	3.40765	-0.03872
H	4.18740	2.30535	0.34581
H	3.13483	4.52711	2.23438
H	4.43501	3.39191	2.64085
H	5.78731	4.24291	0.68517
H	4.48815	5.37352	0.27582
H	6.29509	6.54120	1.59270
H	4.77499	6.52919	2.52702
H	6.08448	5.38990	2.93936
H	-1.13156	1.30491	-3.63098
H	0.00770	-0.08001	-3.60785
H	-1.44893	-1.29090	-1.98916
H	-2.60925	0.06322	-1.93897
H	-3.05960	-0.22291	-4.40230
H	-1.90011	-1.56019	-4.46109
H	-4.26995	-2.43029	-4.29428
H	-3.33062	-2.89600	-2.84999
H	-4.49617	-1.54917	-2.75579
H	-1.68160	1.81140	-0.19156
H	-0.48378	3.10625	-0.21300
H	-1.32326	3.81297	-2.52796
H	-2.56968	2.55772	-2.42735
H	-3.48941	3.64262	-0.33676
H	-2.24790	4.90123	-0.44566
H	-4.42547	5.72276	-1.42003
H	-3.18131	5.63722	-2.69643
H	-4.43210	4.37145	-2.58714
H	1.47786	3.34618	-1.90022
H	0.57388	2.92940	-3.37109
H	3.24320	1.68258	-2.45316
H	2.32419	1.06863	-3.81608
H	2.49816	3.33937	-4.95943
H	3.39863	3.97141	-3.57122
H	4.97562	3.31397	-5.43101
H	5.25213	2.28021	-4.00047
H	4.34175	1.64331	-5.39293
H	1.82932	-3.41828	-0.16289
H	0.09177	-3.08369	0.04852
H	0.79740	-3.71706	-4.00069
H	2.33034	-3.95457	-3.12772
H	0.88191	-5.18489	-1.55512
H	-0.52180	-4.17542	-2.00026
H	1.60058	-1.68842	2.46190
H	0.19820	-3.01308	3.98595
H	-1.67555	-0.40809	0.26828
H	-3.26129	-1.63989	1.68026
H	-2.74520	-4.41561	5.53370
H	-1.37883	-4.87742	4.46905
H	-1.25388	-3.43967	5.53891
H	-4.49374	-3.68082	4.41037
H	-4.39580	-2.63636	2.96837
H	-3.97223	-4.37315	2.83950
H	-5.64190	-2.85347	-0.05768
H	-7.47149	-1.98265	-1.45982
H	-5.83621	0.38299	2.53357
H	-7.67698	1.42647	1.26755
H	-10.06809	0.26262	-2.65625
H	-8.42408	-0.37699	-2.95359
H	-9.60864	-1.33403	-2.00458
H	-10.18279	1.90224	-1.32448
H	-9.78971	1.64908	0.39770
H	-8.61069	2.46426	-0.68185
H	3.39904	0.41647	-0.27651
C	5.30592	-2.17241	-1.14260
H	4.09890	-1.08524	-2.68073
C	5.10121	-3.62638	-1.63487
H	6.28563	-1.81472	-1.53072
H	5.37697	-2.16501	-0.03759
C	6.26459	-4.56400	-1.25902
H	4.15441	-4.01647	-1.21185
H	4.97004	-3.62287	-2.73604
C	6.05756	-6.00943	-1.74239
H	7.20757	-4.16306	-1.68299
H	6.39830	-4.55734	-0.15818
H	6.90703	-6.65839	-1.46108
H	5.14083	-6.44856	-1.30665
H	5.95357	-6.05084	-2.84262

8b

129

XYZ

Ru	0.13239	-0.29822	-0.62748
C	-0.55498	-2.07678	-1.44405
C	-1.76782	-2.59619	-1.77726

N	-1.48846	-0.21066	0.70144
C	-2.23400	0.92290	0.84940
C	-3.37628	1.02304	1.63970
C	-3.84101	-0.10199	2.37556
C	-3.08753	-1.29405	2.19333
C	-1.96335	-1.30232	1.37228
N	-4.94615	-0.04223	3.20740
C	-5.48349	-1.27700	3.77193
P	1.11369	1.93366	-0.08022
C	0.80817	2.72564	1.61542
C	1.21293	4.19319	1.86214
C	0.97427	4.63025	3.32267
C	1.36221	6.09512	3.58716
N	-0.59394	0.61303	-2.54555
C	-1.62759	0.91312	-3.53729
C	-0.85455	1.07204	-4.88577
C	0.42790	0.23153	-4.67391
C	0.50069	0.17615	-3.15238
O	1.45570	-0.32079	-2.43284
P	1.52144	-1.50077	0.77383
C	1.12841	-3.20765	1.50653
C	0.87117	-4.37794	0.53992
C	0.65935	-5.71270	1.28371
C	0.39280	-6.89135	0.33210
C	0.65936	3.35009	-1.24015
C	-0.80996	3.80221	-1.19479
C	-1.11620	4.94628	-2.18309
C	-2.58636	5.39632	-2.14593
C	2.99740	2.10314	-0.17103
C	3.59807	2.00901	-1.58804
C	5.13979	1.97038	-1.57207
C	5.75104	1.93383	-2.98283
C	-5.79089	1.14840	3.19429
C	2.18825	-0.74369	2.37190
C	1.13428	-0.56078	3.48152
C	1.69929	0.12698	4.74125
C	0.65848	0.26644	5.86486
C	3.09675	-1.87487	-0.18522
C	4.23983	-2.64507	0.50476
C	5.42376	-2.91306	-0.44869
C	6.58319	-3.66298	0.22884
H	2.62990	0.23639	2.11035
H	3.01692	-1.37446	2.75105
H	0.27956	0.02355	3.09007
H	0.72550	-1.54756	3.77468
H	2.57190	-0.44810	5.11097
H	2.08790	1.12963	4.46961
H	1.08513	0.76624	6.75320
H	-0.21224	0.86005	5.52993
H	0.28288	-0.72338	6.18498
H	1.98176	-3.45506	2.17057
H	0.25475	-3.08552	2.17480

H	-0.01143	-4.15160	-0.08490
H	1.72502	-4.48700	-0.15845
H	1.54728	-5.93382	1.91065
H	-0.19178	-5.60697	1.98714
H	0.23809	-7.83417	0.88763
H	-0.50804	-6.70965	-0.28245
H	1.24123	-7.04558	-0.36043
H	3.45560	-0.89789	-0.55981
H	2.75623	-2.41362	-1.08986
H	4.61154	-2.07712	1.38078
H	3.87202	-3.61416	0.89765
H	5.06305	-3.49228	-1.32169
H	5.78996	-1.94825	-0.85296
H	7.41564	-3.84109	-0.47555
H	6.98508	-3.08860	1.08444
H	6.25357	-4.64618	0.61364
H	1.33168	4.20848	-1.04026
H	0.88795	2.98380	-2.25747
H	-1.45381	2.93502	-1.43455
H	-1.08291	4.13290	-0.17127
H	-0.45679	5.80944	-1.96112
H	-0.85482	4.61631	-3.20836
H	-2.77996	6.20964	-2.86833
H	-3.26570	4.55927	-2.39355
H	-2.86648	5.76681	-1.14185
H	-0.26902	2.59663	1.83535
H	1.33338	2.07402	2.34005
H	2.28174	4.34607	1.61355
H	0.64353	4.86432	1.18986
H	-0.09329	4.47617	3.57897
H	1.54882	3.96611	3.99922
H	1.17647	6.37904	4.63879
H	2.43401	6.26922	3.37770
H	0.78191	6.78490	2.94626
H	3.41832	1.29291	0.45536
H	3.30175	3.05907	0.30045
H	3.19922	1.11137	-2.09633
H	3.27043	2.88176	-2.18737
H	5.52861	2.85105	-1.02084
H	5.47526	1.08048	-1.00124
H	6.85496	1.89592	-2.94597
H	5.40117	1.04825	-3.54453
H	5.46577	2.83055	-3.56426
H	-2.35852	0.08009	-3.59155
H	-2.19555	1.82683	-3.27390
H	1.33693	0.66300	-5.12879
H	0.32275	-0.80015	-5.06321
H	-1.45253	0.74720	-5.75473
H	-0.58851	2.13402	-5.04080
H	-1.41964	-2.22943	1.18712
H	-3.37825	-2.23198	2.67299
H	-1.88679	1.78317	0.27347

H	-3.89876	1.98241	1.66851
H	-6.33380	-1.03246	4.42676
H	-5.83407	-1.98600	2.99243
H	-4.72188	-1.79063	4.38776
H	-6.58641	1.03209	3.94616
H	-5.20556	2.04773	3.46220
H	-6.26639	1.32638	2.20640
H	0.29049	-2.70847	-1.78404
C	-3.14962	-2.00493	-1.57467
H	-1.78727	-3.57612	-2.29500
C	-4.07359	-2.21098	-2.79627
H	-3.65518	-2.46406	-0.69478
H	-3.06303	-0.92505	-1.34971
C	-5.48817	-1.63342	-2.60499
H	-3.60683	-1.75306	-3.69265
H	-4.14837	-3.29628	-3.01838
C	-6.40661	-1.85048	-3.81968
H	-5.94891	-2.09074	-1.70555
H	-5.41137	-0.54842	-2.38885
H	-7.41293	-1.42399	-3.65315
H	-5.98746	-1.37551	-4.72647
H	-6.53121	-2.92759	-4.03869

[8b-9b]‡

129

XYZ

Ru	0.31912	0.00264	0.31768
C	2.17141	0.04088	0.69847
C	3.41099	-0.13796	1.17898
N	-0.77088	1.59569	-0.70037
C	-2.07640	1.93294	-0.52612
C	-2.67170	3.08113	-1.04601
C	-1.90637	3.99251	-1.82495
C	-0.53472	3.64731	-1.99537
C	-0.03235	2.47878	-1.43089
N	-2.45294	5.13191	-2.37624
C	-1.59168	6.09145	-3.06278
P	-1.61414	-1.33937	0.99877
C	-3.16009	-1.61121	-0.07850
C	-4.35289	-2.38866	0.51398
C	-5.49859	-2.57960	-0.50234
C	-6.69548	-3.35071	0.07975
N	0.16820	1.18160	2.13434
C	0.39359	2.63139	2.06597
C	0.34440	3.12868	3.53376
C	0.84296	1.89922	4.30594
C	0.49228	0.71367	3.37238
O	0.52538	-0.48125	3.75401
P	0.80693	-1.16185	-1.67817
C	1.69469	-0.20450	-3.03960
C	3.08074	0.35709	-2.68145
C	3.68651	1.20696	-3.81721
C	5.07968	1.76188	-3.47678
C	-2.38102	-0.93482	2.66082
C	-3.14365	0.39949	2.73783
C	-3.72870	0.65780	4.14169
C	-4.47858	1.99646	4.24351
C	-1.16403	-3.14385	1.29605
C	0.08716	-3.41127	2.15537
C	0.15879	-4.87314	2.64095
C	1.43269	-5.16680	3.45103
C	-3.83065	5.50064	-2.06043
C	-0.53372	-1.94789	-2.74412
C	-1.54560	-0.96305	-3.35921
C	-2.65987	-1.66949	-4.15778
C	-3.64875	-0.68565	-4.80497
C	1.94673	-2.62901	-1.40170
C	2.53574	-3.37411	-2.61427
C	3.44177	-4.55041	-2.19253
C	4.04306	-5.30488	-3.39014
H	-1.05850	-2.68360	-2.10399
H	-0.04444	-2.52749	-3.55189
H	-1.99771	-0.34391	-2.56106
H	-1.01826	-0.25845	-4.03218
H	-2.20188	-2.30636	-4.94108
H	-3.20933	-2.35926	-3.48583
H	-4.43947	-1.21614	-5.36530
H	-4.14234	-0.05659	-4.04120
H	-3.13359	-0.00843	-5.51147
H	1.76692	-0.86296	-3.92858
H	1.02104	0.62745	-3.32391
H	3.00470	0.95938	-1.75547
H	3.77310	-0.47239	-2.43662
H	3.74554	0.59799	-4.74200
H	2.99908	2.04585	-4.05030
H	5.48480	2.37427	-4.30253
H	5.04712	2.39547	-2.57138
H	5.79791	0.94429	-3.28183
H	1.36998	-3.33046	-0.76717
H	2.75588	-2.23732	-0.75610
H	1.72642	-3.76402	-3.26313
H	3.12563	-2.67617	-3.24081
H	4.25572	-4.16746	-1.54561
H	2.85733	-5.25273	-1.56540
H	4.68596	-6.14018	-3.05930
H	3.25096	-5.72755	-4.03608
H	4.66087	-4.63468	-4.01647
H	-3.04462	-1.77579	2.94599
H	-1.52402	-0.91737	3.36003
H	-2.44525	1.21698	2.47825
H	-3.97302	0.42385	1.99798
H	-4.41123	-0.17232	4.41502
H	-2.90444	0.63432	4.88051
H	-4.88460	2.15839	5.25847

H	-3.80915	2.84578	4.01147
H	-5.32685	2.03687	3.53391
H	-3.50832	-0.61653	-0.41724
H	-2.80402	-2.12377	-0.99269
H	-4.02284	-3.38388	0.87019
H	-4.74640	-1.85737	1.40206
H	-5.83573	-1.58698	-0.86321
H	-5.11077	-3.11328	-1.39341
H	-7.50015	-3.47319	-0.66745
H	-6.39464	-4.35987	0.41707
H	-7.12473	-2.82325	0.95172
H	-1.06037	-3.62761	0.30324
H	-2.04512	-3.61161	1.77897
H	0.99126	-3.17887	1.56081
H	0.12132	-2.71532	3.01350
H	-0.73272	-5.09595	3.26191
H	0.10399	-5.56060	1.77123
H	1.45733	-6.21387	3.80434
H	2.34023	-4.99597	2.84253
H	1.49914	-4.50762	4.33551
H	1.38590	2.84750	1.60689
H	-0.36693	3.12799	1.43480
H	0.39813	1.76264	5.30640
H	1.94390	1.91170	4.43516
H	0.94326	4.04384	3.69269
H	-0.70081	3.35807	3.81635
H	1.02685	2.22636	-1.52786
H	0.15154	4.29099	-2.55072
H	-2.65999	1.24909	0.09227
H	-3.72749	3.26027	-0.82952
H	-2.20978	6.91172	-3.45712
H	-0.82539	6.52655	-2.38893
H	-1.07236	5.61854	-3.91770
H	-4.09166	6.41955	-2.60616
H	-4.53617	4.70829	-2.37489
H	-3.97790	5.68586	-0.97668
H	1.26029	-1.03834	1.25111
C	4.56020	0.82067	0.92297
H	3.61183	-0.97944	1.86177
C	5.09885	1.47385	2.21682
H	5.39732	0.28736	0.42171
H	4.23084	1.61360	0.22361
C	6.29713	2.41066	1.97297
H	4.27592	2.03354	2.70341
H	5.39258	0.67840	2.93196
C	6.83379	3.05491	3.26239
H	7.11019	1.84273	1.47666
H	6.00022	3.20512	1.25838
H	7.69213	3.72051	3.05790
H	6.05286	3.65747	3.76226
H	7.16968	2.28573	3.98247

9b

129

XYZ

Ru	0.28070	-0.16131	0.42732
C	2.08425	-0.24568	0.84974
C	3.26523	-0.52704	1.40502
N	-0.13348	1.79803	-0.66934
C	-1.30308	2.48809	-0.63608
C	-1.51129	3.72581	-1.24254
C	-0.45222	4.35646	-1.95358
C	0.78247	3.64721	-1.96709
C	0.88437	2.41140	-1.33221
N	-0.61048	5.57272	-2.58264
C	0.53697	6.22205	-3.21132
P	-2.09689	-0.93220	0.76999
C	-3.43763	-0.65297	-0.54350
C	-4.89550	-0.99420	-0.17606
C	-5.86032	-0.84410	-1.37092
C	-7.31849	-1.17412	-1.00925
N	0.15119	0.90334	2.30608
C	0.68936	2.26548	2.41072
C	0.53616	2.66193	3.90211
C	0.63480	1.30166	4.60677
C	0.16765	0.29396	3.52535
O	-0.13674	-0.89197	3.79297
P	0.73237	-1.43299	-1.50491
C	1.90530	-0.73115	-2.80830
C	3.37015	-0.52267	-2.38741
C	4.21697	0.12526	-3.50230
C	5.69132	0.31153	-3.10659
C	-2.95473	-0.37203	2.33442
C	-3.27884	1.13164	2.38579
C	-3.96412	1.54260	3.70498
C	-4.27654	3.04666	3.77585
C	-2.18104	-2.80449	0.94204
C	-1.74898	-3.35337	2.31849
C	-1.47091	-4.86873	2.27894
C	-1.11021	-5.44116	3.66009
C	-1.86372	6.30817	-2.43429
C	-0.61860	-2.01808	-2.68561
C	-1.27115	-0.90876	-3.53246
C	-2.42391	-1.42819	-4.41549
C	-3.05215	-0.33021	-5.28966
C	1.55049	-3.06044	-1.04551
C	2.07724	-4.00299	-2.14310
C	2.71254	-5.28078	-1.55389
C	3.25042	-6.23779	-2.63101
H	-1.38435	-2.53566	-2.07605
H	-0.18308	-2.78337	-3.35831
H	-1.64436	-0.10068	-2.87379
H	-0.50762	-0.44293	-4.18582
H	-2.04932	-2.24877	-5.05986

H	-3.20500	-1.88003	-3.77095
H	-3.87472	-0.72854	-5.91049
H	-3.46504	0.48630	-4.66860
H	-2.30328	0.11554	-5.97073
H	1.86158	-1.39695	-3.69426
H	1.47092	0.23705	-3.12562
H	3.41347	0.09890	-1.47354
H	3.82355	-1.49281	-2.10462
H	4.15430	-0.49524	-4.41916
H	3.77677	1.10765	-3.76997
H	6.27179	0.78702	-3.91782
H	5.78625	0.94639	-2.20643
H	6.16814	-0.65956	-2.87787
H	0.80830	-3.58720	-0.41402
H	2.36588	-2.77153	-0.35547
H	1.25957	-4.29915	-2.82990
H	2.83275	-3.48466	-2.76616
H	3.53236	-4.99353	-0.86607
H	1.96117	-5.80577	-0.93114
H	3.70026	-7.14078	-2.18050
H	2.44363	-6.56928	-3.31125
H	4.02693	-5.74922	-3.24883
H	-3.87380	-0.97749	2.46650
H	-2.24822	-0.63305	3.14430
H	-2.33022	1.69020	2.27685
H	-3.93547	1.42289	1.53800
H	-4.90032	0.96186	3.83107
H	-3.30868	1.25796	4.55074
H	-4.75983	3.31610	4.73244
H	-3.35307	3.64889	3.68601
H	-4.95664	3.35490	2.95899
H	-3.37629	0.40277	-0.87035
H	-3.12699	-1.25176	-1.42096
H	-4.96028	-2.03159	0.20769
H	-5.24019	-0.34052	0.64841
H	-5.79944	0.19155	-1.76185
H	-5.52368	-1.50299	-2.19651
H	-7.98721	-1.06089	-1.88148
H	-7.41418	-2.21412	-0.64575
H	-7.69296	-0.50728	-0.21041
H	-1.51214	-3.20462	0.15609
H	-3.20523	-3.15016	0.69895
H	-0.85992	-2.80550	2.68375
H	-2.54905	-3.15167	3.05767
H	-2.35391	-5.40454	1.87254
H	-0.64043	-5.06518	1.57005
H	-0.89065	-6.52323	3.60645
H	-0.22270	-4.93290	4.07862
H	-1.93989	-5.30156	4.37791
H	1.76108	2.28531	2.10530
H	0.15307	2.96115	1.73859
H	0.03244	1.20588	5.52637
H	1.68137	1.05022	4.87149
H	1.29132	3.40176	4.22485
H	-0.46303	3.10742	4.07191
H	1.83552	1.87206	-1.32770
H	1.66876	4.05472	-2.45962
H	-2.11146	2.02402	-0.06541
H	-2.49315	4.19534	-1.14492
H	0.20547	7.15786	-3.68566
H	1.33401	6.46670	-2.47958
H	0.97549	5.57994	-3.99884
H	-1.80492	7.24100	-3.01470
H	-2.71950	5.72186	-2.82045
H	-2.07240	6.57017	-1.37689
H	0.34699	-1.53462	1.27697
C	4.54286	0.27460	1.21271
H	3.30536	-1.37640	2.10745
C	5.09891	0.85858	2.53135
H	5.32705	-0.36401	0.75026
H	4.35791	1.10277	0.50036
C	6.41264	1.64014	2.34214
H	4.33126	1.51679	2.98374
H	5.26058	0.03347	3.25492
C	6.97032	2.21596	3.65478
H	7.16998	0.97647	1.87745
H	6.24529	2.46478	1.61939
H	7.90693	2.77802	3.48590
H	6.24569	2.90454	4.12776
H	7.18803	1.41273	4.38312

[9b-10]‡

129

XYZ

Ru	0.04914	-0.37165	-0.47846
C	-1.48487	-0.62255	-1.56429
C	-2.47792	-0.90017	-2.42494
N	-0.47796	1.80898	0.16991
C	0.32859	2.62213	0.89851
C	-0.02834	3.88581	1.36914
C	-1.32369	4.40501	1.08897
C	-2.16677	3.56249	0.31041
C	-1.70934	2.31380	-0.10849
N	-1.73481	5.64178	1.54153
C	-3.04176	6.16144	1.14991
P	2.47238	-0.50443	-0.26295
C	3.53304	0.32183	1.08308
C	5.06937	0.27533	0.95899
C	5.78205	0.91021	2.17198
C	7.31530	0.87766	2.05334
N	0.04614	0.24283	-2.63573
C	-0.38525	1.56667	-3.12408
C	0.16414	1.68225	-4.56988
C	0.24453	0.21572	-5.01633

C	0.45026	-0.55891	-3.69268
O	0.89913	-1.71211	-3.62809
P	-0.56502	-1.30278	1.53691
C	-1.97846	-0.48482	2.47895
C	-3.34226	-0.46778	1.77069
C	-4.39004	0.37021	2.53134
C	-5.77643	0.35564	1.86659
C	3.38065	0.03964	-1.80984
C	3.35749	1.55941	-2.05226
C	4.09226	1.96643	-3.34573
C	4.08170	3.48425	-3.59309
C	3.06557	-2.28781	-0.09507
C	2.98847	-3.12515	-1.38793
C	3.16107	-4.63292	-1.11657
C	3.14955	-5.47323	-2.40463
C	-0.79933	6.50128	2.26039
C	0.64136	-1.50934	2.97845
C	0.95204	-0.22716	3.77459
C	2.03802	-0.43939	4.84919
C	2.32013	0.82535	5.67737
C	-1.16365	-3.07312	1.31946
C	-1.81898	-3.80855	2.50365
C	-2.26204	-5.23936	2.13055
C	-2.92053	-5.99015	3.30012
H	1.57460	-1.92599	2.55314
H	0.23858	-2.27572	3.66983
H	1.26809	0.58040	3.08511
H	0.03107	0.13673	4.27015
H	1.72741	-1.26351	5.52238
H	2.97430	-0.78107	4.36299
H	3.09679	0.64380	6.44195
H	2.67032	1.65392	5.03405
H	1.40912	1.17151	6.20038
H	-2.07111	-0.97513	3.46947
H	-1.65273	0.55612	2.66585
H	-3.21547	-0.06592	0.74808
H	-3.72041	-1.50334	1.65189
H	-4.47516	-0.00250	3.57221
H	-4.02468	1.41419	2.60740
H	-6.50177	0.97386	2.42631
H	-5.72811	0.74625	0.83312
H	-6.18370	-0.67129	1.81395
H	-0.27981	-3.63289	0.95630
H	-1.85208	-3.03328	0.45447
H	-1.11905	-3.86724	3.36115
H	-2.70162	-3.24394	2.86514
H	-2.96558	-5.18999	1.27589
H	-1.38297	-5.80936	1.76956
H	-3.22882	-7.00884	3.00345
H	-2.22647	-6.08629	4.15604
H	-3.82145	-5.45913	3.66051
H	4.42558	-0.32612	-1.75840

H	2.88824	-0.49007	-2.64469
H	2.30588	1.90142	-2.09687
H	3.82295	2.09311	-1.19792
H	5.13963	1.60549	-3.30021
H	3.62741	1.44464	-4.20563
H	4.61578	3.74754	-4.52402
H	3.04766	3.86751	-3.68008
H	4.56832	4.02864	-2.76192
H	3.21025	1.37962	1.14386
H	3.22331	-0.14256	2.03943
H	5.41580	-0.77109	0.84934
H	5.38956	0.80250	0.03934
H	5.44093	1.95883	2.28793
H	5.46947	0.38283	3.09563
H	7.79917	1.33628	2.93453
H	7.68738	-0.16029	1.96874
H	7.65880	1.42783	1.15761
H	2.41723	-2.74590	0.67662
H	4.09971	-2.30205	0.30228
H	2.02722	-2.93929	-1.90275
H	3.78040	-2.79293	-2.08811
H	4.10708	-4.81065	-0.56395
H	2.34589	-4.97416	-0.44616
H	3.25012	-6.55230	-2.18639
H	2.20869	-5.32408	-2.96458
H	3.98188	-5.18681	-3.07431
H	-1.49402	1.63174	-3.12050
H	-0.00490	2.36464	-2.46246
H	1.04953	-0.01370	-5.73370
H	-0.70827	-0.12848	-5.46876
H	-0.47185	2.31851	-5.21082
H	1.17624	2.12789	-4.55480
H	-2.34811	1.66389	-0.71678
H	-3.17436	3.87360	0.02269
H	1.32460	2.22304	1.11241
H	0.70449	4.45780	1.94388
H	-3.19600	7.14277	1.62308
H	-3.12948	6.28879	0.05144
H	-3.85664	5.49094	1.48491
H	-1.31435	7.42792	2.55498
H	-0.43028	6.01049	3.18181
H	0.07872	6.77544	1.64016
H	0.33491	-1.88550	-0.92664
C	-3.85101	-1.39773	-2.00525
H	-2.30986	-0.82748	-3.50891
C	-5.00594	-0.49401	-2.49496
H	-4.00512	-2.41857	-2.41751
H	-3.90203	-1.49941	-0.90544
C	-6.40302	-1.06416	-2.18512
H	-4.90065	0.50991	-2.03492
H	-4.90766	-0.33879	-3.58930
C	-7.54845	-0.16588	-2.68176

H	-6.49503	-2.06898	-2.64448
H	-6.50063	-1.21796	-1.09161
H	-8.53694	-0.60144	-2.44805
H	-7.50413	0.83499	-2.21275
H	-7.49624	-0.02128	-3.77708

10

129

XYZ

C	0.88064	2.89400	0.24672
N	0.12450	2.13094	-0.58583
C	-0.72741	2.82989	-1.38605
C	-0.85137	4.21958	-1.39087
C	-0.05503	5.01158	-0.51769
C	0.83278	4.28476	0.32482
Ru	0.10974	-0.19676	-0.50682
C	-2.20469	-0.05895	-2.85062
C	-1.05640	-0.25335	-2.14677
N	-0.13465	6.38964	-0.48986
C	-1.14104	7.07428	-1.29582
P	-1.39027	-0.48885	1.12514
C	-2.72238	-1.77435	0.78145
C	-2.19646	-3.19929	0.52990
C	-3.33399	-4.21304	0.29179
C	-2.81707	-5.63765	0.02963
N	0.17784	-0.46333	-2.92692
C	0.57907	-1.73821	-3.40443
C	1.66875	-1.51576	-4.47057
C	1.99412	-0.01268	-4.40620
C	0.70459	0.59928	-3.81864
O	0.11840	-2.82177	-3.06384
P	2.24961	-0.45233	0.57188
C	2.67069	0.12402	2.33740
C	4.12762	0.22719	2.82912
C	4.21927	0.56875	4.33193
C	5.66726	0.69061	4.83616
C	-2.38385	1.05056	1.61287
C	-3.83426	0.90819	2.11944
C	-4.45105	2.26590	2.51615
C	-5.90618	2.14870	3.00049
C	-0.73925	-1.08680	2.79569
C	-1.71601	-1.48531	3.92065
C	-0.98456	-2.03439	5.16416
C	-1.94056	-2.43728	6.29953
C	3.57181	0.42082	-0.48055
C	5.03220	-0.07649	-0.51215
C	5.91249	0.76272	-1.46272
C	7.36888	0.27226	-1.52626
C	2.88755	-2.22309	0.67056
C	2.81887	-2.99675	-0.65825
C	3.15889	-4.49278	-0.50999
C	3.11569	-5.23686	-1.85579

C	0.62787	7.14059	0.50258
H	-2.18306	-0.16398	-3.95041
H	-2.37533	1.69565	0.71506
H	-1.76660	1.57615	2.36943
H	-4.46110	0.44743	1.33058
H	-3.88319	0.22414	2.98848
H	-3.83087	2.72932	3.30995
H	-4.40218	2.95436	1.64868
H	-6.32216	3.13462	3.27670
H	-6.55508	1.71922	2.21475
H	-5.98101	1.49307	3.88810
H	-3.44155	-1.77779	1.62434
H	-3.26987	-1.42672	-0.11212
H	-1.52039	-3.18007	-0.34455
H	-1.58767	-3.53952	1.39314
H	-4.01822	-4.21963	1.16517
H	-3.94222	-3.87761	-0.57239
H	-3.64805	-6.34457	-0.14822
H	-2.15676	-5.66143	-0.85629
H	-2.23399	-6.01574	0.89059
H	-0.07116	-0.27940	3.14990
H	-0.08184	-1.94375	2.55153
H	-2.32648	-0.61177	4.22264
H	-2.42785	-2.25236	3.55688
H	-0.37180	-2.90875	4.86613
H	-0.26971	-1.27155	5.53366
H	-1.38855	-2.82607	7.17429
H	-2.54360	-1.57488	6.64004
H	-2.64326	-3.22486	5.96958
H	3.16429	0.40221	-1.51016
H	3.54457	1.48804	-0.17997
H	5.47621	-0.05497	0.49957
H	5.06156	-1.13371	-0.83855
H	5.47038	0.74417	-2.47956
H	5.89073	1.82346	-1.14047
H	7.97387	0.88809	-2.21607
H	7.84909	0.31421	-0.53082
H	7.42277	-0.77513	-1.87699
H	2.17106	1.10421	2.47139
H	2.11521	-0.57762	2.98978
H	4.66228	-0.72523	2.64630
H	4.66689	1.00483	2.25349
H	3.67763	1.51698	4.52451
H	3.68759	-0.21039	4.91393
H	5.70158	0.93205	5.91386
H	6.22271	-0.25393	4.68655
H	6.21375	1.48667	4.29670
H	2.23705	-2.72239	1.41647
H	3.91809	-2.24130	1.07826
H	1.80206	-2.89595	-1.08139
H	3.51190	-2.54159	-1.39578
H	4.15949	-4.61125	-0.04556

H	2.43636	-4.95547	0.19199
H	3.28649	-6.32135	-1.72769
H	2.13677	-5.09725	-2.34955
H	3.89351	-4.85633	-2.54510
H	-0.03655	0.81789	-4.61542
H	0.87449	1.52499	-3.24707
H	2.52628	-2.18460	-4.28784
H	1.23602	-1.80360	-5.44842
H	2.25197	0.42837	-5.38411
H	2.84175	0.16853	-3.72076
H	-1.34184	2.21307	-2.05269
H	-1.57616	4.67490	-2.07101
H	1.55559	2.33679	0.90181
H	1.48332	4.79094	1.04296
H	-1.04049	8.16049	-1.15125
H	-1.00313	6.86157	-2.37342
H	-2.17381	6.78081	-1.01598
H	0.44595	8.21634	0.35923
H	0.34143	6.87591	1.54148
H	1.71494	6.96342	0.38985
H	0.07192	-1.80694	-0.47571
C	-3.55468	0.29175	-2.27333
C	-4.65610	-0.74788	-2.58986
H	-3.46368	0.42420	-1.17818
H	-3.89590	1.27471	-2.67261
C	-6.04364	-0.36108	-2.04497
H	-4.71787	-0.88653	-3.68904
H	-4.35351	-1.73190	-2.17902
C	-7.13419	-1.39550	-2.37066
H	-5.97919	-0.22729	-0.94557
H	-6.33527	0.62744	-2.45500
H	-8.11627	-1.09502	-1.96192
H	-7.25013	-1.52245	-3.46327
H	-6.88304	-2.38578	-1.94725

[10-11]‡

129

XYZ

C	0.65024	3.22844	0.00015
N	0.05212	2.32305	-0.82310
C	-1.00558	2.80213	-1.53775
C	-1.49691	4.10387	-1.44679
C	-0.88629	5.03691	-0.56142
C	0.22883	4.54640	0.17457
Ru	0.13690	0.05180	-0.44100
C	-1.99165	-0.34059	-2.96994
C	-0.77767	-0.41475	-2.32744
N	-1.33845	6.33610	-0.43376
C	-2.54844	6.75457	-1.13474
P	-1.18020	-0.65777	1.19553
C	-2.19498	-2.22233	0.90118
C	-1.35729	-3.51267	0.83982
C	-2.20284	-4.75467	0.49188
C	-1.37346	-6.04865	0.44238
N	0.39151	-0.49353	-3.20070
C	0.98300	-1.69685	-3.57654
C	2.01467	-1.37928	-4.67631
C	2.14204	0.15648	-4.66003
C	0.79580	0.62954	-4.05982
O	0.70673	-2.81325	-3.13015
P	2.14557	-0.10574	0.67533
C	2.62548	0.69654	2.33912
C	4.09879	0.85977	2.76458
C	4.24042	1.38145	4.21092
C	5.70362	1.57782	4.64276
C	-2.50178	0.67061	1.54617
C	-3.94962	0.25819	1.88554
C	-4.85323	1.47645	2.16945
C	-6.30818	1.08813	2.48317
C	-0.53551	-0.97752	2.94360
C	-1.51836	-1.41925	4.04591
C	-0.81757	-1.64566	5.40266
C	-1.78645	-2.07950	6.51570
C	3.37708	0.67442	-0.54214
C	4.85406	0.22487	-0.57550
C	5.67431	0.99951	-1.62853
C	7.14538	0.55463	-1.69230
C	2.85202	-1.83715	0.94880
C	2.75575	-2.73310	-0.29826
C	3.00952	-4.22550	-0.00739
C	3.05522	-5.06956	-1.29336
C	-0.75310	7.21098	0.57707
H	-2.00088	-0.38125	-4.07257
H	-2.50515	1.31096	0.64460
H	-2.08356	1.29571	2.36113
H	-4.38170	-0.31810	1.04335
H	-3.97114	-0.41718	2.76269
H	-4.43072	2.05327	3.01689
H	-4.82966	2.15650	1.29448
H	-6.93152	1.97882	2.68196
H	-6.76600	0.54027	1.63870
H	-6.36499	0.43421	3.37335
H	-2.97953	-2.31124	1.67851
H	-2.71404	-2.07852	-0.06540
H	-0.55647	-3.38640	0.08596
H	-0.85198	-3.68269	1.81285
H	-3.02165	-4.86499	1.23200
H	-2.69292	-4.59243	-0.48877
H	-2.00023	-6.92127	0.18308
H	-0.56956	-5.97519	-0.31246
H	-0.89779	-6.25678	1.41938
H	-0.04353	-0.03272	3.24260
H	0.27212	-1.72776	2.84120
H	-2.30936	-0.65524	4.17650

H	-2.03293	-2.35422	3.74916
H	-0.02608	-2.41179	5.27941
H	-0.29806	-0.71398	5.70437
H	-1.25827	-2.23529	7.47366
H	-2.56908	-1.31643	6.68463
H	-2.29560	-3.02621	6.25587
H	2.92684	0.50550	-1.54025
H	3.32295	1.76927	-0.37522
H	5.32562	0.35833	0.41501
H	4.91278	-0.85675	-0.80146
H	5.20424	0.86939	-2.62456
H	5.62305	2.08471	-1.40670
H	7.70803	1.12377	-2.45426
H	7.65082	0.70525	-0.72015
H	7.22748	-0.51846	-1.94656
H	2.12552	1.68561	2.35159
H	2.09645	0.09010	3.09999
H	4.63394	-0.10669	2.67982
H	4.61359	1.56319	2.08110
H	3.69246	2.34056	4.30743
H	3.74062	0.67335	4.90194
H	5.77304	1.94787	5.68168
H	6.26756	0.62809	4.58708
H	6.21863	2.30869	3.99163
H	2.25237	-2.27840	1.77019
H	3.89711	-1.77646	1.31341
H	1.75482	-2.60917	-0.75703
H	3.48172	-2.38863	-1.06262
H	3.95758	-4.34778	0.55619
H	2.20590	-4.60076	0.65804
H	3.11951	-6.15031	-1.07002
H	2.15851	-4.89044	-1.91309
H	3.93578	-4.80297	-1.90823
H	0.03884	0.80739	-4.85282
H	0.88488	1.55250	-3.46099
H	2.95755	-1.91956	-4.48885
H	1.61039	-1.75375	-5.63694
H	2.32843	0.59739	-5.65398
H	2.97105	0.46258	-3.99591
H	-1.47708	2.07561	-2.21079
H	-2.35760	4.38094	-2.06129
H	1.51371	2.85667	0.55934
H	0.77504	5.18299	0.87560
H	-2.73966	7.81704	-0.92115
H	-2.43411	6.64494	-2.23032
H	-3.43906	6.17119	-0.82155
H	-1.24072	8.19626	0.52771
H	-0.88172	6.81074	1.60416
H	0.32962	7.36021	0.39983
H	-0.55543	-1.26647	-1.36260
C	-3.34344	-0.25189	-2.31473
C	-4.27263	-1.43689	-2.67659

H	-3.21986	-0.18654	-1.21680
H	-3.85766	0.68840	-2.61991
C	-5.68876	-1.31288	-2.08418
H	-4.34309	-1.51848	-3.78080
H	-3.80384	-2.38087	-2.33328
C	-6.60573	-2.49002	-2.45670
H	-5.61752	-1.23567	-0.97993
H	-6.14604	-0.36245	-2.42726
H	-7.61351	-2.37560	-2.01756
H	-6.72466	-2.57103	-3.55335
H	-6.18959	-3.44927	-2.09692

11

129

XYZ

C	2.48813	0.94704	-2.34264
N	2.12871	0.71702	-1.04433
C	3.15561	0.54546	-0.16218
C	4.50304	0.55942	-0.52169
C	4.87958	0.77150	-1.87870
C	3.80667	0.97088	-2.79486
Ru	0.11787	0.21020	-0.60635
C	2.04311	-3.06952	-0.05412
C	1.72093	-2.53078	1.14532
N	6.20545	0.79442	-2.28150
C	7.26215	0.77514	-1.27517
P	-1.96559	-0.71491	-0.49800
C	-2.09193	-2.41587	0.33681
C	-2.06523	-2.36160	1.87605
C	-1.98141	-3.75895	2.52309
C	-2.02853	-3.70345	4.05989
N	2.25783	-2.95025	2.37100
C	1.81623	-2.46033	3.60037
C	2.56542	-3.22412	4.70001
C	3.73463	-3.91136	3.96698
C	3.25197	-4.02134	2.49845
O	0.95433	-1.59084	3.75167
P	-0.46606	2.23723	0.06024
C	-1.77535	3.16173	-0.97651
C	-2.77262	4.12390	-0.29711
C	-3.72117	4.79645	-1.31143
C	-4.74158	5.74079	-0.65316
C	-2.61904	-1.06698	-2.26082
C	-3.38296	-2.36949	-2.57605
C	-3.86015	-2.43407	-4.04232
C	-4.61590	-3.73210	-4.37490
C	-3.48312	0.12020	0.27264
C	-4.86117	-0.55879	0.15434
C	-5.96825	0.21113	0.90556
C	-7.35229	-0.44823	0.77993
C	0.95980	3.48233	0.00305
C	0.70369	4.94199	0.42387

C	1.97877	5.80893	0.35022
C	1.73847	7.27269	0.75759
C	-1.07042	2.47817	1.83484
C	-0.06376	1.98082	2.88820
C	-0.61067	2.06441	4.32784
C	0.39762	1.56648	5.37756
C	6.53555	1.20650	-3.64237
H	2.74804	-3.91124	-0.11341
H	-1.72403	-0.99716	-2.90812
H	-3.24269	-0.18989	-2.52970
H	-2.73246	-3.24348	-2.37164
H	-4.26079	-2.48079	-1.91027
H	-4.51207	-1.56172	-4.25044
H	-2.98589	-2.32882	-4.71542
H	-4.95071	-3.74882	-5.42803
H	-3.97660	-4.62002	-4.21261
H	-5.51157	-3.84709	-3.73625
H	-3.00246	-2.94485	-0.00539
H	-1.22629	-3.00530	-0.02373
H	-1.20808	-1.75054	2.21788
H	-2.97839	-1.85396	2.24695
H	-2.80815	-4.39465	2.14469
H	-1.04256	-4.24928	2.19560
H	-1.93970	-4.71085	4.50755
H	-1.20856	-3.07508	4.45207
H	-2.98152	-3.26553	4.41221
H	-3.53487	1.13376	-0.16899
H	-3.23288	0.27038	1.34068
H	-5.14759	-0.64639	-0.91222
H	-4.81462	-1.59293	0.54967
H	-5.69164	0.29257	1.97585
H	-6.01455	1.25006	0.52181
H	-8.12434	0.12189	1.32777
H	-7.67036	-0.51263	-0.27751
H	-7.34270	-1.47649	1.18719
H	1.76517	3.04644	0.62387
H	1.33373	3.44922	-1.03800
H	-0.07176	5.39490	-0.22519
H	0.30547	4.98025	1.45721
H	2.75759	5.36465	1.00209
H	2.38490	5.77148	-0.68068
H	2.66691	7.86906	0.69549
H	0.98786	7.75209	0.10168
H	1.36425	7.34206	1.79603
H	-1.19742	3.70118	-1.75457
H	-2.32871	2.37308	-1.51733
H	-3.37894	3.57110	0.44821
H	-2.23529	4.90936	0.26932
H	-3.12018	5.35832	-2.05459
H	-4.25608	4.01187	-1.88374
H	-5.41037	6.20148	-1.40280
H	-5.37536	5.19964	0.07407
H	-4.23590	6.55925	-0.10748
H	-2.01323	1.90885	1.93118
H	-1.32038	3.54348	2.01068
H	0.21327	0.93424	2.65356
H	0.87070	2.57643	2.82599
H	-0.90635	3.10966	4.55459
H	-1.53823	1.46101	4.39492
H	-0.01971	1.62803	6.39982
H	0.67603	0.51589	5.18009
H	1.32304	2.17379	5.35850
H	2.78336	-5.00268	2.27946
H	4.06811	-3.86981	1.76833
H	2.87284	-2.53534	5.50374
H	1.86320	-3.95431	5.14705
H	3.99763	-4.89679	4.38553
H	4.63699	-3.27625	4.01399
H	2.85071	0.38847	0.87689
H	5.25127	0.41482	0.26162
H	1.64934	1.11712	-3.02586
H	3.99030	1.16593	-3.85442
H	8.24014	0.78322	-1.78026
H	7.21581	1.64804	-0.58992
H	7.20805	-0.14478	-0.66276
H	7.62573	1.14644	-3.78210
H	6.06442	0.53488	-4.38465
H	6.21158	2.24553	-3.86371
H	1.02365	-1.67615	1.22862
C	1.50426	-2.57775	-1.37355
C	1.03252	-3.72030	-2.30217
H	0.64175	-1.87693	-1.19837
H	2.27926	-1.98817	-1.90637
C	0.67336	-3.24891	-3.72239
H	1.83778	-4.48216	-2.36452
H	0.16302	-4.23240	-1.84361
C	0.19166	-4.39002	-4.63345
H	-0.10874	-2.46677	-3.65883
H	1.55853	-2.75854	-4.17538
H	-0.05859	-4.02172	-5.64464
H	0.96723	-5.17126	-4.74372
H	-0.71222	-4.87557	-4.22090

[5-6m]‡

129

XYZ

C	1.54557	-1.89771	-2.30692
N	1.25130	-1.70882	-0.99317
C	2.02696	-2.39936	-0.12230
C	3.07635	-3.24610	-0.48220
C	3.40076	-3.42071	-1.85699
C	2.57582	-2.71293	-2.77551
Ru	-0.49673	-0.19322	-0.38588
C	-2.26281	-0.59327	-1.67253

C	-1.23291	-0.50814	-2.49892
N	4.44542	-4.22398	-2.27027
C	5.18030	-5.01982	-1.29208
P	0.71903	1.67388	-0.97541
C	0.19566	2.53675	-2.55987
C	-1.25197	3.06118	-2.55519
C	-1.66568	3.67803	-3.90636
C	-3.10803	4.21162	-3.90544
N	-1.92529	-1.91210	-0.44791
C	-2.93760	-2.23727	0.46641
C	-3.29376	-3.73276	0.32678
C	-2.63631	-4.14606	-1.00034
C	-1.45261	-3.15948	-1.08994
O	-3.47429	-1.46291	1.27051
P	-0.02269	-0.10990	1.91731
C	1.79848	0.11106	2.44950
C	2.14779	0.94093	3.70205
C	3.66714	0.98596	3.96803
C	4.03860	1.82910	5.19970
C	2.53632	1.32611	-1.35662
C	3.53124	2.45849	-1.67980
C	4.89410	1.91608	-2.16133
C	5.91423	3.02732	-2.46142
C	0.74288	3.11725	0.24384
C	1.14277	4.53824	-0.20603
C	1.07889	5.55193	0.95634
C	1.44687	6.98244	0.52760
C	-0.50106	-1.67429	2.85467
C	-0.19827	-1.76774	4.36238
C	-0.68986	-3.09459	4.97906
C	-0.39366	-3.20666	6.48416
C	-0.88689	1.20730	2.95382
C	-2.42211	1.10321	2.99548
C	-3.06968	2.33319	3.66330
C	-4.60219	2.23072	3.74354
C	4.65227	-4.46758	-3.69492
H	-1.61532	0.87444	0.06244
H	2.49639	0.61524	-2.20513
H	2.91502	0.72961	-0.50453
H	3.11960	3.12997	-2.45890
H	3.69619	3.08543	-0.78201
H	5.30498	1.23071	-1.39306
H	4.73745	1.29892	-3.06859
H	6.87726	2.61005	-2.80714
H	5.54271	3.70930	-3.24889
H	6.11772	3.63789	-1.56194
H	0.90160	3.36346	-2.77347
H	0.32055	1.79045	-3.36745
H	-1.93172	2.22800	-2.29839
H	-1.37891	3.82040	-1.75723
H	-0.96649	4.49792	-4.16892
H	-1.55393	2.91382	-4.70203

H	-3.38294	4.63985	-4.88639
H	-3.83009	3.40625	-3.67598
H	-3.24116	5.00313	-3.14444
H	1.38370	2.79639	1.08951
H	-0.28859	3.14410	0.64266
H	2.16169	4.54621	-0.63379
H	0.46696	4.88106	-1.01318
H	0.05895	5.54465	1.39015
H	1.75842	5.22083	1.76740
H	1.39387	7.68551	1.37829
H	2.47391	7.02666	0.11949
H	0.76114	7.35337	-0.25675
H	-1.58584	-1.78516	2.67375
H	-0.01463	-2.51439	2.31982
H	0.89114	-1.67100	4.54347
H	-0.67929	-0.92610	4.89899
H	-1.77964	-3.19166	4.80523
H	-0.21696	-3.94237	4.44316
H	-0.75620	-4.16515	6.89762
H	0.69242	-3.14439	6.68508
H	-0.88353	-2.39195	7.04930
H	2.21451	-0.90953	2.56723
H	2.32020	0.54362	1.57562
H	1.77318	1.97702	3.58530
H	1.63849	0.52914	4.59443
H	4.04517	-0.04827	4.09808
H	4.18228	1.38868	3.07265
H	5.13103	1.84425	5.36505
H	3.70255	2.87627	5.08347
H	3.56630	1.42852	6.11589
H	-0.59261	2.18257	2.52420
H	-0.48092	1.18161	3.98304
H	-2.81561	0.97210	1.97153
H	-2.72302	0.19053	3.54516
H	-2.65326	2.46851	4.68289
H	-2.78954	3.24473	3.09655
H	-5.04493	3.12695	4.21555
H	-5.04570	2.12322	2.73678
H	-4.91214	1.34973	4.33554
H	-1.12957	-2.91016	-2.11731
H	-0.57580	-3.54653	-0.53791
H	-2.85031	-4.27541	1.18579
H	-4.38719	-3.87316	0.38474
H	-3.33363	-3.98165	-1.84322
H	-2.31186	-5.20118	-1.03121
H	1.77990	-2.27132	0.93599
H	3.62619	-3.76618	0.30646
H	0.85781	-1.38092	-2.98948
H	2.72344	-2.79881	-3.85556
H	5.99131	-5.56037	-1.80318
H	5.63924	-4.37474	-0.51858
H	4.53467	-5.76445	-0.78151

H	5.53959	-5.10522	-3.82614
H	3.78573	-4.97607	-4.16594
H	4.83218	-3.52057	-4.23862
H	-1.25397	0.14724	-3.37699
C	-3.69777	-0.15672	-1.57641
C	-4.64271	-0.96784	-2.49265
H	-4.05129	-0.20267	-0.53278
H	-3.73549	0.91136	-1.87380
C	-6.09925	-0.47023	-2.43578
H	-4.27239	-0.92253	-3.53627
H	-4.60977	-2.03645	-2.19898
C	-7.04862	-1.27856	-3.33621
H	-6.45654	-0.51026	-1.38753
H	-6.13131	0.59864	-2.72930
H	-8.08511	-0.89961	-3.27665
H	-6.73374	-1.23003	-4.39548
H	-7.06466	-2.34511	-3.04347

6m

129

XYZ

C	2.18794	-1.66921	1.49892
N	0.90708	-1.60088	1.04284
C	0.12740	-2.66798	1.35875
C	0.55072	-3.78058	2.08454
C	1.89345	-3.85152	2.55276
C	2.71324	-2.73376	2.22994
Ru	0.21614	0.23999	-0.23705
C	3.13639	1.19762	-0.64667
C	2.03687	1.16936	0.16775
N	2.36592	-4.92919	3.27095
C	1.49321	-6.06864	3.53611
P	-0.80004	1.46464	1.33407
C	-0.00349	3.09035	1.83480
C	0.23357	4.07201	0.67304
C	0.90295	5.38282	1.13138
C	1.13494	6.37067	-0.02425
N	3.09040	0.49092	-1.89851
C	3.62022	0.91184	-3.11438
C	3.07337	-0.02156	-4.21048
C	2.43971	-1.19809	-3.44994
C	2.15194	-0.61709	-2.04127
O	4.37305	1.87264	-3.30353
P	-1.72394	-0.79890	-1.18486
C	-3.14825	-1.52764	-0.13016
C	-4.61027	-1.40637	-0.60589
C	-5.60433	-2.06071	0.37658
C	-7.06993	-1.92748	-0.07067
C	-0.89710	0.57620	2.99508
C	-1.60147	1.21238	4.20994
C	-1.44810	0.35809	5.48668
C	-2.15367	0.96785	6.70984
C	-2.57030	2.01093	0.96239
C	-3.19239	3.20986	1.70988
C	-4.65932	3.45240	1.29502
C	-5.29369	4.65828	2.00839
C	-1.26620	-2.33015	-2.20624
C	-2.37044	-3.10839	-2.94787
C	-1.81767	-4.29989	-3.75802
C	-2.91007	-5.07608	-4.51296
C	-2.66979	0.20738	-2.47410
C	-1.79610	0.70378	-3.64020
C	-2.55490	1.64975	-4.59365
C	-1.68671	2.14028	-5.76439
C	3.76223	-4.96491	3.69678
H	-0.09380	1.52606	-1.16919
H	0.16298	0.36589	3.23948
H	-1.34600	-0.41122	2.77484
H	-1.19676	2.22477	4.40639
H	-2.68028	1.34203	3.99637
H	-1.84761	-0.65798	5.29447
H	-0.36988	0.22597	5.70673
H	-2.02567	0.33573	7.60709
H	-1.74964	1.96990	6.94563
H	-3.23939	1.08044	6.53153
H	-0.63432	3.55685	2.61630
H	0.96091	2.84106	2.31678
H	0.85843	3.57163	-0.08964
H	-0.72940	4.30994	0.17642
H	0.28159	5.86548	1.91372
H	1.87232	5.14393	1.61340
H	1.62226	7.29809	0.32766
H	1.77987	5.92557	-0.80375
H	0.17993	6.65516	-0.50448
H	-3.20619	1.11447	1.09838
H	-2.56685	2.22166	-0.12282
H	-3.15029	3.05835	2.80461
H	-2.60729	4.12632	1.50203
H	-4.70735	3.60208	0.19778
H	-5.25366	2.54054	1.50629
H	-6.34229	4.80780	1.69397
H	-5.28884	4.52226	3.10596
H	-4.74105	5.58995	1.78624
H	-0.50217	-1.99071	-2.93213
H	-0.73091	-3.00145	-1.50547
H	-3.12050	-3.48456	-2.22376
H	-2.91606	-2.43068	-3.63447
H	-1.06186	-3.92882	-4.47910
H	-1.27722	-4.98589	-3.07513
H	-2.48383	-5.91940	-5.08562
H	-3.66235	-5.49081	-3.81618
H	-3.44339	-4.42174	-5.22733
H	-2.90274	-2.59931	0.01778
H	-3.05842	-1.05894	0.86849

H	-4.87620	-0.33808	-0.72804
H	-4.73352	-1.87020	-1.60354
H	-5.34766	-3.13298	0.49432
H	-5.47908	-1.60499	1.37953
H	-7.75759	-2.40520	0.65026
H	-7.36484	-0.86567	-0.16229
H	-7.23290	-2.40375	-1.05536
H	-3.09483	1.07880	-1.94071
H	-3.52510	-0.37604	-2.86453
H	-0.90992	1.22392	-3.22776
H	-1.41653	-0.16192	-4.22094
H	-3.45637	1.13746	-4.98715
H	-2.92437	2.52149	-4.01703
H	-2.24739	2.82588	-6.42523
H	-0.79451	2.68085	-5.39820
H	-1.33354	1.29374	-6.38274
H	2.29296	-1.35658	-1.23805
H	1.07621	-0.21937	-2.01188
H	2.32599	0.55000	-4.79408
H	3.87758	-0.31072	-4.90770
H	3.15910	-2.03188	-3.35827
H	1.53002	-1.59739	-3.92983
H	-0.90686	-2.61589	1.00462
H	-0.16481	-4.58378	2.27883
H	2.81108	-0.80323	1.24036
H	3.75984	-2.68476	2.54212
H	2.04838	-6.82159	4.11515
H	0.60369	-5.77165	4.12632
H	1.14107	-6.54608	2.59927
H	3.94424	-5.89293	4.25904
H	4.45829	-4.94193	2.83421
H	4.00612	-4.11090	4.35862
H	2.22499	1.72377	1.10588
C	4.44090	1.91377	-0.33126
C	5.67659	0.99051	-0.22654
H	4.64757	2.66981	-1.11149
H	4.30035	2.44956	0.62753
C	6.96699	1.75765	0.11752
H	5.49354	0.21031	0.54189
H	5.81644	0.46173	-1.18912
C	8.20506	0.85009	0.21236
H	7.13670	2.53678	-0.65174
H	6.82916	2.29749	1.07669
H	9.11522	1.42620	0.46098
H	8.07561	0.07568	0.99226
H	8.38985	0.32853	-0.74532

7m

148

XYZ

C	-0.94419	-1.09310	2.82648
N	0.09643	-0.43465	2.24220
C	1.08458	-0.06255	3.09947
C	1.08298	-0.29455	4.47479
C	-0.01774	-0.96163	5.08186
C	-1.04726	-1.37095	4.19025
Ru	0.06651	-0.04608	-0.06662
C	-2.33710	-2.20555	-0.69126
C	-1.17110	-1.74647	-0.15052
N	-0.08300	-1.19073	6.44416
C	1.06915	-0.88100	7.28484
P	1.71634	-1.56722	-0.63498
C	1.25618	-2.85140	-1.92293
C	0.81044	-2.27855	-3.28074
C	0.59069	-3.38154	-4.33504
C	0.09612	-2.81933	-5.67831
N	-3.25694	-1.37157	-1.44777
C	-3.28283	-1.30221	-2.82412
C	-4.45109	-0.37602	-3.22229
C	-5.34636	-0.33644	-1.96900
C	-4.37920	-0.67216	-0.80237
O	-2.50738	-1.86676	-3.60573
P	1.45138	1.92365	-0.10265
C	3.11730	1.95425	0.84416
C	4.34376	2.68721	0.26369
C	5.57220	2.60676	1.19422
C	6.81366	3.30750	0.61603
C	2.23094	-2.66373	0.81680
C	3.26815	-3.79335	0.65992
C	3.39164	-4.65650	1.93393
C	4.42917	-5.78467	1.80298
C	3.37359	-0.94120	-1.30724
C	4.25783	-1.86595	-2.17042
C	5.58057	-1.18785	-2.58541
C	6.46724	-2.08696	-3.46381
C	0.63715	3.45091	0.66710
C	1.41077	4.77994	0.74963
C	0.59281	5.90073	1.42629
C	1.35115	7.23653	1.50802
C	1.98816	2.63050	-1.77746
C	0.84681	2.84807	-2.78525
C	1.34764	3.38940	-4.14003
C	0.21504	3.59425	-5.16022
C	-1.16172	-2.01136	6.98618
H	-0.11480	0.10695	-1.66351
H	1.27405	-3.08851	1.18197
H	2.55805	-1.96493	1.61139
H	2.99989	-4.44947	-0.19111
H	4.26232	-3.36564	0.42412
H	3.65573	-4.00500	2.79140
H	2.40009	-5.09011	2.17409
H	4.49209	-6.38777	2.72705
H	4.17109	-6.46877	0.97330
H	5.43791	-5.37973	1.59796

H	2.11596	-3.53754	-2.05214
H	0.42675	-3.44059	-1.48976
H	-0.13368	-1.72470	-3.13970
H	1.56003	-1.55394	-3.66248
H	1.52965	-3.95557	-4.48348
H	-0.15804	-4.09886	-3.94546
H	-0.05399	-3.62257	-6.42373
H	-0.86491	-2.29565	-5.53519
H	0.82318	-2.10061	-6.10355
H	3.95236	-0.58565	-0.43206
H	3.12209	-0.03948	-1.89391
H	4.48911	-2.80309	-1.63025
H	3.70696	-2.16499	-3.08270
H	5.35322	-0.24798	-3.12783
H	6.14035	-0.88877	-1.67597
H	7.40361	-1.57559	-3.75185
H	6.74265	-3.01742	-2.93306
H	5.94338	-2.37741	-4.39324
H	-0.31037	3.60895	0.11909
H	0.33782	3.12655	1.68364
H	2.35493	4.64108	1.31282
H	1.70234	5.11654	-0.26522
H	-0.35554	6.04383	0.87071
H	0.30330	5.57427	2.44553
H	0.74362	8.01952	1.99719
H	2.28814	7.12982	2.08611
H	1.62302	7.60399	0.50085
H	2.88824	2.36719	1.84814
H	3.38354	0.89322	1.00644
H	4.60998	2.25064	-0.71859
H	4.10633	3.75175	0.07435
H	5.31640	3.05422	2.17612
H	5.80770	1.54217	1.39557
H	7.67632	3.23592	1.30296
H	7.11379	2.85582	-0.34762
H	6.61830	4.38069	0.43348
H	2.71314	1.91552	-2.21116
H	2.54135	3.57709	-1.62366
H	0.32370	1.88504	-2.93826
H	0.09837	3.55349	-2.36990
H	1.88403	4.34724	-3.98147
H	2.09545	2.68499	-4.55661
H	0.59966	3.97993	-6.12185
H	-0.31100	2.64386	-5.36580
H	-0.53427	4.31709	-4.78552
H	-4.84913	-1.32163	-0.04201
H	-4.02435	0.23755	-0.28513
H	-4.03629	0.62020	-3.47122
H	-4.94956	-0.75935	-4.12790
H	-6.12891	-1.11378	-2.04315
H	-5.85276	0.63248	-1.81544
H	1.93677	0.44804	2.64323
H	1.94119	0.04298	5.06191
H	-1.72753	-1.41759	2.13125
H	-1.92855	-1.91187	4.54547
H	0.82181	-1.10842	8.33293
H	1.96947	-1.46712	7.00473
H	1.32678	0.19368	7.22505
H	-1.05948	-2.06327	8.08078
H	-2.14973	-1.56719	6.75993
H	-1.14988	-3.04637	6.58524
H	-0.69858	-2.59408	0.39862
C	-2.76076	-3.67576	-0.66348
C	-4.10726	-4.00942	0.02146
H	-2.80767	-4.04126	-1.71215
H	-1.95955	-4.25692	-0.16586
C	-4.40478	-5.52105	0.05741
H	-4.10226	-3.61199	1.05793
H	-4.93567	-3.49716	-0.50841
C	-5.74265	-5.86265	0.73525
H	-4.40226	-5.91672	-0.97809
H	-3.57939	-6.04156	0.58415
H	-5.92547	-6.95273	0.75112
H	-5.76333	-5.50410	1.78183
H	-6.59101	-5.38894	0.20638
C	-2.18560	1.89841	-0.97337
C	-2.24053	1.64704	1.31084
C	-3.20716	2.84698	-0.94459
H	-1.73860	1.58577	-1.91898
C	-3.27301	2.57774	1.44320
H	-1.84116	1.15129	2.19952
C	-3.78737	3.23984	0.29515
H	-3.54731	3.26991	-1.89324
H	-3.66300	2.78180	2.44378
N	-1.66401	1.29400	0.13137
N	-4.78128	4.19772	0.37537
C	-5.43926	4.44841	1.65341
H	-4.70792	4.77058	2.41896
H	-6.17346	5.25876	1.52826
H	-5.97086	3.55342	2.03958
C	-5.36694	4.73744	-0.84796
H	-4.59581	5.22076	-1.47732
H	-5.87085	3.95833	-1.45763
H	-6.11168	5.50314	-0.58266

[7m-1]‡

148

XYZ

C	-2.16428	-1.25326	1.68131
N	-0.83101	-0.97851	1.70030
C	-0.26451	-0.93991	2.94097
C	-0.96544	-1.09702	4.13643
C	-2.37056	-1.33637	4.11404
C	-2.95195	-1.43429	2.82096

Ru	0.24593	-0.05526	-0.04478
C	-2.67983	-1.27363	-1.96636
C	-1.34687	-1.19773	-1.64549
N	-3.11661	-1.46535	5.27724
C	-2.42992	-1.57238	6.56073
P	1.61224	-1.91401	-0.31810
C	1.84811	-2.57029	-2.07899
C	2.36635	-1.55154	-3.10855
C	2.43703	-2.14065	-4.53216
C	2.96730	-1.14436	-5.57629
N	-3.52279	-0.10768	-2.08260
C	-3.76518	0.51480	-3.29938
C	-4.79114	1.63775	-3.04275
C	-5.42118	1.27649	-1.68456
C	-4.32746	0.43661	-0.97493
O	-3.24102	0.22977	-4.37846
P	1.96740	1.41887	0.30460
C	3.01362	1.14187	1.89769
C	4.52098	1.46708	1.95044
C	5.13147	1.21391	3.34532
C	6.64003	1.50805	3.41056
C	0.87072	-3.45375	0.49900
C	1.54848	-4.83348	0.38850
C	0.70036	-5.95469	1.02664
C	1.36728	-7.33834	0.94497
C	3.39406	-1.96462	0.34449
C	4.44340	-2.88951	-0.30718
C	5.79954	-2.84295	0.42782
C	6.87308	-3.72459	-0.23258
C	1.47540	3.22803	0.60946
C	2.56121	4.27111	0.93356
C	1.98358	5.69489	1.08568
C	3.05151	6.74866	1.42539
C	3.33337	1.69346	-0.99385
C	2.80900	2.34723	-2.28431
C	3.86045	2.40710	-3.41035
C	3.34082	3.10018	-4.68135
C	-4.50108	-1.91881	5.18925
H	-0.70294	-0.26499	-1.80594
H	-0.16109	-3.50802	0.09890
H	0.75043	-3.18374	1.56527
H	1.73831	-5.09117	-0.67218
H	2.53769	-4.81135	0.88664
H	0.49919	-5.70015	2.08671
H	-0.28897	-5.98856	0.52794
H	0.73724	-8.12002	1.40691
H	1.54968	-7.63273	-0.10545
H	2.34327	-7.34384	1.46554
H	2.51399	-3.45375	-2.04892
H	0.85911	-2.94175	-2.41024
H	1.70568	-0.66315	-3.09727
H	3.37342	-1.19034	-2.81352
H	3.07801	-3.04597	-4.52493
H	1.42565	-2.48278	-4.82958
H	2.99759	-1.59334	-6.58578
H	2.32675	-0.24488	-5.62883
H	3.99233	-0.81040	-5.32768
H	3.30204	-2.20387	1.42379
H	3.77025	-0.92775	0.29985
H	4.08694	-3.93619	-0.33488
H	4.59958	-2.58962	-1.36201
H	6.15535	-1.79379	0.46996
H	5.65503	-3.15884	1.48075
H	7.83242	-3.67169	0.31332
H	6.55985	-4.78502	-0.25817
H	7.06292	-3.40910	-1.27541
H	0.90898	3.55280	-0.28350
H	0.73771	3.20046	1.43408
H	3.08340	3.99934	1.87177
H	3.33514	4.28496	0.14033
H	1.46661	5.97867	0.14694
H	1.20357	5.68830	1.87369
H	2.61070	7.75721	1.52687
H	3.56126	6.50877	2.37731
H	3.82674	6.79993	0.63818
H	2.47986	1.71920	2.68118
H	2.86948	0.07912	2.16283
H	5.06419	0.84925	1.20702
H	4.70661	2.52000	1.66410
H	4.59923	1.83623	4.09313
H	4.94584	0.16060	3.63738
H	7.04807	1.31789	4.42002
H	7.20132	0.87548	2.69779
H	6.85293	2.56322	3.15630
H	3.74553	0.69447	-1.23500
H	4.16624	2.29365	-0.57840
H	1.91714	1.78680	-2.63259
H	2.46228	3.37819	-2.06738
H	4.76397	2.93541	-3.04367
H	4.18610	1.37700	-3.65755
H	4.11035	3.12572	-5.47425
H	2.45690	2.57506	-5.08845
H	3.03987	4.14421	-4.47256
H	-4.75393	-0.38370	-0.36963
H	-3.70173	1.05713	-0.30667
H	-4.24542	2.59993	-3.00133
H	-5.50672	1.69687	-3.87922
H	-6.32291	0.65675	-1.84292
H	-5.71735	2.15373	-1.08451
H	0.81488	-0.76044	2.95109
H	-0.40994	-1.04334	5.07643
H	-2.60559	-1.33561	0.68071
H	-4.01284	-1.66441	2.68760
H	-3.17954	-1.63343	7.36488

H	-1.77435	-2.46714	6.62596
H	-1.80834	-0.67737	6.74971
H	-4.94256	-1.92376	6.19792
H	-5.09950	-1.23035	4.56382
H	-4.59373	-2.94009	4.76159
H	-0.82136	-2.15268	-1.73396
C	-3.34074	-2.57275	-2.40421
C	-4.57679	-3.02317	-1.59119
H	-3.64521	-2.45820	-3.46728
H	-2.58306	-3.37993	-2.38191
C	-5.17997	-4.34592	-2.10232
H	-4.29613	-3.13491	-0.52340
H	-5.35570	-2.23511	-1.63253
C	-6.41413	-4.79847	-1.30404
H	-5.44987	-4.23230	-3.17135
H	-4.40469	-5.13760	-2.06587
H	-6.82301	-5.74900	-1.69225
H	-6.16584	-4.95186	-0.23694
H	-7.22070	-4.04288	-1.35223
C	-1.40985	2.38432	-1.00012
C	-1.68241	2.03733	1.26210
C	-2.20159	3.53186	-0.97095
H	-0.94066	2.05603	-1.93240
C	-2.47706	3.17759	1.39263
H	-1.44724	1.42940	2.13974
C	-2.77036	3.98009	0.25583
H	-2.34226	4.08496	-1.90286
H	-2.85099	3.43630	2.38652
N	-1.12760	1.61554	0.08968
N	-3.55463	5.11923	0.33464
C	-3.93417	5.63706	1.64574
H	-3.05585	5.92002	2.26384
H	-4.56542	6.52874	1.51124
H	-4.52430	4.88985	2.20894
C	-3.65754	6.00119	-0.82480
H	-2.67784	6.42943	-1.12484
H	-4.07756	5.46156	-1.69392
H	-4.34017	6.83089	-0.58547

8az

129

XYZ

C	1.38541	-1.56091	1.77381
N	1.20769	-1.49053	0.43023
C	1.90949	-2.40127	-0.29246
C	2.74885	-3.37207	0.25329
C	2.92135	-3.45139	1.66322
C	2.20220	-2.48721	2.42283
Ru	-0.08380	0.06717	-0.67471
C	2.87701	1.15884	-1.20646
C	1.68230	0.63849	-1.60215
N	3.73109	-4.40237	2.25590
C	4.55026	-5.27607	1.42196
P	0.01282	1.95354	0.63750
C	0.55266	3.50341	-0.28995
C	-0.40036	3.95916	-1.40875
C	0.12715	5.19366	-2.16866
C	-0.82488	5.66050	-3.28262
N	-0.09216	-1.06911	-2.54413
C	0.80707	-0.61370	-3.38248
C	0.71186	-1.12863	-4.80108
C	-0.71050	-1.73742	-4.79344
C	-0.99034	-1.98192	-3.28229
O	1.74497	0.24665	-3.03473
P	-2.05870	-0.94133	0.24681
C	-2.23056	-1.10905	2.13129
C	-3.47282	-1.80810	2.71702
C	-3.40746	-1.94909	4.25267
C	-4.64853	-2.63491	4.84871
C	1.19536	1.96542	2.13307
C	1.87774	3.26728	2.60361
C	2.76175	3.04727	3.84943
C	3.46125	4.33127	4.32710
C	-1.60022	2.57393	1.40594
C	-1.67041	3.95816	2.08003
C	-3.09965	4.30904	2.54626
C	-3.19262	5.68405	3.22898
C	-2.20086	-2.79934	-0.17633
C	-3.55074	-3.38314	-0.63664
C	-3.46197	-4.88903	-0.96185
C	-4.79563	-5.48122	-1.44849
C	-3.76523	-0.23610	-0.16319
C	-3.96231	0.17412	-1.63350
C	-5.27930	0.93882	-1.87321
C	-5.47677	1.34979	-3.34217
C	3.96807	-4.35427	3.69491
H	2.93484	1.47562	-0.15549
H	1.98059	1.22634	1.88431
H	0.61769	1.52316	2.97162
H	2.50410	3.67479	1.78615
H	1.12555	4.04669	2.83177
H	2.13919	2.63673	4.67035
H	3.52110	2.27178	3.62392
H	4.08386	4.14545	5.22109
H	4.11917	4.74432	3.54006
H	2.72444	5.11374	4.58847
H	0.70099	4.32444	0.43732
H	1.53680	3.26147	-0.72901
H	-0.54847	3.11992	-2.11369
H	-1.40078	4.19640	-0.99119
H	0.29746	6.02578	-1.45499
H	1.11800	4.95425	-2.60331
H	-0.42541	6.54233	-3.81611
H	-0.98612	4.86036	-4.02879

H	-1.81485	5.93652	-2.87295
H	-1.91013	1.80408	2.13714
H	-2.33715	2.52236	0.58147
H	-0.99260	3.99016	2.95520
H	-1.31871	4.74435	1.38433
H	-3.78214	4.28171	1.67317
H	-3.46072	3.52402	3.24107
H	-4.22652	5.90849	3.54807
H	-2.54764	5.73055	4.12637
H	-2.86991	6.49151	2.54566
H	-1.43526	-2.98975	-0.94636
H	-1.85421	-3.34411	0.72453
H	-4.32503	-3.22927	0.14039
H	-3.91102	-2.84442	-1.53554
H	-2.68167	-5.04810	-1.73319
H	-3.11693	-5.43702	-0.06198
H	-4.70152	-6.55835	-1.67677
H	-5.58667	-5.36883	-0.68377
H	-5.14653	-4.97347	-2.36621
H	-1.31783	-1.65080	2.44829
H	-2.14413	-0.09457	2.56405
H	-4.38726	-1.24384	2.44841
H	-3.58807	-2.81563	2.27115
H	-2.49850	-2.52178	4.52679
H	-3.28420	-0.94485	4.70585
H	-4.57400	-2.72478	5.94756
H	-5.56825	-2.06458	4.62075
H	-4.77872	-3.65322	4.43700
H	-3.86113	0.66282	0.47839
H	-4.56266	-0.93510	0.15335
H	-3.09791	0.79005	-1.94970
H	-3.94925	-0.72743	-2.28015
H	-6.13681	0.31770	-1.54315
H	-5.29294	1.84344	-1.23196
H	-6.41986	1.90818	-3.48433
H	-4.64818	1.99429	-3.68954
H	-5.50957	0.46428	-4.00445
H	-0.77472	-3.03295	-2.99695
H	-2.03896	-1.77575	-3.00886
H	0.85428	-0.32241	-5.54141
H	1.49931	-1.88582	-4.99033
H	-0.79741	-2.65626	-5.39769
H	-1.43260	-1.00250	-5.19155
H	1.77636	-2.33502	-1.37663
H	3.26986	-4.05389	-0.42395
H	0.83696	-0.81461	2.35521
H	2.27948	-2.44423	3.51222
H	5.11063	-5.96893	2.06786
H	3.92155	-5.88240	0.74183
H	5.27841	-4.70997	0.80452
H	4.61079	-5.19991	3.98298
H	4.46731	-3.41448	4.01091
H	3.01894	-4.44431	4.25748
H	-0.95668	1.09184	-1.57885
C	4.15535	1.32132	-2.00160
C	5.34733	0.54875	-1.38743
H	3.99900	0.98995	-3.04381
H	4.43577	2.39731	-2.05434
C	6.67570	0.76559	-2.13643
H	5.47252	0.85385	-0.32738
H	5.10089	-0.53197	-1.36481
C	7.85671	0.00084	-1.51462
H	6.55294	0.45957	-3.19512
H	6.90859	1.84957	-2.15860
H	8.79601	0.17718	-2.07023
H	8.02439	0.31066	-0.46589
H	7.66894	-1.08946	-1.51131

[8az-10z]‡

129

XYZ

C	1.13371	1.10483	2.30039
N	1.44825	0.19057	1.34732
C	2.65572	-0.41899	1.49879
C	3.53965	-0.14678	2.54492
C	3.20660	0.80812	3.54291
C	1.94312	1.44395	3.38386
Ru	0.07435	-0.18027	-0.53049
C	2.56441	-0.20505	-2.56907
C	1.51779	-0.24405	-1.73805
N	4.04627	1.09563	4.60190
C	5.37673	0.49634	4.65263
P	-0.42042	2.04054	-0.79780
C	-0.74882	2.51703	-2.58614
C	-2.06596	1.99184	-3.18377
C	-2.20302	2.32021	-4.68460
C	-3.52486	1.81932	-5.29007
N	0.82059	-2.22973	-1.30557
C	1.77118	-3.13506	-0.89404
C	1.54466	-4.48636	-1.62005
C	0.66942	-4.11905	-2.82677
C	-0.04446	-2.82834	-2.34377
O	2.68352	-2.96583	-0.06270
P	-1.73918	-1.04802	0.88024
C	-2.02351	-0.29985	2.59208
C	-3.00256	-0.95542	3.58638
C	-3.16283	-0.12866	4.88014
C	-4.11940	-0.77705	5.89508
C	1.00244	3.20180	-0.32467
C	1.27066	4.47967	-1.14604
C	2.44507	5.29971	-0.57181
C	2.75721	6.56138	-1.39430
C	-1.90122	2.80054	0.08969
C	-2.24906	4.28443	-0.13787

C	-3.52432	4.71702	0.61629
C	-3.87560	6.19942	0.40528
C	-1.36725	-2.85512	1.27503
C	-2.51775	-3.84202	1.55818
C	-1.99869	-5.25634	1.89271
C	-3.12914	-6.26824	2.14487
C	-3.50644	-1.06577	0.23126
C	-3.68609	-1.63423	-1.18742
C	-5.14459	-1.55231	-1.68083
C	-5.33247	-2.12430	-3.09598
C	3.70208	2.16498	5.53225
H	2.91702	0.83280	-2.71451
H	1.90072	2.55816	-0.34316
H	0.84688	3.46867	0.73917
H	1.50258	4.20986	-2.19474
H	0.36919	5.12189	-1.18440
H	2.21312	5.58512	0.47416
H	3.34575	4.65606	-0.52037
H	3.60017	7.12852	-0.95993
H	3.02827	6.30310	-2.43484
H	1.88388	7.23883	-1.43650
H	-0.71024	3.61877	-2.68267
H	0.10608	2.10392	-3.15475
H	-2.11716	0.89615	-3.03767
H	-2.93017	2.42585	-2.64018
H	-2.11967	3.41608	-4.83283
H	-1.35020	1.87061	-5.23102
H	-3.59364	2.05879	-6.36664
H	-3.62137	0.72275	-5.18383
H	-4.39608	2.28013	-4.78786
H	-1.73006	2.61876	1.16948
H	-2.76314	2.16176	-0.18690
H	-1.40483	4.92270	0.18897
H	-2.38696	4.48096	-1.21944
H	-4.37257	4.08342	0.28807
H	-3.39324	4.51635	1.69876
H	-4.79175	6.47981	0.95548
H	-3.05921	6.85802	0.75586
H	-4.04600	6.42145	-0.66457
H	-0.76296	-3.21590	0.42322
H	-0.66509	-2.82393	2.13206
H	-3.15043	-3.48481	2.39281
H	-3.18368	-3.90677	0.67518
H	-1.35757	-5.61343	1.06219
H	-1.34078	-5.20226	2.78303
H	-2.72861	-7.27056	2.38096
H	-3.76676	-5.95410	2.99238
H	-3.78162	-6.36871	1.25745
H	-1.01949	-0.23969	3.05326
H	-2.33428	0.74851	2.40632
H	-3.99934	-1.08903	3.12194
H	-2.64246	-1.96763	3.85297
H	-2.16716	0.01395	5.34605
H	-3.52820	0.88541	4.62038
H	-4.21654	-0.16306	6.80858
H	-5.13129	-0.90318	5.46695
H	-3.76026	-1.77743	6.20038
H	-3.84098	-0.00878	0.24918
H	-4.15358	-1.61388	0.94322
H	-3.01814	-1.09000	-1.88162
H	-3.35509	-2.69204	-1.21444
H	-5.80508	-2.09210	-0.97222
H	-5.47576	-0.49428	-1.66194
H	-6.38448	-2.05148	-3.42614
H	-4.71103	-1.58016	-3.83116
H	-5.04031	-3.19013	-3.13861
H	-1.03933	-3.06423	-1.90923
H	-0.22065	-2.10130	-3.15429
H	2.51228	-4.95429	-1.86725
H	1.02116	-5.16769	-0.91946
H	-0.04457	-4.90770	-3.12368
H	1.30254	-3.89893	-3.70638
H	2.88411	-1.19813	0.76050
H	4.48446	-0.69580	2.57345
H	0.16425	1.59919	2.17848
H	1.58844	2.20033	4.08899
H	5.88629	0.82757	5.57022
H	5.31587	-0.60840	4.67759
H	6.00174	0.78617	3.78288
H	4.47736	2.22970	6.31071
H	3.63019	3.15345	5.03196
H	2.73573	1.96382	6.03412
H	-0.91394	-0.35280	-1.79114
C	3.38080	-1.28134	-3.25259
C	4.77475	-1.48754	-2.61245
H	2.82403	-2.23415	-3.22614
H	3.51051	-1.01779	-4.32395
C	5.64329	-2.50506	-3.37602
H	5.30533	-0.51414	-2.56401
H	4.62696	-1.82519	-1.56976
C	7.02295	-2.72177	-2.73150
H	5.10636	-3.47359	-3.42949
H	5.77244	-2.16776	-4.42496
H	7.62489	-3.45768	-3.29548
H	7.59808	-1.77746	-2.69279
H	6.92212	-3.09260	-1.69478

10z

129

XYZ

C	1.27784	-2.00203	1.60366
N	1.39809	-1.28455	0.45538
C	2.58382	-1.42263	-0.20431
C	3.62245	-2.24774	0.22940

C	3.49676	-2.99395	1.43278
C	2.26399	-2.83568	2.12747
Ru	-0.21135	0.06113	-0.39950
C	2.19829	2.06628	0.05267
C	1.24249	1.50270	-0.73973
N	4.50152	-3.82207	1.89785
C	5.79563	-3.83598	1.22118
P	-0.99936	1.17151	1.33731
C	-1.14294	3.04154	1.17585
C	-2.24316	3.53386	0.21928
C	-2.20824	5.06234	0.01477
C	-3.32287	5.56834	-0.91634
N	1.28562	1.89458	-2.15277
C	1.81193	1.13154	-3.16335
C	1.45131	1.79319	-4.50320
C	0.95757	3.20006	-4.11477
C	0.49304	3.03064	-2.64753
O	2.44844	0.06630	-3.03181
P	-1.76938	-1.74195	-0.67728
C	-2.08120	-3.05785	0.65412
C	-2.85764	-4.33884	0.28835
C	-2.95922	-5.33061	1.46676
C	-3.72928	-6.61309	1.10867
C	0.01678	0.95024	2.92745
C	0.21021	2.13317	3.89939
C	1.02960	1.73236	5.14391
C	1.24517	2.89828	6.12372
C	-2.73162	0.71416	1.92515
C	-3.32150	1.44975	3.14511
C	-4.75493	0.98304	3.47726
C	-5.35052	1.69330	4.70455
C	-1.10362	-2.85169	-2.05204
C	-0.37185	-2.09372	-3.17675
C	0.28583	-3.03889	-4.20250
C	0.99894	-2.28212	-5.33567
C	-3.54328	-1.42798	-1.23908
C	-3.68809	-0.55427	-2.49818
C	-5.15215	-0.16979	-2.79234
C	-5.30651	0.67912	-4.06537
C	4.37635	-4.44276	3.21262
H	2.18911	1.79136	1.11886
H	1.00622	0.59328	2.58914
H	-0.44738	0.09852	3.46557
H	0.72778	2.96197	3.37872
H	-0.76764	2.53534	4.22906
H	0.51579	0.89918	5.66490
H	2.01087	1.33218	4.81940
H	1.83136	2.58200	7.00540
H	1.78929	3.73101	5.64077
H	0.28073	3.29826	6.48858
H	-1.29962	3.47942	2.18019
H	-0.15201	3.37469	0.81713

H	-2.13172	3.02365	-0.75623
H	-3.24184	3.24926	0.60870
H	-2.28753	5.56944	0.99774
H	-1.22038	5.34943	-0.39789
H	-3.26950	6.66341	-1.05511
H	-3.25189	5.09916	-1.91518
H	-4.32340	5.33086	-0.50876
H	-2.70754	-0.37489	2.12641
H	-3.38644	0.84927	1.04362
H	-2.67839	1.28562	4.03200
H	-3.33244	2.54303	2.96656
H	-5.40592	1.15359	2.59658
H	-4.75075	-0.11242	3.64717
H	-6.37456	1.33814	4.91893
H	-4.73745	1.51391	5.60743
H	-5.40103	2.78698	4.54861
H	-0.39003	-3.54320	-1.56037
H	-1.91912	-3.47647	-2.46858
H	-1.07540	-1.41599	-3.69766
H	0.41751	-1.44339	-2.74616
H	1.01414	-3.68723	-3.67462
H	-0.48392	-3.71840	-4.62347
H	1.50826	-2.97714	-6.02830
H	0.27788	-1.68935	-5.93054
H	1.75150	-1.58654	-4.92202
H	-1.07512	-3.33234	1.02762
H	-2.58791	-2.54228	1.49450
H	-3.88099	-4.08333	-0.05010
H	-2.36613	-4.84634	-0.56451
H	-1.93815	-5.59413	1.80907
H	-3.45013	-4.82907	2.32511
H	-3.78236	-7.30675	1.96717
H	-4.76570	-6.38381	0.79827
H	-3.24307	-7.14995	0.27292
H	-4.05401	-0.93642	-0.38692
H	-4.04954	-2.40058	-1.39659
H	-3.07346	0.35937	-2.37826
H	-3.27710	-1.09441	-3.37432
H	-5.76517	-1.08942	-2.88505
H	-5.56277	0.38504	-1.92452
H	-6.36312	0.94532	-4.24992
H	-4.73090	1.62033	-3.98956
H	-4.93777	0.13508	-4.95503
H	-0.58544	2.78668	-2.56761
H	0.68738	3.92466	-2.02769
H	2.31769	1.78537	-5.18550
H	0.65632	1.18559	-4.97826
H	0.14994	3.58029	-4.76345
H	1.79341	3.92154	-4.16230
H	2.68356	-0.84042	-1.12987
H	4.53208	-2.29006	-0.37476
H	0.32545	-1.88514	2.12941

H	2.06629	-3.35371	3.06937
H	6.45285	-4.56621	1.71735
H	5.68698	-4.14221	0.16360
H	6.29486	-2.84502	1.24328
H	5.26081	-5.07020	3.40069
H	4.30128	-3.69448	4.02943
H	3.48381	-5.09555	3.26178
H	-1.26713	1.01096	-1.21008
C	3.32656	2.99127	-0.35423
C	4.72305	2.34014	-0.20363
H	3.19251	3.31114	-1.40534
H	3.30482	3.91339	0.26775
C	5.88439	3.29045	-0.55100
H	4.84637	1.97489	0.83719
H	4.76439	1.44362	-0.85267
C	7.26786	2.63366	-0.40766
H	5.75640	3.65795	-1.58923
H	5.83092	4.18766	0.09897
H	8.08173	3.33673	-0.66302
H	7.43882	2.28509	0.62834
H	7.36167	1.75534	-1.07328

[10z-11z]‡

129

XYZ

C	0.27414	-2.74278	1.57923
N	0.87345	-2.08902	0.54159
C	2.21601	-2.31199	0.41755
C	2.95890	-3.12492	1.26977
C	2.33315	-3.78394	2.36535
C	0.93584	-3.55825	2.49760
Ru	-0.02557	-0.12847	-0.08048
C	2.75278	1.20215	-1.16463
C	1.73038	0.33204	-1.44005
N	3.03360	-4.59764	3.23269
C	4.48406	-4.71411	3.10674
P	-0.27205	1.89974	0.83278
C	0.08371	3.47456	-0.15017
C	-1.03564	3.88893	-1.12210
C	-0.65343	5.11205	-1.98115
C	-1.77348	5.55158	-2.93876
N	1.82511	-0.50672	-2.61851
C	2.82747	-1.42227	-2.90127
C	2.40030	-2.22125	-4.14449
C	1.24901	-1.40022	-4.74815
C	0.67070	-0.65014	-3.52762
O	3.86722	-1.61074	-2.25302
P	-2.22048	-0.79892	-0.01976
C	-3.28161	-1.02948	1.55623
C	-4.63703	-1.76484	1.52918
C	-5.35752	-1.72739	2.89447
C	-6.70171	-2.47528	2.89085
C	1.03743	1.93566	2.21566
C	1.82865	3.22941	2.50349
C	2.82625	3.05353	3.66760
C	3.64375	4.32386	3.95718
C	-1.82079	2.41150	1.78330
C	-1.81254	3.73932	2.56607
C	-3.15775	4.01777	3.27004
C	-3.16055	5.33534	4.06356
C	-2.31771	-2.53440	-0.79761
C	-3.56553	-2.96509	-1.59693
C	-3.46613	-4.42497	-2.08812
C	-4.68528	-4.86696	-2.91537
C	-3.46733	0.18835	-1.04513
C	-3.00452	0.52957	-2.47079
C	-3.97110	1.47570	-3.21040
C	-3.52830	1.78164	-4.65125
C	2.36682	-5.14641	4.40902
H	2.64587	1.79332	-0.24384
H	1.74314	1.12381	1.95569
H	0.51158	1.59898	3.13159
H	2.38683	3.53809	1.59771
H	1.14115	4.06471	2.74125
H	2.27245	2.75114	4.57925
H	3.51164	2.21484	3.43319
H	4.34892	4.16888	4.79370
H	4.23438	4.62869	3.07334
H	2.98583	5.17137	4.22614
H	0.31042	4.30888	0.54139
H	1.00752	3.26712	-0.72338
H	-1.27995	3.03575	-1.78472
H	-1.96313	4.12198	-0.56028
H	-0.38325	5.95640	-1.31523
H	0.26093	4.87525	-2.56080
H	-1.46899	6.42441	-3.54426
H	-2.04508	4.73787	-3.63672
H	-2.68819	5.83199	-2.38353
H	-2.02157	1.57064	2.47431
H	-2.65239	2.41346	1.05207
H	-1.00793	3.72354	3.32707
H	-1.58174	4.58370	1.88694
H	-3.96703	4.03808	2.51276
H	-3.39478	3.17425	3.94899
H	-4.13443	5.50865	4.55589
H	-2.38346	5.32989	4.85048
H	-2.95984	6.20006	3.40395
H	-1.42331	-2.61271	-1.44429
H	-2.14749	-3.25339	0.02863
H	-4.48069	-2.85349	-0.98614
H	-3.69942	-2.30319	-2.47425
H	-2.54398	-4.54394	-2.69175
H	-3.34671	-5.09654	-1.21406
H	-4.58719	-5.91467	-3.25267

H	-5.61808	-4.79055	-2.32597
H	-4.80837	-4.23550	-3.81502
H	-2.61332	-1.52164	2.29126
H	-3.43180	-0.00203	1.94282
H	-5.30324	-1.31986	0.76362
H	-4.48697	-2.82240	1.23533
H	-4.69282	-2.16138	3.66852
H	-5.52043	-0.67132	3.18942
H	-7.19378	-2.42909	3.87923
H	-7.39837	-2.04165	2.14931
H	-6.56538	-3.54245	2.63407
H	-3.64823	1.12614	-0.48300
H	-4.43292	-0.35343	-1.07447
H	-1.99355	0.97966	-2.41171
H	-2.88743	-0.40064	-3.06287
H	-4.98719	1.03180	-3.22320
H	-4.05802	2.42285	-2.64082
H	-4.23429	2.46480	-5.15731
H	-2.52974	2.25728	-4.66938
H	-3.46572	0.85710	-5.25542
H	-0.13740	-1.21813	-3.02363
H	0.26375	0.34308	-3.78515
H	3.26084	-2.37498	-4.81635
H	2.06532	-3.22209	-3.80668
H	0.48451	-2.01083	-5.25791
H	1.64423	-0.67352	-5.48098
H	2.72691	-1.79314	-0.39726
H	4.02853	-3.22839	1.07192
H	-0.80235	-2.58699	1.67710
H	0.35396	-4.01891	3.30001
H	4.85507	-5.42279	3.86259
H	4.76738	-5.10187	2.10999
H	4.99845	-3.74270	3.25722
H	3.08113	-5.76460	4.97348
H	1.98551	-4.35220	5.08389
H	1.51395	-5.79145	4.12168
H	0.42842	0.77133	-1.42071
C	4.02294	1.47269	-1.93651
C	5.30552	1.09078	-1.15916
H	4.01231	0.94090	-2.90386
H	4.06910	2.56081	-2.16796
C	6.59875	1.43709	-1.91973
H	5.30740	1.60861	-0.17708
H	5.26780	0.00547	-0.95653
C	7.87274	1.06425	-1.14271
H	6.59465	0.91070	-2.89503
H	6.61223	2.52137	-2.15455
H	8.78623	1.31635	-1.71202
H	7.92239	1.59933	-0.17554
H	7.90104	-0.01950	-0.92452

11z

129

XYZ

C	-0.48884	-2.56903	-2.25494
N	-0.85527	-1.74162	-1.22866
C	-2.20102	-1.67648	-0.97934
C	-3.17134	-2.36055	-1.70955
C	-2.78728	-3.21868	-2.77665
C	-1.38762	-3.29478	-3.03445
Ru	0.30516	-0.12623	-0.54786
C	-2.91484	1.55635	1.67042
C	-2.14765	0.45634	1.83831
N	-3.71261	-3.94758	-3.50525
C	-5.13762	-3.69770	-3.30721
P	0.93424	2.04030	-0.66608
C	0.77459	3.34524	0.70688
C	1.88794	3.31114	1.76933
C	1.68464	4.37122	2.87147
C	2.80712	4.37973	3.92294
N	-2.53241	-0.72556	2.52528
C	-3.64731	-1.51069	2.22267
C	-3.46042	-2.86687	2.92267
C	-2.28676	-2.65071	3.89487
C	-1.50081	-1.48114	3.25949
O	-4.58523	-1.19402	1.48521
P	2.19752	-1.10028	-0.00672
C	3.56845	-1.01334	-1.32061
C	4.85389	-1.85561	-1.19449
C	5.86197	-1.56316	-2.32694
C	7.13237	-2.42625	-2.24352
C	-0.38294	2.61932	-1.91947
C	-1.04504	4.00657	-1.77937
C	-2.08721	4.27145	-2.88596
C	-2.76392	5.64698	-2.75969
C	2.53998	2.60182	-1.48865
C	2.68504	4.06561	-1.94716
C	4.03972	4.33927	-2.63484
C	4.19421	5.79510	-3.10630
C	2.01887	-2.97807	0.26116
C	3.01509	-3.73899	1.16090
C	2.71704	-5.25265	1.21213
C	3.68092	-6.02714	2.12707
C	3.17611	-0.55484	1.51470
C	2.37215	-0.54899	2.82586
C	3.19983	-0.08231	4.04020
C	2.40008	-0.08926	5.35409
C	-3.28136	-4.67207	-4.69575
H	-2.46599	2.35367	1.05932
H	-1.17920	1.84405	-1.89420
H	0.08635	2.52206	-2.91782
H	-1.54043	4.09169	-0.79230
H	-0.27591	4.80297	-1.80853
H	-1.59590	4.18784	-3.87618

H	-2.85645	3.47402	-2.85758
H	-3.50071	5.81112	-3.56667
H	-3.29657	5.74345	-1.79530
H	-2.02102	6.46472	-2.81247
H	0.71897	4.35873	0.26266
H	-0.20061	3.15365	1.19499
H	1.92792	2.30469	2.22951
H	2.87548	3.47689	1.29217
H	1.61071	5.37307	2.40225
H	0.70986	4.19425	3.36826
H	2.63190	5.14930	4.69651
H	2.88382	3.40253	4.43521
H	3.78889	4.58960	3.45846
H	2.66843	1.91749	-2.34976
H	3.35461	2.35072	-0.77964
H	1.86966	4.32263	-2.65226
H	2.57651	4.75097	-1.08325
H	4.86090	4.08571	-1.93463
H	4.15204	3.65441	-3.49903
H	5.17129	5.96073	-3.59509
H	3.40586	6.06533	-3.83348
H	4.11999	6.50084	-2.25799
H	0.99284	-3.11328	0.65004
H	2.01588	-3.42930	-0.75166
H	4.05382	-3.59000	0.81161
H	2.97776	-3.33377	2.19095
H	1.67407	-5.40636	1.55515
H	2.76402	-5.66821	0.18530
H	3.44136	-7.10574	2.14747
H	4.72772	-5.92277	1.78571
H	3.63215	-5.65322	3.16686
H	3.05732	-1.24782	-2.27405
H	3.82787	0.06090	-1.38476
H	5.34776	-1.66916	-0.22003
H	4.60231	-2.93424	-1.21582
H	5.36556	-1.72610	-3.30467
H	6.13797	-0.49000	-2.29991
H	7.83290	-2.19629	-3.06665
H	7.66865	-2.25900	-1.29071
H	6.88789	-3.50338	-2.30240
H	3.52927	0.47180	1.29331
H	4.07888	-1.18892	1.62316
H	1.48426	0.09974	2.69187
H	1.97586	-1.56443	3.03105
H	4.09293	-0.73057	4.14708
H	3.58600	0.93873	3.84613
H	3.01521	0.25113	6.20680
H	1.51946	0.57681	5.28911
H	2.03246	-1.10519	5.59228
H	-0.72078	-1.82480	2.54861
H	-1.01349	-0.83265	4.00790
H	-4.40052	-3.18844	3.40095

H	-3.22066	-3.61128	2.13842
H	-1.65583	-3.54459	4.03191
H	-2.66525	-2.34962	4.88819
H	-2.51033	-1.00946	-0.16913
H	-4.21445	-2.22387	-1.41576
H	0.58535	-2.62408	-2.44873
H	-0.98981	-3.91355	-3.84285
H	-5.71473	-4.38143	-3.94856
H	-5.43023	-3.89085	-2.25825
H	-5.42625	-2.65508	-3.55747
H	-4.14237	-5.21017	-5.12105
H	-2.86751	-4.00133	-5.47859
H	-2.50809	-5.42196	-4.44282
H	-1.11498	0.39818	1.39813
C	-4.29355	1.83583	2.20821
C	-5.34960	2.00994	1.09079
H	-4.61311	1.02580	2.88673
H	-4.25578	2.77109	2.80830
C	-6.74547	2.36844	1.63375
H	-5.01532	2.79982	0.38630
H	-5.39813	1.06442	0.52099
C	-7.79282	2.55010	0.52214
H	-7.07772	1.57137	2.32821
H	-6.68050	3.29672	2.23755
H	-8.78662	2.79953	0.93661
H	-7.50437	3.36356	-0.16983
H	-7.90013	1.62667	-0.07654

Table S10. Cartesian coordinates (Å) of the optimized structures involved in catalytic system **1_c**atONIOM{BP86/LANL2DZ(Ru)/6-31G*(H, C, N, O & P):HF/STO-3G} level.

1_c

147

XYZ

Ru	-0.00002	-0.00003	0.53246
P	-1.24323	1.98436	0.02572
P	-1.48472	-1.82867	0.39644
C	0.17136	3.14746	0.39190
H	0.29435	3.99352	-0.30204
H	0.08371	3.54818	1.41429
P	1.48471	1.82858	0.39655
P	1.24324	-1.98435	0.02571
C	-0.17133	-3.14751	0.39182
H	-0.29427	-3.99361	-0.30209
H	-0.08372	-3.54817	1.41423
C	-2.61283	-2.26434	-1.15579
C	-2.77009	-3.76393	-1.51785
C	-4.00649	-1.59981	-1.07907
H	-2.07461	-1.79429	-1.97713
C	-3.55931	-3.94111	-2.83667
H	-3.28789	-4.29653	-0.72558
H	-1.79841	-4.23217	-1.64210
C	-4.79502	-1.77161	-2.39704
H	-4.58565	-2.03608	-0.26992
H	-3.89765	-0.54126	-0.86913
C	-4.94125	-3.26095	-2.76793
H	-3.67898	-5.00238	-3.04598
H	-2.98790	-3.51055	-3.65681
H	-5.77829	-1.31677	-2.29425
H	-4.27642	-1.24977	-3.19871
H	-5.44951	-3.35768	-3.72513
H	-5.55411	-3.76141	-2.02069
C	-2.48649	-2.29901	1.99482
C	-3.40812	-1.16524	2.48711
C	-3.24574	-3.64821	1.99202
H	-1.68211	-2.38033	2.72343
C	-3.96213	-1.47835	3.89531
H	-4.24238	-1.02607	1.80449
H	-2.83593	-0.24105	2.51737
C	-3.79379	-3.96948	3.40208
H	-4.08065	-3.61286	1.29736
H	-2.58848	-4.45464	1.67314
C	-4.70076	-2.83366	3.92082
H	-4.63889	-0.68590	4.20898
H	-3.13865	-1.50358	4.60614
H	-4.35122	-4.90395	3.37443
H	-2.96074	-4.10731	4.08865
H	-5.02912	-3.05782	4.93375
H	-5.59045	-2.77152	3.29685
C	1.63783	-2.59874	-1.80648
C	2.46178	-1.57107	-2.61064
C	2.23921	-4.01393	-1.97578
H	0.64097	-2.61560	-2.24512
C	2.52275	-1.95586	-4.10632
H	3.47542	-1.51316	-2.22208
H	2.01028	-0.58697	-2.50535
C	2.29243	-4.40975	-3.47059
H	3.24833	-4.04666	-1.57434
H	1.64853	-4.75006	-1.43527
C	3.09138	-3.37841	-4.29510
H	3.13884	-1.23905	-4.64569
H	1.52171	-1.90902	-4.53070
H	2.74598	-5.39393	-3.57173
H	1.27885	-4.47682	-3.86131
H	3.06713	-3.65011	-5.34847
H	4.13260	-3.39482	-3.97863
C	2.71034	-2.52456	1.17162
C	2.75936	-4.03235	1.53167
C	4.09435	-2.06898	0.65319
C	3.86417	-4.31609	2.57521
H	2.95340	-4.62930	0.64525
H	1.80710	-4.35739	1.94033
C	5.20394	-2.35098	1.69128
H	4.33980	-2.58831	-0.26915
H	4.07696	-1.00668	0.43584
C	5.24490	-3.84355	2.07677
H	3.89335	-5.38246	2.79034
H	3.62112	-3.80276	3.50349
H	6.16637	-2.04672	1.28459
H	5.02245	-1.75356	2.58223
H	5.99310	-4.00601	2.84981
H	5.53763	-4.43328	1.21011
H	2.50332	-1.98031	2.08980
C	2.61285	2.26428	-1.15569
C	2.77006	3.76387	-1.51776
C	4.00654	1.59982	-1.07897
H	2.07466	1.79419	-1.97703
C	3.55925	3.94108	-2.83659
H	3.28785	4.29649	-0.72550
H	1.79836	4.23209	-1.64201
C	4.79506	1.77165	-2.39695
H	4.58568	2.03611	-0.26983
H	3.89776	0.54126	-0.86904
C	4.94123	3.26098	-2.76785
H	3.67887	5.00235	-3.04591
H	2.98785	3.51048	-3.65672
H	5.77835	1.31684	-2.29416
H	4.27648	1.24977	-3.19861
H	5.44947	3.35772	-3.72506

H	5.55407	3.76148	-2.02062
C	2.48652	2.29890	1.99490
C	3.40819	1.16513	2.48712
C	3.24577	3.64810	1.99207
H	1.68218	2.38021	2.72355
C	3.96227	1.47822	3.89530
H	4.24242	1.02599	1.80446
H	2.83601	0.24094	2.51739
C	3.79389	3.96936	3.40211
H	4.08065	3.61276	1.29737
H	2.58848	4.45452	1.67323
C	4.70089	2.83354	3.92080
H	4.63905	0.68577	4.20892
H	3.13883	1.50344	4.60618
H	4.35131	4.90383	3.37444
H	2.96087	4.10717	4.08872
H	5.02929	3.05770	4.93372
H	5.59055	2.77142	3.29679
C	-2.71034	2.52449	1.17170
C	-2.75936	4.03226	1.53185
C	-4.09435	2.06895	0.65325
H	-2.50330	1.98016	2.08983
C	-3.86415	4.31591	2.57544
H	-2.95341	4.62927	0.64548
H	-1.80710	4.35726	1.94053
C	-5.20393	2.35087	1.69139
H	-4.33982	2.58838	-0.26903
H	-4.07697	1.00668	0.43580
C	-5.24489	3.84340	2.07699
H	-3.89333	5.38227	2.79065
H	-3.62108	3.80251	3.50368
H	-6.16636	2.04663	1.28468
H	-5.02242	1.75337	2.58229
H	-5.99308	4.00580	2.85007
H	-5.53764	4.43320	1.21039
C	-1.63788	2.59886	-1.80638
C	-2.46196	1.57133	-2.61057
C	-2.23914	4.01411	-1.97553
H	-0.64104	2.61566	-2.24510
C	-2.52297	1.95626	-4.10622
H	-3.47558	1.51348	-2.22197
H	-2.01055	0.58718	-2.50539
C	-2.29240	4.41007	-3.47030
H	-3.24824	4.04690	-1.57402
H	-1.64837	4.75014	-1.43497
C	-3.09148	3.37887	-4.29486
H	-3.13915	1.23955	-4.64562
H	-1.52195	1.90936	-4.53066
H	-2.74588	5.39430	-3.57132
H	-1.27884	4.47709	-3.86107
H	-3.06726	3.65066	-5.34821
H	-4.13268	3.39534	-3.97833

2_c

160

XYZ

Ru	0.02360	0.05065	0.09523
P	1.09349	-2.00115	0.42126
N	-0.04945	0.14555	-3.40138
C	0.07760	-1.01550	-4.11445
C	-0.07162	-0.66315	-5.61041
C	-0.01692	0.87783	-5.64789
C	-0.44095	1.29690	-4.21307
O	0.24699	-2.15467	-3.65027
P	1.67718	1.68175	-0.09195
H	-1.53567	1.47700	-4.15533
H	0.06365	2.22155	-3.87884
H	0.71367	-1.16258	-6.20155
H	-1.04397	-1.05990	-5.96053
H	-0.66563	1.32775	-6.41826
H	1.01725	1.21545	-5.83994
H	-0.06812	0.13564	-2.34418
C	-0.28663	-3.06817	-0.28240
H	-0.11762	-3.17134	-1.36661
H	-0.47734	-4.05907	0.15696
P	-1.58895	-1.69774	-0.12439
C	-2.72457	-2.16894	1.38949
C	-4.07305	-1.41156	1.35902
C	-2.97123	-3.67743	1.64194
H	-2.16247	-1.78466	2.23649
C	-4.86282	-1.62243	2.67034
H	-4.68054	-1.75980	0.52782
H	-3.89665	-0.34905	1.21540
C	-3.76338	-3.89469	2.95251
H	-3.52704	-4.11684	0.81880
H	-2.02698	-4.20887	1.71688
C	-5.09858	-3.12234	2.94248
H	-5.81712	-1.10310	2.60834
H	-4.30756	-1.18735	3.49891
H	-3.95225	-4.95764	3.08955
H	-3.16138	-3.56001	3.79505
H	-5.60642	-3.25063	3.89615
H	-5.74684	-3.53170	2.17002
C	-2.56001	-1.98780	-1.77106
C	-3.35401	-3.31256	-1.86893
C	-3.45954	-0.79704	-2.16870
H	-1.74844	-2.03986	-2.49219
C	-3.91046	-3.51035	-3.29755
H	-4.18740	-3.30779	-1.17116
H	-2.71569	-4.15588	-1.61425
C	-4.03226	-0.98653	-3.59171
H	-4.28618	-0.69440	-1.47023
H	-2.87846	0.12074	-2.13138
C	-4.79842	-2.32035	-3.71797

H	-4.48543	-4.43337	-3.34153
H	-3.08120	-3.60787	-3.99541
H	-4.69443	-0.15732	-3.83349
H	-3.21645	-0.97258	-4.31098
H	-5.13717	-2.45598	-4.74308
H	-5.68292	-2.28976	-3.08435
C	0.96141	-2.41971	2.34427
C	1.06916	-3.89462	2.81150
C	1.89465	-1.52935	3.18945
H	-0.05193	-2.08476	2.54051
C	0.70664	-4.01410	4.31047
H	2.07819	-4.26895	2.67567
H	0.40630	-4.53339	2.23292
C	1.56223	-1.64843	4.69392
H	2.93473	-1.80678	3.03306
H	1.76595	-0.49777	2.87047
C	1.61670	-3.11477	5.17343
H	0.80273	-5.05039	4.62920
H	-0.33175	-3.72340	4.45690
H	2.26032	-1.04690	5.27303
H	0.56524	-1.24973	4.87062
H	1.31344	-3.17462	6.21678
H	2.64111	-3.47719	5.11119
C	2.77088	-2.64130	-0.31484
C	3.58021	-3.64791	0.53773
C	2.56281	-3.23134	-1.73066
H	3.37369	-1.74200	-0.41076
C	4.94464	-3.95095	-0.12497
H	3.02569	-4.57739	0.64206
H	3.75807	-3.25340	1.53377
C	3.91885	-3.52228	-2.41031
H	2.00920	-4.16420	-1.65382
H	1.97334	-2.55986	-2.35188
C	4.77147	-4.48759	-1.56078
H	5.48610	-4.67665	0.47883
H	5.53976	-3.04005	-0.14932
H	3.74490	-3.95198	-3.39460
H	4.46162	-2.59011	-2.55271
H	5.74705	-4.62810	-2.02170
H	4.28499	-5.46041	-1.52444
P	-1.00549	2.14152	0.25472
C	0.50006	3.15873	-0.26284
H	0.43824	3.42556	-1.33099
H	0.70858	4.07594	0.31051
C	2.70122	1.90422	-1.72849
C	3.60537	3.15807	-1.80706
C	3.49541	0.64531	-2.13429
H	1.90865	2.02321	-2.46204
C	4.19360	3.32387	-3.22729
H	4.42773	3.07265	-1.10168
H	3.04146	4.05138	-1.54632
C	4.09666	0.80234	-3.54935
H	4.30015	0.45764	-1.42755
H	2.82818	-0.21230	-2.12034
C	4.97783	2.06523	-3.65370
H	4.84721	4.19376	-3.25323
H	3.38526	3.50249	-3.93376
H	4.68593	-0.07837	-3.79661
H	3.28833	0.86658	-4.27460
H	5.33742	2.18245	-4.67397
H	5.84988	1.95158	-3.01241
C	2.81849	2.07287	1.44360
C	4.08867	1.18949	1.44628
C	3.20307	3.55254	1.69444
H	2.19626	1.75382	2.27601
C	4.86796	1.33122	2.77305
H	4.74319	1.47589	0.62691
H	3.81641	0.14728	1.29856
C	3.98192	3.69965	3.02286
H	3.81772	3.93235	0.88357
H	2.31280	4.17329	1.74623
C	5.23772	2.80395	3.04555
H	5.77068	0.72481	2.73271
H	4.25775	0.95447	3.59137
H	4.26799	4.74041	3.16209
H	3.33122	3.42735	3.85164
H	5.73404	2.88897	4.01007
H	5.93885	3.14636	2.28679
C	-2.45676	2.93970	-0.76039
C	-3.85250	2.56745	-0.20723
C	-2.36355	4.47251	-0.96441
H	-2.35388	2.47547	-1.73932
C	-4.97530	3.05843	-1.14873
H	-3.99508	3.01659	0.77248
H	-3.93088	1.49065	-0.08755
C	-3.47965	4.96589	-1.91402
H	-2.45979	4.98797	-0.01348
H	-1.39953	4.74387	-1.38546
C	-4.87788	4.57920	-1.38908
H	-5.94423	2.81342	-0.71831
H	-4.89984	2.53655	-2.10063
H	-3.41163	6.04659	-2.02210
H	-3.33122	4.52996	-2.90011
H	-5.63885	4.89062	-2.10164
H	-5.07034	5.10492	-0.45577
C	-1.14905	2.66737	2.14136
C	-1.52194	4.13553	2.46539
C	-2.05840	1.71001	2.93800
C	-1.39439	4.41079	3.98232
H	-2.54639	4.33773	2.16307
H	-0.87916	4.82668	1.92460
C	-1.94128	1.97081	4.45605
H	-3.09593	1.83600	2.63700
H	-1.76096	0.68737	2.71396

C	-2.26948	3.43918	4.80192
H	-1.68541	5.43808	4.19373
H	-0.35393	4.29925	4.28145
H	-2.61452	1.30771	4.99591
H	-0.92853	1.74182	4.78175
H	-2.11702	3.61327	5.86518
H	-3.31862	3.63408	4.58758
H	-0.12953	2.49775	2.47977

$[2_c\text{-}3_c]^{\ddagger}$

160

XYZ

Ru	0.07525	0.01052	-0.32708
P	1.23291	1.30611	1.19028
N	-0.16452	-0.19706	-2.72700
C	-0.24946	0.66705	-3.80664
C	-0.29714	-0.16697	-5.10580
C	-0.59885	-1.59754	-4.63269
C	-0.09332	-1.60608	-3.16099
O	-0.24249	1.90401	-3.74545
P	-2.10688	0.95233	-0.20555
H	0.93389	-2.00857	-3.08934
H	-0.72162	-2.23169	-2.50718
H	-1.03584	0.25496	-5.80667
H	0.69536	-0.08711	-5.59082
H	-0.11751	-2.38157	-5.24297
H	-1.68827	-1.78309	-4.65163
H	0.46755	0.58040	-1.88681
C	2.90572	0.46356	1.03007
H	3.78418	1.11949	0.94632
H	3.07283	-0.21908	1.87714
P	2.42016	-0.58112	-0.45392
C	3.17115	-2.29389	0.06264
C	2.66584	-3.45871	-0.81858
C	4.70974	-2.36921	0.21452
H	2.74832	-2.44140	1.05393
C	3.08805	-4.82396	-0.22958
H	3.06596	-3.37314	-1.82505
H	1.58247	-3.41537	-0.89609
C	5.13758	-3.73014	0.81141
H	5.19058	-2.24885	-0.75234
H	5.06689	-1.56829	0.85857
C	4.61838	-4.90445	-0.04505
H	2.75375	-5.62521	-0.88568
H	2.60163	-4.96725	0.73338
H	6.22285	-3.77481	0.87909
H	4.74461	-3.81955	1.82242
H	4.88511	-5.84934	0.42403
H	5.10008	-4.87790	-1.02061
C	3.46864	0.12743	-1.94764
C	4.83600	0.76182	-1.58046
C	3.68020	-0.89096	-3.09421
H	2.82611	0.91851	-2.32654
C	5.48149	1.43966	-2.81163
H	5.51473	0.00455	-1.19701
H	4.71966	1.51344	-0.80677
C	4.30895	-0.21394	-4.33284
H	4.33723	-1.69243	-2.76751
H	2.73512	-1.33709	-3.38195
C	5.65476	0.45057	-3.98127
H	6.44780	1.85329	-2.52949
H	4.85312	2.26842	-3.13192
H	4.45510	-0.95580	-5.11546
H	3.62326	0.53782	-4.71845
H	6.05104	0.97162	-4.85029
H	6.37547	-0.31626	-3.70337
C	0.94388	1.18473	3.14100
C	2.18117	1.29437	4.07236
C	-0.16750	2.13774	3.64062
H	0.57049	0.17022	3.25056
C	1.80597	1.01671	5.54730
H	2.62492	2.28249	4.01430
H	2.94698	0.58383	3.77635
C	-0.53922	1.87794	5.11887
H	0.14966	3.17270	3.53708
H	-1.05913	2.01307	3.03467
C	0.69741	1.97100	6.03474
H	2.68960	1.12665	6.17303
H	1.46396	-0.01150	5.64706
H	-1.29145	2.59709	5.43734
H	-0.97890	0.88657	5.21058
H	0.42021	1.72728	7.05834
H	1.07298	2.99241	6.03262
C	1.54749	3.20602	0.86135
C	2.57598	3.92035	1.77149
C	1.93600	3.41739	-0.61968
H	0.58113	3.67609	1.02655
C	2.69364	5.42225	1.41520
H	3.55402	3.45360	1.67350
H	2.28216	3.84724	2.81286
C	2.01826	4.91599	-0.97786
H	2.90884	2.96882	-0.80209
H	1.21976	2.90555	-1.26080
C	3.03594	5.64037	-0.07248
H	3.45486	5.88222	2.04261
H	1.74888	5.91367	1.63995
H	2.30667	5.02548	-2.02122
H	1.03903	5.37538	-0.86110
H	3.04491	6.70546	-0.29494
H	4.03455	5.25705	-0.27329
P	-1.44732	-1.58273	0.85349
C	-2.72579	-0.21001	1.14519
H	-3.79513	-0.47098	1.17840
H	-2.46543	0.26121	2.10831

C	-3.22517	0.58949	-1.75254
C	-4.67024	0.11026	-1.45986
C	-3.27078	1.77624	-2.74467
H	-2.69523	-0.22146	-2.24483
C	-5.39295	-0.29308	-2.76637
H	-5.23852	0.89678	-0.97028
H	-4.66640	-0.74689	-0.79554
C	-3.97338	1.36669	-4.05799
H	-3.82700	2.60035	-2.30821
H	-2.26239	2.11877	-2.96818
C	-5.40536	0.86012	-3.78999
H	-6.41233	-0.59704	-2.53592
H	-4.88793	-1.15333	-3.20151
H	-4.00331	2.21969	-4.73267
H	-3.39865	0.58412	-4.54791
H	-5.86315	0.52337	-4.71786
H	-6.01237	1.67726	-3.40480
C	-2.57989	2.68832	0.50244
C	-2.05957	3.87474	-0.34261
C	-4.07882	2.89560	0.83090
H	-2.04236	2.71475	1.44567
C	-2.24497	5.20283	0.42595
H	-2.60222	3.94200	-1.27880
H	-1.01252	3.72769	-0.58941
C	-4.28706	4.22410	1.59455
H	-4.66261	2.92329	-0.08536
H	-4.45601	2.07335	1.43521
C	-3.72608	5.42380	0.80147
H	-1.89456	6.03083	-0.18686
H	-1.64132	5.18882	1.33142
H	-5.34809	4.37222	1.78603
H	-3.78780	4.16435	2.55983
H	-3.82792	6.33306	1.39040
H	-4.30993	5.55741	-0.10719
C	-2.44460	-2.93887	-0.11914
C	-1.60054	-4.21843	-0.33356
C	-3.85269	-3.33771	0.39006
H	-2.57810	-2.47299	-1.09346
C	-2.28052	-5.16543	-1.34814
H	-1.47790	-4.74269	0.61147
H	-0.60919	-3.95743	-0.69446
C	-4.55014	-4.25469	-0.64147
H	-3.78125	-3.87466	1.32927
H	-4.47107	-2.46250	0.56445
C	-3.71566	-5.52489	-0.90930
H	-1.68864	-6.07250	-1.45259
H	-2.31080	-4.68213	-2.32264
H	-5.53523	-4.53218	-0.27162
H	-4.69262	-3.71009	-1.57271
H	-4.19621	-6.12728	-1.67735
H	-3.67560	-6.12506	-0.00224
C	-1.13975	-2.19748	2.66341
C	-2.31456	-2.89636	3.38666
C	0.13615	-3.06332	2.77942
C	-1.96914	-3.12969	4.87529
H	-2.51305	-3.86108	2.92960
H	-3.22099	-2.29947	3.31272
C	0.49153	-3.33625	4.25885
H	-0.00682	-4.01158	2.26694
H	0.96642	-2.55458	2.29763
C	-0.68873	-3.97937	5.01717
H	-2.80066	-3.63038	5.36722
H	-1.82669	-2.17084	5.36988
H	1.36153	-3.98781	4.31018
H	0.75916	-2.39828	4.74096
H	-0.43356	-4.09112	6.06890
H	-0.87374	-4.97524	4.61910
H	-0.94781	-1.26839	3.19413

3_c

160

XYZ

Ru	0.09839	0.04336	-0.45873
P	1.62342	0.95298	1.13888
N	-0.53344	-0.57735	-2.41856
C	-0.31713	0.23091	-3.49156
C	-0.66641	-0.52552	-4.79597
C	-1.46813	-1.74047	-4.30494
C	-0.93337	-1.92124	-2.86060
O	0.09944	1.41599	-3.48112
P	-1.75737	1.49948	-0.28188
H	-0.07230	-2.62360	-2.85703
H	-1.68552	-2.35647	-2.18603
H	-1.20330	0.13620	-5.49566
H	0.27786	-0.82369	-5.29302
H	-1.34385	-2.64957	-4.92050
H	-2.54726	-1.49871	-4.27735
H	0.73989	1.10721	-1.46928
C	3.00954	-0.27525	0.79407
H	4.00168	0.16086	0.60677
H	3.11129	-0.98473	1.62924
P	2.15572	-1.16696	-0.63302
C	2.43514	-2.99690	-0.05901
C	1.52826	-4.01005	-0.79357
C	3.89899	-3.50043	-0.05508
H	2.10377	-2.97048	0.97730
C	1.62441	-5.41123	-0.14759
H	1.81442	-4.08338	-1.83926
H	0.49762	-3.66754	-0.76297
C	4.00006	-4.89591	0.60269
H	4.27217	-3.57018	-1.07319
H	4.53974	-2.80402	0.48169
C	3.08290	-5.91386	-0.10707
H	1.00833	-6.11305	-0.70611

H	1.23105	-5.36831	0.86633
H	5.03147	-5.24134	0.56851
H	3.71595	-4.82248	1.65079
H	3.12970	-6.87281	0.40497
H	3.43849	-6.06956	-1.12376
C	3.20556	-0.80615	-2.22004
C	4.73612	-0.65649	-2.02474
C	2.92247	-1.82096	-3.35227
H	2.80962	0.15554	-2.53844
C	5.41044	-0.20591	-3.34121
H	5.17516	-1.59904	-1.70974
H	4.95893	0.07738	-1.25626
C	3.59103	-1.37444	-4.67154
H	3.30398	-2.80255	-3.08182
H	1.85239	-1.91018	-3.50736
C	5.11150	-1.18653	-4.49341
H	6.48539	-0.12915	-3.18895
H	5.04645	0.78439	-3.60763
H	3.39827	-2.11543	-5.44476
H	3.14579	-0.43680	-4.99781
H	5.55036	-0.81451	-5.41688
H	5.57296	-2.14844	-4.27742
C	1.43504	0.84016	3.08437
C	2.72127	0.63028	3.92682
C	0.60395	2.01987	3.64661
H	0.84146	-0.06052	3.21294
C	2.38434	0.44168	5.42452
H	3.39454	1.47432	3.83148
H	3.25890	-0.24819	3.58204
C	0.28341	1.83587	5.14732
H	1.14090	2.95573	3.51703
H	-0.32858	2.10857	3.09625
C	1.56835	1.62913	5.97409
H	3.30796	0.33472	5.98984
H	1.81563	-0.47637	5.55612
H	-0.25667	2.70735	5.51198
H	-0.36778	0.97337	5.27468
H	1.31273	1.45176	7.01666
H	2.17314	2.53303	5.93520
C	2.41936	2.69846	0.78524
C	3.53685	3.15336	1.75820
C	2.95094	2.77060	-0.66738
H	1.59409	3.39957	0.87857
C	4.05176	4.56893	1.40418
H	4.36902	2.45303	1.72487
H	3.17211	3.18114	2.77851
C	3.43935	4.19075	-1.02330
H	3.78579	2.08427	-0.77925
H	2.17244	2.45668	-1.35723
C	4.54332	4.65765	-0.05370
H	4.85646	4.83674	2.08629
H	3.24712	5.28604	1.55515
H	3.81563	4.19569	-2.04406
H	2.60352	4.88575	-0.98176
H	4.83646	5.68038	-0.28154
H	5.42387	4.03017	-0.17742
P	-1.76161	-1.07686	0.96427
C	-2.56416	0.62460	1.18249
H	-3.65790	0.67709	1.28917
H	-2.12062	1.06391	2.09093
C	-3.05497	1.42647	-1.71821
C	-4.54802	1.53994	-1.31815
C	-2.73722	2.44456	-2.83903
H	-2.88971	0.43289	-2.12717
C	-5.45865	1.29626	-2.54429
H	-4.75968	2.52618	-0.91441
H	-4.80303	0.81558	-0.55154
C	-3.63637	2.19480	-4.06978
H	-2.92367	3.45355	-2.48314
H	-1.68772	2.35691	-3.12381
C	-5.13165	2.26914	-3.69581
H	-6.50045	1.40646	-2.24883
H	-5.32513	0.27290	-2.88969
H	-3.41214	2.93208	-4.83781
H	-3.41673	1.21435	-4.48604
H	-5.74633	2.03216	-4.56185
H	-5.37832	3.28399	-3.38941
C	-1.59669	3.30879	0.36048
C	-0.80995	4.23536	-0.59663
C	-2.91892	3.99533	0.78347
H	-0.99511	3.19119	1.25897
C	-0.50891	5.58904	0.08672
H	-1.38779	4.42591	-1.49421
H	0.11320	3.75488	-0.90744
C	-2.63356	5.35047	1.47069
H	-3.54066	4.17251	-0.09001
H	-3.48182	3.35934	1.46347
C	-1.80811	6.27906	0.55491
H	0.02052	6.23762	-0.60816
H	0.14181	5.43074	0.94446
H	-3.57381	5.83140	1.73340
H	-2.08764	5.17658	2.39612
H	-1.56970	7.19984	1.08344
H	-2.40514	6.54776	-0.31437
C	-3.17663	-2.13922	0.15670
C	-2.80253	-3.64121	0.11050
C	-4.61981	-1.98933	0.70239
H	-3.18919	-1.77209	-0.86640
C	-3.78820	-4.43011	-0.78058
H	-2.82704	-4.05840	1.11440
H	-1.79299	-3.76714	-0.27174
C	-5.61158	-2.75189	-0.20685
H	-4.69734	-2.39067	1.70659
H	-4.91557	-0.94630	0.74681

C	-5.24444	-4.24726	-0.30481
H	-3.52488	-5.48579	-0.76674
H	-3.69678	-4.08446	-1.80822
H	-6.61953	-2.64460	0.18903
H	-5.60268	-2.31006	-1.20124
H	-5.92283	-4.74952	-0.99120
H	-5.36625	-4.71164	0.67200
C	-1.55225	-1.64628	2.80296
C	-2.84229	-1.95241	3.59939
C	-0.56179	-2.82912	2.92844
C	-2.51310	-2.22137	5.08559
H	-3.33028	-2.83417	3.19546
H	-3.54116	-1.12197	3.52737
C	-0.23963	-3.13835	4.40829
H	-0.98161	-3.71791	2.46428
H	0.35949	-2.59431	2.40157
C	-1.52188	-3.39529	5.22714
H	-3.43098	-2.44594	5.62511
H	-2.08362	-1.32622	5.53109
H	0.41424	-4.00631	4.46387
H	0.29865	-2.29800	4.84114
H	-1.26665	-3.54013	6.27478
H	-1.99668	-4.31002	4.87758
H	-1.08937	-0.78291	3.27360

$[3_c\text{-}4_c]^‡$

176

XYZ

Ru	-0.86007	0.45505	-0.21389
C	1.09775	3.11063	-1.65124
C	1.24421	2.03227	-2.21542
P	0.93943	0.05192	1.38903
N	-1.88580	1.65322	-1.60664
C	-2.25103	2.94415	-1.34146
C	-2.62574	3.65913	-2.66097
C	-2.80580	2.51066	-3.66263
C	-1.88947	1.41603	-3.06153
O	-2.28113	3.51968	-0.22815
P	-2.75526	-0.17841	0.79758
H	-0.86040	1.51303	-3.46904
H	-2.23856	0.40306	-3.30144
H	-3.51056	4.30117	-2.51563
H	-1.78278	4.32073	-2.94472
H	-2.53482	2.76537	-4.70290
H	-3.85670	2.16482	-3.66262
H	-0.94712	1.85699	0.53854
C	0.90181	4.42668	-1.03547
C	1.13262	5.59522	-2.03032
H	-0.12751	4.46980	-0.62457
H	1.59027	4.54434	-0.17416
C	0.91988	6.96898	-1.36909
H	2.15727	5.52941	-2.44567
H	0.44040	5.48092	-2.88694
C	1.13444	8.13963	-2.34280
H	-0.10398	7.01466	-0.94895
H	1.61103	7.07095	-0.50814
H	0.97615	9.11203	-1.84294
H	2.16111	8.13648	-2.75414
H	0.43324	8.08337	-3.19598
C	2.63230	0.60569	0.69588
H	2.41167	1.62841	0.34787
H	3.35566	0.68855	1.52167
P	3.39020	-0.13306	-0.88619
C	4.53837	-1.57951	-0.34419
C	4.94813	-2.41056	-1.58759
C	5.80115	-1.26135	0.49390
H	3.89616	-2.21988	0.25509
C	5.68076	-3.70900	-1.17948
H	5.60290	-1.82696	-2.22901
H	4.06684	-2.66118	-2.17312
C	6.52641	-2.55696	0.92420
H	6.49161	-0.66508	-0.09589
H	5.54444	-0.68121	1.37734
C	6.91458	-3.40762	-0.30322
H	5.98638	-4.25213	-2.07159
H	4.99721	-4.35020	-0.62675
H	7.41830	-2.30376	1.49405
H	5.87737	-3.13774	1.57623
H	7.37353	-4.33910	0.02184
H	7.65330	-2.86931	-0.89392
C	4.48723	1.36920	-1.40746
C	5.33475	2.09568	-0.33127
C	5.37459	1.02938	-2.63017
H	3.74396	2.08515	-1.74873
C	5.97536	3.37592	-0.91626
H	6.12527	1.45033	0.03878
H	4.71655	2.37204	0.51841
C	6.01927	2.30307	-3.22185
H	6.16640	0.34307	-2.34005
H	4.78053	0.53672	-3.39736
C	6.83864	3.05943	-2.15541
H	6.58627	3.85785	-0.15535
H	5.18973	4.07578	-1.19422
H	6.66125	2.03393	-4.05837
H	5.23807	2.95555	-3.60683
H	7.23197	3.98220	-2.57697
H	7.68914	2.44998	-1.85565
C	1.39622	-1.70636	2.11895
C	2.75470	-1.87340	2.84811
C	0.27307	-2.27581	3.01878
H	1.44546	-2.32672	1.22663
C	3.02347	-3.36201	3.16758
H	2.76119	-1.32699	3.78234
H	3.56762	-1.48654	2.24700

C	0.52689	-3.75439	3.39288
H	0.19510	-1.69040	3.93140
H	-0.68172	-2.20384	2.51111
C	1.90586	-3.94560	4.05550
H	3.98212	-3.45518	3.67382
H	3.08664	-3.93142	2.24243
H	-0.25781	-4.09731	4.06432
H	0.47486	-4.36595	2.49472
H	2.08579	-5.00360	4.23443
H	1.91585	-3.44465	5.02150
C	0.80578	1.28458	2.90755
C	1.68935	0.99679	4.14709
C	1.02852	2.74608	2.44627
H	-0.22973	1.19529	3.21915
C	1.41177	2.01063	5.28336
H	2.74297	1.04326	3.88122
H	1.48878	0.00564	4.53805
C	0.71668	3.74729	3.57879
H	2.06312	2.88472	2.14453
H	0.38924	2.94908	1.59242
C	1.58980	3.46879	4.81826
H	2.07909	1.80085	6.11720
H	0.39359	1.87194	5.64092
H	0.89167	4.76008	3.22196
H	-0.33437	3.67535	3.84980
H	1.32431	4.14719	5.62641
H	2.63525	3.64591	4.57299
P	-1.50596	-1.90521	-1.08289
C	-2.70990	-2.01127	0.38239
H	-3.67314	-2.51375	0.21022
H	-2.20463	-2.54522	1.20118
C	-4.37624	0.47904	-0.05672
C	-5.59342	-0.48085	-0.07476
C	-4.81942	1.85748	0.48713
H	-4.04789	0.62735	-1.08161
C	-6.73021	0.10287	-0.94687
H	-5.96747	-0.63946	0.93326
H	-5.32308	-1.45209	-0.47635
C	-5.93591	2.45348	-0.39830
H	-5.21275	1.74650	1.49283
H	-3.96894	2.53817	0.51351
C	-7.15292	1.50763	-0.47010
H	-7.58530	-0.57023	-0.92363
H	-6.39105	0.16142	-1.97935
H	-6.24202	3.41685	0.00409
H	-5.55097	2.62690	-1.40072
H	-7.90386	1.91307	-1.14516
H	-7.60876	1.43030	0.51521
C	-2.98276	-0.14860	2.71693
C	-3.03015	1.27412	3.32211
C	-4.16338	-0.98271	3.27351
H	-2.06733	-0.61428	3.07100
C	-2.92546	1.20188	4.86259
H	-3.96614	1.75884	3.07006
H	-2.23191	1.88730	2.91297
C	-4.09163	-1.06394	4.81615
H	-5.10751	-0.52436	2.99064
H	-4.15258	-1.98977	2.86185
C	-4.06350	0.34184	5.45363
H	-2.97034	2.20587	5.27958
H	-1.96636	0.77338	5.14616
H	-4.94619	-1.62262	5.19306
H	-3.19468	-1.60891	5.10457
H	-3.94178	0.25387	6.53137
H	-5.01617	0.83596	5.27310
C	-2.67288	-2.17831	-2.61795
C	-1.85749	-2.33379	-3.92555
C	-3.75309	-3.28781	-2.55106
H	-3.20717	-1.23361	-2.67334
C	-2.77801	-2.24403	-5.16343
H	-1.35445	-3.29783	-3.93225
H	-1.09166	-1.56408	-3.98809
C	-4.69444	-3.17803	-3.77354
H	-3.29994	-4.27255	-2.55529
H	-4.34428	-3.20558	-1.64448
C	-3.90949	-3.29109	-5.09722
H	-2.18877	-2.39355	-6.06584
H	-3.20963	-1.24667	-5.21709
H	-5.44517	-3.96408	-3.72175
H	-5.21766	-2.22442	-3.74187
H	-4.58552	-3.15744	-5.93907
H	-3.48271	-4.28913	-5.17722
C	-0.45231	-3.50717	-0.82696
C	-1.19462	-4.85612	-0.97322
C	0.82688	-3.51023	-1.69909
C	-0.27854	-6.03061	-0.56221
H	-1.49053	-5.00616	-2.00671
H	-2.09536	-4.86539	-0.36351
C	1.74734	-4.69626	-1.32896
H	0.56109	-3.58521	-2.75030
H	1.36908	-2.57560	-1.56844
C	1.00578	-6.04658	-1.41622
H	-0.81584	-6.96889	-0.68433
H	-0.01666	-5.93933	0.48985
H	2.60754	-4.70893	-1.99414
H	2.12073	-4.55803	-0.31642
H	1.66220	-6.84797	-1.08374
H	0.74421	-6.24849	-2.45311
H	-0.13809	-3.42575	0.21019
H	1.39603	1.09010	-2.71273

4_c

176

XYZ

Ru	-0.80807	-0.52740	-0.29139
C	0.54387	-2.13736	-0.92941
C	0.71068	-1.90943	0.31550
P	0.94104	1.05331	-1.01540
N	-2.09451	-2.28974	-0.40679
C	-2.56944	-2.66732	-1.62582
C	-3.04606	-4.13740	-1.57686
C	-3.08441	-4.46001	-0.07619
C	-2.07034	-3.44236	0.50900
O	-2.61351	-1.97318	-2.67366
P	-2.84033	0.79436	-0.29752
H	-1.04861	-3.87555	0.56145
H	-2.33904	-3.14819	1.53443
H	-4.00537	-4.25858	-2.10746
H	-2.29918	-4.75375	-2.11643
H	-2.82668	-5.50519	0.17201
H	-4.09336	-4.25899	0.33011
H	-1.10684	-0.65817	-1.85818
C	0.70274	-2.91454	-2.18591
C	1.03039	-4.40932	-1.94484
H	-0.22101	-2.82416	-2.78765
H	1.51055	-2.47164	-2.80854
C	1.22822	-5.18631	-3.26002
H	1.94033	-4.49613	-1.32048
H	0.20966	-4.86845	-1.36084
C	1.54009	-6.67533	-3.03513
H	0.31727	-5.08621	-3.88288
H	2.04884	-4.71908	-3.84127
H	1.67798	-7.20798	-3.99323
H	2.46475	-6.80549	-2.44231
H	0.72072	-7.17698	-2.48756
C	2.67847	0.26779	-1.16309
H	2.51430	-0.65798	-1.73201
H	3.32251	0.91045	-1.77905
P	3.52790	-0.28641	0.45508
C	5.08020	0.84953	0.65764
C	6.00590	0.30221	1.77289
C	5.93447	1.22519	-0.58087
H	4.65815	1.77415	1.04478
C	7.08676	1.33715	2.15880
H	6.49980	-0.60572	1.43679
H	5.41939	0.04764	2.65334
C	7.01307	2.26865	-0.20735
H	6.42707	0.34561	-0.98292
H	5.31277	1.63385	-1.37287
C	7.92100	1.75632	0.93028
H	7.73943	0.91751	2.92180
H	6.60805	2.21571	2.58705
H	7.61579	2.49938	-1.08363
H	6.52869	3.19148	0.10617
H	8.63239	2.52991	1.21216
H	8.49328	0.90122	0.57568
C	4.11913	-2.04656	-0.05541
C	5.26042	-2.16815	-1.09413
C	4.45193	-2.89785	1.19593
H	3.22882	-2.48102	-0.50107
C	5.49215	-3.64497	-1.48790
H	6.18611	-1.77510	-0.68259
H	5.02591	-1.59093	-1.98618
C	4.70144	-4.37569	0.81836
H	5.33644	-2.50954	1.69424
H	3.63051	-2.84328	1.90692
C	5.80950	-4.50690	-0.24781
H	6.31229	-3.70858	-2.20051
H	4.60212	-4.03193	-1.97982
H	4.97858	-4.93811	1.70786
H	3.77984	-4.80813	0.43465
H	5.91844	-5.54921	-0.54053
H	6.75910	-4.18754	0.17769
C	1.39568	2.69169	-0.04544
C	2.70850	3.42679	-0.41899
C	0.23940	3.71964	-0.03846
H	1.52406	2.33290	0.97280
C	3.00545	4.56062	0.58889
H	2.63923	3.86667	-1.40563
H	3.54380	2.74071	-0.43303
C	0.51403	4.89198	0.93169
H	0.09040	4.11870	-1.03841
H	-0.68490	3.23665	0.25269
C	1.85472	5.58649	0.61986
H	3.93349	5.05603	0.31090
H	3.14539	4.14077	1.58302
H	-0.29990	5.61154	0.86838
H	0.53556	4.51619	1.95237
H	2.05622	6.35154	1.36671
H	1.78905	6.08250	-0.34652
C	0.63061	1.56972	-2.87803
C	1.44068	2.76963	-3.43029
C	0.81256	0.35192	-3.81729
H	-0.41860	1.84725	-2.89970
C	1.04256	3.09777	-4.88921
H	2.50627	2.55477	-3.39507
H	1.26178	3.65769	-2.83488
C	0.39357	0.68298	-5.26564
H	1.85476	0.04346	-3.82208
H	0.21356	-0.47550	-3.45348
C	1.19443	1.87813	-5.81802
H	1.65787	3.92042	-5.24862
H	0.00810	3.43473	-4.90771
H	0.55160	-0.19192	-5.89263
H	-0.66927	0.91369	-5.29208
H	0.85003	2.13189	-6.81840
H	2.24603	1.60799	-5.89274
P	-1.34976	0.21583	2.08430

C	-2.60939	1.47453	1.45142
H	-3.51390	1.62740	2.05566
H	-2.10701	2.45060	1.38395
C	-4.47764	-0.24713	-0.20730
C	-5.57266	0.27155	0.75878
C	-5.08835	-0.49969	-1.60619
H	-4.11454	-1.20198	0.16396
C	-6.73843	-0.74152	0.84672
H	-5.96050	1.22844	0.41985
H	-5.17602	0.41721	1.75797
C	-6.23990	-1.52466	-1.51617
H	-5.49032	0.42568	-2.00757
H	-4.31395	-0.87501	-2.27581
C	-7.33634	-1.04751	-0.54149
H	-7.50931	-0.34559	1.50523
H	-6.37317	-1.66503	1.29178
H	-6.66633	-1.67620	-2.50553
H	-5.84765	-2.48179	-1.18017
H	-8.11013	-1.80663	-0.44764
H	-7.80710	-0.14930	-0.93680
C	-3.15454	2.38271	-1.34747
C	-3.27945	2.11229	-2.86637
C	-4.33298	3.27008	-0.87769
H	-2.24341	2.95643	-1.20162
C	-3.25756	3.44687	-3.64537
H	-4.21178	1.60386	-3.08376
H	-2.47627	1.46350	-3.20444
C	-4.33690	4.60976	-1.65000
H	-5.27619	2.76022	-1.05481
H	-4.26291	3.47107	0.18923
C	-4.39386	4.38070	-3.17567
H	-3.36185	3.24852	-4.71003
H	-2.29932	3.94110	-3.49757
H	-5.18907	5.20865	-1.33473
H	-3.43678	5.16896	-1.40154
H	-4.32661	5.33584	-3.69245
H	-5.35275	3.93658	-3.43562
C	-2.39626	-0.93778	3.23862
C	-1.49444	-1.94663	3.99113
C	-3.38103	-0.27369	4.23434
H	-2.99743	-1.49734	2.52706
C	-2.34464	-3.02579	4.69869
H	-0.90090	-1.42359	4.73711
H	-0.80683	-2.42411	3.29699
C	-4.25210	-1.35618	4.91313
H	-2.84550	0.26844	5.00539
H	-4.02973	0.43546	3.72955
C	-3.37951	-2.39227	5.65185
H	-1.69036	-3.69369	5.25513
H	-2.86061	-3.62364	3.95046
H	-4.93514	-0.88081	5.61430
H	-4.85500	-1.85961	4.15996
H	-4.01130	-3.16823	6.07878
H	-2.86185	-1.90360	6.47518
C	-0.16435	1.28777	3.17009
C	-0.75657	1.87360	4.47356
C	1.16391	0.55171	3.47295
C	0.24752	2.84336	5.13719
H	-0.96992	1.07446	5.17674
H	-1.68830	2.39718	4.27014
C	2.17363	1.49614	4.16451
H	0.97628	-0.29990	4.12137
H	1.60281	0.17167	2.55269
C	1.58238	2.13146	5.44039
H	-0.18174	3.23335	6.05789
H	0.42710	3.68973	4.47733
H	3.07492	0.93988	4.41318
H	2.46052	2.28466	3.47186
H	2.29149	2.84055	5.86233
H	1.41381	1.35636	6.18546
H	0.06655	2.12709	2.51995
H	1.15825	-2.15408	1.27115

[4_c-5_c]‡

176

XYZ

Ru	0.75492	0.60267	-0.03657
C	-0.49524	2.34096	-0.65311
C	-0.77869	1.90416	0.53571
P	-0.96713	-0.86092	-1.11805
N	2.02448	2.32197	0.38779
C	2.49396	3.03254	-0.67997
C	2.92735	4.44644	-0.22613
C	2.88604	4.36919	1.30608
C	1.87219	3.22259	1.54352
O	2.55187	2.65684	-1.87782
P	2.86837	-0.60916	-0.55943
H	0.83222	3.61438	1.58598
H	2.05355	2.70456	2.49329
H	3.90536	4.71654	-0.65879
H	2.18760	5.16919	-0.62521
H	2.58587	5.30945	1.80226
H	3.87763	4.08090	1.70251
H	0.62843	1.52919	-1.40957
C	-0.67099	3.42132	-1.68008
C	-1.15018	4.75609	-1.06664
H	0.28950	3.57312	-2.20841
H	-1.39691	3.09914	-2.45754
C	-1.34895	5.85289	-2.12975
H	-2.09714	4.59733	-0.51615
H	-0.40946	5.09349	-0.31595
C	-1.80762	7.19275	-1.53012
H	-0.40053	5.99856	-2.68392
H	-2.09083	5.50814	-2.87823

H	-1.93991	7.96053	-2.31349
H	-2.77194	7.08545	-0.99922
H	-1.06899	7.57900	-0.80345
C	-2.72141	-0.10028	-1.11964
H	-2.56806	0.90238	-1.54453
H	-3.36906	-0.64847	-1.81655
P	-3.56244	0.21859	0.56676
C	-5.06874	-0.99062	0.65460
C	-5.99426	-0.61097	1.83768
C	-5.93100	-1.25504	-0.60694
H	-4.60714	-1.93672	0.92861
C	-7.02683	-1.72619	2.11753
H	-6.52917	0.30825	1.61388
H	-5.40196	-0.43431	2.73321
C	-6.96113	-2.37750	-0.34107
H	-6.46507	-0.35571	-0.89654
H	-5.30934	-1.54373	-1.45005
C	-7.86678	-2.03635	0.86082
H	-7.68115	-1.42301	2.93249
H	-6.50655	-2.62755	2.43577
H	-7.57081	-2.53063	-1.22945
H	-6.43548	-3.30968	-0.14296
H	-8.54179	-2.86575	1.06192
H	-8.47882	-1.17068	0.61494
C	-4.22789	2.00145	0.26368
C	-5.38271	2.19466	-0.74896
C	-4.58261	2.69070	1.60566
H	-3.36029	2.51975	-0.13559
C	-5.67519	3.69632	-0.97051
H	-6.28851	1.72132	-0.37999
H	-5.13467	1.73199	-1.70202
C	-4.89875	4.18995	1.40245
H	-5.44466	2.21030	2.06092
H	-3.75244	2.59039	2.30139
C	-6.01907	4.39605	0.36122
H	-6.50149	3.80836	-1.66992
H	-4.80410	4.17270	-1.41581
H	-5.19356	4.63229	2.35203
H	-3.99989	4.70461	1.07009
H	-6.17333	5.45957	0.19073
H	-6.95153	3.99015	0.74918
C	-1.32896	-2.69477	-0.54457
C	-2.69340	-3.32300	-0.92551
C	-0.19150	-3.66532	-0.94591
H	-1.32814	-2.61668	0.53777
C	-2.87418	-4.70070	-0.24739
H	-2.78001	-3.45832	-1.99630
H	-3.50337	-2.67702	-0.61836
C	-0.36458	-5.05727	-0.29651
H	-0.16562	-3.78526	-2.02540
H	0.76706	-3.25747	-0.64873
C	-1.73864	-5.66914	-0.63415
H	-3.83394	-5.12134	-0.54043
H	-2.89050	-4.57575	0.83335
H	0.43013	-5.71684	-0.63942
H	-0.26778	-4.96670	0.78336
H	-1.86111	-6.61463	-0.11005
H	-1.78951	-5.87774	-1.70100
C	-0.67141	-1.00852	-3.05514
C	-1.62886	-1.94856	-3.83344
C	-0.67166	0.38793	-3.72707
H	0.33034	-1.42039	-3.14397
C	-1.27814	-2.01422	-5.33865
H	-2.65693	-1.61072	-3.72874
H	-1.57271	-2.95719	-3.44303
C	-0.31638	0.31592	-5.22841
H	-1.65533	0.83928	-3.62861
H	0.04072	1.03686	-3.23046
C	-1.27585	-0.61852	-5.98888
H	-1.99529	-2.66049	-5.84106
H	-0.29563	-2.46671	-5.45719
H	-0.35812	1.31764	-5.65071
H	0.70372	-0.04291	-5.34547
H	-0.97655	-0.69623	-7.03197
H	-2.28231	-0.20527	-5.96633
P	1.45257	-0.72734	1.89956
C	2.70308	-1.76206	0.94115
H	3.61846	-2.07457	1.46265
H	2.19137	-2.67952	0.61382
C	4.51065	0.37244	-0.22023
C	5.65212	-0.39806	0.49086
C	5.05956	1.05117	-1.49813
H	4.16727	1.16119	0.44492
C	6.81200	0.56243	0.84403
H	6.03447	-1.19016	-0.14638
H	5.30311	-0.86013	1.40765
C	6.21151	2.01799	-1.14719
H	5.44212	0.29957	-2.18231
H	4.25387	1.60336	-1.98267
C	7.35039	1.28790	-0.40580
H	7.61210	-0.00036	1.32125
H	6.45829	1.29877	1.56305
H	6.59530	2.46910	-2.05984
H	5.83006	2.82213	-0.52195
H	8.12224	1.99724	-0.11436
H	7.81047	0.56154	-1.07317
C	3.17318	-1.85449	-2.00507
C	3.20913	-1.17788	-3.39672
C	4.39519	-2.79144	-1.84884
H	2.28558	-2.48146	-1.97937
C	3.19544	-2.24554	-4.51428
H	4.10763	-0.57970	-3.50179
H	2.36341	-0.50561	-3.51177
C	4.39372	-3.86860	-2.95809

H	5.31522	-2.21831	-1.92209
H	4.38579	-3.27696	-0.87530
C	4.37752	-3.22665	-4.36180
H	3.24644	-1.75703	-5.48513
H	2.26003	-2.80039	-4.47710
H	5.27268	-4.50133	-2.85187
H	3.51855	-4.50489	-2.84054
H	4.31392	-4.00373	-5.12072
H	5.31120	-2.69092	-4.52153
C	2.50152	0.13015	3.28909
C	1.59493	0.79981	4.35125
C	3.58726	-0.71869	3.99959
H	3.01874	0.91640	2.74661
C	2.42317	1.68815	5.30685
H	1.09230	0.03682	4.93952
H	0.82797	1.39998	3.86787
C	4.43217	0.17491	4.93720
H	3.13662	-1.50931	4.58947
H	4.24802	-1.18941	3.27919
C	3.54876	0.88104	5.98637
H	1.76710	2.11534	6.06245
H	2.85765	2.51389	4.74776
H	5.18287	-0.43501	5.43588
H	4.95847	0.92122	4.34541
H	4.15978	1.54137	6.59810
H	3.11116	0.13580	6.64801
C	0.31178	-2.03573	2.74706
C	0.93846	-2.88824	3.87625
C	-1.01783	-1.40332	3.22699
C	-0.04596	-3.98758	4.33692
H	1.17514	-2.26415	4.73232
H	1.86279	-3.35027	3.53577
C	-2.00460	-2.48725	3.71764
H	-0.82618	-0.71056	4.04131
H	-1.47591	-0.83350	2.42090
C	-1.37777	-3.37257	4.81521
H	0.40597	-4.56359	5.14194
H	-0.23684	-4.67306	3.51358
H	-2.90498	-2.00976	4.09840
H	-2.30025	-3.11378	2.87889
H	-2.07122	-4.16381	5.09241
H	-1.19640	-2.77174	5.70426
H	0.07833	-2.72003	1.93732
H	-1.37872	2.12754	1.41459

5$_c$

176

XYZ

Ru	0.92902	0.39225	-0.46090
C	-0.76976	2.63080	-1.75210
C	-0.54761	1.83343	-0.67601
P	-0.71672	-1.25521	-0.97941
N	2.27915	2.04408	-0.99549
C	2.20554	1.72144	-2.28186
C	2.75410	2.78947	-3.21819
C	3.36211	3.80005	-2.21423
C	2.67476	3.44847	-0.85563
O	1.60259	0.62881	-2.59967
P	3.04330	-0.85015	0.24825
H	1.77723	4.07939	-0.69364
H	3.35325	3.60323	0.00261
H	3.47552	2.38070	-3.94727
H	1.91544	3.22329	-3.79680
H	3.19033	4.85103	-2.50251
H	4.45298	3.65022	-2.13386
H	-0.19839	2.46736	-2.67970
C	-1.71576	3.81646	-1.81158
C	-0.99833	5.14402	-2.15500
H	-2.50362	3.65099	-2.57962
H	-2.23741	3.93267	-0.84102
C	-1.95101	6.34895	-2.27041
H	-0.23223	5.35010	-1.37981
H	-0.44490	5.02298	-3.10951
C	-1.22683	7.66338	-2.60731
H	-2.71479	6.13709	-3.04583
H	-2.50716	6.46639	-1.31851
H	-1.93434	8.50877	-2.68582
H	-0.48292	7.91983	-1.82996
H	-0.68782	7.58673	-3.57023
C	-2.49755	-0.65704	-1.28598
H	-2.36379	0.30366	-1.80801
H	-3.01157	-1.34631	-1.97145
P	-3.53551	-0.25324	0.25497
C	-4.87311	-1.64132	0.34651
C	-5.93391	-1.29845	1.42250
C	-5.58823	-2.12024	-0.94353
H	-4.31368	-2.49149	0.72875
C	-6.83787	-2.51460	1.72613
H	-6.55916	-0.47751	1.08064
H	-5.44566	-0.97489	2.33938
C	-6.48368	-3.34686	-0.65139
H	-6.20867	-1.32868	-1.35097
H	-4.86704	-2.38837	-1.71075
C	-7.52333	-3.03440	0.44520
H	-7.59128	-2.23672	2.46061
H	-6.23679	-3.31045	2.16180
H	-6.99212	-3.65267	-1.56374
H	-5.86156	-4.18033	-0.33097
H	-8.10228	-3.92753	0.67138
H	-8.21733	-2.28083	0.07786
C	-4.36713	1.38898	-0.30180
C	-5.45061	1.32649	-1.40553
C	-4.90792	2.16664	0.92489
H	-3.53348	1.96192	-0.69847

C	-5.91042	2.74459	-1.81371
H	-6.31768	0.77765	-1.04796
H	-5.06914	0.80603	-2.28154
C	-5.38993	3.58124	0.53028
H	-5.73645	1.62781	1.37811
H	-4.12817	2.25201	1.67844
C	-6.44421	3.52586	-0.59498
H	-6.68612	2.67193	-2.57369
H	-5.07350	3.28455	-2.25112
H	-5.80776	4.08070	1.40231
H	-4.53878	4.16960	0.19467
H	-6.71804	4.53564	-0.89389
H	-7.34495	3.04091	-0.22297
C	-1.15103	-2.80192	0.12808
C	-2.08673	-3.88782	-0.46137
C	0.09908	-3.49192	0.71186
H	-1.68373	-2.33901	0.95626
C	-2.49982	-4.90336	0.62923
H	-1.58091	-4.43606	-1.24646
H	-2.97485	-3.44407	-0.89723
C	-0.27026	-4.54099	1.78550
H	0.65654	-3.97859	-0.08492
H	0.74761	-2.74729	1.15771
C	-1.25964	-5.58932	1.23764
H	-3.15938	-5.65221	0.19539
H	-3.05421	-4.39668	1.41630
H	0.63331	-5.03371	2.13940
H	-0.71958	-4.03579	2.63796
H	-1.56079	-6.26474	2.03568
H	-0.76713	-6.18651	0.47270
C	-0.15636	-1.79312	-2.77481
C	-0.38726	-3.25825	-3.21596
C	-0.73975	-0.83166	-3.83949
H	0.91582	-1.62495	-2.75478
C	0.25650	-3.51977	-4.59825
H	-1.45136	-3.47288	-3.27832
H	0.05101	-3.94743	-2.49933
C	-0.09036	-1.08275	-5.21757
H	-1.81372	-0.97499	-3.92765
H	-0.55517	0.19667	-3.54286
C	-0.27829	-2.54608	-5.66764
H	0.05968	-4.54720	-4.89885
H	1.33563	-3.40589	-4.51592
H	-0.53039	-0.41194	-5.95263
H	0.97098	-0.85176	-5.15729
H	0.23716	-2.71666	-6.61060
H	-1.33603	-2.74087	-5.83470
P	1.03154	0.42477	1.85247
C	2.53116	-0.70901	2.06626
H	3.29082	-0.35160	2.77807
H	2.21403	-1.70731	2.40559
C	4.61964	0.25676	0.04410
C	5.58958	0.34330	1.24866
C	5.38884	-0.09570	-1.25241
H	4.18261	1.23984	-0.10272
C	6.71487	1.36614	0.96716
H	6.03880	-0.62287	1.45756
H	5.05746	0.65519	2.14303
C	6.51819	0.92275	-1.52519
H	5.83111	-1.08484	-1.17358
H	4.70205	-0.10801	-2.09618
C	7.48254	1.02210	-0.32558
H	7.40138	1.38780	1.81126
H	6.28085	2.35973	0.87413
H	7.06571	0.62528	-2.41722
H	6.08356	1.90037	-1.71978
H	8.23673	1.78195	-0.51918
H	8.00047	0.07351	-0.19702
C	3.59598	-2.70604	0.16001
C	3.66162	-3.23118	-1.29497
C	4.88839	-3.07367	0.92768
H	2.78369	-3.23254	0.65080
C	3.89872	-4.75842	-1.31997
H	4.46421	-2.74065	-1.83838
H	2.73377	-3.00071	-1.81290
C	5.11030	-4.60372	0.91221
H	5.75020	-2.59544	0.47193
H	4.82633	-2.73014	1.95811
C	5.16813	-5.14441	-0.53194
H	3.98731	-5.09525	-2.35078
H	3.03854	-5.26386	-0.88521
H	6.03579	-4.84080	1.43314
H	4.29949	-5.09176	1.44965
H	5.27748	-6.22676	-0.51598
H	6.04286	-4.73562	-1.03407
C	1.72878	2.08312	2.57979
C	0.82089	3.29721	2.27132
C	2.09997	2.07190	4.08333
H	2.64914	2.21485	2.01396
C	1.51018	4.61994	2.67690
H	-0.11614	3.21141	2.81385
H	0.58436	3.31828	1.21175
C	2.81245	3.38571	4.47820
H	1.20229	1.97406	4.68741
H	2.74662	1.22980	4.31857
C	1.92957	4.61075	4.16194
H	0.83326	5.45112	2.49080
H	2.39070	4.77323	2.05713
H	3.05112	3.36411	5.53981
H	3.75176	3.46496	3.93425
H	2.46883	5.52513	4.40059
H	1.04084	4.58334	4.78954
C	-0.29624	-0.29310	3.07642
C	0.24052	-0.90267	4.39698

C	-1.41919	0.72674	3.37961
C	-0.90361	-1.56217	5.20154
H	0.70035	-0.13470	5.01114
H	1.00022	-1.65225	4.19341
C	-2.56335	0.06860	4.18209
H	-1.02229	1.55641	3.95782
H	-1.81634	1.12612	2.44970
C	-2.04113	-0.56114	5.48982
H	-0.50784	-1.95330	6.13677
H	-1.30025	-2.40483	4.63884
H	-3.32203	0.81483	4.40861
H	-3.03319	-0.69903	3.57099
H	-2.85311	-1.06616	6.00887
H	-1.67225	0.22389	6.14716
H	-0.74332	-1.10357	2.50735
H	-1.17829	2.03596	0.21078

6_c

176

XYZ

Ru	0.90433	0.52866	-0.41328
C	-1.31584	2.70462	0.31578
C	-0.54137	2.01399	-0.55395
P	-0.72328	-1.10161	-0.98411
N	2.14804	2.30376	-0.88137
C	1.95975	2.10585	-2.18115
C	2.34393	3.29617	-3.04989
C	3.00087	4.23807	-2.01020
C	2.48132	3.71031	-0.63397
O	1.38504	1.01246	-2.54895
P	3.08387	-0.71927	-0.04446
H	-1.41753	2.35072	1.35059
H	1.57569	4.26206	-0.30920
H	3.23929	3.81833	0.16238
H	3.00539	3.01145	-3.88679
H	1.42538	3.72702	-3.49382
H	2.74840	5.30037	-2.16835
H	4.10027	4.14651	-2.05538
H	-0.50737	2.41145	-1.59012
C	-2.02410	4.03267	0.06056
C	-2.00174	4.56985	-1.38023
H	-3.07677	3.97274	0.40971
H	-1.56400	4.79792	0.72575
C	-2.73658	5.91461	-1.53918
H	-0.95093	4.69020	-1.71268
H	-2.44984	3.82178	-2.06547
C	-2.70216	6.45641	-2.97815
H	-3.79014	5.79932	-1.21374
H	-2.28658	6.66001	-0.85256
H	-3.23379	7.42164	-3.06161
H	-1.66233	6.61548	-3.32037
H	-3.17806	5.74930	-3.68305
C	-2.52046	-0.52641	-1.18572
H	-2.40677	0.43046	-1.71725
H	-3.05694	-1.21848	-1.85361
P	-3.55757	-0.03423	0.33098
C	-4.66308	-1.57329	0.69464
C	-5.38816	-1.39723	2.05335
C	-5.68403	-2.05299	-0.36576
H	-3.94447	-2.37523	0.83556
C	-6.06108	-2.71395	2.50365
H	-6.14853	-0.62474	1.97594
H	-4.68106	-1.07576	2.81394
C	-6.34601	-3.38269	0.06410
H	-6.46554	-1.30860	-0.48882
H	-5.20551	-2.18627	-1.33348
C	-7.04210	-3.23851	1.43394
H	-6.59048	-2.55360	3.44085
H	-5.29521	-3.46494	2.68801
H	-7.07186	-3.68954	-0.68653
H	-5.58971	-4.16272	0.12306
H	-7.45020	-4.19817	1.74476
H	-7.87586	-2.54528	1.34065
C	-4.63627	1.32940	-0.50239
C	-5.28005	1.04658	-1.88459
C	-5.71193	1.87030	0.47120
H	-3.91023	2.12354	-0.65275
C	-5.93917	2.32532	-2.45144
H	-6.03938	0.27519	-1.80553
H	-4.53258	0.69527	-2.59060
C	-6.38788	3.14332	-0.08610
H	-6.47807	1.11621	0.63585
H	-5.26144	2.09438	1.43599
C	-6.99773	2.88832	-1.48027
H	-6.40166	2.10234	-3.41112
H	-5.17348	3.07851	-2.62580
H	-7.16387	3.47611	0.60060
H	-5.65082	3.94033	-0.15607
H	-7.41016	3.81323	-1.87838
H	-7.81787	2.17825	-1.39089
C	-1.06533	-2.75072	0.00128
C	-2.04518	-3.78356	-0.61334
C	0.22966	-3.48483	0.40906
H	-1.52639	-2.37535	0.91289
C	-2.36534	-4.90444	0.40284
H	-1.60394	-4.24762	-1.48659
H	-2.96773	-3.30999	-0.93097
C	-0.04908	-4.63496	1.40349
H	0.71751	-3.88939	-0.47449
H	0.91521	-2.78277	0.86783
C	-1.07878	-5.63449	0.83925
H	-3.05639	-5.61412	-0.04747
H	-2.85512	-4.48213	1.27763
H	0.88110	-5.15107	1.63329

H	-0.42755	-4.21797	2.33441
H	-1.31305	-6.38622	1.59008
H	-0.64977	-6.15088	-0.01727
C	-0.24647	-1.48537	-2.84751
C	-0.47829	-2.91675	-3.38884
C	-0.89234	-0.45958	-3.81201
H	0.82279	-1.29991	-2.86612
C	0.10506	-3.06556	-4.81402
H	-1.54162	-3.14132	-3.42102
H	-0.00124	-3.65179	-2.74676
C	-0.30226	-0.59646	-5.23246
H	-1.96683	-0.61543	-3.86396
H	-0.70708	0.54767	-3.45068
C	-0.49055	-2.02472	-5.78303
H	-0.09256	-4.07111	-5.18075
H	1.18532	-2.94108	-4.77239
H	-0.78398	0.12008	-5.89464
H	0.75750	-0.35269	-5.20196
H	-0.01585	-2.11553	-6.75788
H	-1.55203	-2.22418	-5.91754
P	1.22582	0.30674	1.88803
C	2.73995	-0.82980	1.81014
H	3.56306	-0.57424	2.49461
H	2.44651	-1.86770	2.03021
C	4.60200	0.46662	-0.24657
C	5.67565	0.44489	0.86962
C	5.26110	0.30728	-1.63804
H	4.12473	1.44208	-0.23107
C	6.73486	1.54467	0.62394
H	6.17418	-0.51884	0.91150
H	5.21899	0.61771	1.84012
C	6.32241	1.40412	-1.87814
H	5.74507	-0.66208	-1.71886
H	4.50144	0.36287	-2.41494
C	7.39009	1.40042	-0.76494
H	7.49600	1.49172	1.39991
H	6.26071	2.52135	0.69829
H	6.79498	1.24527	-2.84532
H	5.83600	2.37638	-1.90792
H	8.09494	2.21345	-0.92559
H	7.95177	0.46900	-0.80472
C	3.67404	-2.52818	-0.41552
C	3.65134	-2.86774	-1.92562
C	5.02804	-2.94144	0.20969
H	2.91214	-3.13963	0.05807
C	3.92276	-4.37209	-2.15522
H	4.40250	-2.28903	-2.45540
H	2.68491	-2.60671	-2.34892
C	5.28767	-4.44976	-0.00910
H	5.84076	-2.38181	-0.24382
H	5.03376	-2.72669	1.27619
C	5.25429	-4.80944	-1.50936
H	3.94541	-4.57796	-3.22338
H	3.10812	-4.95428	-1.72881
H	6.25437	-4.71610	0.41356
H	4.53088	-5.02676	0.51890
H	5.39023	-5.88163	-1.63434
H	6.08064	-4.31468	-2.01604
C	2.03437	1.85676	2.73985
C	1.12425	3.10618	2.72079
C	2.58997	1.64637	4.17041
H	2.87888	2.06001	2.08381
C	1.89609	4.35907	3.19373
H	0.27069	2.95400	3.37551
H	0.74031	3.26647	1.71821
C	3.38909	2.88718	4.63060
H	1.76972	1.49183	4.86586
H	3.23197	0.77044	4.21957
C	2.51074	4.15486	4.59427
H	1.22194	5.21285	3.20884
H	2.68924	4.58436	2.48438
H	3.76354	2.72345	5.63928
H	4.25144	3.02338	3.98076
H	3.10522	5.02358	4.86959
H	1.71387	4.05904	5.32926
C	0.06472	-0.60074	3.15951
C	0.77079	-1.30706	4.34620
C	-1.06634	0.31766	3.67935
C	-0.24353	-2.09968	5.20238
H	1.26287	-0.57958	4.98294
H	1.53403	-1.99136	3.98672
C	-2.07366	-0.47340	4.54351
H	-0.64662	1.12030	4.27977
H	-1.59131	0.76838	2.84071
C	-1.37430	-1.18693	5.71810
H	0.27553	-2.55936	6.04113
H	-0.67236	-2.90242	4.60595
H	-2.83596	0.20461	4.92143
H	-2.57377	-1.21386	3.92342
H	-2.09764	-1.77558	6.27846
H	-0.95871	-0.44601	6.39829
H	-0.39999	-1.37628	2.55608

[6_c-$7a_c$]‡

176

XYZ

Ru	0.66318	-0.30214	-0.54907
C	-1.22155	-1.82187	-2.62708
C	-0.56803	-1.13800	-1.68326
P	-1.04710	-0.40591	1.22391
N	1.94929	-0.86238	-2.23058
C	2.47287	-2.12222	-2.19527
C	2.93586	-2.54132	-3.61087
C	2.85118	-1.24020	-4.42068

C	1.76849	-0.45749	-3.64056
O	2.55989	-2.88148	-1.19782
P	2.87198	-0.06255	0.77372
H	-1.84120	-1.16567	-3.26028
H	0.75359	-0.74027	-3.98998
H	1.86000	0.62764	-3.77241
H	3.93070	-3.01623	-3.57728
H	2.22404	-3.30525	-3.98341
H	2.58878	-1.38042	-5.48464
H	3.81486	-0.69826	-4.37770
H	0.37009	-1.96073	-0.70342
C	-1.15796	-3.28143	-3.06382
C	-0.60457	-4.29723	-2.04966
H	-2.17479	-3.59777	-3.37639
H	-0.54627	-3.33250	-3.99177
C	-0.53218	-5.72216	-2.63261
H	0.40383	-3.99101	-1.71137
H	-1.25295	-4.30550	-1.15118
C	0.00409	-6.75214	-1.62447
H	-1.53717	-6.03320	-2.98520
H	0.11852	-5.71401	-3.53050
H	0.06186	-7.76215	-2.06945
H	1.01598	-6.47484	-1.27743
H	-0.64861	-6.81551	-0.73362
C	-2.76758	-0.87118	0.57371
H	-2.58210	-1.85614	0.11650
H	-3.46737	-1.02571	1.40682
P	-3.53976	0.07922	-0.88604
C	-4.72915	1.37569	-0.09806
C	-5.18658	2.38179	-1.18540
C	-5.96284	0.89112	0.70211
H	-4.09234	1.93414	0.58395
C	-5.94948	3.57462	-0.56483
H	-5.83463	1.88718	-1.90430
H	-4.32269	2.75151	-1.73352
C	-6.71062	2.08039	1.34736
H	-6.65179	0.37689	0.03795
H	-5.67066	0.18560	1.47677
C	-7.15261	3.10002	0.27685
H	-6.29236	4.24037	-1.35444
H	-5.27310	4.14406	0.06933
H	-7.58046	1.71486	1.88974
H	-6.05998	2.57145	2.06836
H	-7.63042	3.95383	0.75303
H	-7.88960	2.63695	-0.37646
C	-4.57596	-1.37072	-1.62726
C	-5.42946	-2.25844	-0.68503
C	-5.44145	-0.89319	-2.81883
H	-3.79769	-2.01013	-2.03669
C	-6.00907	-3.46729	-1.45588
H	-6.25299	-1.69168	-0.26212
H	-4.82957	-2.62634	0.14295

C	-6.03193	-2.09147	-3.59555
H	-6.26009	-0.27273	-2.46120
H	-4.84205	-0.28598	-3.49411
C	-6.85117	-3.01094	-2.66574
H	-6.62217	-4.06717	-0.78594
H	-5.19251	-4.09773	-1.80235
H	-6.66278	-1.72895	-4.40485
H	-5.22138	-2.66215	-4.04437
H	-7.20228	-3.87914	-3.21975
H	-7.72925	-2.47446	-2.31088
C	-1.44724	1.09572	2.41122
C	-2.83151	1.12520	3.11033
C	-0.33682	1.30143	3.47073
H	-1.42769	1.95193	1.74389
C	-3.03246	2.45234	3.87798
H	-2.93249	0.30830	3.81418
H	-3.62756	1.02059	2.38495
C	-0.53133	2.61327	4.26466
H	-0.32998	0.46982	4.16997
H	0.63533	1.32143	2.99144
C	-1.92267	2.66839	4.92626
H	-4.00429	2.43966	4.36716
H	-3.03323	3.28326	3.17554
H	0.24434	2.69458	5.02355
H	-0.41930	3.46242	3.59385
H	-2.05920	3.62809	5.42019
H	-1.99112	1.89544	5.68921
C	-0.73250	-1.91265	2.44617
C	-1.75145	-2.07821	3.60382
C	-0.61784	-3.24942	1.67051
H	0.23654	-1.70103	2.89144
C	-1.39426	-3.26954	4.52340
H	-2.75123	-2.23159	3.20526
H	-1.77587	-1.18770	4.21973
C	-0.25900	-4.43429	2.59419
H	-1.56433	-3.47001	1.18476
H	0.13524	-3.16476	0.89457
C	-1.28175	-4.58770	3.73556
H	-2.15401	-3.35899	5.29745
H	-0.44770	-3.06639	5.02043
H	-0.22178	-5.34553	2.00122
H	0.73111	-4.28252	3.01766
H	-0.98233	-5.39346	4.40246
H	-2.25410	-4.84815	3.32220
P	1.41627	2.03634	-0.49173
C	2.67138	1.82230	0.89800
H	3.57137	2.45275	0.88372
H	2.14271	2.03656	1.83981
C	4.52731	-0.30214	-0.20476
C	5.67124	0.69734	0.10492
C	5.06622	-1.74906	-0.09585
H	4.21138	-0.14703	-1.23411

C	6.84435	0.49155	-0.88161
H	6.03725	0.55389	1.11712
H	5.33262	1.72449	0.02675
C	6.23505	-1.96621	-1.08149
H	5.42807	-1.93317	0.91147
H	4.25896	-2.44660	-0.32258
C	7.37485	-0.95597	-0.83518
H	7.64422	1.18884	-0.63963
H	6.50695	0.72034	-1.89062
H	6.61291	-2.98094	-0.97549
H	5.87140	-1.85930	-2.10107
H	8.15609	-1.08465	-1.58144
H	7.82118	-1.14364	0.13959
C	3.13120	-0.57040	2.61551
C	3.17040	-2.10388	2.82063
C	4.33033	0.08485	3.34243
H	2.22724	-0.20609	3.09773
C	3.12336	-2.44751	4.32686
H	4.08125	-2.51585	2.39924
H	2.33805	-2.57407	2.30438
C	4.28932	-0.24406	4.85249
H	5.26355	-0.28963	2.93131
H	4.31948	1.16431	3.20723
C	4.28008	-1.76897	5.09145
H	3.18106	-3.52633	4.45547
H	2.17396	-2.11955	4.74552
H	5.15123	0.20165	5.34505
H	3.39770	0.19778	5.29337
H	4.19001	-1.97382	6.15621
H	5.22663	-2.18955	4.75762
C	2.45139	2.65578	-2.01009
C	1.53846	3.21901	-3.12731
C	3.59925	3.65847	-1.72682
H	2.90701	1.74050	-2.37670
C	2.34247	3.48920	-4.41914
H	1.09252	4.15430	-2.80028
H	0.72908	2.52449	-3.33918
C	4.41641	3.91369	-3.01461
H	3.20705	4.60532	-1.37110
H	4.26774	3.27717	-0.96264
C	3.52204	4.44679	-4.15255
H	1.68227	3.91600	-5.17131
H	2.72220	2.54951	-4.81372
H	5.20951	4.62760	-2.80120
H	4.88878	2.98557	-3.33023
H	4.11195	4.56821	-5.05852
H	3.13803	5.42785	-3.87924
C	0.32906	3.50388	0.13414
C	1.02722	4.88158	0.23231
C	-0.99523	3.62759	-0.65795
C	0.09581	5.91718	0.90206
H	1.28317	5.24445	-0.75836
H	1.94898	4.80028	0.80449
C	-1.92861	4.67927	-0.01581
H	-0.79103	3.92166	-1.68307
H	-1.49830	2.66352	-0.69600
C	-1.23102	6.04838	0.12612
H	0.59762	6.88179	0.94340
H	-0.10984	5.61270	1.92634
H	-2.82397	4.78569	-0.62380
H	-2.24168	4.33381	0.96708
H	-1.89064	6.74628	0.63749
H	-1.02951	6.45516	-0.86292
H	0.07862	3.20846	1.14873

$7a_c$

176

XYZ

Ru	-0.82205	-0.58362	-0.23173
C	1.02762	-3.12048	0.08077
C	0.35901	-1.97970	-0.10753
P	0.89012	0.81289	-1.22277
N	-2.25561	-2.26882	-0.34538
C	-2.64840	-2.63998	-1.60229
C	-3.24712	-4.06290	-1.57216
C	-3.45705	-4.34245	-0.07756
C	-2.42308	-3.39606	0.58754
O	-2.53876	-1.97350	-2.65809
P	-2.96500	0.88296	-0.04151
H	1.43340	-3.27447	1.09366
H	-1.45551	-3.91488	0.75167
H	-2.77688	-3.04730	1.57111
H	-4.15633	-4.12512	-2.19326
H	-2.50120	-4.74905	-2.02100
H	-3.30999	-5.39823	0.21205
H	-4.48094	-4.05423	0.22538
H	-1.17789	-0.80592	-1.78883
C	1.15884	-4.30156	-0.87890
C	0.94065	-3.98415	-2.36847
H	2.15898	-4.76271	-0.74731
H	0.43412	-5.08682	-0.56955
C	0.98029	-5.24316	-3.25503
H	-0.02736	-3.46554	-2.50483
H	1.72360	-3.27508	-2.70569
C	0.79245	-4.93027	-4.74911
H	1.94283	-5.77334	-3.10327
H	0.18890	-5.94564	-2.92328
H	0.80793	-5.85080	-5.36032
H	-0.17101	-4.41953	-4.92912
H	1.59549	-4.26721	-5.12176
C	2.62149	0.03527	-1.30280
H	2.43612	-0.99992	-1.62876
H	3.22894	0.52231	-2.07939
P	3.53286	-0.13834	0.36060

C	4.96650	1.15247	0.29841
C	5.91691	0.95496	1.50598
C	5.80948	1.31846	-0.99245
H	4.45269	2.09650	0.46424
C	6.89274	2.14461	1.65030
H	6.49557	0.04356	1.37814
H	5.34029	0.84808	2.42240
C	6.77668	2.51828	-0.86396
H	6.39326	0.42363	-1.18166
H	5.17109	1.47263	-1.85814
C	7.70675	2.35970	0.35714
H	7.56759	1.96561	2.48505
H	6.32868	3.04764	1.87586
H	7.37206	2.60537	-1.77076
H	6.20204	3.43693	-0.76278
H	8.33707	3.24080	0.45886
H	8.36326	1.50611	0.19973
C	4.26355	-1.90511	0.16086
C	5.39877	-2.12824	-0.86693
C	4.67897	-2.48400	1.53655
H	3.40415	-2.47953	-0.17384
C	5.73489	-3.63165	-0.99472
H	6.29728	-1.60314	-0.55397
H	5.11221	-1.73945	-1.84191
C	5.03459	-3.98410	1.42643
H	5.53833	-1.94642	1.93020
H	3.86579	-2.35984	2.24843
C	6.13424	-4.22801	0.37143
H	6.54697	-3.76551	-1.70686
H	4.86880	-4.16360	-1.38310
H	5.36563	-4.35380	2.39502
H	4.14271	-4.54329	1.15162
H	6.31707	-5.29576	0.26926
H	7.06318	-3.76941	0.70551
C	1.37611	2.56616	-0.52137
C	2.48798	3.36770	-1.24470
C	0.14428	3.47810	-0.32971
H	1.75905	2.31920	0.46699
C	2.87337	4.62223	-0.42771
H	2.14817	3.69968	-2.21724
H	3.36813	2.75605	-1.40239
C	0.49414	4.77195	0.44082
H	-0.27415	3.74256	-1.29796
H	-0.62146	2.94041	0.21745
C	1.65075	5.54099	-0.22889
H	3.66094	5.16415	-0.94729
H	3.26625	4.32578	0.54276
H	-0.38723	5.40735	0.50222
H	0.78009	4.51512	1.45824
H	1.92370	6.39936	0.38137
H	1.32342	5.91822	-1.19573
C	0.41894	0.96454	-3.11450

C	0.90172	2.21041	-3.89517
C	0.83381	-0.31848	-3.87448
H	-0.66604	0.98630	-3.09661
C	0.33298	2.20877	-5.33419
H	1.98795	2.23116	-3.94450
H	0.57139	3.12082	-3.40453
C	0.25436	-0.32534	-5.30519
H	1.91745	-0.38268	-3.93513
H	0.46873	-1.18932	-3.33969
C	0.70353	0.92045	-6.09520
H	0.70932	3.07914	-5.86847
H	-0.75049	2.29915	-5.28757
H	0.57906	-1.22677	-5.82047
H	-0.83185	-0.35374	-5.25063
H	0.23801	0.92828	-7.07855
H	1.78117	0.88721	-6.24449
P	-1.12650	0.21025	2.14153
C	-2.57251	1.36269	1.74620
H	-3.40719	1.32695	2.46177
H	-2.20928	2.40257	1.73531
C	-4.53756	-0.23372	0.01298
C	-5.56734	0.06885	1.12944
C	-5.22381	-0.30186	-1.37261
H	-4.09872	-1.21070	0.19842
C	-6.70214	-0.98167	1.11747
H	-6.00148	1.05646	1.00150
H	-5.08995	0.04717	2.10536
C	-6.36052	-1.34765	-1.37178
H	-5.64640	0.66393	-1.63552
H	-4.48683	-0.57100	-2.12735
C	-7.39035	-1.05698	-0.26070
H	-7.43217	-0.73116	1.88482
H	-6.28839	-1.95683	1.36644
H	-6.85259	-1.34534	-2.34212
H	-5.93806	-2.33839	-1.22270
H	-8.15198	-1.83379	-0.24810
H	-7.89104	-0.11263	-0.46635
C	-3.43303	2.60582	-0.78505
C	-3.43040	2.61146	-2.33276
C	-4.74099	3.23184	-0.24294
H	-2.61987	3.24957	-0.46250
C	-3.61404	4.04719	-2.87509
H	-4.22978	1.98380	-2.71501
H	-2.49466	2.20085	-2.70209
C	-4.91492	4.67135	-0.77919
H	-5.59825	2.64068	-0.55128
H	-4.72858	3.25263	0.84478
C	-4.89930	4.69802	-2.32199
H	-3.65202	4.02132	-3.96211
H	-2.75450	4.65257	-2.59351
H	-5.85233	5.08369	-0.41132
H	-4.11114	5.29821	-0.39767

H	-4.97371	5.72512	-2.67305
H	-5.76691	4.16040	-2.69949
C	-1.89533	-1.02139	3.41506
C	-0.97098	-2.23485	3.68033
C	-2.37483	-0.42813	4.76127
H	-2.77427	-1.38420	2.88700
C	-1.70987	-3.31338	4.50459
H	-0.08995	-1.91582	4.23021
H	-0.63449	-2.65990	2.73858
C	-3.14729	-1.49874	5.56571
H	-1.52155	-0.10798	5.35188
H	-3.01341	0.43786	4.60272
C	-2.26451	-2.73776	5.82506
H	-1.02762	-4.13335	4.71844
H	-2.52962	-3.71794	3.91512
H	-3.47546	-1.07293	6.51185
H	-4.03745	-1.79382	5.01359
H	-2.84248	-3.49862	6.34528
H	-1.43679	-2.45717	6.47369
C	0.14076	1.36590	3.03834
C	-0.43545	2.33635	4.10062
C	1.33277	0.57117	3.62293
C	0.66427	3.29497	4.61316
H	-0.83065	1.78544	4.94673
H	-1.25081	2.92169	3.68366
C	2.42975	1.52748	4.14154
H	0.99487	-0.05087	4.44728
H	1.75406	-0.08089	2.86012
C	1.86732	2.51600	5.18402
H	0.24552	3.94379	5.37979
H	1.00077	3.93113	3.79746
H	3.23666	0.94551	4.58178
H	2.84778	2.08289	3.30446
H	2.64465	3.21279	5.49034
H	1.55353	1.96718	6.06985
H	0.52673	1.97674	2.22701

[$7a_c$-$8a_c$]‡

176

XYZ

Ru	0.77971	0.44197	-0.38105
C	-1.34727	2.82420	-0.03343
C	-0.48823	1.82587	-0.25728
P	-0.67068	-1.36464	-0.82517
N	1.31167	2.47177	-1.21488
C	1.15343	2.70514	-2.57094
C	1.76570	4.08014	-2.92818
C	2.05108	4.76535	-1.58081
C	1.99365	3.59452	-0.55226
O	0.65017	1.94883	-3.41470
P	3.00576	-0.66097	-0.26039
H	-2.15862	2.50965	0.64535
H	1.45405	3.87951	0.36773
H	3.01797	3.29503	-0.25511
H	2.68481	3.89487	-3.51577
H	1.07554	4.63556	-3.58586
H	1.27776	5.51908	-1.35137
H	3.02550	5.28338	-1.55118
H	0.60216	0.35768	-1.97148
C	-1.36588	4.26609	-0.50027
C	-2.73717	4.73525	-1.03266
H	-1.07670	4.93511	0.34035
H	-0.60795	4.40273	-1.28879
C	-2.74006	6.22167	-1.43813
H	-3.02035	4.11329	-1.90413
H	-3.51231	4.56481	-0.25810
C	-4.10036	6.70071	-1.97229
H	-2.44644	6.83882	-0.56484
H	-1.96086	6.39023	-2.20851
H	-4.06981	7.76800	-2.25678
H	-4.40406	6.12434	-2.86582
H	-4.89406	6.57921	-1.21168
C	-2.45911	-0.84384	-1.17378
H	-2.31662	-0.03215	-1.90623
H	-3.00083	-1.65707	-1.68218
P	-3.49618	0.00938	0.16855
C	-4.55570	-1.37410	0.98556
C	-5.20533	-0.83374	2.28524
C	-5.62530	-2.11014	0.14240
H	-3.82235	-2.11210	1.29761
C	-5.85907	-1.97231	3.10075
H	-5.96406	-0.09294	2.04731
H	-4.45350	-0.33860	2.89538
C	-6.27188	-3.26197	0.94612
H	-6.40813	-1.41539	-0.14932
H	-5.18870	-2.51012	-0.77018
C	-6.89733	-2.74239	2.25776
H	-6.33737	-1.56057	3.98733
H	-5.08771	-2.66185	3.43845
H	-7.03575	-3.74623	0.34081
H	-5.51726	-4.01084	1.17773
H	-7.29631	-3.57554	2.83286
H	-7.72947	-2.08200	2.02139
C	-4.62014	1.07659	-0.98157
C	-5.22420	0.42268	-2.25154
C	-5.73702	1.78733	-0.17762
H	-3.93022	1.84610	-1.32227
C	-5.95151	1.47512	-3.11973
H	-5.93361	-0.35394	-1.98251
H	-4.44447	-0.04211	-2.84828
C	-6.47822	2.83469	-1.03890
H	-6.46137	1.05781	0.17670
H	-5.31228	2.27573	0.69712
C	-7.05517	2.19794	-2.32010

H	-6.38570	0.99003	-3.99185
H	-5.22959	2.20568	-3.47933
H	-7.28006	3.28447	-0.45652
H	-5.78837	3.62980	-1.31244
H	-7.51806	2.96479	-2.93789
H	-7.83152	1.48413	-2.05083
C	-0.97136	-2.82387	0.44309
C	-2.04562	-3.89426	0.11804
C	0.33491	-3.55261	0.83527
H	-1.31510	-2.28591	1.32525
C	-2.29664	-4.80588	1.34169
H	-1.71926	-4.52274	-0.70145
H	-2.97940	-3.43463	-0.18485
C	0.12439	-4.49357	2.04412
H	0.69829	-4.14073	-0.00352
H	1.10585	-2.82990	1.07837
C	-0.99760	-5.51681	1.77378
H	-3.05498	-5.54608	1.09447
H	-2.67764	-4.21306	2.17078
H	1.05354	-5.01434	2.26784
H	-0.13528	-3.90223	2.91961
H	-1.17721	-6.11183	2.66684
H	-0.68203	-6.19807	0.98599
C	-0.18122	-2.12445	-2.56899
C	-0.54225	-3.60834	-2.82957
C	-0.72059	-1.25810	-3.73681
H	0.90175	-2.04180	-2.58022
C	0.01762	-4.09072	-4.18909
H	-1.62208	-3.73668	-2.83064
H	-0.13330	-4.24690	-2.05309
C	-0.13900	-1.72776	-5.08795
H	-1.80339	-1.33613	-3.78562
H	-0.45486	-0.21607	-3.57675
C	-0.47063	-3.20964	-5.35555
H	-0.27990	-5.12492	-4.35234
H	1.10480	-4.06794	-4.15507
H	-0.54173	-1.10863	-5.88673
H	0.94037	-1.59137	-5.08477
H	-0.00721	-3.53815	-6.28363
H	-1.54676	-3.32552	-5.46931
P	1.57940	0.43767	1.97300
C	3.02044	-0.74158	1.62868
H	3.98594	-0.50926	2.10244
H	2.72584	-1.74569	1.96917
C	4.47085	0.55157	-0.59708
C	5.88530	0.06029	-0.20183
C	4.47111	1.06740	-2.05598
H	4.23079	1.39683	0.04432
C	6.91799	1.19876	-0.36789
H	6.18420	-0.77084	-0.83416
H	5.90044	-0.28915	0.82803
C	5.51100	2.19577	-2.23871
H	4.70940	0.25780	-2.73949
H	3.48056	1.43320	-2.31101
C	6.92262	1.73940	-1.81313
H	7.90800	0.83042	-0.10605
H	6.67791	2.00645	0.32091
H	5.52413	2.50932	-3.28038
H	5.21706	3.05757	-1.64345
H	7.62022	2.57057	-1.89210
H	7.26915	0.95804	-2.48683
C	3.52154	-2.43544	-0.85064
C	4.02689	-2.45056	-2.31347
C	4.51657	-3.18920	0.06772
H	2.58597	-2.98782	-0.83130
C	4.22601	-3.90078	-2.80863
H	4.97610	-1.92777	-2.38628
H	3.31860	-1.93758	-2.96042
C	4.72244	-4.63934	-0.42776
H	5.47736	-2.68354	0.08375
H	4.14709	-3.21743	1.08921
C	5.19952	-4.67322	-1.89449
H	4.60754	-3.88590	-3.82741
H	3.26593	-4.41214	-2.82678
H	5.44949	-5.13826	0.20990
H	3.78463	-5.18463	-0.34076
H	5.28277	-5.70465	-2.23040
H	6.18981	-4.22695	-1.96216
C	2.41168	2.02272	2.72168
C	1.35529	2.97720	3.32901
C	3.58622	1.82327	3.71242
H	2.82099	2.51263	1.84294
C	1.98732	4.33964	3.69110
H	0.93442	2.53903	4.23029
H	0.54115	3.12857	2.62324
C	4.22243	3.18751	4.06824
H	3.24584	1.34746	4.62634
H	4.35203	1.18630	3.27885
C	3.18011	4.16278	4.65357
H	1.23432	4.97788	4.14871
H	2.32514	4.83310	2.78217
H	5.02779	3.03318	4.78363
H	4.66104	3.62315	3.17259
H	3.64652	5.12667	4.84584
H	2.82198	3.77781	5.60652
C	0.61073	-0.48482	3.36590
C	1.32749	-0.61947	4.73019
C	-0.81344	0.09001	3.56009
C	0.51354	-1.52313	5.68401
H	1.43556	0.35745	5.19244
H	2.32399	-1.03605	4.59853
C	-1.63391	-0.79359	4.52750
H	-0.75933	1.09716	3.96382
H	-1.32292	0.15259	2.60043

C	-0.91488	-0.97415	5.88117
H	1.02151	-1.58679	6.64422
H	0.46337	-2.53004	5.27440
H	-2.61050	-0.34275	4.68926
H	-1.79703	-1.76936	4.07429
H	-1.48555	-1.65037	6.51419
H	-0.86278	-0.01495	6.39263
H	0.49997	-1.48657	2.95790

8a$_c$

176

XYZ

Ru	0.96581	0.50170	-0.29389
C	-1.27115	2.68904	0.62269
C	-0.13581	2.13428	0.12954
P	-0.67679	-0.77332	-1.28977
N	1.00571	2.97479	-0.24992
C	1.00598	3.64755	-1.50698
C	2.02444	4.79551	-1.43773
C	2.77791	4.59653	-0.11260
C	1.81594	3.73649	0.74000
O	0.27435	3.39550	-2.45491
P	3.13917	-0.60442	-0.31461
H	-2.04219	1.98630	0.96650
H	1.15378	4.36953	1.36032
H	2.34621	3.04681	1.40744
H	2.66411	4.78932	-2.33597
H	1.45063	5.74162	-1.45773
H	3.02867	5.54257	0.39552
H	3.72090	4.05282	-0.28367
H	1.11614	0.89711	-1.83916
C	-1.60085	4.16670	0.74833
C	-2.47166	4.72939	-0.40497
H	-2.12605	4.34806	1.70940
H	-0.67769	4.77685	0.78939
C	-2.75544	6.23707	-0.26187
H	-1.96112	4.52878	-1.36616
H	-3.43408	4.18374	-0.44493
C	-3.61902	6.79999	-1.40340
H	-3.25461	6.42719	0.71013
H	-1.79297	6.78738	-0.22189
H	-3.79848	7.88365	-1.28069
H	-3.13133	6.64775	-2.38401
H	-4.60469	6.29978	-1.44214
C	-2.49922	-0.21399	-1.20749
H	-2.42050	0.85601	-1.46680
H	-3.05794	-0.71161	-2.01658
P	-3.48296	-0.13894	0.41784
C	-4.42349	-1.80973	0.59328
C	-5.01565	-1.92555	2.02135
C	-5.51418	-2.18193	-0.44077
H	-3.64087	-2.55977	0.52708
C	-5.55813	-3.34783	2.28916
H	-5.82538	-1.21285	2.15178
H	-4.25457	-1.68432	2.75904
C	-6.05227	-3.61030	-0.19360
H	-6.34594	-1.48723	-0.36438
H	-5.12600	-2.11411	-1.45463
C	-6.61212	-3.75356	1.23738
H	-5.99576	-3.39241	3.28459
H	-4.73412	-4.05839	2.26467
H	-6.83217	-3.83760	-0.91785
H	-5.25096	-4.33104	-0.34171
H	-6.93075	-4.77974	1.40928
H	-7.48927	-3.11827	1.34456
C	-4.74705	1.23099	-0.08629
C	-5.43764	1.14825	-1.47229
C	-5.81140	1.45711	1.01541
H	-4.12033	2.11832	-0.10576
C	-6.26098	2.42691	-1.75271
H	-6.10263	0.29170	-1.52096
H	-4.69789	1.03082	-2.25931
C	-6.63667	2.73501	0.74382
H	-6.49140	0.60971	1.05828
H	-5.32962	1.53885	1.98767
C	-7.30548	2.68070	-0.64562
H	-6.76058	2.33423	-2.71515
H	-5.58913	3.28037	-1.81648
H	-7.39614	2.85170	1.51459
H	-5.98303	3.60336	0.79620
H	-7.83111	3.61342	-0.84013
H	-8.04484	1.88198	-0.65793
C	-0.88889	-2.64449	-0.75249
C	-1.96071	-3.52445	-1.44644
C	0.45553	-3.40233	-0.77502
H	-1.18795	-2.54669	0.28956
C	-2.10291	-4.88684	-0.72873
H	-1.68163	-3.72537	-2.47274
H	-2.92274	-3.02573	-1.46972
C	0.35529	-4.79243	-0.10547
H	0.79268	-3.52686	-1.80158
H	1.20522	-2.81434	-0.25868
C	-0.76193	-5.64889	-0.73521
H	-2.86576	-5.48280	-1.22574
H	-2.42665	-4.73341	0.29867
H	1.30950	-5.30915	-0.19050
H	0.14968	-4.66568	0.95517
H	-0.86083	-6.58528	-0.18988
H	-0.49407	-5.89530	-1.76079
C	-0.41291	-0.68722	-3.23234
C	-0.91732	-1.86666	-4.09934
C	-0.96759	0.64653	-3.79257
H	0.66686	-0.65146	-3.33131
C	-0.52787	-1.66961	-5.58442

H	-1.99811	-1.96389	-4.02548
H	-0.47506	-2.79936	-3.76415
C	-0.54815	0.84418	-5.26519
H	-2.05310	0.64688	-3.74638
H	-0.59928	1.48022	-3.19935
C	-1.03609	-0.32592	-6.14321
H	-0.92824	-2.49334	-6.17269
H	0.55627	-1.70572	-5.67298
H	-0.95945	1.78096	-5.63530
H	0.53560	0.91750	-5.32712
H	-0.68975	-0.19945	-7.16705
H	-2.12418	-0.33255	-6.16566
P	1.46337	-0.36901	2.00345
C	3.05291	-1.24888	1.45875
H	3.93488	-1.10371	2.10259
H	2.86946	-2.33453	1.40228
C	4.48739	0.79870	-0.31411
C	5.69036	0.62699	0.64784
C	4.97209	1.09757	-1.75429
H	3.92734	1.66735	0.02411
C	6.61638	1.86433	0.59832
H	6.26936	-0.25367	0.38742
H	5.34193	0.49652	1.66893
C	5.91927	2.31746	-1.80131
H	5.50612	0.23919	-2.15093
H	4.11301	1.27912	-2.39708
C	7.10967	2.14283	-0.83613
H	7.46655	1.70388	1.25838
H	6.07608	2.73325	0.96903
H	6.28393	2.44828	-2.81803
H	5.36975	3.21724	-1.53683
H	7.72796	3.03796	-0.84567
H	7.73021	1.31394	-1.17135
C	3.87308	-2.14604	-1.23730
C	3.67407	-2.07428	-2.77011
C	5.33678	-2.51327	-0.89461
H	3.25402	-2.96258	-0.87527
C	4.07035	-3.41135	-3.43667
H	4.27646	-1.27605	-3.19399
H	2.63484	-1.84950	-2.99558
C	5.72334	-3.85759	-1.55360
H	6.01538	-1.74493	-1.25314
H	5.46557	-2.59164	0.18289
C	5.51869	-3.80960	-3.08242
H	3.96536	-3.32389	-4.51607
H	3.39073	-4.19423	-3.10559
H	6.76261	-4.08657	-1.32632
H	5.11405	-4.65440	-1.13133
H	5.75101	-4.78043	-3.51510
H	6.20783	-3.08664	-3.51467
C	2.12225	0.88384	3.33032
C	1.02615	1.88588	3.76908
C	2.82460	0.31019	4.58406
H	2.87238	1.43982	2.77061
C	1.64004	3.03554	4.59979
H	0.28356	1.37579	4.37597
H	0.51203	2.28831	2.89957
C	3.46969	1.45349	5.40130
H	2.10096	-0.19455	5.21717
H	3.58783	-0.41456	4.31005
C	2.41353	2.49735	5.82266
H	0.84954	3.70583	4.93064
H	2.31554	3.61405	3.97314
H	3.95334	1.04016	6.28419
H	4.24035	1.93569	4.80293
H	2.89728	3.31977	6.34549
H	1.71382	2.03665	6.51733
C	0.49188	-1.78985	2.89703
C	1.30887	-2.69593	3.85305
C	-0.77972	-1.27044	3.60919
C	0.44544	-3.87742	4.35159
H	1.64748	-2.13264	4.71552
H	2.19075	-3.08746	3.35340
C	-1.64203	-2.44597	4.12220
H	-0.50150	-0.64879	4.45598
H	-1.36655	-0.65760	2.92703
C	-0.83450	-3.37598	5.05120
H	1.02995	-4.48530	5.03930
H	0.17346	-4.50965	3.50895
H	-2.50650	-2.05492	4.65433
H	-2.01183	-3.01847	3.27419
H	-1.44813	-4.22347	5.34924
H	-0.56219	-2.83603	5.95602
H	0.16784	-2.41304	2.06755

[$8a_c$-$9a_c$]‡

176

XYZ

Ru	-0.80221	0.14697	0.00279
C	0.22893	2.50637	-1.41076
C	-0.94352	1.99318	-0.90959
P	1.06646	0.45284	1.46055
N	-2.15119	2.68674	-1.28136
C	-2.73348	3.65614	-0.48161
C	-3.88863	4.29028	-1.28052
C	-3.69321	3.78496	-2.72660
C	-2.78746	2.52740	-2.59482
O	-2.37903	3.94443	0.66522
P	-3.02098	-0.53370	0.33685
H	1.16490	2.02827	-1.09884
H	-2.02067	2.47669	-3.38805
H	-3.36348	1.58384	-2.63462
H	-4.85273	3.97751	-0.83899
H	-3.83719	5.38755	-1.18220

H	-3.17289	4.55212	-3.32743
H	-4.64086	3.55239	-3.24038
H	-1.15128	1.67690	0.56843
C	0.38355	3.63682	-2.40415
C	1.43967	4.67977	-1.96549
H	0.68093	3.24219	-3.40179
H	-0.58617	4.14913	-2.54913
C	1.65663	5.79734	-3.00329
H	1.12802	5.12361	-0.99996
H	2.40504	4.16743	-1.77425
C	2.70409	6.83381	-2.56318
H	1.96365	5.34534	-3.96826
H	0.69132	6.30696	-3.19743
H	2.83774	7.62316	-3.32486
H	2.40651	7.32561	-1.61855
H	3.68914	6.35971	-2.39515
C	2.85059	0.82117	0.88102
H	2.80251	1.72997	0.26124
H	3.47926	1.06542	1.75192
P	3.68238	-0.59713	-0.06925
C	5.50791	-0.42627	0.53377
C	6.43972	-1.39029	-0.24150
C	6.15752	0.97943	0.60953
H	5.45372	-0.79823	1.55589
C	7.83465	-1.47242	0.41855
H	6.55768	-1.04764	-1.26685
H	5.99937	-2.38478	-0.27941
C	7.54558	0.90214	1.28654
H	6.28053	1.39706	-0.38447
H	5.52786	1.66392	1.17182
C	8.47877	-0.07553	0.54170
H	8.47883	-2.12548	-0.16694
H	7.73785	-1.91338	1.40882
H	7.99468	1.89308	1.31073
H	7.42717	0.57333	2.31728
H	9.42898	-0.15201	1.06629
H	8.68644	0.31377	-0.45316
C	3.53011	-0.01852	-1.89625
C	4.34290	1.21900	-2.34720
C	3.81233	-1.20121	-2.85763
H	2.47510	0.23239	-1.99529
C	3.99930	1.60800	-3.80263
H	5.40642	1.00196	-2.29299
H	4.14473	2.06522	-1.69365
C	3.49075	-0.82672	-4.32225
H	4.85745	-1.49422	-2.79084
H	3.21842	-2.06538	-2.56914
C	4.26376	0.43241	-4.76611
H	4.59464	2.46918	-4.09932
H	2.95245	1.89805	-3.86114
H	3.73719	-1.66190	-4.97512
H	2.42322	-0.64142	-4.41794

H	3.96967	0.70783	-5.77681
H	5.32986	0.21377	-4.78511
C	1.35833	-1.11553	2.57536
C	2.68432	-1.19383	3.37494
C	0.14834	-1.36522	3.50877
H	1.39066	-1.92403	1.85106
C	2.79931	-2.53858	4.12949
H	2.75611	-0.38596	4.09446
H	3.53019	-1.10651	2.70230
C	0.27469	-2.69484	4.28623
H	0.05350	-0.55172	4.22277
H	-0.76289	-1.37783	2.91826
C	1.59967	-2.76273	5.07153
H	3.72565	-2.55153	4.70026
H	2.84978	-3.35348	3.40998
H	-0.567050	-2.79529	4.96851
H	0.22917	-3.53005	3.59049
H	1.68951	-3.72908	5.56335
H	1.60081	-1.99957	5.84730
C	0.76531	1.97882	2.64003
C	1.56832	2.01555	3.96492
C	0.98774	3.30395	1.87003
H	-0.29112	1.91231	2.88819
C	1.15245	3.23281	4.82468
H	2.63500	2.07395	3.75950
H	1.39405	1.11847	4.54913
C	0.55411	4.51691	2.72024
H	2.04133	3.41194	1.62422
H	0.42630	3.29665	0.94065
C	1.31778	4.56081	4.05945
H	1.74977	3.24963	5.73448
H	0.11186	3.11671	5.12144
H	0.73837	5.43145	2.16074
H	-0.51613	4.46005	2.90686
H	0.95541	5.38414	4.67157
H	2.37479	4.73704	3.86867
P	-0.96595	-2.22306	-0.44766
C	-2.60890	-2.38317	0.43878
H	-3.33165	-3.10364	0.02945
H	-2.42368	-2.64654	1.49368
C	-4.59631	-0.47661	-0.84517
C	-5.50662	-1.73089	-0.86590
C	-5.45541	0.78597	-0.60405
H	-4.15594	-0.38461	-1.83575
C	-6.62182	-1.58840	-1.92946
H	-5.96200	-1.88825	0.10826
H	-4.93156	-2.62041	-1.10427
C	-6.54998	0.93553	-1.68384
H	-5.93999	0.72591	0.36573
H	-4.82287	1.66742	-0.60006
C	-7.45831	-0.31003	-1.72019
H	-7.26721	-2.46390	-1.89246

H	-6.16811	-1.56139	-2.91838
H	-7.14543	1.82248	-1.47724
H	-6.08502	1.07355	-2.65780
H	-8.19048	-0.21480	-2.51932
H	-8.00593	-0.38518	-0.78272
C	-3.62918	-0.18030	2.14292
C	-3.80519	1.32580	2.44504
C	-4.86616	-0.97570	2.62788
H	-2.77976	-0.52131	2.72981
C	-4.02094	1.54968	3.95849
H	-4.66332	1.72138	1.91049
H	-2.92848	1.87535	2.10857
C	-5.08915	-0.75607	4.14242
H	-5.75657	-0.65388	2.09507
H	-4.73731	-2.03904	2.43607
C	-5.23150	0.74403	4.47766
H	-4.17461	2.60946	4.15073
H	-3.12727	1.24655	4.50057
H	-5.98073	-1.29281	4.46110
H	-4.24633	-1.17037	4.69249
H	-5.32883	0.87127	5.55392
H	-6.14176	1.12813	4.02120
C	-1.44263	-2.36640	-2.31572
C	-0.33184	-1.78831	-3.22294
C	-1.89018	-3.75353	-2.83623
H	-2.29439	-1.69436	-2.38768
C	-0.81554	-1.69120	-4.68720
H	0.55368	-2.41816	-3.17854
H	-0.05644	-0.79698	-2.86796
C	-2.39205	-3.64880	-4.29468
H	-1.05623	-4.44980	-2.80716
H	-2.68281	-4.16023	-2.21199
C	-1.30472	-3.05634	-5.21512
H	-0.00610	-1.32263	-5.31354
H	-1.62728	-0.96897	-4.74639
H	-2.68155	-4.63582	-4.64991
H	-3.27803	-3.01764	-4.32645
H	-1.69759	-2.94303	-6.22330
H	-0.46449	-3.74608	-5.26823
C	0.00931	-3.79178	0.13687
C	-0.79779	-5.11319	0.16193
C	1.34087	-3.97693	-0.62820
C	0.04333	-6.24691	0.79223
H	-1.08120	-5.40900	-0.84275
H	-1.71221	-4.98572	0.73608
C	2.17747	-5.12291	-0.01582
H	1.14013	-4.20566	-1.67217
H	1.91863	-3.05501	-0.59694
C	1.37585	-6.44026	0.03860
H	-0.52856	-7.17249	0.77597
H	0.24804	-6.00859	1.83418
H	3.08232	-5.26423	-0.60325
H	2.48325	-4.84499	0.99083
H	1.96712	-7.21140	0.52783
H	1.17029	-6.78040	-0.97454
H	0.25359	-3.55590	1.16920

9a$_c$

176

XYZ

Ru	0.88134	0.04860	-0.08496
C	-0.07950	2.71682	-0.99831
C	1.17575	2.16608	-0.93703
P	-1.02269	-0.88740	-1.24020
N	2.29649	2.98226	-0.56565
C	2.97180	3.74854	-1.51206
C	3.91200	4.70085	-0.75201
C	3.86501	4.23763	0.71979
C	2.58973	3.35749	0.82275
O	2.81016	3.67943	-2.73094
P	3.05225	-0.69171	0.12689
H	-0.87122	2.12669	-1.46778
H	1.74156	3.91986	1.26377
H	2.72947	2.44812	1.42951
H	4.91937	4.67803	-1.20031
H	3.52488	5.72867	-0.88752
H	3.83135	5.07594	1.43554
H	4.75573	3.63638	0.96377
H	1.42393	1.29414	-1.62587
C	-0.46720	4.11729	-0.60370
C	-0.86623	4.94569	-1.85415
H	-1.32307	4.09484	0.09861
H	0.36095	4.62875	-0.08296
C	-1.28586	6.38722	-1.50974
H	-0.01196	4.95610	-2.55788
H	-1.69797	4.43527	-2.38140
C	-1.67299	7.20812	-2.75137
H	-2.13596	6.36261	-0.79839
H	-0.45459	6.89159	-0.97713
H	-1.96284	8.23904	-2.47928
H	-0.83099	7.27162	-3.46493
H	-2.52656	6.74865	-3.28354
C	-2.76005	-0.08545	-1.27898
H	-2.61557	0.97976	-1.52214
H	-3.34780	-0.52512	-2.10012
P	-3.79389	-0.28825	0.30234
C	-5.55849	-0.48300	-0.45668
C	-6.63771	-0.55381	0.65203
C	-6.01970	0.48505	-1.57703
H	-5.50943	-1.47286	-0.90749
C	-8.00284	-0.99553	0.07778
H	-6.75431	0.42010	1.12174
H	-6.32948	-1.25332	1.42658
C	-7.37617	0.03126	-2.16446

H	-6.13114	1.49314	-1.19145
H	-5.28403	0.52456	-2.37612
C	-8.45694	-0.06598	-1.06722
H	-8.74955	-0.99768	0.86941
H	-7.92197	-2.01441	-0.29604
H	-7.69388	0.73339	-2.93283
H	-7.25701	-0.94015	-2.64053
H	-9.38661	-0.43489	-1.49567
H	-8.65396	0.92719	-0.66807
C	-3.64791	1.40386	1.20534
C	-4.39791	2.62299	0.61444
C	-4.05291	1.20636	2.68959
H	-2.58195	1.62815	1.18424
C	-4.16113	3.90137	1.45122
H	-5.46598	2.42545	0.59996
H	-4.08307	2.80111	-0.41167
C	-3.82821	2.48467	3.52728
H	-5.10253	0.92837	2.75103
H	-3.47993	0.38878	3.12069
C	-4.57516	3.69015	2.92161
H	-4.72734	4.72514	1.02103
H	-3.10945	4.17434	1.41328
H	-4.16479	2.31458	4.54804
H	-2.76416	2.70665	3.56761
H	-4.36108	4.58727	3.49884
H	-5.64819	3.51608	2.97461
C	-1.45971	-2.72906	-0.76440
C	-2.82970	-3.28891	-1.22876
C	-0.31167	-3.68517	-1.16925
H	-1.49807	-2.70148	0.31940
C	-3.04850	-4.73088	-0.71454
H	-2.91205	-3.29263	-2.30914
H	-3.62990	-2.66862	-0.84316
C	-0.54868	-5.12889	-0.67240
H	-0.20369	-3.70516	-2.24995
H	0.62091	-3.30703	-0.76129
C	-1.90893	-5.67122	-1.15383
H	-4.00076	-5.10238	-1.08793
H	-3.10783	-4.72159	0.37178
H	0.25479	-5.76950	-1.03042
H	-0.52105	-5.15122	0.41477
H	-2.07510	-6.66817	-0.75096
H	-1.90410	-5.75365	-2.23897
C	-0.64249	-0.93066	-3.17397
C	-1.56262	-1.81434	-4.05614
C	-0.60092	0.49894	-3.76736
H	0.36105	-1.34296	-3.22730
C	-1.10603	-1.81526	-5.53423
H	-2.58973	-1.46019	-4.00732
H	-1.55238	-2.84076	-3.70891
C	-0.13651	0.49985	-5.24061
H	-1.58879	0.95083	-3.71393
H	0.07755	1.12115	-3.19667
C	-1.04167	-0.38963	-6.11405
H	-1.79133	-2.42540	-6.11954
H	-0.12214	-2.27525	-5.60277
H	-0.14069	1.52056	-5.61743
H	0.88788	0.13712	-5.29504
H	-0.66402	-0.42063	-7.13392
H	-2.04359	0.03392	-6.14874
P	1.04741	-1.08851	1.86014
C	2.77082	-1.79063	1.64206
H	3.44768	-1.66231	2.50206
H	2.75764	-2.85666	1.35675
C	4.76603	0.20982	0.49694
C	5.80066	-0.61710	1.30213
C	5.41777	0.76533	-0.79251
H	4.48242	1.05753	1.11644
C	7.07082	0.21222	1.60462
H	6.08897	-1.50711	0.75150
H	5.37524	-0.94436	2.24608
C	6.69649	1.58200	-0.49516
H	5.69052	-0.05581	-1.44800
H	4.70650	1.38859	-1.32946
C	7.71459	0.75116	0.31146
H	7.78524	-0.40626	2.14451
H	6.81013	1.04757	2.25181
H	7.14295	1.90658	-1.43295
H	6.44051	2.47568	0.06828
H	8.58166	1.36149	0.55568
H	8.06266	-0.08347	-0.29397
C	3.45674	-2.03915	-1.27106
C	3.39443	-1.43719	-2.68935
C	4.71491	-2.93411	-1.14448
H	2.59313	-2.68894	-1.16482
C	3.35497	-2.54996	-3.76077
H	4.25972	-0.80830	-2.87804
H	2.50854	-0.81042	-2.76926
C	4.69329	-4.06105	-2.20550
H	5.61787	-2.34630	-1.28660
H	4.76991	-3.38269	-0.15440
C	4.57710	-3.48548	-3.63331
H	3.33525	-2.10711	-4.75485
H	2.44328	-3.13282	-3.64532
H	5.59727	-4.66200	-2.12388
H	3.84784	-4.71889	-2.01305
H	4.49984	-4.29798	-4.35316
H	5.48141	-2.92766	-3.86918
C	1.33942	0.24175	3.28373
C	0.17470	1.25006	3.36582
C	1.70078	-0.24748	4.70828
H	2.20317	0.77683	2.89597
C	0.53540	2.44678	4.27509
H	-0.71579	0.76514	3.75854

H	-0.05765	1.61021	2.36497
C	2.08220	0.94200	5.62073
H	0.85408	-0.76229	5.15456
H	2.53132	-0.94927	4.67507
C	0.94058	1.97771	5.68870
H	-0.31134	3.12682	4.34275
H	1.36071	2.99934	3.83133
H	2.31087	0.57829	6.62075
H	2.98084	1.41943	5.23475
H	1.25064	2.83176	6.28745
H	0.07910	1.53054	6.18105
C	0.04972	-2.57482	2.61848
C	0.80069	-3.43468	3.66611
C	-1.32647	-2.14101	3.17607
C	-0.04305	-4.66472	4.07362
H	1.01515	-2.85345	4.55669
H	1.75126	-3.77596	3.26448
C	-2.17426	-3.36206	3.59936
H	-1.18703	-1.49824	4.04157
H	-1.87198	-1.56785	2.42910
C	-1.42231	-4.24120	4.62025
H	0.49228	-5.23851	4.82757
H	-0.18045	-5.31185	3.20954
H	-3.11511	-3.02134	4.02690
H	-2.41348	-3.95681	2.72043
H	-2.01407	-5.12368	4.85464
H	-1.28703	-3.68432	5.54549
H	-0.12538	-3.21743	1.76140
H	-2.59969	2.99982	-0.44050
H	-0.85575	3.30052	-0.35165
C	-2.29862	5.75940	-0.47303
H	-1.08276	5.07794	-2.14814
H	-2.80842	4.71126	-2.31013
C	-2.54682	7.17665	-1.01632
H	-3.18975	5.41427	0.08875
H	-1.46633	5.78342	0.25814
H	-2.77794	7.88625	-0.20153
H	-1.65962	7.55989	-1.55383
H	-3.39624	7.19051	-1.72439
C	-2.57013	-0.90897	-1.11389
H	-2.44451	-0.15103	-1.89933
H	-3.03463	-1.78922	-1.58537
P	-3.73652	-0.05968	0.11749
C	-4.80882	-1.47750	0.86141
C	-5.55273	-0.97178	2.12403
C	-5.80374	-2.22857	-0.05671
H	-4.08078	-2.20531	1.20645
C	-6.23796	-2.13372	2.87904
H	-6.30607	-0.23858	1.84745
H	-4.85307	-0.47643	2.79304
C	-6.47217	-3.40624	0.68928
H	-6.58404	-1.55004	-0.38938
H	-5.29869	-2.60547	-0.94339
C	-7.19837	-2.91670	1.95961
H	-6.78461	-1.74168	3.73447
H	-5.47726	-2.81078	3.26307
H	-7.18046	-3.90178	0.02818
H	-5.71635	-4.13912	0.96412
H	-7.61611	-3.76495	2.49810
H	-8.02740	-2.27248	1.67313
C	-4.81390	0.95060	-1.12343
C	-5.33309	0.26196	-2.41168
C	-5.98238	1.66448	-0.40057
H	-4.12189	1.72259	-1.44889
C	-5.99946	1.29783	-3.34624
H	-6.06032	-0.50775	-2.17375
H	-4.51464	-0.21444	-2.94449
C	-6.66140	2.69766	-1.32764
H	-6.72700	0.93779	-0.08375
H	-5.61706	2.16737	0.49271
C	-7.15075	2.03908	-2.63450
H	-6.37870	0.79605	-4.23436
H	-5.25261	2.01870	-3.67274
H	-7.50003	3.15927	-0.80982
H	-5.95056	3.48639	-1.56586
H	-7.56744	2.79629	-3.29555
H	-7.94711	1.33318	-2.40562
C	-1.20806	-2.64876	0.72183
C	-2.04811	-3.87261	0.27332
C	0.02116	-3.15295	1.50393

$[5_c\text{-}9b_c]^{‡}$

176

XYZ

Ru	1.02548	0.31364	-0.70556
C	-1.45381	2.32714	-2.15666
C	-0.46652	1.43109	-2.30647
P	-0.77256	-1.32807	-0.64766
N	2.47769	1.51822	-1.77860
C	2.87244	0.81809	-2.88125
C	3.52783	1.75762	-3.91023
C	3.74021	3.06379	-3.12239
C	2.71482	2.95910	-1.95422
O	2.67588	-0.41157	-3.03921
P	3.00257	-0.82038	0.36263
H	1.76852	3.48660	-2.20269
H	3.11127	3.41371	-1.03063
H	4.44839	1.31465	-4.32614
H	2.81895	1.88455	-4.75166
H	3.58795	3.97677	-3.72363
H	4.76638	3.10243	-2.71417
H	-2.21009	2.37911	-2.96848
C	-1.71753	3.31455	-1.03625
C	-1.97277	4.73952	-1.58079

H	-1.81444	-2.06612	1.41274
C	-2.48420	-4.72024	1.49130
H	-1.46051	-4.50579	-0.38109
H	-2.92828	-3.56266	-0.28000
C	-0.38357	-4.01433	2.72141
H	0.65246	-3.74372	0.84549
H	0.60217	-2.30690	1.85139
C	-1.26291	-5.20708	2.29673
H	-3.06218	-5.57446	1.14481
H	-3.12949	-4.13112	2.13902
H	0.51171	-4.37313	3.22544
H	-0.93100	-3.39666	3.43075
H	-1.59212	-5.75563	3.17665
H	-0.67567	-5.89001	1.68597
C	-0.11415	-2.09959	-2.32024
C	-0.23371	-3.62684	-2.53215
C	-0.70681	-1.34231	-3.53344
H	0.95028	-1.87086	-2.29241
C	0.50939	-4.03809	-3.82632
H	-1.27911	-3.91588	-2.61304
H	0.19979	-4.17210	-1.69721
C	0.06513	-1.73214	-4.80988
H	-1.74965	-1.62001	-3.65926
H	-0.65025	-0.24721	-3.34394
C	0.00147	-3.25565	-5.05567
H	0.38516	-5.10755	-3.98724
H	1.57322	-3.84925	-3.69653
H	-0.35717	-1.20723	-5.66414
H	1.10314	-1.42017	-4.71004
H	0.59809	-3.52244	-5.92575
H	-1.02658	-3.54597	-5.26503
P	1.15023	1.08884	1.43387
C	2.46622	-0.13104	2.03963
H	3.24797	0.30181	2.68294
H	2.00195	-0.95367	2.60257
C	4.64400	0.11673	-0.08606
C	5.56007	0.53094	1.09245
C	5.45348	-0.64743	-1.16263
H	4.26388	1.02140	-0.55126
C	6.74450	1.38272	0.57859
H	5.95228	-0.34077	1.60727
H	5.00748	1.11753	1.82054
C	6.63858	0.20460	-1.66844
H	5.84792	-1.57278	-0.75327
H	4.80328	-0.89962	-1.99768
C	7.55232	0.63799	-0.50377
H	7.39207	1.63854	1.41496
H	6.36219	2.31403	0.16531
H	7.21103	-0.36892	-2.39449
H	6.25837	1.08754	-2.17673
H	8.34746	1.28071	-0.87561
H	8.02134	-0.24026	-0.06408

C	3.47788	-2.64421	0.84293
C	3.56906	-3.57344	-0.39211
C	4.72844	-2.80275	1.74016
H	2.63162	-2.97628	1.43414
C	3.74855	-5.04645	0.04074
H	4.40632	-3.28472	-1.02077
H	2.66843	-3.47959	-0.99408
C	4.88884	-4.27607	2.18116
H	5.62368	-2.50356	1.20419
H	4.64327	-2.17109	2.62177
C	4.97497	-5.21756	0.96154
H	3.85944	-5.67196	-0.84252
H	2.85566	-5.38011	0.56599
H	5.78573	-4.37517	2.78925
H	4.04133	-4.56208	2.80117
H	5.04233	-6.25004	1.29780
H	5.88108	-4.99720	0.40056
C	2.07049	2.75355	1.85850
C	1.39768	4.01262	1.26357
C	2.36590	2.98334	3.36195
H	3.02400	2.62412	1.34946
C	2.30112	5.25304	1.44839
H	0.44654	4.19727	1.75327
H	1.19894	3.86466	0.20717
C	3.28193	4.21431	3.55186
H	1.43854	3.15554	3.90102
H	2.84377	2.11337	3.80484
C	2.65157	5.47952	2.93382
H	1.79414	6.12975	1.05102
H	3.21797	5.12059	0.87767
H	3.46180	4.36998	4.61376
H	4.24500	4.02223	3.08271
H	3.33929	6.31716	3.02878
H	1.74784	5.73540	3.48354
C	-0.36272	0.87739	2.63949
C	-0.02465	0.40280	4.07589
C	-1.24734	2.14571	2.69629
C	-1.31675	0.14167	4.88422
H	0.56900	1.15227	4.59162
H	0.55849	-0.51323	4.05267
C	-2.53808	1.88864	3.50357
H	-0.70100	2.95781	3.16661
H	-1.51197	2.45949	1.69088
C	-2.22018	1.39071	4.92811
H	-1.05251	-0.16182	5.89529
H	-1.86299	-0.68246	4.42943
H	-3.11817	2.80755	3.55390
H	-3.14132	1.14987	2.98160
H	-3.14325	1.15827	5.45491
H	-1.71710	2.17964	5.48396
H	-0.94982	0.09465	2.16487
H	0.64070	1.43200	-2.29826

[6_c-$7b_c$]‡

176

XYZ

Ru	-0.09809	0.93724	-0.38686
C	-2.63225	2.44606	0.79500
C	-1.72217	1.68850	0.06654
P	-0.76081	-1.34131	-0.47308
N	0.44680	2.79995	-1.26636
C	1.29892	2.38675	-2.20479
C	1.54148	3.45727	-3.27273
C	0.86628	4.69957	-2.64639
C	-0.13965	4.09229	-1.62142
O	1.74418	1.18894	-2.18841
P	3.60608	-0.94783	-0.38531
H	-2.47071	2.60569	1.88025
H	-1.14363	3.94401	-2.07659
H	-0.27927	4.73827	-0.73669
H	2.61464	3.58591	-3.49608
H	1.04969	3.13921	-4.21359
H	0.36883	5.35178	-3.38512
H	1.61826	5.30896	-2.11272
C	-3.84701	3.15168	0.21647
C	-3.71584	4.69179	0.29210
H	-3.98028	2.84232	-0.83769
H	-4.75959	2.84641	0.77299
C	-4.96489	5.42553	-0.23279
H	-3.52604	4.99418	1.34214
H	-2.82608	5.00559	-0.28706
C	-4.83708	6.95613	-0.15733
H	-5.15334	5.11991	-1.28138
H	-5.85144	5.09748	0.34649
H	-5.74518	7.45620	-0.53921
H	-4.68113	7.29358	0.88415
H	-3.97946	7.31560	-0.75546
C	-2.54496	-1.62821	-1.08890
H	-2.50847	-1.20254	-2.10502
H	-2.78303	-2.69718	-1.19793
P	-3.94595	-0.66150	-0.28735
C	-4.70121	-1.79673	1.06122
C	-5.58181	-0.95689	2.02089
C	-5.47096	-3.06355	0.61474
H	-3.83768	-2.11903	1.63845
C	-6.02516	-1.79174	3.24349
H	-6.46515	-0.59591	1.49997
H	-5.02777	-0.08457	2.36117
C	-5.90103	-3.90843	1.83575
H	-6.36188	-2.77724	0.06180
H	-4.85812	-3.67195	-0.04709
C	-6.76099	-3.07763	2.81133
H	-6.67361	-1.19199	3.87896
H	-5.14951	-2.05874	3.83207
H	-6.46153	-4.77806	1.49832
H	-5.01564	-4.27227	2.35351
H	-7.00640	-3.67469	3.68719
H	-7.69788	-2.81086	2.32601
C	-5.13388	-0.44743	-1.78255
C	-5.42388	-1.68537	-2.66983
C	-6.46521	0.21961	-1.35969
H	-4.59558	0.26999	-2.40079
C	-6.25516	-1.28644	-3.91084
H	-5.97341	-2.43465	-2.10724
H	-4.49522	-2.14361	-3.00007
C	-7.30291	0.62149	-2.59425
H	-7.04770	-0.46819	-0.75107
H	-6.26492	1.10195	-0.75582
C	-7.57319	-0.59362	-3.50610
H	-6.47133	-2.17274	-4.50417
H	-5.66994	-0.61248	-4.53347
H	-8.24600	1.05632	-2.26929
H	-6.76895	1.38434	-3.15741
H	-8.11091	-0.27497	-4.39667
H	-8.20698	-1.30505	-2.97999
C	-0.59734	-2.61106	1.00398
C	-1.51247	-3.86112	0.98372
C	0.87027	-3.06531	1.19365
H	-0.87725	-2.02339	1.87629
C	-1.35664	-4.67056	2.29248
H	-1.26509	-4.50420	0.14530
H	-2.55229	-3.57866	0.87516
C	1.04532	-3.87321	2.49898
H	1.17620	-3.68745	0.35723
H	1.53092	-2.20545	1.20620
C	0.10727	-5.09710	2.52395
H	-1.99511	-5.55071	2.24937
H	-1.69349	-4.06502	3.13177
H	2.07982	-4.19832	2.58903
H	0.82836	-3.23514	3.35325
H	0.19560	-5.61295	3.47778
H	0.40629	-5.79637	1.74554
C	0.22942	-2.13193	-1.95247
C	-0.14196	-3.59381	-2.30966
C	0.12044	-1.24053	-3.21585
H	1.26761	-2.11009	-1.62582
C	0.73497	-4.13495	-3.46200
H	-1.18569	-3.65221	-2.60917
H	-0.01242	-4.24397	-1.45133
C	0.98726	-1.78710	-4.37178
H	-0.91286	-1.18601	-3.54968
H	0.45964	-0.23411	-2.98177
C	0.62807	-3.24601	-4.71603
H	0.42967	-5.15260	-3.69806
H	1.77225	-4.17369	-3.13505
H	0.85411	-1.15599	-5.24798

H	2.03584	-1.731320	-4.08807
H	1.29148	-3.62070	-5.49278
H	-0.38760	-3.28974	-5.10463
P	1.57485	1.03659	1.23964
C	3.23529	0.07185	1.17062
H	4.07439	0.71664	1.46005
H	3.16205	-0.69268	1.96169
C	4.50681	0.24052	-1.59073
C	5.51194	1.27381	-1.02503
C	5.16458	-0.55518	-2.74427
H	3.66344	0.78983	-2.00981
C	6.01222	2.22198	-2.13974
H	6.36938	0.77829	-0.57813
H	5.04259	1.87210	-0.24968
C	5.65913	0.38810	-3.86329
H	6.01259	-1.12697	-2.37340
H	4.45166	-1.26347	-3.16083
C	6.64405	1.43812	-3.30871
H	6.73967	2.91865	-1.72717
H	5.17501	2.80841	-2.51271
H	6.14168	-0.19273	-4.64708
H	4.80513	0.89383	-4.30928
H	6.93684	2.12628	-4.09916
H	7.54653	0.93825	-2.96168
C	4.85656	-2.18438	0.41366
C	5.02747	-3.44193	-0.47556
C	6.24600	-1.65635	0.84635
H	4.34297	-2.51025	1.31677
C	5.83573	-4.54066	0.25147
H	5.53898	-3.18193	-1.39902
H	4.05068	-3.83568	-0.74892
C	7.05015	-2.74874	1.58838
H	6.81332	-1.34301	-0.02620
H	6.13828	-0.78967	1.49494
C	7.20893	-4.01262	0.71730
H	5.97423	-5.39334	-0.41039
H	5.27297	-4.88832	1.11578
H	8.03091	-2.36182	1.85830
H	6.53772	-3.00937	2.51252
H	7.73145	-4.78514	1.27791
H	7.81711	-3.77448	-0.15326
C	2.12895	2.89916	1.41687
C	0.98612	3.75278	2.01790
C	3.45424	3.20732	2.16037
H	2.25090	3.20540	0.38132
C	1.31228	5.26089	1.94324
H	0.83508	3.48682	3.06071
H	0.06071	3.55173	1.48518
C	3.78415	4.71683	2.08508
H	3.38726	2.91617	3.20412
H	4.28369	2.66125	1.72390
C	2.64134	5.57910	2.65748

H	0.50274	5.83008	2.39572
H	1.38294	5.56515	0.90151
H	4.70498	4.91049	2.63192
H	3.95814	4.99341	1.04702
H	2.88260	6.63399	2.54394
H	2.53776	5.38084	3.72261
C	1.01835	0.38666	2.99155
C	1.94552	0.75119	4.17724
C	-0.44211	0.73924	3.35694
C	1.52067	-0.00968	5.45460
H	1.88981	1.81740	4.37735
H	2.98131	0.51313	3.94917
C	-0.88675	-0.01469	4.63124
H	-0.53661	1.80679	3.53082
H	-1.09913	0.49126	2.52834
C	0.05101	0.28733	5.81917
H	2.17024	0.27479	6.28006
H	1.64597	-1.07913	5.29618
H	-1.90572	0.26988	4.88589
H	-0.88753	-1.08576	4.43805
H	-0.24220	-0.30496	6.68359
H	-0.04490	1.33570	6.09487
H	1.06456	-0.69243	2.86918
H	-2.84166	1.05115	0.27778

$7b_c$

176

XYZ

Ru	-0.20334	1.04362	-0.12565
C	-2.55317	2.36027	1.51032
C	-1.71502	1.64895	0.49648
P	-0.92319	-1.20085	-0.48473
N	0.39251	2.91217	-0.97978
C	1.33860	2.54866	-1.84610
C	1.70945	3.69006	-2.79893
C	1.00517	4.90169	-2.14736
C	-0.11608	4.24938	-1.28245
O	1.78327	1.35273	-1.86088
P	3.53247	-1.03409	-0.57470
H	-1.89119	3.06882	2.05227
H	-1.06836	4.18234	-1.85125
H	-0.33002	4.82473	-0.36355
H	2.80267	3.80008	-2.90396
H	1.30744	3.46083	-3.80590
H	0.60181	5.62520	-2.87707
H	1.71318	5.44341	-1.49416
C	-3.81530	3.10836	1.01299
C	-3.53729	4.30558	0.09160
H	-4.46647	2.38093	0.49599
H	-4.37054	3.45265	1.90911
C	-4.81635	5.05438	-0.32850
H	-2.84924	5.01472	0.59757

H	-3.00478	3.94912	-0.81129
C	-4.53936	6.24902	-1.25666
H	-5.50421	4.34598	-0.83270
H	-5.35036	5.40540	0.57783
H	-5.47344	6.76546	-1.54259
H	-3.88209	6.99155	-0.76686
H	-4.03733	5.92356	-2.18672
C	-2.71302	-1.35480	-1.08136
H	-2.67808	-0.82658	-2.04744
H	-2.97580	-2.40230	-1.29258
P	-4.07250	-0.43383	-0.14998
C	-4.83180	-1.74363	1.03496
C	-5.70679	-1.03508	2.09997
C	-5.60708	-2.94424	0.43933
H	-3.96812	-2.14003	1.56413
C	-6.14304	-2.01684	3.21103
H	-6.59386	-0.61179	1.63525
H	-5.15143	-0.21158	2.54371
C	-6.02889	-3.93794	1.54614
H	-6.50273	-2.59142	-0.06519
H	-5.00219	-3.46559	-0.29952
C	-6.88202	-3.23849	2.62521
H	-6.78735	-1.50290	3.92157
H	-5.26408	-2.35610	3.75588
H	-6.59180	-4.75868	1.10573
H	-5.14027	-4.36383	2.00808
H	-7.12192	-3.94195	3.41994
H	-7.82209	-2.91308	2.18379
C	-5.27378	-0.08246	-1.61586
C	-5.55335	-1.20927	-2.64448
C	-6.61570	0.50842	-1.11780
H	-4.75298	0.71154	-2.14970
C	-6.39319	-0.67710	-3.82873
H	-6.09243	-2.02721	-2.17552
H	-4.62231	-1.61490	-3.03106
C	-7.46352	1.04409	-2.29332
H	-7.18719	-0.25608	-0.59651
H	-6.43057	1.31448	-0.41147
C	-7.71992	-0.05627	-3.34406
H	-6.59838	-1.48887	-4.52402
H	-5.81923	0.07487	-4.36681
H	-8.41169	1.42348	-1.91744
H	-6.94228	1.87641	-2.76213
H	-8.26424	0.35885	-4.18984
H	-8.34254	-0.83434	-2.90636
C	-0.76660	-2.59041	0.87229
C	-1.67065	-3.83980	0.72621
C	0.70261	-3.04725	1.03709
H	-1.06110	-2.08155	1.78883
C	-1.52330	-4.76297	1.95822
H	-1.40538	-4.40144	-0.16388
H	-2.71136	-3.55568	0.62899
C	0.86947	-3.96825	2.26627
H	1.02383	-3.58717	0.15036
H	1.35316	-2.18532	1.13695
C	-0.05818	-5.19657	2.16903
H	-2.15259	-5.64101	1.82742
H	-1.87611	-4.23863	2.84427
H	1.90563	-4.29220	2.34047
H	0.63616	-3.41053	3.17106
H	0.02375	-5.79527	3.07376
H	0.25675	-5.82057	1.33489
C	0.05167	-1.82408	-2.04063
C	-0.32122	-3.24337	-2.53918
C	-0.08436	-0.80566	-3.20113
H	1.09463	-1.82987	-1.72877
C	0.52839	-3.65568	-3.76296
H	-1.37276	-3.27815	-2.81495
H	-0.16528	-3.97651	-1.75485
C	0.75801	-1.22376	-4.42627
H	-1.12431	-0.71834	-3.50565
H	0.25589	0.17114	-2.86763
C	0.39107	-2.63946	-4.91317
H	0.21866	-4.64359	-4.09843
H	1.57319	-3.72505	-3.46651
H	0.60436	-0.50452	-5.22805
H	1.81229	-1.19459	-4.16098
H	1.03594	-2.92810	-5.74058
H	-0.63356	-2.64457	-5.28005
P	1.74611	0.95506	1.32946
C	3.32904	-0.10197	1.06540
H	4.22724	0.46632	1.33615
H	3.25864	-0.91264	1.80881
C	4.51081	0.14339	-1.73052
C	5.65688	1.00561	-1.14607
C	5.01724	-0.62123	-2.97773
H	3.71893	0.81844	-2.05337
C	6.20421	1.98518	-2.21041
H	6.47329	0.38052	-0.79549
H	5.30255	1.58251	-0.29709
C	5.55955	0.35190	-4.04805
H	5.81310	-1.30884	-2.69964
H	4.21095	-1.21309	-3.40524
C	6.68314	1.23907	-3.47300
H	7.02601	2.56068	-1.78835
H	5.42122	2.68904	-2.48522
H	5.93404	-0.21146	-4.90061
H	4.74747	0.98267	-4.40400
H	7.01072	1.95578	-4.22337
H	7.54013	0.61721	-3.22084
C	4.67218	-2.45144	0.07533
C	4.68372	-3.64315	-0.91503
C	6.12193	-2.10466	0.49302
H	4.15523	-2.79616	0.96947

C	5.39270	-4.87403	-0.30566
H	5.19301	-3.36284	-1.83375
H	3.66353	-3.91130	-1.18168
C	6.82792	-3.32955	1.11887
H	6.69226	-1.78418	-0.37494
H	6.12643	-1.28506	1.20840
C	6.82683	-4.52921	0.14816
H	5.42140	-5.67999	-1.03634
H	4.82158	-5.23083	0.54939
H	7.85150	-3.06813	1.38032
H	6.31834	-3.60903	2.03906
H	7.28062	-5.39387	0.62823
H	7.43148	-4.28611	-0.72348
C	2.42733	2.77164	1.52730
C	1.39054	3.66588	2.24947
C	3.82420	2.97147	2.16928
H	2.49017	3.11444	0.49814
C	1.80118	5.15416	2.20287
H	1.30226	3.36425	3.28985
H	0.41673	3.54504	1.78376
C	4.23893	4.46074	2.11489
H	3.82280	2.64714	3.20507
H	4.57940	2.39293	1.64830
C	3.20248	5.36576	2.81059
H	1.06842	5.74881	2.74454
H	1.80040	5.49645	1.17054
H	5.21180	4.57909	2.58801
H	4.34350	4.76544	1.07539
H	3.49754	6.40832	2.71170
H	3.17581	5.13342	3.87351
C	1.32405	0.25355	3.09752
C	2.37398	0.52448	4.20318
C	-0.08006	0.64534	3.61199
C	2.03586	-0.27518	5.48250
H	2.38578	1.58181	4.45192
H	3.37163	0.25386	3.86694
C	-0.43476	-0.14124	4.89457
H	-0.11883	1.70894	3.82823
H	-0.81988	0.44548	2.84291
C	0.62313	0.06964	5.99799
H	2.77373	-0.05717	6.25206
H	2.09415	-1.34065	5.26825
H	-1.41134	0.17506	5.25536
H	-0.50292	-1.20193	4.66047
H	0.38490	-0.54619	6.86279
H	0.60328	1.10835	6.32200
H	1.30848	-0.82015	2.92641
H	-2.86354	1.60129	2.25717

8b$_c$

176

XYZ

Ru	-0.24706	0.58940	-0.67685
C	-2.59739	2.65186	-1.18669
C	-1.69949	1.54553	-0.74684
P	-1.09823	-1.00250	0.83433
N	0.13652	0.99818	-2.78837
C	0.94366	-0.03326	-2.99602
C	1.20782	-0.29180	-4.47757
C	0.61064	0.97839	-5.13198
C	-0.37031	1.53528	-4.05016
O	1.34166	-0.72408	-1.98586
P	3.46703	-1.28988	0.41167
H	-3.56301	2.21372	-1.50635
H	-1.40991	1.18535	-4.23117
H	-0.39782	2.64141	-4.04541
H	2.27927	-0.44821	-4.69171
H	0.67767	-1.21544	-4.78426
H	0.10325	0.77970	-6.09175
H	1.41081	1.71568	-5.32325
C	-2.89122	3.81628	-0.20456
C	-3.69393	3.40318	1.04082
H	-1.94447	4.30300	0.09084
H	-3.46702	4.57573	-0.77153
C	-4.11726	4.60176	1.91062
H	-4.59541	2.84121	0.72401
H	-3.09703	2.69279	1.64600
C	-4.90196	4.18772	3.16690
H	-3.21846	5.17778	2.20893
H	-4.73336	5.29388	1.30120
H	-5.19878	5.06651	3.76734
H	-5.82296	3.63726	2.89881
H	-4.29716	3.52686	3.81533
C	-2.77844	-1.72281	0.35231
H	-2.54986	-2.46674	-0.42048
H	-3.21714	-2.27682	1.19699
P	-4.07328	-0.49939	-0.27003
C	-5.63976	-1.32036	0.50635
C	-6.82628	-0.32454	0.52876
C	-6.11262	-2.68831	-0.04347
H	-5.34816	-1.47754	1.54344
C	-8.00832	-0.87653	1.35775
H	-7.16933	-0.12557	-0.48350
H	-6.50238	0.62431	0.95129
C	-7.28449	-3.25012	0.79420
H	-6.44524	-2.58115	-1.07268
H	-5.29311	-3.40408	-0.03811
C	-8.46430	-2.25625	0.83783
H	-8.84061	-0.17631	1.32188
H	-7.70521	-0.96774	2.39907
H	-7.61582	-4.19716	0.37247
H	-6.94154	-3.44777	1.80804
H	-9.25497	-2.64859	1.47423
H	-8.87710	-2.14535	-0.16307

C	-4.08150	-0.73229	-2.18246
C	-3.91477	-2.15021	-2.78628
C	-5.32618	-0.04886	-2.80224
H	-3.20785	-0.15580	-2.47997
C	-3.80299	-2.08166	-4.32736
H	-4.75834	-2.78266	-2.52433
H	-3.01819	-2.62775	-2.40137
C	-5.21891	0.02806	-4.34139
H	-6.22364	-0.60705	-2.54547
H	-5.44175	0.95481	-2.39929
C	-5.02173	-1.37369	-4.95449
H	-3.71388	-3.08857	-4.73088
H	-2.89684	-1.54217	-4.59569
H	-6.11781	0.48653	-4.74908
H	-4.37737	0.66260	-4.61200
H	-4.88826	-1.29253	-6.03137
H	-5.91465	-1.97151	-4.78132
C	-1.32544	-0.37565	2.66528
C	-2.59001	-0.86852	3.40864
C	-0.06082	-0.64142	3.51947
H	-1.42098	0.70137	2.54709
C	-2.69905	-0.21494	4.80595
H	-2.57043	-1.94954	3.52419
H	-3.47878	-0.61391	2.83814
C	-0.17326	0.01887	4.91161
H	0.08331	-1.70884	3.65769
H	0.82034	-0.26312	3.00797
C	-1.43526	-0.46654	5.65351
H	-3.57361	-0.60816	5.32034
H	-2.84544	0.85693	4.68810
H	0.71393	-0.21480	5.49683
H	-0.21511	1.09991	4.79933
H	-1.52339	0.04693	6.60865
H	-1.34456	-1.53086	5.86203
C	-0.08598	-2.64945	0.93189
C	-0.72903	-3.75724	1.80695
C	0.18673	-3.20926	-0.48689
H	0.87435	-2.37936	1.36502
C	0.17461	-5.00853	1.88052
H	-1.68763	-4.04947	1.38615
H	-0.91580	-3.39934	2.81488
C	1.08467	-4.46464	-0.42290
H	-0.75117	-3.47915	-0.96582
H	0.66785	-2.45152	-1.10241
C	0.47286	-5.56125	0.47247
H	-0.31255	-5.77156	2.48462
H	1.10960	-4.75045	2.37393
H	1.23687	-4.85147	-1.42843
H	2.05889	-4.18508	-0.02925
H	1.15398	-6.40683	0.54351
H	-0.45087	-5.92304	0.02494
P	1.84235	1.57233	0.18749

C	3.32287	0.55312	0.83969
H	4.26810	1.09505	0.69797
H	3.15393	0.51790	1.93020
C	4.26581	-1.37651	-1.33101
C	5.39292	-0.38069	-1.69937
C	4.72332	-2.82127	-1.64848
H	3.40730	-1.15810	-1.96440
C	5.79017	-0.52036	-3.18726
H	6.27392	-0.55181	-1.08773
H	5.06841	0.63924	-1.52159
C	5.11981	-2.97087	-3.13427
H	5.57926	-3.08744	-1.03203
H	3.92553	-3.52342	-1.41955
C	6.22151	-1.96308	-3.52237
H	6.60089	0.16934	-3.41459
H	4.94346	-0.24421	-3.81249
H	5.46598	-3.98568	-3.32059
H	4.24335	-2.80555	-3.75761
H	6.44249	-2.04557	-4.58463
H	7.13496	-2.20078	-2.98041
C	4.75662	-1.74846	1.77572
C	4.75589	-3.27675	2.03197
C	6.21313	-1.24604	1.62427
H	4.34813	-1.28238	2.67111
C	5.59958	-3.64086	3.27476
H	5.15606	-3.80163	1.16776
H	3.73566	-3.62569	2.17646
C	7.05326	-1.59196	2.87526
H	6.67808	-1.70988	0.75821
H	6.23186	-0.16966	1.46857
C	7.04325	-3.11032	3.15018
H	5.61396	-4.72125	3.40442
H	5.13534	-3.21331	4.16146
H	8.07669	-1.24953	2.73471
H	6.64964	-1.06579	3.73825
H	7.59608	-3.32475	4.06254
H	7.54746	-3.62598	2.33511
C	2.62807	2.68515	-1.20125
C	1.65445	3.80708	-1.63750
C	4.02561	3.30905	-0.95482
H	2.72120	1.98919	-2.03251
C	2.15263	4.49859	-2.92617
H	1.57774	4.55201	-0.84959
H	0.66321	3.39595	-1.80524
C	4.54660	3.97765	-2.24834
H	3.97084	4.06364	-0.17736
H	4.74585	2.56469	-0.63267
C	3.57497	5.06820	-2.74483
H	1.46677	5.29853	-3.19756
H	2.15356	3.77907	-3.74194
H	5.52594	4.41320	-2.05996
H	4.66956	3.22128	-3.02106

H	3.93251	5.48082	-3.68595
H	3.54877	5.88112	-2.02167
C	1.59035	2.62839	1.79935
C	2.80526	3.45800	2.28290
C	0.33929	3.53362	1.75789
C	2.53585	4.03545	3.69133
H	2.98059	4.28660	1.60360
H	3.70874	2.85480	2.30898
C	0.05188	4.14057	3.15003
H	0.48829	4.34157	1.04737
H	-0.51708	2.95657	1.42141
C	1.26958	4.91690	3.69429
H	3.39460	4.62090	4.01383
H	2.41318	3.21893	4.40035
H	-0.80729	4.80451	3.08568
H	-0.20568	3.34409	3.84467
H	1.06450	5.26644	4.70405
H	1.44353	5.79457	3.07479
H	1.40505	1.86213	2.54860
H	-2.12017	3.06222	-2.10214

[8b$_c$-9b$_c$]‡

176

XYZ

Ru	-0.15300	0.69501	-0.70892
C	-2.65525	2.69786	-0.49508
C	-1.74949	1.62653	-0.56019
P	-1.00705	-1.17956	0.43514
N	0.40404	1.74895	-2.50902
C	1.17291	0.79425	-3.03284
C	1.43899	1.00981	-4.52330
C	0.90226	2.44386	-4.74185
C	-0.08277	2.65531	-3.55039
O	1.53409	-0.19887	-2.30902
P	3.46825	-1.37041	0.04125
H	-3.63361	2.54185	-0.98345
H	-1.12404	2.38892	-3.84565
H	-0.10550	3.70555	-3.20628
H	2.50489	0.88060	-4.77841
H	0.87230	0.25350	-5.10198
H	0.41175	2.58704	-5.71997
H	1.73002	3.17227	-4.67101
C	-2.57887	3.99412	0.29500
C	-3.59694	4.00588	1.46271
H	-1.55698	4.15492	0.67213
H	-2.81263	4.83733	-0.38639
C	-3.61003	5.34276	2.22953
H	-4.61148	3.79917	1.06628
H	-3.36439	3.17571	2.15681
C	-4.61648	5.35430	3.39235
H	-2.59289	5.55306	2.61601
H	-3.84630	6.16521	1.52472
H	-4.60622	6.32227	3.92480
H	-5.64694	5.18078	3.03083
H	-4.38501	4.56314	4.12918
C	-2.69997	-1.76241	-0.18989
H	-2.48907	-2.12801	-1.20282
H	-3.01204	-2.63891	0.39641
P	-4.17204	-0.57171	-0.22121
C	-5.55070	-1.78031	0.38758
C	-6.78812	-0.98397	0.87265
C	-6.00427	-2.93428	-0.53887
H	-5.10952	-2.23745	1.27129
C	-7.80131	-1.90303	1.59234
H	-7.28139	-0.50776	0.02860
H	-6.47499	-0.19270	1.55070
C	-6.99966	-3.86818	0.18750
H	-6.49383	-2.53197	-1.42170
H	-5.14706	-3.51413	-0.87476
C	-8.22814	-3.08357	0.69448
H	-8.67729	-1.32677	1.88364
H	-7.34963	-2.29041	2.50364
H	-7.32062	-4.65855	-0.48833
H	-6.50100	-4.34316	1.03027
H	-8.88931	-3.74809	1.24704
H	-8.78741	-2.70342	-0.15827
C	-4.40856	-0.19740	-2.09690
C	-4.30598	-1.33840	-3.14147
C	-5.72358	0.59020	-2.32166
H	-3.58215	0.48139	-2.29442
C	-4.36490	-0.76833	-4.57800
H	-5.11673	-2.04962	-3.01717
H	-3.37402	-1.88476	-3.02633
C	-5.79871	1.16314	-3.75459
H	-6.57866	-0.06314	-2.16271
H	-5.79970	1.40382	-1.60352
C	-5.65455	0.04646	-4.80937
H	-4.31142	-1.58441	-5.29610
H	-3.50004	-0.12958	-4.74625
H	-6.74529	1.68197	-3.89316
H	-5.00365	1.89346	-3.89158
H	-5.64462	0.47891	-5.80783
H	-6.51505	-0.61741	-4.75075
C	-1.17509	-1.14143	2.37987
C	-2.47433	-1.75449	2.95410
C	0.06076	-1.74863	3.09120
H	-1.19743	-0.07898	2.60933
C	-2.54958	-1.55800	4.48599
H	-2.52690	-2.81754	2.73156
H	-3.33861	-1.27794	2.50062
C	-0.01415	-1.54660	4.62137
H	0.12459	-2.81358	2.89215
H	0.97372	-1.29882	2.71092
C	-1.31352	-2.14627	5.19648

H	-3.45364	-2.02968	4.86580
H	-2.62009	-0.49476	4.70684
H	0.84907	-2.01464	5.09040
H	0.02554	-0.48461	4.85224
H	-1.37177	-1.94708	6.26449
H	-1.30090	-3.22667	5.06603
C	-0.02452	-2.80749	0.02166
C	-0.67193	-4.12464	0.52524
C	0.23477	-2.92906	-1.50108
H	0.94274	-2.69214	0.50479
C	0.24670	-5.33486	0.24356
H	-1.61685	-4.29089	0.01560
H	-0.88398	-4.08112	1.58901
C	1.14045	-4.14231	-1.81005
H	-0.70737	-3.05366	-2.02947
H	0.71193	-2.02704	-1.87793
C	0.54843	-5.45758	-1.26358
H	-0.23177	-6.24425	0.60218
H	1.17880	-5.21858	0.79295
H	1.28363	-4.22130	-2.88577
H	2.11739	-3.97675	-1.36220
H	1.24185	-6.27796	-1.43713
H	-0.37201	-5.69064	-1.79551
P	1.70806	1.39625	0.54602
C	3.19892	0.26773	0.95994
H	4.11781	0.86547	1.03332
H	2.98715	-0.08293	1.98466
C	4.38564	-0.94174	-1.58825
C	5.47518	0.15819	-1.57979
C	4.94392	-2.22521	-2.25039
H	3.56024	-0.58782	-2.20415
C	5.97844	0.44840	-3.01292
H	6.32163	-0.14010	-0.96797
H	5.07913	1.07692	-1.15972
C	5.44542	-1.94370	-3.68438
H	5.77115	-2.62066	-1.66495
H	4.17335	-2.99164	-2.28536
C	6.51214	-0.82921	-3.69317
H	6.76209	1.20298	-2.97868
H	5.16083	0.85526	-3.60460
H	5.85956	-2.85417	-4.11328
H	4.60426	-1.64333	-4.30590
H	6.80837	-0.60701	-4.71638
H	7.39906	-1.17469	-3.16528
C	4.68641	-2.15617	1.31929
C	4.75314	-3.69331	1.13420
C	6.11971	-1.58200	1.43234
H	4.19422	-1.97924	2.27437
C	5.52993	-4.36706	2.28816
H	5.23943	-3.93679	0.19265
H	3.74643	-4.10352	1.09179
C	6.89230	-2.24117	2.59816
H	6.66653	-1.76316	0.51063
H	6.08878	-0.50594	1.58832
C	6.94734	-3.77448	2.43345
H	5.59511	-5.43864	2.10996
H	4.98440	-4.22507	3.21917
H	7.90253	-1.83869	2.64158
H	6.40375	-1.99628	3.53937
H	7.44883	-4.22081	3.28980
H	7.53255	-4.01969	1.54922
C	2.52880	2.91884	-0.35213
C	1.54839	4.11379	-0.42954
C	3.90144	3.43410	0.15246
H	2.66900	2.54467	-1.36345
C	2.08125	5.20640	-1.38307
H	1.42138	4.54822	0.55877
H	0.57597	3.77258	-0.77172
C	4.45583	4.51102	-0.80935
H	3.80307	3.87314	1.13966
H	4.62760	2.63216	0.22670
C	3.47838	5.69595	-0.94949
H	1.38617	6.04342	-1.40188
H	2.13754	4.80605	-2.39283
H	5.41578	4.86536	-0.43878
H	4.62853	4.06584	-1.78726
H	3.86416	6.40948	-1.67470
H	3.40032	6.21191	0.00559
C	1.31539	1.88329	2.39222
C	2.45982	2.56133	3.18548
C	0.02582	2.71690	2.56090
C	2.09500	2.65609	4.68496
H	2.62257	3.56730	2.81045
H	3.39224	2.01389	3.07829
C	-0.36353	2.83703	4.05209
H	0.17824	3.71478	2.16151
H	-0.78554	2.26199	2.00006
C	0.78331	3.44273	4.88858
H	2.90465	3.14259	5.22535
H	1.98626	1.65393	5.09508
H	-1.25276	3.45671	4.14589
H	-0.61252	1.85280	4.44250
H	0.51268	3.44434	5.94246
H	0.93764	4.47794	4.59050
H	1.13495	0.91587	2.85271
H	-1.93196	2.22631	-1.64944

$9b_c$

176

XYZ

Ru	0.90622	0.20605	-0.74079
C	-1.41977	2.30689	-1.83199
C	-0.55669	1.25710	-1.81141
P	-0.70653	-1.54369	-0.47571

N	2.16039	1.50969	-2.00324
C	2.06709	0.65065	-3.01247
C	2.47638	1.23220	-4.35825
C	3.04228	2.61121	-3.93928
C	2.43877	2.85470	-2.51866
O	1.54424	-0.50492	-2.77781
P	3.03648	-0.68466	0.28079
H	1.49494	3.43437	-2.57762
H	3.13471	3.40757	-1.86362
H	3.19401	0.58902	-4.89697
H	1.57467	1.32035	-4.99497
H	2.77946	3.41876	-4.64339
H	4.14390	2.56769	-3.87653
H	-1.89027	2.51860	-2.81360
C	-1.88333	3.26984	-0.76582
C	-1.85644	4.74360	-1.23193
H	-2.92732	3.02542	-0.47525
H	-1.28932	3.14483	0.15087
C	-2.44277	5.72759	-0.20212
H	-0.81666	5.03918	-1.47830
H	-2.42645	4.83200	-2.18073
C	-2.42943	7.18890	-0.68190
H	-3.48271	5.42568	0.03643
H	-1.87791	5.64553	0.74805
H	-2.85753	7.87021	0.07587
H	-1.39930	7.53053	-0.89586
H	-3.01746	7.30912	-1.61109
C	-2.47149	-1.20065	-1.08405
H	-2.29582	-0.68072	-2.03780
H	-2.97579	-2.15328	-1.30940
P	-3.59240	0.02767	-0.16498
C	-4.76389	-1.07211	0.90007
C	-5.55314	-0.18888	1.89993
C	-5.74096	-2.04610	0.19763
H	-4.08258	-1.67194	1.49657
C	-6.30460	-1.05236	2.93863
H	-6.27435	0.42678	1.36910
H	-4.87389	0.48554	2.41575
C	-6.48219	-2.92891	1.22825
H	-6.48111	-1.48424	-0.36523
H	-5.21191	-2.68413	-0.50689
C	-7.24673	-2.06403	2.25255
H	-6.87724	-0.40981	3.60461
H	-5.58236	-1.59106	3.54916
H	-7.17727	-3.58863	0.71241
H	-5.76408	-3.55768	1.75073
H	-7.71310	-2.70186	3.00074
H	-8.04285	-1.52484	1.74275
C	-4.57703	0.70866	-1.67815
C	-5.10117	-0.28194	-2.74993
C	-5.72909	1.64329	-1.23463
H	-3.82944	1.32960	-2.16522
C	-5.67242	0.48635	-3.96436
H	-5.88359	-0.91407	-2.34127
H	-4.30318	-0.93414	-3.09369
C	-6.31054	2.41887	-2.43798
H	-6.52711	1.06223	-0.77804
H	-5.37268	2.35055	-0.48885
C	-6.79673	1.45446	-3.54010
H	-6.05401	-0.22152	-4.69782
H	-4.87251	1.04983	-4.44062
H	-7.13596	3.04606	-2.10656
H	-5.54423	3.07554	-2.84493
H	-7.14119	2.02148	-4.40261
H	-7.64400	0.88101	-3.16848
C	-1.14732	-2.44804	1.19655
C	-2.14330	-3.63581	1.14457
C	0.10046	-2.89173	1.99101
H	-1.62832	-1.65236	1.76267
C	-2.54969	-4.06908	2.57222
H	-1.68711	-4.48893	0.65800
H	-3.03258	-3.37983	0.57971
C	-0.26412	-3.36173	3.41782
H	0.60173	-3.70263	1.46835
H	0.80057	-2.06851	2.06114
C	-1.31318	-4.49151	3.39147
H	-3.25204	-4.89797	2.51218
H	-3.05525	-3.24878	3.07737
H	0.63424	-3.70178	3.92938
H	-0.65941	-2.51918	3.98124
H	-1.60780	-4.74511	4.40763
H	-0.87409	-5.38240	2.94668
C	-0.11049	-2.84260	-1.82139
C	-0.34241	-4.34738	-1.54101
C	-0.66151	-2.49480	-3.22686
H	0.96140	-2.67799	-1.85876
C	0.33408	-5.22091	-2.62402
H	-1.40686	-4.56833	-1.53034
H	0.06573	-4.62655	-0.57396
C	0.02285	-3.35403	-4.31231
H	-1.73315	-2.67180	-3.26633
H	-0.47678	-1.44719	-3.44317
C	-0.16439	-4.85980	-4.03744
H	0.13436	-6.27039	-2.41581
H	1.41190	-5.07955	-2.57603
H	-0.39232	-3.10280	-5.28620
H	1.08401	-3.11577	-4.33791
H	0.37486	-5.44570	-4.77895
H	-1.21877	-5.11554	-4.12369
P	1.15011	1.15724	1.38629
C	2.61064	0.09026	1.94810
H	3.41424	0.62148	2.48043
H	2.25927	-0.71491	2.61152
C	4.57352	0.28361	-0.39328

C	5.59532	0.80824	0.64604
C	5.29881	-0.50857	-1.50763
H	4.10624	1.14247	-0.86694
C	6.66977	1.68040	-0.04444
H	6.08637	-0.01493	1.15643
H	5.09783	1.40905	1.40200
C	6.37217	0.36361	-2.19625
H	5.78548	-1.38591	-1.09058
H	4.57892	-0.84993	-2.24844
C	7.38750	0.90961	-1.17140
H	7.39430	2.01334	0.69616
H	6.19700	2.56805	-0.46020
H	6.88851	-0.22591	-2.95112
H	5.88953	1.19521	-2.70455
H	8.10007	1.56418	-1.66884
H	7.94922	0.08208	-0.74215
C	3.61934	-2.44730	0.83762
C	3.71575	-3.45826	-0.33044
C	4.91188	-2.48915	1.68798
H	2.80954	-2.78164	1.47934
C	3.96797	-4.88737	0.20211
H	4.52410	-3.18569	-1.00231
H	2.79671	-3.44583	-0.90945
C	5.15660	-3.91555	2.23175
H	5.76768	-2.19692	1.08634
H	4.84115	-1.79360	2.52147
C	5.23297	-4.94441	1.08414
H	4.07120	-5.57383	-0.63572
H	3.10792	-5.21195	0.78466
H	6.08159	-3.93147	2.80467
H	4.34901	-4.18703	2.90891
H	5.35240	-5.94520	1.49384
H	6.10856	-4.73588	0.47239
C	2.04895	2.88474	1.41914
C	1.24669	4.00731	0.72660
C	2.54107	3.38950	2.79818
H	2.92575	2.68806	0.80521
C	2.10503	5.28260	0.56243
H	0.36814	4.25063	1.31800
H	0.90854	3.66851	-0.24727
C	3.43251	4.64143	2.63167
H	1.68865	3.66022	3.41476
H	3.09909	2.62081	3.32640
C	2.66544	5.77116	1.91429
H	1.50216	6.06634	0.10876
H	2.93177	5.07632	-0.11353
H	3.76535	4.98162	3.61037
H	4.31971	4.38138	2.05748
H	3.32364	6.62313	1.75710
H	1.84556	6.10489	2.54736
C	-0.06763	0.97608	2.89322
C	0.56822	1.04946	4.30535

C	-1.27671	1.93488	2.79609
C	-0.47735	0.73089	5.39820
H	0.96185	2.04195	4.49334
H	1.39517	0.34982	4.39107
C	-2.31331	1.64311	3.90489
H	-0.94233	2.96583	2.88573
H	-1.75621	1.82336	1.82698
C	-1.67966	1.69243	5.30995
H	-0.00958	0.80888	6.37764
H	-0.82440	-0.29398	5.28390
H	-3.12456	2.36494	3.83826
H	-2.74140	0.65624	3.74227
H	-2.42175	1.42714	6.06006
H	-1.34684	2.70624	5.52377
H	-0.44628	-0.03497	2.76695
H	-0.48033	0.78017	-2.81289

$[9b_c\text{-}10b_c]^{\ddagger}$

176

XYZ

Ru	-0.82227	-0.33802	-0.71189
C	0.94858	-1.66352	-3.00696
C	0.34573	-1.11245	-1.94546
P	0.86716	1.42797	-0.37561
N	-2.20990	-1.61749	-1.81112
C	-2.72547	-1.06100	-2.95443
C	-3.22605	-2.17893	-3.89959
C	-3.21074	-3.43452	-3.01747
C	-2.11615	-3.07918	-1.98135
O	-2.78463	0.15593	-3.23904
P	-3.04495	0.78963	0.14056
H	0.80390	-1.17271	-3.98284
H	-1.11421	-3.36142	-2.36734
H	-2.25874	-3.61330	-1.03129
H	-4.20319	-1.92021	-4.34025
H	-2.50083	-2.25676	-4.73429
H	-2.99090	-4.37160	-3.55975
H	-4.18559	-3.55854	-2.50915
H	-0.56206	0.10499	-2.33630
C	1.69436	-2.99079	-3.03622
C	1.85554	-3.69833	-1.67932
H	1.14945	-3.66746	-3.73068
H	2.69075	-2.85097	-3.50612
C	2.59195	-5.04611	-1.79358
H	2.39013	-3.03542	-0.97138
H	0.85284	-3.86154	-1.23975
C	2.73886	-5.76630	-0.44259
H	2.04988	-5.70202	-2.50495
H	3.59591	-4.88218	-2.23476
H	3.27338	-6.72741	-0.55107
H	3.30175	-5.14689	0.27998
H	1.75008	-5.98077	0.00411

C	2.62309	1.02439	-0.97484
H	2.42413	0.54076	-1.94499
H	3.17573	1.95434	-1.17598
P	3.68784	-0.29235	-0.11692
C	4.86202	0.67485	1.06363
C	5.60782	-0.31303	1.99661
C	5.88237	1.67506	0.46685
H	4.18294	1.24019	1.69636
C	6.35306	0.43766	3.12345
H	6.32817	-0.89461	1.42771
H	4.90324	-1.01472	2.43506
C	6.62012	2.44765	1.58468
H	6.62157	1.13873	-0.12146
H	5.39080	2.38350	-0.19615
C	7.33653	1.47930	2.54940
H	6.89293	-0.27511	3.74373
H	5.62850	0.94000	3.76156
H	7.34453	3.12816	1.14140
H	5.90593	3.05055	2.14164
H	7.79788	2.03905	3.36047
H	8.13283	0.96492	2.01485
C	4.68901	-0.88160	-1.65784
C	5.28393	0.17636	-2.62306
C	5.78476	-1.90335	-1.26669
H	3.93420	-1.42397	-2.22115
C	5.87210	-0.50365	-3.88107
H	6.07189	0.74188	-2.13572
H	4.52001	0.88379	-2.93340
C	6.38495	-2.58679	-2.51599
H	6.58556	-1.40257	-0.72780
H	5.36998	-2.66113	-0.60509
C	6.94661	-1.54571	-3.50657
H	6.30381	0.25172	-4.53488
H	5.07175	-0.99286	-4.43271
H	7.17409	-3.27272	-2.21413
H	5.61372	-3.17413	-3.00995
H	7.30557	-2.04542	-4.40399
H	7.79699	-1.03932	-3.05364
C	1.30106	2.31041	1.31074
C	2.40335	3.40120	1.29863
C	0.06271	2.88055	2.03950
H	1.67780	1.48241	1.90881
C	2.77992	3.80886	2.74201
H	2.05206	4.28651	0.78274
H	3.29139	3.05757	0.78138
C	0.40259	3.31636	3.48309
H	-0.32533	3.73834	1.49718
H	-0.72399	2.13751	2.07066
C	1.55116	4.34495	3.50454
H	3.55490	4.57197	2.71085
H	3.18828	2.95054	3.27159
H	-0.48314	3.73978	3.95261
H	0.69213	2.44273	4.06336
H	1.82217	4.57446	4.53290
H	1.21554	5.27043	3.04078
C	0.35156	2.76950	-1.71614
C	0.62868	4.25837	-1.39168
C	0.93432	2.45368	-3.11781
H	-0.72438	2.63994	-1.78403
C	0.00395	5.18489	-2.46179
H	1.70028	4.43856	-1.35958
H	0.21673	4.52856	-0.42451
C	0.29300	3.35668	-4.19430
H	2.00837	2.61952	-3.12086
H	0.75389	1.41588	-3.37756
C	0.51688	4.84847	-3.87589
H	0.23642	6.21978	-2.21800
H	-1.07845	5.08077	-2.43812
H	0.72237	3.11776	-5.16498
H	-0.77360	3.14859	-4.24886
H	0.00624	5.46841	-4.60976
H	1.57926	5.07665	-3.93812
P	-1.40445	-1.11394	1.50846
C	-2.73654	0.15940	1.90543
H	-3.60285	-0.18197	2.48779
H	-2.27082	0.97363	2.48115
C	-4.65496	-0.12229	-0.43042
C	-5.70199	-0.44242	0.66631
C	-5.33519	0.60280	-1.61683
H	-4.25768	-1.06258	-0.80483
C	-6.85364	-1.29500	0.08392
H	-6.11631	0.47155	1.08248
H	-5.25109	-0.99628	1.48281
C	-6.48043	-0.25378	-2.19936
H	-5.75142	1.54991	-1.28563
H	-4.59200	0.79276	-2.39011
C	-7.52462	-0.60138	-1.11873
H	-7.58974	-1.48395	0.86303
H	-6.45864	-2.25869	-0.23161
H	-6.95744	0.28721	-3.01394
H	-6.07000	-1.17172	-2.61379
H	-8.29167	-1.25058	-1.53579
H	-8.01599	0.30952	-0.78188
C	-3.45138	2.64828	0.48091
C	-3.56904	3.50415	-0.80349
C	-4.67197	2.90758	1.39755
H	-2.57127	2.99634	1.01522
C	-3.65775	5.00580	-0.44758
H	-4.45557	3.22569	-1.36366
H	-2.71456	3.33081	-1.45094
C	-4.76607	4.40638	1.76466
H	-5.58735	2.61562	0.89033
H	-4.59839	2.31862	2.30913
C	-4.84277	5.28774	0.50019

H	-3.76963	5.58888	-1.35931
H	-2.73169	5.32118	0.02929
H	-5.64391	4.57376	2.38559
H	-3.89378	4.68895	2.35105
H	-4.84810	6.33825	0.78338
H	-5.77619	5.08598	-0.02165
C	-2.32750	-2.81221	1.65847
C	-1.34285	-4.00468	1.59373
C	-3.28993	-3.00445	2.85870
H	-2.93319	-2.82733	0.75659
C	-2.10094	-5.34462	1.45839
H	-0.74686	-4.03648	2.50211
H	-0.66159	-3.88363	0.75511
C	-4.06998	-4.33099	2.70680
H	-2.73950	-3.03363	3.79288
H	-4.00064	-2.18710	2.92697
C	-3.11154	-5.53576	2.60833
H	-1.38581	-6.16460	1.45520
H	-2.62784	-5.36812	0.50717
H	-4.73585	-4.45638	3.55834
H	-4.68841	-4.28506	1.81256
H	-3.68210	-6.44861	2.45108
H	-2.57410	-5.64490	3.54847
C	-0.16267	-0.92832	2.97339
C	-0.71623	-1.26957	4.37772
C	1.16475	-1.68396	2.72490
C	0.31049	-0.90519	5.47311
H	-0.92116	-2.33352	4.44646
H	-1.64853	-0.73947	4.56078
C	2.19787	-1.35830	3.82811
H	0.98935	-2.75554	2.71631
H	1.57454	-1.41440	1.75326
C	1.64360	-1.64495	5.23951
H	-0.09257	-1.16656	6.44945
H	0.48308	0.16914	5.46849
H	3.09618	-1.94857	3.66336
H	2.48009	-0.30958	3.76076
H	2.37159	-1.34189	5.98909
H	1.48394	-2.71529	5.35416
H	0.06384	0.13451	2.96752

$10b_c$

176

XYZ

Ru	-0.81175	-0.12399	-0.80495
C	1.17886	-1.45134	-2.86627
C	0.46533	-0.91946	-1.87090
P	0.86175	1.55961	-0.21619
N	-2.12212	-1.20052	-2.17054
C	-2.63096	-0.49954	-3.22531
C	-3.11351	-1.47668	-4.32007
C	-3.13075	-2.83804	-3.60873
C	-2.06837	-2.63540	-2.49889
O	-2.70633	0.74838	-3.33602
P	-3.02692	0.75819	0.22345
H	1.18452	-0.85085	-3.79316
H	-1.05912	-2.91154	-2.86926
H	-2.26857	-3.25845	-1.61255
H	-4.07844	-1.15534	-4.74627
H	-2.36941	-1.45186	-5.14120
H	-2.90096	-3.69711	-4.26417
H	-4.12297	-3.01841	-3.15365
H	-1.14255	0.93165	-1.96566
C	1.82606	-2.82762	-2.97494
C	1.90121	-3.63380	-1.66883
H	1.25750	-3.41482	-3.72990
H	2.84449	-2.72757	-3.40292
C	2.57593	-5.00576	-1.85315
H	2.44357	-3.04383	-0.90522
H	0.87572	-3.77704	-1.27860
C	2.64537	-5.82247	-0.55172
H	2.02943	-5.58556	-2.62464
H	3.60078	-4.85956	-2.25088
H	3.13880	-6.79841	-0.71076
H	3.21258	-5.28139	0.22823
H	1.63402	-6.01985	-0.14990
C	2.61288	1.25023	-0.87394
H	2.42163	0.98685	-1.92547
H	3.18442	2.19049	-0.86874
P	3.63365	-0.24846	-0.31072
C	4.80442	0.39100	1.07872
C	5.49242	-0.81094	1.77495
C	5.87211	1.46125	0.74453
H	4.12695	0.82195	1.81144
C	6.23304	-0.36575	3.05634
H	6.20707	-1.27538	1.10072
H	4.75299	-1.56637	2.02846
C	6.60485	1.93068	2.02261
H	6.60865	1.04485	0.06311
H	5.42184	2.31983	0.25134
C	7.26427	0.74166	2.75283
H	6.73348	-1.22085	3.50649
H	5.50937	0.00495	3.77982
H	7.36219	2.66685	1.76020
H	5.89604	2.41678	2.68980
H	7.72274	1.08407	3.67834
H	8.05678	0.33306	2.12883
C	4.65036	-0.49606	-1.93206
C	5.29907	0.73608	-2.61460
C	5.70552	-1.61836	-1.77570
H	3.88942	-0.86370	-2.61566
C	5.88972	0.34717	-3.98972
H	6.09538	1.14282	-1.99923
H	4.56549	1.52420	-2.75984

C	6.31026	-2.01333	-3.14187
H	6.50993	-1.28494	-1.12416
H	5.25379	-2.49418	-1.31462
C	6.92331	-0.79065	-3.85598
H	6.35774	1.21807	-4.44465
H	5.08511	0.02792	-4.64920
H	7.07218	-2.77694	-2.99781
H	5.53188	-2.44369	-3.76862
H	7.28385	-1.08012	-4.84094
H	7.78019	-0.43356	-3.28766
C	1.28476	2.11160	1.60935
C	2.44953	3.11316	1.82051
C	0.04552	2.62778	2.37565
H	1.58621	1.17280	2.07110
C	2.77401	3.26022	3.32511
H	2.18481	4.09130	1.43854
H	3.34055	2.79355	1.29421
C	0.33840	2.81544	3.88187
H	-0.27994	3.57734	1.95842
H	-0.77398	1.92907	2.26124
C	1.54168	3.75185	4.11127
H	3.59456	3.96373	3.45009
H	3.10124	2.30279	3.72560
H	-0.54401	3.21735	4.37594
H	0.54849	1.84646	4.32993
H	1.77273	3.80222	5.17323
H	1.28431	4.75754	3.78480
C	0.39356	3.14212	-1.26850
C	0.90422	4.51964	-0.77984
C	0.78488	2.95257	-2.75596
H	-0.69001	3.15857	-1.22295
C	0.32972	5.66026	-1.65397
H	1.99058	4.55609	-0.81830
H	0.60127	4.70060	0.24633
C	0.18350	4.07498	-3.62861
H	1.86621	2.97611	-2.86182
H	0.42705	1.99106	-3.11206
C	0.65498	5.46229	-3.14779
H	0.73024	6.61148	-1.30845
H	-0.75001	5.69639	-1.52384
H	0.47935	3.92289	-4.66440
H	-0.90232	4.02052	-3.58825
H	0.17743	6.24476	-3.73383
H	1.72925	5.55174	-3.29789
P	-1.38765	-1.37679	1.34187
C	-2.71254	-0.13428	1.86104
H	-3.58723	-0.54019	2.38618
H	-2.24160	0.58551	2.54744
C	-4.59902	-0.08332	-0.52539
C	-5.65966	-0.59076	0.48364
C	-5.26704	0.80729	-1.60044
H	-4.17162	-0.94303	-1.03564
C	-6.78089	-1.36144	-0.25193
H	-6.10117	0.23962	1.02772
H	-5.21200	-1.25676	1.21402
C	-6.38147	0.03145	-2.33583
H	-5.71085	1.68284	-1.13545
H	-4.51139	1.12943	-2.31671
C	-7.44021	-0.49931	-1.34745
H	-7.52920	-1.68257	0.47020
H	-6.35942	-2.25689	-0.70451
H	-6.85319	0.68453	-3.06700
H	-5.94322	-0.80280	-2.87863
H	-8.18477	-1.08841	-1.87879
H	-7.95740	0.33885	-0.88411
C	-3.46981	2.53705	0.82678
C	-3.58588	3.57001	-0.32026
C	-4.70798	2.63685	1.75103
H	-2.60424	2.81728	1.42095
C	-3.69704	5.00030	0.25479
H	-4.46463	3.36787	-0.92341
H	-2.72458	3.50193	-0.97825
C	-4.83071	4.06330	2.33474
H	-5.61074	2.40977	1.19061
H	-4.63705	1.92010	2.56634
C	-4.89908	5.12373	1.21530
H	-3.80403	5.71195	-0.56118
H	-2.78140	5.25014	0.78749
H	-5.72117	4.12524	2.95716
H	-3.97264	4.26521	2.97314
H	-4.92221	6.11934	1.65348
H	-5.82204	4.99321	0.65371
C	-2.36278	-3.05034	1.25806
C	-1.41868	-4.25033	1.00707
C	-3.32267	-3.38754	2.42748
H	-2.97423	-2.91725	0.36874
C	-2.22573	-5.52533	0.67440
H	-0.81774	-4.43677	1.89384
H	-0.74023	-4.02678	0.18857
C	-4.15409	-4.64424	2.07982
H	-2.76511	-3.58521	3.33640
H	-3.99903	-2.56415	2.63390
C	-3.24233	-5.85403	1.78750
H	-1.54179	-6.36075	0.54028
H	-2.75263	-5.38097	-0.26642
H	-4.82039	-4.87482	2.90868
H	-4.77420	-4.43738	1.20983
H	-3.84778	-6.70888	1.49396
H	-2.70860	-6.12868	2.69540
C	-0.19466	-1.38011	2.85652
C	-0.78523	-1.89373	4.19092
C	1.13905	-2.10328	2.54905
C	0.21292	-1.66678	5.34822
H	-0.98644	-2.95827	4.12102

H	-1.72402	-1.39120	4.41378
C	2.14397	-1.91752	3.70935
H	0.96162	-3.16528	2.40282
H	1.57116	-1.71605	1.62807
C	1.55348	-2.37351	5.06025
H	-0.21448	-2.04564	6.27436
H	0.38262	-0.60001	5.47949
H	3.04757	-2.48408	3.49698
H	2.42559	-0.86862	3.77699
H	2.26104	-2.16443	5.85976
H	1.39357	-3.44974	5.03951
H	0.03023	-0.32429	2.98744

[$7a_c$-$10b_c$]‡

176

XYZ

Ru	0.70152	0.38284	-0.63760
C	-1.31245	2.80539	-0.86477
C	-0.50151	1.75520	-0.74226
P	-0.98096	-1.43418	-0.55549
N	2.03564	1.58547	-1.81027
C	2.54197	1.05808	-2.96440
C	3.10140	2.20140	-3.84542
C	3.18391	3.39605	-2.88273
C	2.07558	3.05497	-1.85624
O	2.57066	-0.14913	-3.29353
P	2.97631	-0.93141	-0.04764
H	-2.32935	2.56411	-1.20592
H	1.09789	3.46001	-2.19332
H	2.27716	3.47865	-0.85562
H	4.05388	1.91001	-4.31797
H	2.37259	2.38356	-4.66060
H	3.02915	4.37931	-3.36197
H	4.17014	3.41643	-2.38184
H	0.45355	-0.05283	-2.15565
C	-1.07396	4.27718	-0.56848
C	-1.55146	5.19203	-1.71865
H	-1.61084	4.56599	0.36097
H	-0.00155	4.46128	-0.38422
C	-1.36096	6.69064	-1.41823
H	-1.00606	4.92374	-2.64542
H	-2.62341	4.99155	-1.92373
C	-1.84479	7.60024	-2.56022
H	-1.90120	6.94788	-0.48479
H	-0.28917	6.88917	-1.21606
H	-1.69330	8.66789	-2.31906
H	-1.29975	7.38745	-3.49858
H	-2.92221	7.45131	-2.76068
C	-2.72035	-0.91900	-1.10855
H	-2.51465	-0.33545	-2.02062
H	-3.28562	-1.81116	-1.41983
P	-3.77394	0.28906	-0.08097
C	-4.93687	-0.79642	1.00140
C	-5.60730	0.09026	2.08194
C	-6.01295	-1.67724	0.32128
H	-4.25806	-1.45997	1.52972
C	-6.35383	-0.76850	3.12786
H	-6.31416	0.77444	1.61955
H	-4.85502	0.69528	2.58256
C	-6.74890	-2.55551	1.35934
H	-6.74687	-1.04628	-0.17215
H	-5.56761	-2.31504	-0.43918
C	-7.39689	-1.68888	2.45960
H	-6.84475	-0.12019	3.85099
H	-5.63455	-1.37681	3.67298
H	-7.51295	-3.14814	0.85996
H	-6.04424	-3.24915	1.81332
H	-7.86044	-2.32766	3.20865
H	-8.18392	-1.07985	2.01907
C	-4.78893	1.05411	-1.53633
C	-5.42658	0.11684	-2.59439
C	-5.85009	2.05432	-1.01573
H	-4.03476	1.63796	-2.05959
C	-6.01933	0.94018	-3.76142
H	-6.21978	-0.47866	-2.15378
H	-4.68557	-0.57119	-2.99219
C	-6.45546	2.87926	-2.17364
H	-6.65200	1.51886	-0.51261
H	-5.40079	2.72819	-0.28903
C	-7.06003	1.96184	-3.25712
H	-6.48176	0.26895	-4.48255
H	-5.21661	1.46628	-4.27437
H	-7.22208	3.54723	-1.78577
H	-5.67808	3.49790	-2.61762
H	-7.42190	2.56181	-4.08959
H	-7.91489	1.43025	-2.84317
C	-1.43152	-2.49981	1.01691
C	-2.54594	-3.56982	0.88578
C	-0.19421	-3.16160	1.66627
H	-1.79322	-1.74129	1.70980
C	-2.91879	-4.13787	2.27489
H	-2.20741	-4.39339	0.26909
H	-3.43245	-3.15959	0.41603
C	-0.53082	-3.75939	3.05148
H	0.18361	-3.95336	1.02487
H	0.59956	-2.43292	1.77833
C	-1.69098	-4.77135	2.96078
H	-3.70220	-4.88443	2.16162
H	-3.31418	-3.34263	2.90350
H	0.35235	-4.24410	3.46306
H	-0.80776	-2.95625	3.73132
H	-1.95820	-5.11652	3.95733
H	-1.36833	-5.63983	2.38991
C	-0.47376	-2.61453	-2.03709

C	-0.82006	-4.11838	-1.90713
C	-1.00690	-2.09298	-3.39576
H	0.60690	-2.51968	-2.06479
C	-0.21608	-4.92806	-3.07929
H	-1.89880	-4.25423	-1.90913
H	-0.43489	-4.52704	-0.97821
C	-0.38082	-2.88102	-4.56658
H	-2.08720	-2.20394	-3.44084
H	-0.76397	-1.04059	-3.50567
C	-0.68081	-4.38874	-4.44650
H	-0.50072	-5.97335	-2.97475
H	0.86913	-4.88009	-3.02499
H	-0.77469	-2.50037	-5.50657
H	0.69495	-2.71840	-4.57332
H	-0.18296	-4.93492	-5.24497
H	-1.75093	-4.55530	-4.55503
P	1.47832	0.86135	1.71248
C	2.74704	-0.52970	1.79264
H	3.65512	-0.33335	2.37738
H	2.25801	-1.39874	2.25852
C	4.58500	0.01319	-0.57053
C	5.66053	0.21501	0.52647
C	5.22882	-0.60779	-1.83402
H	4.19689	0.99063	-0.84654
C	6.81552	1.09889	-0.00003
H	6.06534	-0.74076	0.84788
H	5.23590	0.69883	1.39930
C	6.37407	0.28360	-2.36150
H	5.63974	-1.58505	-1.59803
H	4.46507	-0.72222	-2.60247
C	7.44773	0.51141	-1.27785
H	7.57134	1.20114	0.77638
H	6.43201	2.09456	-0.21423
H	6.82340	-0.18535	-3.23436
H	5.97091	1.24279	-2.67780
H	8.21676	1.18522	-1.64972
H	7.93016	-0.43475	-1.03977
C	3.35059	-2.82481	0.06422
C	3.42720	-3.53111	-1.31112
C	4.58393	-3.20771	0.91906
H	2.47326	-3.21460	0.57486
C	3.48817	-5.06469	-1.12760
H	4.31114	-3.21019	-1.85190
H	2.56768	-3.26670	-1.91966
C	4.65507	-4.74006	1.11172
H	5.49463	-2.87499	0.42860
H	4.54033	-2.72638	1.89339
C	4.68602	-5.47480	-0.24501
H	3.56828	-5.54349	-2.10127
H	2.56516	-5.41296	-0.66802
H	5.54236	-4.99218	1.68906
H	3.78995	-5.07097	1.68326
H	4.67416	-6.55033	-0.08112
H	5.61263	-5.23400	-0.76240
C	2.52129	2.45353	2.08011
C	1.61797	3.69607	2.26052
C	3.54508	2.39482	3.24221
H	3.08276	2.58398	1.15693
C	2.46332	4.98900	2.26885
H	1.07854	3.62432	3.20167
H	0.88375	3.74449	1.46212
C	4.41308	3.67489	3.24336
H	3.03461	2.32165	4.19668
H	4.19478	1.52985	3.15561
C	3.54349	4.94336	3.36991
H	1.81210	5.84637	2.42544
H	2.93885	5.11321	1.29809
H	5.12130	3.62854	4.06824
H	4.98883	3.71969	2.32098
H	4.17320	5.82829	3.30741
H	3.06356	4.95308	4.34671
C	0.30209	0.48999	3.19497
C	0.91488	0.61071	4.61021
C	-1.00937	1.30820	3.10682
C	-0.08812	0.11939	5.67841
H	1.15806	1.64773	4.82179
H	1.83410	0.03271	4.68012
C	-2.01701	0.84468	4.18344
H	-0.80011	2.36452	3.25136
H	-1.45388	1.19853	2.11948
C	-1.40927	0.91272	5.60053
H	0.35272	0.23030	6.66704
H	-0.28900	-0.93946	5.52800
H	-2.90713	1.46808	4.13592
H	-2.32417	-0.17796	3.97342
H	-2.11900	0.51918	6.32514
H	-1.21899	1.95186	5.86182
H	0.04233	-0.55491	3.04248

[10b$_c$-11b$_c$]‡

176

XYZ

Ru	0.80346	0.13641	-0.61099
C	-1.16581	2.40436	-1.82126
C	-0.41522	1.40351	-1.33695
P	-0.64026	-1.65104	-0.08976
N	1.29985	1.33723	-2.45891
C	0.96091	0.81678	-3.70717
C	1.25014	1.87629	-4.78882
C	2.21128	2.86283	-4.11068
C	1.90705	2.67405	-2.59712
O	0.47543	-0.29670	-3.94803
P	2.99764	-0.72336	0.14223
H	-0.91841	2.79511	-2.81980

H	1.20453	3.44824	-2.22769
H	2.82684	2.74321	-1.99232
H	1.64156	1.39993	-5.70242
H	0.28451	2.35145	-5.05608
H	2.07790	3.91030	-4.43260
H	3.25686	2.58040	-4.32648
H	0.66303	-0.83745	-1.88086
C	-2.29203	3.13671	-1.11114
C	-2.14393	4.67263	-1.20622
H	-3.26661	2.86173	-1.56234
H	-2.34665	2.81785	-0.05699
C	-3.29667	5.42987	-0.51978
H	-1.17988	4.98530	-0.76006
H	-2.09605	4.97018	-2.27461
C	-3.16148	6.95836	-0.62360
H	-4.25983	5.11167	-0.96719
H	-3.34310	5.13312	0.54720
H	-4.00048	7.47419	-0.12244
H	-2.22357	7.30968	-0.15432
H	-3.14805	7.28787	-1.67934
C	-2.37114	-1.49570	-0.83939
H	-2.12912	-1.24946	-1.88630
H	-2.87430	-2.47549	-0.83650
P	-3.51657	-0.07110	-0.35036
C	-4.69695	-0.78635	0.98971
C	-5.46893	0.36973	1.67566
C	-5.68398	-1.91458	0.60317
H	-4.02278	-1.18687	1.74149
C	-6.24786	-0.13148	2.91300
H	-6.17019	0.82086	0.97853
H	-4.77215	1.14803	1.97956
C	-6.45409	-2.42985	1.84092
H	-6.40548	-1.54395	-0.11990
H	-5.15567	-2.74439	0.13864
C	-7.20819	-1.28198	2.54465
H	-6.80992	0.69123	3.35069
H	-5.54262	-0.47960	3.66533
H	-7.15866	-3.20209	1.53816
H	-5.75443	-2.88379	2.53988
H	-7.69829	-1.65624	3.44121
H	-7.98506	-0.90409	1.88272
C	-4.47860	0.10714	-2.01590
C	-4.91723	-1.17577	-2.76946
C	-5.69254	1.05967	-1.87968
H	-3.74590	0.60673	-2.64788
C	-5.49615	-0.82341	-4.15942
H	-5.67254	-1.71241	-2.20268
H	-4.07475	-1.84757	-2.90567
C	-6.27537	1.42494	-3.26330
H	-6.47297	0.58429	-1.29030
H	-5.40249	1.96951	-1.35937
C	-6.68039	0.15926	-4.04713

H	-5.81976	-1.73367	-4.66058
H	-4.71390	-0.37660	-4.76990
H	-7.14003	2.07378	-3.13735
H	-5.53137	1.97941	-3.83184
H	-7.02816	0.43435	-5.04082
H	-7.50780	-0.33025	-3.53674
C	-1.06693	-2.14425	1.75345
C	-2.14179	-3.22992	2.01969
C	0.19334	-2.49903	2.57683
H	-1.45596	-1.20785	2.15079
C	-2.49830	-3.28589	3.52326
H	-1.77533	-4.20698	1.72931
H	-3.04067	-3.04290	1.44352
C	-0.11992	-2.58928	4.08846
H	0.59661	-3.45334	2.24792
H	0.96495	-1.75387	2.41671
C	-1.24911	-3.60146	4.37198
H	-3.25691	-4.04797	3.69032
H	-2.91891	-2.33268	3.83734
H	0.77826	-2.87659	4.63193
H	-0.42014	-1.60915	4.45276
H	-1.50257	-3.58388	5.42998
H	-0.90041	-4.60524	4.13706
C	-0.02454	-3.25585	-1.04579
C	-0.40602	-4.63203	-0.44415
C	-0.44152	-3.22809	-2.53977
H	1.05734	-3.16980	-1.01128
C	0.24688	-5.79279	-1.23241
H	-1.48559	-4.76117	-0.46214
H	-0.08122	-4.70577	0.58860
C	0.24383	-4.36705	-3.32565
H	-1.51659	-3.35945	-2.62469
H	-0.17602	-2.27180	-2.98417
C	-0.11009	-5.74487	-2.73090
H	-0.07206	-6.74077	-0.80305
H	1.32740	-5.73499	-1.12178
H	-0.06785	-4.32300	-4.36706
H	1.32269	-4.22777	-3.30377
H	0.42109	-6.53301	-3.26076
H	-1.17547	-5.92928	-2.85448
P	1.55596	1.41667	1.35671
C	2.97225	0.23435	1.76982
H	3.93135	0.67790	2.07653
H	2.63886	-0.42155	2.58812
C	4.45737	0.13630	-0.78714
C	5.87057	-0.04279	-0.17937
C	4.46458	-0.21896	-2.29325
H	4.20133	1.19090	-0.70806
C	6.90282	0.82357	-0.93656
H	6.17949	-1.08188	-0.24684
H	5.87545	0.23284	0.87277
C	5.50877	0.63016	-3.05271

H	4.70001	-1.27053	-2.43006
H	3.47502	-0.04378	-2.70793
C	6.91746	0.48088	-2.44074
H	7.89101	0.66509	-0.50913
H	6.65614	1.87557	-0.80670
H	5.52691	0.33037	-4.09836
H	5.21481	1.67688	-3.01898
H	7.61762	1.13229	-2.95955
H	7.26462	-0.54212	-2.57271
C	3.54630	-2.51791	0.64210
C	4.09749	-3.32754	-0.55663
C	4.52221	-2.60718	1.84309
H	2.61594	-2.98945	0.94936
C	4.34236	-4.80098	-0.16026
H	5.03730	-2.90068	-0.89451
H	3.40029	-3.28521	-1.39038
C	4.76993	-4.08139	2.23773
H	5.47339	-2.14582	1.59535
H	4.11868	-2.07860	2.70226
C	5.29965	-4.90353	1.04491
H	4.75665	-5.33967	-1.00991
H	3.39430	-5.27011	0.09332
H	5.48203	-4.11926	3.05967
H	3.83906	-4.51937	2.59279
H	5.41526	-5.94489	1.33784
H	6.28246	-4.53354	0.75926
C	2.42427	3.13701	1.12677
C	1.38654	4.28137	1.05333
C	3.56481	3.50934	2.10723
H	2.87156	3.04654	0.14069
C	2.05401	5.60533	0.62003
H	0.92263	4.42608	2.02565
H	0.60237	4.01986	0.34746
C	4.23723	4.83141	1.66839
H	3.18335	3.62920	3.11608
H	4.32021	2.72918	2.13487
C	3.21336	5.98095	1.56612
H	1.31113	6.40021	0.61169
H	2.43290	5.50177	-0.39467
H	5.01690	5.09235	2.38131
H	4.71510	4.68692	0.70136
H	3.70708	6.88158	1.20734
H	2.81642	6.19877	2.55583
C	0.52321	1.43654	2.98867
C	1.17977	2.10527	4.21923
C	-0.89768	2.00522	2.75920
C	0.30783	1.89745	5.47875
H	1.29010	3.17256	4.04992
H	2.17149	1.69289	4.39362
C	-1.77535	1.81858	4.01764
H	-0.84349	3.06393	2.52020
H	-1.36191	1.50433	1.91236
C	-1.11799	2.44750	5.26438
H	0.77284	2.39554	6.32716
H	0.25672	0.83578	5.71212
H	-2.75102	2.26940	3.84887
H	-1.93392	0.75626	4.19209
H	-1.72634	2.24596	6.14357
H	-1.07091	3.52778	5.14121
H	0.41280	0.37889	3.21496

$11b_c$

176

XYZ

Ru	-0.76842	0.08020	-0.56324
C	1.02997	-2.57387	-1.17301
C	0.28250	-1.46213	-1.40887
P	1.01827	1.03256	0.43566
N	-0.19104	-1.30401	-2.79060
C	0.47822	-0.51470	-3.73430
C	-0.11293	-0.82431	-5.11945
C	-1.36076	-1.67778	-4.82812
C	-1.03151	-2.31985	-3.46310
O	1.40614	0.26418	-3.50750
P	-2.71054	1.47792	-0.07911
H	1.27456	-3.21660	-2.03891
H	-0.46187	-3.26383	-3.58825
H	-1.92064	-2.54537	-2.85675
H	-0.30943	0.10913	-5.67275
H	0.64963	-1.38722	-5.69226
H	-1.57164	-2.43469	-5.60220
H	-2.25116	-1.03173	-4.73297
H	-0.15641	1.09297	-1.64892
C	1.55547	-3.09013	0.14163
C	1.26204	-4.59390	0.35669
H	2.65553	-2.94960	0.19838
H	1.14097	-2.49052	0.97078
C	1.93611	-5.17657	1.61314
H	0.16765	-4.75956	0.41079
H	1.61014	-5.15968	-0.53305
C	1.63437	-6.66972	1.82439
H	3.03166	-5.02566	1.53865
H	1.61187	-4.60361	2.50447
H	2.13459	-7.06141	2.72876
H	0.54834	-6.84654	1.93894
H	1.97898	-7.27285	0.96345
C	2.64931	0.74587	-0.51001
H	2.26609	0.41713	-1.49347
H	3.10952	1.73082	-0.66748
P	3.99949	-0.47109	-0.00143
C	5.37529	0.72103	0.63009
C	6.38540	-0.05593	1.51186
C	6.14559	1.59379	-0.38997
H	4.83918	1.40072	1.28924

C	7.36174	0.90224	2.23143
H	6.95746	-0.74912	0.89911
H	5.85208	-0.64750	2.25297
C	7.10286	2.57209	0.32929
H	6.73385	0.96192	-1.05000
H	5.45383	2.16006	-1.00968
C	8.10108	1.81218	1.22795
H	8.08365	0.32681	2.80773
H	6.80494	1.52044	2.93314
H	7.64561	3.16169	-0.40710
H	6.52369	3.26435	0.93748
H	8.73022	2.51982	1.76425
H	8.75374	1.20433	0.60421
C	4.48420	-1.22338	-1.70480
C	4.69170	-0.28907	-2.92354
C	5.70106	-2.16888	-1.54746
H	3.61875	-1.84361	-1.93018
C	4.92062	-1.11647	-4.20936
H	5.55039	0.35731	-2.77148
H	3.81913	0.34241	-3.06798
C	5.94243	-2.99369	-2.83153
H	6.59657	-1.59028	-1.33037
H	5.54140	-2.84533	-0.71008
C	6.11983	-2.07625	-4.05939
H	5.08977	-0.44523	-5.04911
H	4.02379	-1.69206	-4.42912
H	6.82440	-3.61911	-2.70622
H	5.09443	-3.65546	-2.99644
H	6.22749	-2.67952	-4.95869
H	7.03392	-1.49631	-3.94586
C	1.14865	0.50755	2.31638
C	2.53780	0.45679	2.99902
C	0.16148	1.34188	3.17134
H	0.77259	-0.51220	2.29645
C	2.43846	-0.06174	4.45215
H	2.99848	1.43988	3.01294
H	3.19124	-0.20408	2.43819
C	0.07301	0.82889	4.62667
H	0.47068	2.38270	3.19791
H	-0.82730	1.31413	2.72010
C	1.46403	0.78968	5.29060
H	3.42599	-0.05043	4.90914
H	2.09643	-1.09483	4.44381
H	-0.59414	1.47098	5.19887
H	-0.35180	-0.17182	4.63599
H	1.38230	0.38144	6.29593
H	1.85130	1.80280	5.38008
C	1.06672	2.99413	0.46389
C	2.24690	3.60291	1.26723
C	1.05959	3.58079	-0.97122
H	0.14555	3.30373	0.95139
C	2.21259	5.14848	1.26743

H	3.19405	3.28021	0.84347
H	2.22325	3.26363	2.29705
C	1.03793	5.12678	-0.97067
H	1.94206	3.25235	-1.51221
H	0.20084	3.20378	-1.51635
C	2.21657	5.71014	-0.16726
H	3.07089	5.52758	1.81885
H	1.31809	5.48986	1.78518
H	1.07620	5.48531	-1.99730
H	0.10631	5.48278	-0.53784
H	2.15068	6.79600	-0.14185
H	3.15402	5.45040	-0.65496
P	-2.42039	-1.20645	0.86862
C	-3.46318	0.33056	1.21213
H	-4.55971	0.22853	1.23019
H	-3.14557	0.69153	2.20477
C	-3.95457	1.30415	-1.55662
C	-5.39628	1.82360	-1.33744
C	-3.36611	1.88899	-2.86314
H	-4.01530	0.22582	-1.69012
C	-6.29427	1.46506	-2.54391
H	-5.39153	2.90356	-1.22059
H	-5.82555	1.39817	-0.43314
C	-4.26349	1.54427	-4.07298
H	-3.28104	2.96937	-2.78429
H	-2.36502	1.49351	-3.02155
C	-5.71299	2.02821	-3.85762
H	-7.29467	1.86118	-2.38110
H	-6.38081	0.38289	-2.62039
H	-3.85189	1.99956	-4.97129
H	-4.26516	0.46691	-4.22541
H	-6.33425	1.72096	-4.69628
H	-5.72962	3.11575	-3.82067
C	-2.93549	3.23053	0.72899
C	-2.82501	4.38177	-0.29844
C	-4.21135	3.40994	1.59095
H	-2.08274	3.30617	1.40085
C	-2.80632	5.75405	0.41175
H	-3.67252	4.35534	-0.97761
H	-1.92422	4.26620	-0.89500
C	-4.19713	4.78238	2.30311
H	-5.10087	3.34498	0.97165
H	-4.27962	2.62805	2.34190
C	-4.05727	5.94136	1.29476
H	-2.75592	6.54629	-0.33228
H	-1.91426	5.82932	1.03014
H	-5.11405	4.89948	2.87725
H	-3.36667	4.81317	3.00582
H	-3.99553	6.88768	1.82792
H	-4.94300	5.97944	0.66353
C	-3.54780	-2.34834	-0.23586
C	-3.04210	-3.81130	-0.22950

C	-5.07673	-2.30417	0.01260
H	-3.37659	-1.95511	-1.23550
C	-3.79549	-4.67030	-1.26971
H	-3.19219	-4.25012	0.75311
H	-1.97575	-3.83348	-0.44378
C	-5.82406	-3.16073	-1.03600
H	-5.31629	-2.67663	1.00357
H	-5.44421	-1.28391	-0.04937
C	-5.31973	-4.61827	-1.03929
H	-3.44613	-5.69881	-1.20812
H	-3.56967	-4.30937	-2.27072
H	-6.89114	-3.13770	-0.82397
H	-5.67884	-2.72690	-2.02350
H	-5.83109	-5.18278	-1.81607
H	-5.55848	-5.08498	-0.08554
C	-2.36512	-1.92554	2.67282
C	-3.73750	-2.33670	3.25877
C	-1.34083	-3.06867	2.86296
C	-3.60411	-2.69461	4.75653
H	-4.12253	-3.20454	2.73087
H	-4.45988	-1.53190	3.14409
C	-1.21126	-3.45425	4.35459
H	-1.64476	-3.94524	2.29852
H	-0.37032	-2.76012	2.48675
C	-2.57778	-3.82717	4.96606
H	-4.57498	-2.99745	5.14359
H	-3.29151	-1.81284	5.31255
H	-0.52322	-4.29142	4.45118
H	-0.78638	-2.61923	4.90760
H	-2.46363	-4.02959	6.02888
H	-2.94723	-4.73769	4.49826
H	-2.00783	-1.07409	3.24824

12b$_c$

176

XYZ

Ru	-0.52748	-0.11720	-0.33406
C	-1.32711	-2.83827	-1.81307
C	-0.53509	-1.73655	-1.62187
P	0.88216	-1.04034	1.14462
N	0.49700	-1.65416	-2.64899
C	1.54544	-2.53924	-2.76074
C	2.29062	-2.20677	-4.06645
C	1.84422	-0.77158	-4.39650
C	0.45669	-0.65174	-3.71803
O	1.82183	-3.45151	-1.96744
P	-0.79189	2.18676	0.40300
H	-1.10422	-3.44401	-2.70978
H	-0.37718	-0.87423	-4.41852
H	0.28244	0.35163	-3.28691
H	3.37728	-2.32797	-3.93458
H	1.96851	-2.93366	-4.83805
H	1.79830	-0.55429	-5.47706
H	2.53905	-0.05330	-3.92841
H	0.90048	0.22064	-0.97742
C	-2.43434	-3.41082	-0.97567
C	-3.59790	-3.98785	-1.81659
H	-2.02323	-4.25460	-0.38377
H	-2.78637	-2.66462	-0.23957
C	-4.53719	-4.91474	-1.02357
H	-4.18137	-3.16690	-2.27613
H	-3.16968	-4.56248	-2.66362
C	-5.69726	-5.46443	-1.86861
H	-3.94573	-5.75787	-0.61317
H	-4.94401	-4.37699	-0.14460
H	-6.34631	-6.13710	-1.27936
H	-6.33005	-4.64629	-2.26041
H	-5.32177	-6.03686	-2.73715
C	2.68148	-0.41771	1.08677
H	2.60582	0.61096	1.46762
H	3.30158	-0.96973	1.80491
P	3.51830	-0.27084	-0.61421
P	-3.09655	0.44950	0.09432
C	-2.61703	2.09394	0.88037
H	-3.26823	2.95511	0.66258
H	-2.61409	1.94487	1.97337
C	-3.87439	1.04107	-1.57452
C	-4.04308	-0.12787	-2.57398
C	-5.18295	1.86390	-1.48860
H	-3.10646	1.69638	-1.97812
C	-4.44214	0.39845	-3.97054
H	-4.81275	-0.80851	-2.22075
H	-3.11451	-0.69067	-2.64545
C	-5.57838	2.39995	-2.88403
H	-5.99199	1.24369	-1.11315
H	-5.06782	2.70120	-0.80423
C	-5.72648	1.25163	-3.90422
H	-4.59188	-0.44206	-4.64485
H	-3.62965	0.99917	-4.37470
H	-6.51379	2.95077	-2.80777
H	-4.81759	3.09660	-3.23069
H	-5.95065	1.66059	-4.88715
H	-6.56449	0.62037	-3.61494
C	-4.50103	-0.18784	1.28231
C	-5.36296	0.91537	1.94796
C	-5.42011	-1.24229	0.62071
H	-3.94571	-0.68896	2.07382
C	-6.32566	0.29785	2.98874
H	-5.94525	1.44362	1.19839
H	-4.73470	1.64658	2.44780
C	-6.37896	-1.86640	1.65912
H	-6.01086	-0.78061	-0.16555
H	-4.82404	-2.02614	0.16470
C	-7.22653	-0.78328	2.35732

H	-6.93951	1.08518	3.42167
H	-5.74430	-0.14305	3.79623
H	-7.02985	-2.58391	1.16389
H	-5.79994	-2.40994	2.40311
H	-7.84918	-1.23775	3.12498
H	-7.89065	-0.31997	1.63019
C	-0.74040	3.20319	-1.25555
C	-1.72415	4.38849	-1.42094
C	0.69801	3.65646	-1.59738
H	-1.01240	2.44851	-1.99088
C	-1.66029	4.94376	-2.86298
H	-1.47785	5.18761	-0.72878
H	-2.74349	4.07796	-1.20904
C	0.77018	4.21438	-3.03629
H	1.01770	4.43185	-0.90585
H	1.37920	2.81460	-1.49943
C	-0.22696	5.37459	-3.23776
H	-2.33748	5.79073	-2.95431
H	-2.00013	4.17840	-3.55805
H	1.78192	4.55730	-3.24232
H	0.54620	3.41687	-3.74182
H	-0.20254	5.70832	-4.27297
H	0.06911	6.21766	-2.61637
C	-0.18214	3.30211	1.87811
C	1.35391	3.45910	1.96602
C	-0.84987	4.69484	1.97841
H	-0.49812	2.72616	2.74629
C	1.75765	4.14852	3.28951
H	1.71897	4.05321	1.13309
H	1.83396	2.48753	1.90745
C	-0.46762	5.38251	3.30924
H	-0.51823	5.32393	1.15720
H	-1.93195	4.61093	1.91509
C	1.06338	5.51761	3.44620
H	2.83769	4.27729	3.31631
H	1.48524	3.50743	4.12567
H	-0.93107	6.36599	3.35475
H	-0.85716	4.79945	4.14154
H	1.31056	5.94821	4.41423
H	1.43326	6.19871	2.68220
C	4.26019	1.49859	-0.44230
C	5.40106	1.73239	0.57818
C	4.67404	2.06124	-1.82507
H	3.40732	2.08692	-0.11060
C	5.75510	3.23364	0.68413
H	6.29206	1.19254	0.26917
H	5.11710	1.35965	1.56019
C	5.04122	3.56030	-1.73605
H	5.52899	1.51384	-2.21376
H	3.85886	1.93161	-2.53357
C	6.15073	3.80844	-0.69234
H	6.57326	3.36931	1.38897
H	4.89813	3.78150	1.07124
H	5.36721	3.91729	-2.71108
H	4.15673	4.13124	-1.46043
H	6.34603	4.87552	-0.60603
H	7.07198	3.33529	-1.02680
C	4.93454	-1.57839	-0.58104
C	5.86083	-1.39602	-1.80912
C	5.80833	-1.76396	0.68690
H	4.39198	-2.50947	-0.73599
C	6.80374	-2.60757	-1.98388
H	6.46516	-0.49974	-1.68916
H	5.26623	-1.26866	-2.71083
C	6.74646	-2.98360	0.53148
H	6.41497	-0.88069	0.86288
H	5.19077	-1.91018	1.56863
C	7.64725	-2.84473	-0.71371
H	7.46005	-2.44260	-2.83625
H	6.21126	-3.49557	-2.19502
H	7.36357	-3.08627	1.42217
H	6.14789	-3.88822	0.44392
H	8.25334	-3.74053	-0.83322
H	8.32846	-2.00756	-0.57270
C	1.06078	-2.97196	1.07999
C	2.39867	-3.55391	1.59729
C	-0.11097	-3.68418	1.79526
H	1.00502	-3.19072	0.01411
C	2.45704	-5.08101	1.36402
H	2.52597	-3.35078	2.65774
H	3.23234	-3.10228	1.07405
C	-0.06621	-5.20900	1.55054
H	-0.05216	-3.51105	2.86665
H	-1.06041	-3.28612	1.45080
C	1.27151	-5.80486	2.03297
H	3.39584	-5.47047	1.75343
H	2.43921	-5.27333	0.29318
H	-0.89511	-5.68681	2.06952
H	-0.18315	-5.40846	0.48793
H	1.30902	-6.86766	1.80283
H	1.34555	-5.70249	3.11424
C	0.54241	-0.50159	2.99926
C	-0.93937	-0.66621	3.40665
C	1.44258	-1.10035	4.10920
H	0.74514	0.56669	2.96874
C	-1.23203	0.01447	4.76328
H	-1.19483	-1.71856	3.48604
H	-1.57758	-0.23987	2.63830
C	1.16810	-0.42693	5.47465
H	1.25841	-2.16696	4.20626
H	2.49332	-0.97273	3.86360
C	-0.31769	-0.54527	5.87299
H	-2.27521	-0.13778	5.03410
H	-1.07287	1.08749	4.67498

H	1.79131	-0.88543	6.23999
H	1.44338	0.62464	5.41928
H	-0.49586	-0.01187	6.80458
H	-0.56197	-1.59176	6.04475

[12b$_c$-13b$_c$]‡

176

XYZ

Ru	0.71541	-0.21691	-0.30302
C	2.41421	0.33038	-3.10799
C	1.55973	-0.31859	-2.26879
P	-0.67490	1.62003	-0.25767
N	0.98298	-1.54116	-2.84856
C	-0.18770	-1.51048	-3.59194
C	-0.25783	-2.79383	-4.43343
C	0.85768	-3.69777	-3.87576
C	1.81565	-2.71896	-3.14830
O	-1.01179	-0.58629	-3.61537
P	0.11937	-1.42153	1.64374
H	2.57829	-0.12207	-4.10290
H	2.66799	-2.42431	-3.79268
H	2.23036	-3.14035	-2.21640
H	-1.26651	-3.23652	-4.39106
H	-0.07736	-2.50244	-5.48626
H	1.38479	-4.27191	-4.65592
H	0.43717	-4.42223	-3.16007
H	0.13661	0.31184	-1.78209
C	3.16777	1.61433	-2.89219
C	4.66629	1.51660	-3.26604
H	2.72107	2.40962	-3.53012
H	3.05246	1.94709	-1.84634
C	5.40568	2.86584	-3.19483
H	5.16616	0.78287	-2.60396
H	4.75514	1.10885	-4.29455
C	6.89413	2.76030	-3.56703
H	4.90575	3.58991	-3.86957
H	5.30888	3.28497	-2.17330
H	7.39711	3.74268	-3.51057
H	7.42837	2.07047	-2.88721
H	7.02226	2.37675	-4.59657
C	-2.34225	1.45619	-1.15208
H	-2.86474	2.41874	-1.21744
H	-2.06003	1.14583	-2.17216
P	-3.48463	0.10951	-0.47518
P	2.67403	-0.53702	1.05757
C	1.76386	-1.25573	2.54982
H	2.19410	-2.16304	3.00799
H	1.66223	-0.48959	3.33368
C	3.96656	-1.92468	0.66301
C	4.68823	-1.69730	-0.68615
C	5.00475	-2.23460	1.76771
H	3.33945	-2.80764	0.54976
C	5.50848	-2.94736	-1.07604
H	5.36156	-0.84911	-0.60707
H	3.96478	-1.46609	-1.46410
C	5.82302	-3.49226	1.39356
H	5.68780	-1.39733	1.88123
H	4.51731	-2.39447	2.72679
C	6.52157	-3.31972	0.02783
H	6.03439	-2.75963	-2.00975
H	4.83417	-3.78468	-1.24487
H	6.56452	-3.68555	2.16626
H	5.15979	-4.35436	1.35572
H	7.03855	-4.23928	-0.23843
H	7.27197	-2.53543	0.10652
C	3.59883	1.00802	1.79492
C	4.14497	0.86756	3.23927
C	4.72214	1.53078	0.86890
H	2.81504	1.76186	1.81719
C	4.67640	2.22727	3.74976
H	4.95165	0.14140	3.27110
H	3.37060	0.52198	3.91760
C	5.25633	2.89278	1.36592
H	5.54533	0.82169	0.85054
H	4.35366	1.63408	-0.14725
C	5.76730	2.79460	2.81820
H	5.07540	2.10377	4.75464
H	3.85093	2.93379	3.81175
H	6.06031	3.22618	0.71311
H	4.46193	3.63428	1.31004
H	6.07725	3.77648	3.16961
H	6.64071	2.14607	2.85012
C	0.15107	-3.28109	1.08989
C	0.14294	-4.33826	2.22219
C	-0.95826	-3.59239	0.05814
H	1.10427	-3.36163	0.57019
C	0.30651	-5.76601	1.65414
H	-0.79745	-4.29320	2.76390
H	0.94113	-4.14259	2.93452
C	-0.80958	-5.02620	-0.49920
H	-1.93689	-3.49330	0.51918
H	-0.90578	-2.87456	-0.75814
C	-0.80145	-6.07896	0.62810
H	0.27392	-6.48594	2.46951
H	1.27996	-5.85780	1.17621
H	-1.62199	-5.23137	-1.19314
H	0.12222	-5.10014	-1.05448
H	-0.64649	-7.07000	0.20679
H	-1.76712	-6.08431	1.12990
C	-1.19278	-1.20512	3.07169
C	-2.59473	-1.72927	2.68323
C	-0.77134	-1.76966	4.45229
H	-1.26616	-0.12673	3.17767
C	-3.63717	-1.35692	3.76157

H	-2.57266	-2.81079	2.58322
H	-2.89500	-1.31028	1.72429
C	-1.81385	-1.39852	5.53242
H	-0.68088	-2.85065	4.41110
H	0.19553	-1.37391	4.75005
C	-3.22462	-1.89066	5.14906
H	-4.60686	-1.76480	3.48413
H	-3.73999	-0.27449	3.80648
H	-1.51602	-1.83321	6.48453
H	-1.83071	-0.31794	5.65960
H	-3.94340	-1.56763	5.89919
H	-3.23503	-2.97883	5.13465
C	-5.00213	1.10080	0.18556
C	-5.61981	2.24001	-0.66555
C	-6.12477	0.13090	0.63187
H	-4.60672	1.56323	1.08902
C	-6.70812	2.99422	0.13355
H	-6.06902	1.83822	-1.56838
H	-4.85811	2.95091	-0.97374
C	-7.22415	0.86766	1.42963
H	-6.57866	-0.33384	-0.24041
H	-5.70876	-0.66644	1.24390
C	-7.81750	2.03568	0.61456
H	-7.14130	3.77546	-0.48807
H	-6.25251	3.47929	0.99477
H	-8.01289	0.16809	1.69959
H	-6.80007	1.25272	2.35503
H	-8.54040	2.57960	1.21924
H	-8.34817	1.63857	-0.24865
C	-3.91813	-0.81481	-2.09993
C	-4.54203	-2.20077	-1.80063
C	-4.77393	-0.07815	-3.15788
H	-2.93815	-0.98417	-2.54412
C	-4.66961	-3.04703	-3.08775
H	-5.52909	-2.08188	-1.35928
H	-3.92911	-2.73607	-1.07894
C	-4.87185	-0.91070	-4.45608
H	-5.77916	0.08836	-2.77857
H	-4.34119	0.89325	-3.38652
C	-5.47354	-2.30398	-4.17559
H	-5.15064	-3.99588	-2.85735
H	-3.67503	-3.27012	-3.46788
H	-5.48647	-0.38496	-5.18434
H	-3.87794	-1.02270	-4.88463
H	-5.48734	-2.89321	-5.09033
H	-6.50492	-2.19082	-3.84646
C	0.11336	3.09971	-1.26452
C	-0.87698	4.06094	-1.96922
C	1.12819	3.90735	-0.41904
H	0.66912	2.58436	-2.04251
C	-0.12330	5.11267	-2.81619
H	-1.49921	4.57533	-1.24125
H	-1.53443	3.50405	-2.62956
C	1.87620	4.95928	-1.26871
H	0.61559	4.42675	0.38549
H	1.85014	3.23196	0.03303
C	0.88670	5.91160	-1.96893
H	-0.84326	5.79022	-3.27111
H	0.40469	4.60851	-3.62309
H	2.54875	5.52780	-0.62925
H	2.48347	4.45734	-2.01760
H	1.42956	6.61076	-2.60163
H	0.35246	6.49489	-1.22117
C	-1.29817	2.31315	1.45706
C	-0.16387	2.44330	2.50052
C	-2.11642	3.62941	1.43216
H	-1.96897	1.52963	1.80436
C	-0.71030	2.78750	3.90561
H	0.53042	3.22354	2.20428
H	0.40231	1.51792	2.55005
C	-2.67978	3.97391	2.83057
H	-1.49008	4.45389	1.10291
H	-2.94344	3.55350	0.73211
C	-1.55133	4.08016	3.87648
H	0.11822	2.90170	4.60214
H	-1.32858	1.96936	4.26841
H	-3.22734	4.91316	2.78231
H	-3.38376	3.20269	3.13741
H	-1.97333	4.26954	4.86140
H	-0.90882	4.92304	3.62932

$13b_c$

176

XYZ

Ru	1.10466	0.26393	-0.26026
C	2.12233	1.51561	-1.77035
C	0.93926	0.87954	-2.31732
P	-0.86641	1.57147	0.48651
N	0.93944	-0.02793	-3.41633
C	-0.18505	-0.15175	-4.23265
C	0.22024	-0.98215	-5.45995
C	1.55287	-1.64043	-5.05561
C	2.11737	-0.71172	-3.94821
O	-1.28354	0.36757	-4.01639
P	0.55212	-2.04607	0.22662
H	3.05743	1.01343	-2.06605
H	2.84357	0.02414	-4.35094
H	2.62218	-1.27863	-3.14621
H	-0.57683	-1.69345	-5.73124
H	0.33986	-0.28793	-6.31473
H	2.26172	-1.75239	-5.89276
H	1.36783	-2.64731	-4.64110
H	0.00778	1.44946	-2.38833
C	2.33419	3.03430	-1.77761

C	3.69446	3.47406	-1.20410
H	2.26622	3.41558	-2.82224
H	1.54255	3.54835	-1.21537
C	3.89498	5.00142	-1.20614
H	3.79646	3.09100	-0.16864
H	4.51089	3.00592	-1.79237
C	5.26339	5.43482	-0.65375
H	3.77524	5.38416	-2.23994
H	3.08929	5.47628	-0.61052
H	5.37724	6.53399	-0.66604
H	5.39842	5.09537	0.39033
H	6.08959	5.00583	-1.25129
C	-2.40325	0.68850	1.18785
H	-1.99321	0.01921	1.96574
H	-3.02384	1.43281	1.69985
P	-3.46629	-0.53168	0.19373
P	3.06281	-0.89872	0.08886
C	2.36715	-2.62724	0.08341
H	2.50023	-3.07095	-0.91551
H	2.76311	-3.33958	0.82183
C	4.71675	-1.01950	-0.93071
C	5.72832	0.10086	-0.59588
C	5.41965	-2.39935	-0.87331
H	4.38929	-0.86350	-1.95489
C	6.91958	0.07216	-1.57946
H	6.11010	-0.02980	0.41290
H	5.24113	1.07026	-0.64427
C	6.61894	-2.43839	-1.84883
H	5.77543	-2.60227	0.13321
H	4.72808	-3.19369	-1.14006
C	7.62181	-1.30155	-1.56214
H	7.62896	0.85304	-1.31317
H	6.56177	0.28491	-2.58481
H	7.11878	-3.40175	-1.76877
H	6.25187	-2.34584	-2.86915
H	8.41934	-1.31991	-2.30196
H	8.07812	-1.46098	-0.58696
C	3.49937	-0.70330	1.97999
C	4.55889	-1.67322	2.56106
C	3.86718	0.74913	2.35731
H	2.54840	-0.93148	2.45960
C	4.65927	-1.52740	4.09674
H	5.53316	-1.46041	2.12948
H	4.31464	-2.70432	2.31771
C	3.97239	0.91284	3.89106
H	4.81864	1.02333	1.91151
H	3.11818	1.43140	1.96338
C	4.99261	-0.07499	4.49502
H	5.42471	-2.20053	4.47786
H	3.71383	-1.81952	4.54997
H	4.26727	1.93243	4.12992
H	2.99673	0.74098	4.34154
H	5.00062	0.01893	5.57897
H	5.99002	0.17511	4.13875
C	-0.08638	-2.78033	1.93425
C	-1.56685	-3.22708	1.88249
C	0.77726	-3.90077	2.56450
H	-0.03161	-1.92110	2.60174
C	-2.09211	-3.57813	3.29217
H	-1.66931	-4.10479	1.25254
H	-2.17926	-2.44482	1.44450
C	0.24997	-4.28365	3.96795
H	0.77635	-4.78250	1.92815
H	1.80722	-3.57396	2.67111
C	-1.23743	-4.68884	3.93547
H	-3.12847	-3.90221	3.22299
H	-2.06824	-2.69115	3.92246
H	0.84815	-5.10152	4.36470
H	0.37575	-3.43514	4.63786
H	-1.58621	-4.88913	4.94638
H	-1.35032	-5.60732	3.36282
C	-0.08285	-3.17648	-1.22877
C	0.10861	-4.69791	-1.00129
C	-1.51620	-2.87688	-1.71610
H	0.58794	-2.87485	-2.03043
C	-0.20619	-5.49032	-2.29038
H	-0.55045	-5.04873	-0.21287
H	1.12767	-4.91461	-0.69152
C	-1.84027	-3.67929	-2.99740
H	-2.24159	-3.12768	-0.94755
H	-1.60976	-1.81383	-1.92399
C	-1.63542	-5.19402	-2.78842
H	-0.09216	-6.55540	-2.09796
H	0.50994	-5.22034	-3.06407
H	-2.86775	-3.48567	-3.29714
H	-1.19732	-3.33915	-3.80572
H	-1.81855	-5.72440	-3.72058
H	-2.35411	-5.56066	-2.05793
C	-4.81277	-0.89606	1.52904
C	-5.56870	0.27418	2.20984
C	-5.83282	-1.93277	0.99561
H	-4.23872	-1.39007	2.31148
C	-6.47368	-0.24738	3.35034
H	-6.18745	0.79982	1.48938
H	-4.86915	0.99507	2.62408
C	-6.74772	-2.45796	2.12466
H	-6.45326	-1.47888	0.22636
H	-5.30823	-2.76890	0.53765
C	-7.47780	-1.29974	2.83586
H	-7.01098	0.58628	3.79834
H	-5.85421	-0.69034	4.12790
H	-7.47466	-3.15486	1.71225
H	-6.14752	-3.00461	2.84947
H	-8.06562	-1.68655	3.66579

H	-8.16831	-0.82774	2.13956
C	-4.23334	0.37829	-1.31157
C	-4.76452	-0.66325	-2.32880
C	-5.32275	1.45090	-1.07102
H	-3.38146	0.85913	-1.78774
C	-5.15080	0.01648	-3.66133
H	-5.63400	-1.17524	-1.92269
H	-4.00353	-1.41427	-2.51999
C	-5.70019	2.15450	-2.39470
H	-6.21540	0.98186	-0.66556
H	-4.98746	2.18986	-0.34795
C	-6.19194	1.13456	-3.44306
H	-5.55009	-0.72744	-4.34778
H	-4.25523	0.43540	-4.11643
H	-6.47555	2.89499	-2.20713
H	-4.83170	2.68144	-2.78444
H	-6.39260	1.64198	-4.38437
H	-7.12856	0.69576	-3.10369
C	-0.05488	2.23305	2.14686
C	0.78402	3.50922	1.88441
C	-0.99725	2.43910	3.36218
H	0.63716	1.43829	2.41841
C	1.54585	3.97595	3.14492
H	0.13793	4.31795	1.55851
H	1.49487	3.31786	1.08501
C	-0.20603	2.88563	4.61300
H	-1.74822	3.19103	3.14236
H	-1.51940	1.51621	3.59663
C	0.59420	4.17468	4.34124
H	2.06414	4.90663	2.92379
H	2.29967	3.23957	3.40867
H	-0.89903	3.04423	5.43691
H	0.47748	2.09294	4.91093
H	1.16393	4.45028	5.22619
H	-0.09464	4.98997	4.12875
C	-1.87598	3.07787	-0.31607
C	-2.64229	3.97996	0.68798
C	-1.05310	3.97174	-1.27305
H	-2.61401	2.56096	-0.92111
C	-3.54753	4.99655	-0.04781
H	-1.94062	4.53475	1.30292
H	-3.26390	3.39051	1.35436
C	-1.95768	4.97959	-2.01737
H	-0.30472	4.52535	-0.71404
H	-0.53638	3.36008	-2.00367
C	-2.74025	5.86730	-1.03014
H	-4.04356	5.62666	0.68794
H	-4.32231	4.46423	-0.59432
H	-1.34243	5.59929	-2.66620
H	-2.65687	4.43836	-2.65149
H	-3.41137	6.52967	-1.57286
H	-2.04417	6.49146	-0.47314

[13b$_c$-1$_c$]‡

176

XYZ

Ru	-0.94344	-0.12389	-0.02031
C	-1.88090	-2.25054	-1.77120
C	-0.72405	-2.81284	-2.23738
P	0.86861	-0.93825	1.24155
N	0.23762	-2.22498	-3.03015
C	1.28430	-2.97976	-3.59745
C	2.00999	-2.05984	-4.58206
C	1.57486	-0.63640	-4.17763
C	0.21100	-0.82601	-3.46478
O	1.50278	-4.16323	-3.35006
P	-0.67861	2.08265	0.06684
H	-2.15437	-1.22389	-2.05835
H	-0.65409	-0.65999	-4.13908
H	0.08589	-0.18188	-2.56641
H	3.09723	-2.23455	-4.54541
H	1.67192	-2.32836	-5.60265
H	1.49546	0.04991	-5.03590
H	2.29775	-0.21233	-3.45945
H	-0.49769	-3.87314	-2.06164
C	-2.95342	-3.14486	-1.17527
C	-3.89928	-3.73992	-2.24982
H	-2.48541	-3.98266	-0.62228
H	-3.55504	-2.58788	-0.43604
C	-5.02692	-4.60270	-1.65305
H	-4.34001	-2.92117	-2.85363
H	-3.29596	-4.34645	-2.95494
C	-5.95032	-5.20972	-2.72266
H	-4.58137	-5.41462	-1.04355
H	-5.62547	-3.98696	-0.95176
H	-6.74877	-5.82250	-2.26670
H	-6.43615	-4.42022	-3.32586
H	-5.38475	-5.85826	-3.41721
C	2.54453	-0.04184	1.16253
H	2.29126	1.00558	1.38699
H	3.23637	-0.38147	1.94618
P	3.39763	0.01111	-0.53119
P	-3.05016	0.87192	0.20863
C	-2.38819	2.58696	0.63769
H	-2.85305	3.45962	0.15365
H	-2.41810	2.73229	1.72983
C	-4.38190	1.23813	-1.18697
C	-5.28354	0.01337	-1.46766
C	-5.26745	2.49999	-1.02814
H	-3.76003	1.39164	-2.06790
C	-6.14511	0.22812	-2.73272
H	-5.94389	-0.16114	-0.62190
H	-4.67579	-0.87685	-1.59616
C	-6.12334	2.73123	-2.29583

H	-5.93093	2.39363	-0.17559
H	-4.66032	3.38360	-0.85618
C	-7.00303	1.50455	-2.61375
H	-6.78805	-0.63616	-2.88707
H	-5.49480	0.30968	-3.60154
H	-6.75116	3.60880	-2.15406
H	-5.46750	2.93357	-3.14049
H	-7.54990	1.67177	-3.53942
H	-7.73678	1.37282	-1.82084
C	-3.97798	0.39362	1.85865
C	-5.19448	1.26159	2.26240
C	-4.35436	-1.10101	1.91917
H	-3.19210	0.55631	2.59394
C	-5.68583	0.89015	3.68074
H	-6.01291	1.10422	1.56497
H	-4.93862	2.31893	2.23534
C	-4.85952	-1.49075	3.32664
H	-5.13209	-1.32476	1.19317
H	-3.47700	-1.69218	1.66525
C	-6.04716	-0.60782	3.76634
H	-6.55330	1.49502	3.93826
H	-4.90481	1.11590	4.40427
H	-5.15869	-2.53704	3.33109
H	-4.04805	-1.38163	4.04250
H	-6.33911	-0.85965	4.78389
H	-6.90278	-0.80690	3.12375
C	0.48699	3.29866	1.06921
C	0.18286	4.79934	0.83421
C	0.42829	2.99736	2.58521
H	1.49952	3.10314	0.72424
C	1.12499	5.71628	1.64851
H	-0.84699	5.01493	1.11139
H	0.29691	5.04721	-0.21495
C	1.38309	3.90317	3.39448
H	-0.58422	3.14478	2.95305
H	0.68957	1.96266	2.76740
C	1.07459	5.39378	3.15449
H	0.84749	6.75518	1.48137
H	2.14442	5.59112	1.28889
H	1.29082	3.66993	4.45331
H	2.41124	3.69642	3.10397
H	1.78996	6.01372	3.69082
H	0.08471	5.62582	3.54260
C	-0.61903	2.76709	-1.82381
C	-1.44337	4.02173	-2.21460
C	0.84431	2.91328	-2.29756
H	-1.05386	1.92442	-2.35390
C	-1.35401	4.29787	-3.73476
H	-1.09696	4.90289	-1.68397
H	-2.48891	3.88070	-1.95556
C	0.93055	3.19946	-3.81409
H	1.33279	3.72621	-1.76571

H	1.39056	2.00000	-2.07132
C	0.11020	4.44885	-4.19494
H	-1.91243	5.20107	-3.97369
H	-1.81732	3.47630	-4.27778
H	1.97090	3.33665	-4.10269
H	0.54939	2.34286	-4.36522
H	0.14588	4.60199	-5.27165
H	0.54830	5.32776	-3.72582
C	4.24112	1.74386	-0.44605
C	4.97110	2.17065	0.85352
C	5.16853	1.97271	-1.66511
H	3.39662	2.41991	-0.56381
C	5.41679	3.64914	0.77202
H	5.84933	1.55503	1.01859
H	4.32134	2.04418	1.71553
C	5.62666	3.44586	-1.75488
H	6.04827	1.33818	-1.58731
H	4.65128	1.69992	-2.58283
C	6.32230	3.89547	-0.45295
H	5.94718	3.92119	1.68256
H	4.53859	4.28819	0.70417
H	6.30530	3.56797	-2.59680
H	4.76198	4.08049	-1.93918
H	6.57951	4.95084	-0.51646
H	7.25074	3.34087	-0.33083
C	4.74140	-1.36085	-0.39112
C	5.28099	-1.72618	-1.79705
C	5.92820	-1.15434	0.58107
H	4.18632	-2.22557	-0.03874
C	6.17896	-2.98311	-1.74689
H	5.85469	-0.89782	-2.20591
H	4.44965	-1.90795	-2.47375
C	6.81238	-2.42058	0.65199
H	6.54488	-0.32568	0.24373
H	5.56951	-0.91126	1.57883
C	7.33982	-2.80785	-0.74548
H	6.57613	-3.18889	-2.73892
H	5.57747	-3.84131	-1.45368
H	7.64869	-2.24457	1.32579
H	6.23218	-3.24513	1.06132
H	7.91476	-3.72942	-0.68166
H	8.01091	-2.02957	-1.10398
C	0.42617	-0.72823	3.15308
C	-0.50667	-1.84715	3.66409
C	1.59248	-0.51774	4.15386
H	-0.16967	0.17868	3.14705
C	-1.06157	-1.50592	5.06503
H	0.02961	-2.78862	3.73330
H	-1.32665	-1.97371	2.96047
C	1.05499	-0.19703	5.56914
H	2.21595	-1.40606	4.20745
H	2.22752	0.30477	3.83745

C	0.08484	-1.28483	6.07355
H	-1.70572	-2.31215	5.41053
H	-1.66850	-0.60479	5.00646
H	1.88988	-0.09681	6.26023
H	0.53742	0.76006	5.54546
H	-0.31986	-0.99744	7.04193
H	0.62834	-2.21759	6.21148
C	1.48618	-2.77671	1.02533
C	2.53770	-3.28381	2.04463
C	0.33227	-3.80385	0.95493
H	1.95823	-2.76290	0.04419
C	3.08071	-4.67088	1.62598
H	2.08613	-3.37302	3.02844
H	3.37014	-2.59216	2.13321
C	0.85570	-5.18726	0.50777
H	-0.13524	-3.91083	1.92766
H	-0.43058	-3.45311	0.27070
C	1.94323	-5.70225	1.47377
H	3.79743	-5.01870	2.36757
H	3.61042	-4.58019	0.67989
H	0.03115	-5.89647	0.47254
H	1.26782	-5.11552	-0.49708
H	2.34785	-6.64506	1.11134
H	1.49787	-5.89191	2.44858

$7a_c$-I

150

XYZ

Ru	-0.13719	0.45907	-0.40539
C	-2.28893	2.70930	0.20196
C	-1.44229	1.72863	-0.10762
P	-1.86286	-1.19297	-0.61425
N	1.01035	2.10675	-1.28861
C	1.21376	2.06765	-2.62584
C	1.61502	3.46165	-3.15031
C	1.96446	4.24310	-1.87507
C	1.17312	3.47873	-0.77968
O	1.09511	1.06312	-3.38475
P	2.02048	-0.88825	-0.53024
H	-2.31982	3.02035	1.25984
H	0.18244	3.94149	-0.59277
H	1.71766	3.47850	0.17822
H	2.42880	3.38771	-3.89103
H	0.73543	3.88633	-3.67374
H	1.70171	5.31519	-1.91386
H	3.04960	4.17432	-1.67203
H	-0.25455	0.31044	-2.00154
C	-3.19673	3.46855	-0.75562
C	-2.90121	4.98469	-0.79593
H	-3.10276	3.03177	-1.76533
H	-4.25406	3.32235	-0.44676
C	-3.86229	5.75817	-1.71907
H	-2.96201	5.40234	0.23061
H	-1.85673	5.14257	-1.13043
C	-3.57140	7.26774	-1.76465
H	-3.80099	5.33753	-2.74261
H	-4.90543	5.59150	-1.38202
H	-4.27538	7.79586	-2.43301
H	-3.65766	7.72241	-0.75999
H	-2.54740	7.46623	-2.13217
C	-2.33561	-1.27576	-2.43916
H	-1.44313	-1.64041	-2.97275
H	-3.14892	-1.99526	-2.61194
P	-2.86414	0.39290	-3.19227
C	-4.39155	-0.19979	-4.14994
H	-5.18688	-0.45853	-3.46081
C	-1.65832	0.53752	-4.63318
C	-3.58044	-0.71504	0.14610
C	-4.74581	-1.70136	-0.11157
C	-3.50501	-0.38654	1.65501
H	-3.80604	0.21278	-0.37551
C	-6.08447	-1.09717	0.37160
H	-4.57337	-2.63299	0.42125
H	-4.82787	-1.93802	-1.16864
C	-4.83688	0.21653	2.15443
H	-3.28906	-1.28617	2.22588
H	-2.70368	0.32382	1.83263
C	-6.02426	-0.72591	1.86765
H	-6.88731	-1.81143	0.19954
H	-6.30797	-0.20722	-0.21337
H	-4.77060	0.41173	3.22291
H	-5.00249	1.17037	1.65799
H	-6.95544	-0.24966	2.16729
H	-5.91728	-1.63320	2.45944
C	-1.50686	-3.08713	-0.28733
C	-1.58383	-3.45650	1.21350
C	-2.32115	-4.10970	-1.12124
H	-0.47061	-3.19137	-0.59453
C	-1.10035	-4.90033	1.47640
H	-2.60747	-3.36548	1.56662
H	-0.97751	-2.77041	1.79583
C	-1.84775	-5.55772	-0.85282
H	-3.37908	-4.03792	-0.88673
H	-2.20644	-3.90773	-2.18148
C	-1.91065	-5.91447	0.64548
H	-1.19508	-5.12688	2.53654
H	-0.04614	-4.98259	1.21869
H	-2.46621	-6.24740	-1.42357
H	-0.82481	-5.67245	-1.20527
H	-1.52297	-6.91856	0.80411
H	-2.94687	-5.90746	0.97781
P	0.86707	0.22493	1.89576
C	2.03965	-1.14848	1.33908
H	3.01328	-1.21348	1.84775

H	1.51388	-2.10345	1.50246
C	3.56106	0.19241	-0.95827
C	4.81366	-0.01519	-0.07063
C	3.92693	0.08409	-2.45850
H	3.18783	1.19996	-0.79258
C	5.91272	1.00656	-0.44444
H	5.20971	-1.01940	-0.19268
H	4.56661	0.11125	0.97974
C	5.02476	1.10705	-2.82331
H	4.29623	-0.91208	-2.68442
H	3.03471	0.27030	-3.05694
C	6.27758	0.92531	-1.94106
H	6.79609	0.82386	0.16449
H	5.55990	2.01003	-0.21434
H	5.29178	0.99138	-3.87161
H	4.63589	2.11474	-2.69533
H	7.01481	1.68901	-2.17976
H	6.73113	-0.04199	-2.14916
C	2.29328	-2.68754	-1.16834
C	1.91753	-2.83964	-2.66241
C	3.68979	-3.29466	-0.89000
H	1.57825	-3.26834	-0.59030
C	1.96870	-4.32263	-3.09416
H	2.60198	-2.26708	-3.28101
H	0.92120	-2.44014	-2.83529
C	3.72712	-4.78123	-1.31256
H	4.45253	-2.75687	-1.44528
H	3.93313	-3.21459	0.16749
C	3.34820	-4.94844	-2.79912
H	1.74976	-4.39683	-4.15723
H	1.20021	-4.88136	-2.56388
H	4.72338	-5.18202	-1.13677
H	3.03377	-5.34952	-0.69535
H	3.33789	-6.00484	-3.05916
H	4.10263	-4.46718	-3.41837
C	2.07192	1.60463	2.51443
C	1.33800	2.92227	2.86762
C	3.01927	1.21256	3.67443
H	2.68474	1.79938	1.63649
C	2.35826	4.05678	3.11195
H	0.74606	2.79152	3.76750
H	0.65825	3.20132	2.06738
C	4.05302	2.33583	3.92046
H	2.44816	1.06037	4.58612
H	3.54295	0.28512	3.45619
C	3.35779	3.67943	4.22603
H	1.82803	4.96646	3.38558
H	2.90144	4.26038	2.19162
H	4.69936	2.05627	4.75008
H	4.68191	2.44440	3.03898
H	4.10510	4.46274	4.33323
H	2.82897	3.60169	5.17412
C	-0.04658	-0.52662	3.43360
C	0.79130	-1.49368	4.30813
C	-0.71940	0.54156	4.32853
C	-0.10260	-2.16614	5.37599
H	1.59295	-0.95656	4.80624
H	1.24857	-2.26716	3.69750
C	-1.62515	-0.12863	5.38599
H	0.03775	1.12369	4.84542
H	-1.30921	1.22631	3.72327
C	-0.82352	-1.12151	6.25317
H	0.50889	-2.81466	6.00011
H	-0.84143	-2.79325	4.88079
H	-2.07066	0.63745	6.01711
H	-2.43696	-0.65394	4.88729
H	-1.48932	-1.62469	6.95104
H	-0.08814	-0.57481	6.84030
H	-0.84556	-1.10894	2.98458
H	-0.65902	0.73072	-4.24892
H	-1.63862	-0.35748	-5.24548
H	-1.96109	1.36929	-5.25821
H	-4.19424	-1.06031	-4.78025
H	-4.74908	0.60473	-4.78170

$7a_c$-II

144

XYZ

Ru	-0.13905	0.44380	-0.57899
C	-2.41630	2.64453	-0.35340
C	-1.51297	1.67974	-0.52268
P	-1.76140	-1.24744	-1.07701
N	1.08452	2.13096	-1.26561
C	1.46140	2.12236	-2.57190
C	1.89553	3.53535	-3.01298
C	2.05248	4.29890	-1.68967
C	1.13742	3.49817	-0.72419
O	1.45893	1.13514	-3.35476
P	2.03682	-0.82795	-0.32214
H	-2.59188	2.97982	0.68288
H	0.11908	3.93594	-0.67268
H	1.54389	3.49892	0.30023
H	2.80275	3.49574	-3.63914
H	1.08462	3.95308	-3.64231
H	1.77419	5.36626	-1.74637
H	3.10147	4.24758	-1.34260
H	0.07311	0.26570	-2.16573
C	-3.21543	3.35747	-1.43536
C	-2.93765	4.87672	-1.48989
H	-3.00087	2.89533	-2.41525
H	-4.29944	3.20192	-1.24819
C	-3.79523	5.60613	-2.54190
H	-3.12284	5.32344	-0.49091
H	-1.86346	5.04164	-1.70581

C	-3.51952	7.11805	-2.60197
H	-3.61118	5.15560	-3.53759
H	-4.86814	5.43325	-2.32195
H	-4.14800	7.61436	-3.36345
H	-3.72680	7.60166	-1.62900
H	-2.46243	7.32151	-2.85540
C	-1.91479	-1.32235	-2.95039
H	-0.96236	-1.72401	-3.33401
H	-2.72373	-2.00477	-3.25561
P	-2.29283	0.34366	-3.77034
C	-3.59276	-0.81093	-0.61334
C	-4.67961	-1.81867	-1.06052
C	-3.77269	-0.48995	0.88820
H	-3.74962	0.11421	-1.16370
C	-6.09098	-1.24211	-0.80341
H	-4.57905	-2.74886	-0.50705
H	-4.58275	-2.05170	-2.11720
C	-5.18033	0.08385	1.16429
H	-3.63437	-1.38875	1.48416
H	-3.02625	0.23534	1.19629
C	-6.28556	-0.87900	0.68313
H	-6.84044	-1.97014	-1.10770
H	-6.23165	-0.35304	-1.41485
H	-5.29411	0.27320	2.22981
H	-5.28112	1.03761	0.65063
H	-7.26249	-0.42212	0.82657
H	-6.25985	-1.78770	1.28183
C	-1.41802	-3.13506	-0.71302
C	-1.69234	-3.51434	0.76183
C	-2.09328	-4.16547	-1.65439
H	-0.34770	-3.21818	-0.87739
C	-1.22466	-4.95177	1.08240
H	-2.75613	-3.44133	0.97222
H	-1.18223	-2.82164	1.42360
C	-1.63225	-5.60616	-1.33141
H	-3.17400	-4.11594	-1.56262
H	-1.84271	-3.95226	-2.68875
C	-1.89338	-5.97432	0.14264
H	-1.46116	-5.18750	2.11812
H	-0.14361	-5.01410	0.97293
H	-2.15331	-6.30326	-1.98445
H	-0.56868	-5.69918	-1.54100
H	-1.51213	-6.97224	0.34875
H	-2.96541	-5.98870	0.32914
P	0.45425	0.24782	1.86117
C	1.76543	-1.07212	1.52785
H	2.64580	-1.08087	2.18806
H	1.27030	-2.05227	1.62132
C	3.58319	0.30776	-0.52235
C	4.68933	0.15791	0.55160
C	4.18035	0.20082	-1.94633
H	3.14831	1.30018	-0.43326
C	5.79443	1.21815	0.33672
H	5.13655	-0.83129	0.50842
H	4.27683	0.28482	1.54877
C	5.28333	1.26197	-2.15230
H	4.61614	-0.78246	-2.09906
H	3.38736	0.34773	-2.67943
C	6.38972	1.13673	-1.08407
H	6.57885	1.07586	1.07763
H	5.37317	2.20921	0.49383
H	5.71383	1.14688	-3.14479
H	4.84226	2.25485	-2.10133
H	7.12666	1.92590	-1.21768
H	6.90510	0.18565	-1.20469
C	2.46657	-2.62230	-0.88245
C	2.34477	-2.80288	-2.41510
C	3.81903	-3.17641	-0.37239
H	1.68595	-3.22144	-0.41901
C	2.51362	-4.28781	-2.80856
H	3.10261	-2.21425	-2.92311
H	1.37793	-2.43951	-2.75468
C	3.97447	-4.66504	-0.75917
H	4.64281	-2.61714	-0.80612
H	3.88532	-3.07630	0.70904
C	3.84700	-4.86152	-2.28449
H	2.47212	-4.38134	-3.89166
H	1.68829	-4.86729	-2.39996
H	4.94209	-5.02792	-0.41846
H	3.20994	-5.25130	-0.25280
H	3.91370	-5.92037	-2.52550
H	4.67545	-4.36075	-2.78163
C	1.47657	1.67505	2.67167
C	0.64203	2.96407	2.87512
C	2.21603	1.32174	3.98512
H	2.22874	1.89035	1.91525
C	1.55674	4.13760	3.29219
H	-0.09931	2.81125	3.65263
H	0.11051	3.21603	1.96169
C	3.14454	2.48529	4.40322
H	1.49801	1.14741	4.78165
H	2.80616	0.41584	3.87054
C	2.35342	3.79981	4.57037
H	0.95078	5.02544	3.45979
H	2.24848	4.36309	2.48345
H	3.64309	2.23170	5.33667
H	3.91572	2.61894	3.64708
H	3.03784	4.61225	4.80500
H	1.66627	3.70030	5.40851
C	-0.67901	-0.54405	3.22362
C	0.03431	-1.47738	4.23471
C	-1.53736	0.49521	3.98396
C	-1.00209	-2.18642	5.13735
H	0.71651	-0.90904	4.85982

H	0.61997	-2.23142	3.71650
C	-2.58327	-0.21162	4.87515
H	-0.90356	1.10718	4.61891
H	-2.04182	1.15619	3.28283
C	-1.90321	-1.17252	5.87240
H	-0.48141	-2.81094	5.86046
H	-1.61983	-2.84174	4.52634
H	-3.16006	0.53550	5.41627
H	-3.27646	-0.76852	4.24824
H	-2.65815	-1.70270	6.44902
H	-1.30133	-0.59752	6.57348
H	-1.36472	-1.15820	2.64739
H	-0.94270	0.75130	-4.01448
H	-2.47076	-0.22090	-5.08513

7a$_c$-III

155

XYZ

Ru	0.05848	0.43723	-0.47687
C	-1.16546	3.36300	-0.44433
C	-0.72478	2.10708	-0.49441
P	-2.11290	-0.50777	-0.10624
N	1.52032	1.32071	-1.85726
C	1.48978	0.89994	-3.14247
C	2.23072	1.90040	-4.05380
C	3.00642	2.78550	-3.06714
C	2.19054	2.62770	-1.75596
O	0.93931	-0.14759	-3.58911
P	1.64296	-1.55038	-0.33590
H	-0.94887	3.92550	0.47957
H	1.44137	3.43764	-1.64052
H	2.84635	2.66240	-0.87155
H	2.85193	1.37452	-4.79811
H	1.46436	2.47220	-4.61390
H	3.09988	3.84028	-3.38118
H	4.02926	2.38969	-2.92369
H	-0.33678	-0.05260	-1.95637
C	-1.90638	4.11567	-1.54037
C	-1.15342	5.37460	-2.02585
H	-2.09229	3.42850	-2.38445
H	-2.90314	4.42529	-1.15876
C	-1.93658	6.16843	-3.08922
H	-0.93668	6.03476	-1.16031
H	-0.16933	5.07354	-2.43657
C	-1.18996	7.42070	-3.57889
H	-2.15452	5.50599	-3.95063
H	-2.92133	6.46280	-2.67309
H	-1.77471	7.96937	-4.33930
H	-0.98731	8.11772	-2.74426
H	-0.21723	7.15381	-4.03230
C	-3.45055	0.69337	0.61664
C	-4.84338	0.08170	0.90505

C	-2.96204	1.46188	1.86607
H	-3.56098	1.41860	-0.18712
C	-5.85606	1.19036	1.27345
H	-4.77942	-0.61771	1.73459
H	-5.21538	-0.46528	0.04354
C	-3.96171	2.57453	2.25306
H	-2.85003	0.77989	2.70542
H	-1.99319	1.90754	1.66418
C	-5.37509	2.00399	2.49269
H	-6.82390	0.74096	1.48713
H	-5.98550	1.85572	0.42218
H	-3.61253	3.08279	3.14966
H	-3.99847	3.31155	1.45358
H	-6.07226	2.81473	2.69360
H	-5.36146	1.36107	3.37093
C	-2.31170	-2.27289	0.71049
C	-3.48322	-2.32593	1.72027
C	-2.38562	-3.49296	-0.24376
H	-1.39032	-2.37721	1.27562
C	-3.50502	-3.65354	2.51052
H	-4.42989	-2.21916	1.19737
H	-3.40508	-1.50075	2.42046
C	-2.41538	-4.82287	0.54576
H	-3.27222	-3.43778	-0.86799
H	-1.52258	-3.51009	-0.90159
C	-3.57688	-4.86332	1.55834
H	-4.35841	-3.65944	3.18574
H	-2.60671	-3.72738	3.12052
H	-2.50542	-5.65150	-0.15366
H	-1.47359	-4.94758	1.07607
H	-3.53872	-5.78831	2.12983
H	-4.52519	-4.84718	1.02478
P	1.27809	0.46003	1.73707
C	1.87353	-1.32108	1.52276
H	2.84904	-1.57820	1.96221
H	1.11682	-1.97196	1.98998
C	3.35462	-1.20390	-1.15696
C	4.60515	-1.59720	-0.33146
C	3.42675	-1.80145	-2.58311
H	3.34213	-0.12221	-1.26450
C	5.89308	-1.14103	-1.05583
H	4.64629	-2.67261	-0.18333
H	4.57863	-1.13457	0.65121
C	4.71594	-1.34231	-3.29871
H	3.42700	-2.88667	-2.53662
H	2.55439	-1.47617	-3.15041
C	5.97321	-1.71478	-2.48547
H	6.76090	-1.45566	-0.47929
H	5.90970	-0.05390	-1.10321
H	4.76454	-1.80096	-4.28405
H	4.68466	-0.26459	-3.44205
H	6.86362	-1.33552	-2.98249

H	6.06492	-2.79813	-2.43421
C	1.24340	-3.43450	-0.43902
C	0.61168	-3.82370	-1.79773
C	2.40955	-4.38880	-0.08455
H	0.48287	-3.57022	0.32650
C	0.13416	-5.29340	-1.78161
H	1.33479	-3.69549	-2.59750
H	-0.22651	-3.16688	-2.01722
C	1.91968	-5.85479	-0.05741
H	3.20437	-4.30217	-0.81949
H	2.82786	-4.13075	0.88619
C	1.28099	-6.25460	-1.40412
H	-0.25976	-5.55575	-2.76119
H	-0.67687	-5.40319	-1.06461
H	2.75809	-6.51194	0.16463
H	1.18978	-5.97685	0.74068
H	0.90429	-7.27353	-1.34414
H	2.04105	-6.23287	-2.18263
C	2.92993	1.45751	1.86241
C	2.70189	2.98974	1.86972
C	3.87459	1.06888	3.02581
H	3.43230	1.20787	0.92993
C	4.04738	3.73464	1.71933
H	2.24156	3.29523	2.80354
H	2.02907	3.27457	1.06565
C	5.22951	1.79918	2.87834
H	3.43011	1.35051	3.97637
H	4.04675	-0.00435	3.04881
C	5.03812	3.32989	2.83163
H	3.87186	4.80770	1.75511
H	4.48107	3.50755	0.74785
H	5.87615	1.53211	3.71183
H	5.71945	1.46625	1.96542
H	5.99798	3.81445	2.66613
H	4.66125	3.67333	3.79317
C	0.42668	0.46769	3.48143
C	1.04909	-0.47935	4.53874
C	0.26542	1.88658	4.07769
C	0.16811	-0.52200	5.80904
H	2.04479	-0.14214	4.81091
H	1.14358	-1.48747	4.14515
C	-0.62727	1.84755	5.33841
H	1.23621	2.28462	4.35770
H	-0.17006	2.55952	3.34248
C	-0.04805	0.88745	6.39816
H	0.63766	-1.16501	6.55072
H	-0.79619	-0.96134	5.56107
H	-0.70964	2.84996	5.75324
H	-1.62906	1.52445	5.06334
H	-0.71993	0.82959	7.25182
H	0.90241	1.27777	6.75685
H	-0.57299	0.10054	3.26951

C	-2.84483	-0.86172	-1.73455
C	-4.26646	-1.35460	-1.55621
C	-2.79997	0.38583	-2.59371
H	-2.22821	-1.66687	-2.21380
C	-4.94313	-1.58295	-2.89244
H	-4.85100	-0.60019	-0.96669
H	-4.26174	-2.30997	-0.96981
C	-3.47595	0.15721	-3.93027
H	-3.31125	1.22726	-2.05618
H	-1.73388	0.68999	-2.75920
C	-4.89716	-0.33689	-3.75291
H	-6.00960	-1.88563	-2.72653
H	-4.43318	-2.42566	-3.42926
H	-3.48110	1.11265	-4.51649
H	-2.89036	-0.59658	-4.51963
H	-5.34629	-0.55666	-4.75620
H	-5.51527	0.46857	-3.27593

$7a_c$-IV

142

XYZ

Ru	-0.01698	0.66136	-0.56484
C	-1.45460	3.48887	-0.66581
C	-0.88681	2.28563	-0.59365
P	-2.11248	-0.22778	-1.31056
N	1.77414	1.87188	-0.85469
C	2.32530	1.87165	-2.09816
C	3.26337	3.08723	-2.26168
C	3.45181	3.59723	-0.82530
C	2.17423	3.07823	-0.11338
O	2.12388	1.03438	-3.01494
P	1.57542	-1.31830	-0.49579
H	-1.84314	3.93344	0.26494
H	1.36325	3.83606	-0.13418
H	2.37249	2.84347	0.94429
H	4.19291	2.80451	-2.78362
H	2.74384	3.82792	-2.90170
H	3.56596	4.69290	-0.74301
H	4.34837	3.13397	-0.37214
H	0.26221	0.64420	-2.14751
C	-1.56626	4.33260	-1.93024
C	-0.90092	5.72018	-1.79063
H	-1.11058	3.78647	-2.77675
H	-2.63781	4.47792	-2.18636
C	-1.05347	6.59156	-3.05201
H	-1.33966	6.25158	-0.92086
H	0.17371	5.58436	-1.55849
C	-0.39267	7.97344	-2.91291
H	-0.61613	6.05740	-3.91909
H	-2.13099	6.71725	-3.28144
H	-0.51723	8.57551	-3.83103
H	-0.83293	8.54499	-2.07453

H	0.69181	7.87967	-2.71804
C	-2.25044	0.23822	-3.13880
H	-1.50426	-0.29726	-3.71142
H	-3.23286	0.02571	-3.54143
C	-3.69637	0.70810	-0.68995
C	-5.05890	0.20322	-1.22348
C	-3.75279	0.83506	0.84861
H	-3.53248	1.70698	-1.08738
C	-6.19527	1.16385	-0.80301
H	-5.27637	-0.78505	-0.82658
H	-5.04674	0.12809	-2.30722
C	-4.88812	1.78812	1.28433
H	-3.91683	-0.14075	1.29977
H	-2.80425	1.21849	1.21322
C	-6.25228	1.32858	0.72961
H	-7.14594	0.78190	-1.16989
H	-6.03306	2.13535	-1.26558
H	-4.92799	1.83557	2.37073
H	-4.66911	2.79024	0.92155
H	-7.02166	2.05065	0.99483
H	-6.52656	0.37831	1.18410
C	-2.46281	-2.13807	-1.39349
C	-2.90224	-2.71371	-0.02574
C	-3.42356	-2.62068	-2.50976
H	-1.48564	-2.55238	-1.62399
C	-2.99259	-4.25600	-0.05751
H	-3.87385	-2.31338	0.25214
H	-2.19748	-2.41622	0.74511
C	-3.51319	-4.16426	-2.53817
H	-4.41969	-2.21666	-2.35883
H	-3.07425	-2.27790	-3.47921
C	-3.94428	-4.73477	-1.17203
H	-3.33830	-4.61889	0.90841
H	-2.00145	-4.67271	-0.22618
H	-4.22199	-4.46808	-3.30573
H	-2.54385	-4.57681	-2.81010
H	-3.95151	-5.82203	-1.21019
H	-4.95769	-4.40878	-0.94611
P	0.27783	-0.15951	1.84803
C	1.06126	-1.77574	1.26021
H	1.80868	-2.23810	1.92193
H	0.23712	-2.49708	1.13737
C	3.44541	-0.84542	-0.41219
C	4.28762	-1.58699	0.65614
C	4.11944	-0.93979	-1.80253
H	3.40502	0.20780	-0.14485
C	5.72562	-1.01942	0.69911
H	4.33380	-2.65054	0.43819
H	3.84463	-1.47589	1.64164
C	5.55272	-0.36780	-1.74987
H	4.17397	-1.97563	-2.12436
H	3.52230	-0.37790	-2.52171

C	6.40545	-1.08678	-0.68378
H	6.31020	-1.57613	1.42910
H	5.68981	0.01619	1.03170
H	6.01903	-0.47215	-2.72726
H	5.50932	0.69478	-1.52132
H	7.39297	-0.63349	-0.62832
H	6.54042	-2.12858	-0.96859
C	1.36576	-3.00102	-1.40807
C	1.34188	-2.82879	-2.94636
C	2.35399	-4.11882	-0.99500
H	0.37537	-3.32823	-1.09987
C	0.98751	-4.16202	-3.64286
H	2.31128	-2.49150	-3.30040
H	0.61896	-2.06423	-3.22121
C	1.98576	-5.45059	-1.68829
H	3.36777	-3.84947	-1.27623
H	2.33737	-4.25810	0.08404
C	1.95245	-5.29092	-3.22288
H	1.02514	-4.02709	-4.72172
H	-0.03085	-4.44654	-3.38585
H	2.70903	-6.21481	-1.41087
H	1.01047	-5.78257	-1.33721
H	1.64743	-6.22838	-3.68319
H	2.95383	-5.06163	-3.58191
C	1.67316	0.65849	2.90447
C	1.29967	2.08788	3.37069
C	2.18951	-0.16031	4.11264
H	2.49036	0.75206	2.19179
C	2.52911	2.79106	3.98850
H	0.51487	2.04434	4.11893
H	0.92172	2.67009	2.53479
C	3.42888	0.52694	4.73105
H	1.41619	-0.23029	4.87263
H	2.45160	-1.17297	3.81653
C	3.10927	1.97388	5.16247
H	2.24240	3.78151	4.33546
H	3.29323	2.92143	3.22524
H	3.77021	-0.04841	5.58933
H	4.23634	0.53521	4.00153
H	4.01179	2.45297	5.53592
H	2.38954	1.95369	5.97866
C	-1.12482	-0.76898	3.04533
C	-0.80922	-2.05443	3.85245
C	-1.63744	0.33384	4.00270
C	-2.06707	-2.53697	4.61179
H	-0.01512	-1.86720	4.56898
H	-0.47254	-2.85047	3.19472
C	-2.90249	-0.13956	4.75334
H	-0.87283	0.57091	4.73663
H	-1.85961	1.24407	3.45072
C	-2.62730	-1.43849	5.53887
H	-1.81765	-3.42127	5.19474

H	-2.83059	-2.82624	3.89222
H	-3.23132	0.64218	5.43486
H	-3.70564	-0.30962	4.03949
H	-3.54366	-1.78731	6.01018
H	-1.90965	-1.23519	6.33134
H	-1.94009	-1.00853	2.36849
H	-2.04918	1.29725	-3.23128

$7a_c$-V

116

XYZ

Ru	0.75810	-0.43535	-0.56991
C	3.93662	-0.39632	-0.55386
C	2.60607	-0.37440	-0.56187
P	0.95287	-0.12740	-2.88828
N	0.94456	-1.38113	1.35318
C	0.66688	-2.70628	1.46753
C	1.24373	-3.24499	2.79769
C	1.58995	-1.97190	3.58602
C	1.81421	-0.93707	2.45405
O	0.04853	-3.42684	0.64408
P	-1.75522	-0.59315	-0.32579
H	4.47103	0.52708	-0.27738
H	2.87643	-0.92267	2.13034
H	1.56290	0.08948	2.77015
H	0.52876	-3.92318	3.29301
H	2.14634	-3.84140	2.55704
H	2.46706	-2.07160	4.25031
H	0.72908	-1.66575	4.21004
H	0.71943	-2.00112	-0.96335
C	4.78598	-1.63044	-0.83472
C	5.69261	-2.02230	0.35350
H	4.12366	-2.47802	-1.09067
H	5.42430	-1.44440	-1.72483
C	6.58087	-3.24510	0.05457
H	6.33391	-1.15899	0.62689
H	5.05842	-2.22845	1.23791
C	7.48429	-3.63631	1.23626
H	5.93698	-4.10448	-0.21967
H	7.20638	-3.03388	-0.83632
H	8.10830	-4.51552	0.99431
H	8.16373	-2.80822	1.51195
H	6.88480	-3.88664	2.13110
C	1.46073	-1.71858	-3.76862
H	0.69598	-2.47289	-3.63352
H	1.60686	-1.53859	-4.82822
C	2.33211	1.00297	-3.52140
H	3.23795	0.81036	-2.96094
C	-0.41554	0.41921	-4.08613
H	-1.17307	-0.34991	-4.15991
P	-0.18122	1.78191	0.09921
C	-1.93993	1.28970	-0.38575
H	-2.77222	1.75494	0.16166
H	-2.05578	1.55648	-1.45003
C	-2.42894	-1.15247	1.39406
C	-3.64102	-0.36012	1.94484
C	-2.71732	-2.67302	1.41575
H	-1.58010	-0.97340	2.05071
C	-3.98441	-0.83036	3.37748
H	-4.51056	-0.50216	1.30941
H	-3.42864	0.70421	1.97196
C	-3.06035	-3.13726	2.84768
H	-3.55733	-2.90211	0.76631
H	-1.83154	-3.20132	1.05544
C	-4.25357	-2.34845	3.42666
H	-4.85589	-0.28547	3.73542
H	-3.15517	-0.59008	4.04005
H	-3.29196	-4.20018	2.83636
H	-2.19190	-3.00051	3.48855
H	-4.43942	-2.65370	4.45430
H	-5.15045	-2.57338	2.85252
C	-2.93115	-1.17236	-1.73152
C	-2.63494	-2.62731	-2.16887
C	-4.44510	-0.97648	-1.47651
H	-2.66293	-0.52056	-2.55927
C	-3.45040	-2.99495	-3.42911
H	-2.88777	-3.31670	-1.36869
H	-1.57225	-2.74520	-2.36674
C	-5.25627	-1.33651	-2.74240
H	-4.77408	-1.61244	-0.65965
H	-4.65221	0.05455	-1.19667
C	-4.96204	-2.77977	-3.20330
H	-3.26126	-4.03360	-3.69176
H	-3.12132	-2.38244	-4.26656
H	-6.31860	-1.22309	-2.53582
H	-5.00396	-0.64209	-3.54164
H	-5.50677	-2.99015	-4.12132
H	-5.31594	-3.47728	-2.44667
C	-0.30281	2.27740	1.96606
C	1.01270	2.91095	2.48014
C	-1.50039	3.16503	2.38759
H	-0.41921	1.31329	2.45722
C	0.98526	3.04187	4.01929
H	1.13525	3.90137	2.04945
H	1.86671	2.30939	2.17783
C	-1.54687	3.29079	3.92828
H	-1.40691	4.15794	1.95908
H	-2.44025	2.74768	2.03960
C	-0.23004	3.87042	4.48625
H	1.90547	3.51278	4.35857
H	0.94254	2.04981	4.46402
H	-2.38134	3.92895	4.21202
H	-1.72420	2.30900	4.36288
H	-0.26894	3.88902	5.57326

H	-0.11785	4.89829	4.14638
C	0.15790	3.33003	-0.99658
C	-0.77510	4.54370	-0.77251
C	1.63767	3.78204	-0.94372
C	-0.51605	5.62571	-1.84556
H	-0.59030	4.97755	0.20591
H	-1.81853	4.23746	-0.80770
C	1.91176	4.87238	-2.00465
H	1.86874	4.18478	0.03793
H	2.29556	2.93179	-1.10839
C	0.96026	6.07584	-1.83443
H	-1.16459	6.47994	-1.66216
H	-0.76846	5.22920	-2.82717
H	2.94465	5.20419	-1.92308
H	1.78317	4.45130	-2.99967
H	1.13132	6.79614	-2.63158
H	1.17565	6.57529	-0.89183
H	-0.03226	2.95601	-2.00121
H	2.38291	-2.08487	-3.33493
H	-0.00495	0.58683	-5.07490
H	-0.88308	1.33641	-3.74988
H	2.05425	2.04075	-3.39346
H	2.51649	0.81986	-4.57350

7a$_c$-VI

64

XYZ

Ru	0.30512	-0.30919	-0.12646
C	-2.52265	-1.15302	1.08140
C	-1.31011	-0.81327	0.64417
P	0.81395	-2.58162	-0.33108
N	-0.49101	1.68067	-0.30940
C	-0.41455	2.56186	0.72260
C	-0.73499	3.98724	0.20573
C	-1.36214	3.73849	-1.17632
C	-0.74801	2.37152	-1.57734
O	-0.13863	2.30688	1.92141
P	2.32931	0.63845	-1.25086
H	-2.68077	-1.21978	2.17044
H	0.19382	2.50802	-2.15048
H	-1.42169	1.77267	-2.22148
H	-1.37883	4.53153	0.91772
H	0.21752	4.54935	0.13185
H	-1.15864	4.53155	-1.91831
H	-2.46109	3.64459	-1.08121
H	-0.35654	-0.47995	-1.59246
C	-3.73039	-1.39451	0.18424
C	-4.88751	-0.40279	0.43960
H	-3.41254	-1.32651	-0.87303
H	-4.10685	-2.42868	0.33943
C	-6.12245	-0.67960	-0.43901
H	-5.17901	-0.44537	1.50941
H	-4.52275	0.62738	0.25908
C	-7.27491	0.30766	-0.18886
H	-5.82577	-0.63918	-1.50632
H	-6.47526	-1.71527	-0.25797
H	-8.14476	0.08699	-0.83375
H	-7.61694	0.26528	0.86215
H	-6.96011	1.34763	-0.39427
C	0.04713	-3.42743	-1.81767
H	0.19671	-4.48430	-1.74322
H	-1.00154	-3.21676	-1.84631
C	0.17621	-3.67804	1.03702
H	-0.89782	-3.47678	1.18113
C	2.55617	-3.25060	-0.44401
H	3.07918	-2.82470	-1.31546
P	2.02607	0.26750	1.51660
C	3.42471	0.33207	0.26031
H	4.22711	1.07012	0.45574
H	3.87368	-0.67513	0.18806
C	2.53139	2.46927	-1.47980
H	2.02752	3.00313	-0.65879
C	3.26003	0.02387	-2.73487
H	3.28993	-1.07711	-2.75655
C	2.07370	1.95131	2.31215
H	1.74336	2.70521	1.57943
C	2.66077	-0.77770	2.92576
H	2.80059	-1.81946	2.59102
H	3.61833	-0.39312	3.32200
H	1.91278	-0.77846	3.73826
H	2.54503	-4.35112	-0.54999
H	3.11664	-2.99524	0.47192
H	0.69743	-3.42506	1.97703
H	0.33469	-4.74794	0.81067
H	2.70445	0.35684	-3.62851
H	4.28504	0.43487	-2.77219
H	2.02340	2.75126	-2.41495
H	3.60201	2.74246	-1.51011
H	1.36573	1.96954	3.15910
H	3.08398	2.20721	2.68093
H	0.50848	-3.06404	-2.71210
